# The chromosome-scale genome of *Magnolia sinica* (Magnoliaceae) provides insights into the conservation of plant species with extremely small populations (PSESP)

**DOI:** 10.1093/gigascience/giad110

**Published:** 2024-01-11

**Authors:** Lei Cai, Detuan Liu, Fengmao Yang, Rengang Zhang, Quanzheng Yun, Zhiling Dao, Yongpeng Ma, Weibang Sun

**Affiliations:** Yunnan Key Laboratory for Integrative Conservation of Plant Species with Extremely Small Populations/Key Laboratory for Plant Diversity and Biogeography of East Asia, Kunming Institute of Botany, Chinese Academy of Sciences, Kunming, 650201, Yunnan, China; Yunnan Key Laboratory for Integrative Conservation of Plant Species with Extremely Small Populations/Key Laboratory for Plant Diversity and Biogeography of East Asia, Kunming Institute of Botany, Chinese Academy of Sciences, Kunming, 650201, Yunnan, China; University of Chinese Academy of Sciences, 100049 Beijing, China; Yunnan Key Laboratory for Integrative Conservation of Plant Species with Extremely Small Populations/Key Laboratory for Plant Diversity and Biogeography of East Asia, Kunming Institute of Botany, Chinese Academy of Sciences, Kunming, 650201, Yunnan, China; University of Chinese Academy of Sciences, 100049 Beijing, China; Yunnan Key Laboratory for Integrative Conservation of Plant Species with Extremely Small Populations/Key Laboratory for Plant Diversity and Biogeography of East Asia, Kunming Institute of Botany, Chinese Academy of Sciences, Kunming, 650201, Yunnan, China; University of Chinese Academy of Sciences, 100049 Beijing, China; Department of Bioinformatics, Ori (Shandong) Gene Science and Technology Co., Ltd., Weifang, 261000, Shandong, China; Yunnan Key Laboratory for Integrative Conservation of Plant Species with Extremely Small Populations/Key Laboratory for Plant Diversity and Biogeography of East Asia, Kunming Institute of Botany, Chinese Academy of Sciences, Kunming, 650201, Yunnan, China; Yunnan Key Laboratory for Integrative Conservation of Plant Species with Extremely Small Populations/Key Laboratory for Plant Diversity and Biogeography of East Asia, Kunming Institute of Botany, Chinese Academy of Sciences, Kunming, 650201, Yunnan, China; Yunnan Key Laboratory for Integrative Conservation of Plant Species with Extremely Small Populations/Key Laboratory for Plant Diversity and Biogeography of East Asia, Kunming Institute of Botany, Chinese Academy of Sciences, Kunming, 650201, Yunnan, China

**Keywords:** *Magnolia sinica*, PSESP, genome sequencing, deleterious mutation, demographic history, conservation

## Abstract

*Magnolia sinica* (Magnoliaceae) is a highly threatened tree endemic to southeast Yunnan, China. In this study, we generated for the first time a high-quality chromosome-scale genome sequence from *M. sinica*, by combining Illumina and ONT data with Hi-C scaffolding methods. The final assembled genome size of *M. sinica* was 1.84 Gb, with a contig N50 of ca. 45 Mb and scaffold N50 of 92 Mb. Identified repeats constituted approximately 57% of the genome, and 43,473 protein-coding genes were predicted. Phylogenetic analysis shows that the magnolias form a sister clade with the eudicots and the order Ceratophyllales, while the monocots are sister to the other core angiosperms. In our study, a total of 21 individuals from the 5 remnant populations of *M. sinica*, as well as 22 specimens belonging to 8 related Magnoliaceae species, were resequenced. The results showed that *M. sinica* had higher genetic diversity (*θ*w = 0.01126 and *θ*π = 0.01158) than other related species in the Magnoliaceae. However, population structure analysis suggested that the genetic differentiation among the 5 *M. sinica* populations was very low. Analyses of the demographic history of the species using different models consistently revealed that 2 bottleneck events occurred. The contemporary effective population size of *M. sinica* was estimated to be 10.9. The different patterns of genetic loads (inbreeding and numbers of deleterious mutations) suggested constructive strategies for the conservation of these 5 different populations of *M. sinica*. Overall, this high-quality genome will be a valuable genomic resource for conservation of *M. sinica*.

## Introduction

The reduction of species diversity is of global concern and has been closely linked with climate change and human activity. The conservation of biodiversity is therefore a hot topic [1–6]. The resolution of the recently convened CBD COP 15 (15th Conference of the Parties, Convention on Biological Diversity) supports biodiversity conservation issues of global concern, and one of the goals (so-called “30 × 30”) requires that at least 30% of the land, fresh water, and oceans on Earth be protected in some form by 2030. In addition, identification of geographic areas with high concentrations of endemic and rare species diversity is an important step in protecting biodiversity [7]. The mountains of Southwest China are one of the world’s biodiversity hotspots and also affected by climate change and human disturbance, meaning that it is also an area at very high risk of species extinction [8, 9]. The study and protection of the threatened species in this region are therefore of particular importance and urgency [10, 11]. In order to rescue the most highly threatened species and reduce their risks of extinction in this region, Chinese scholars put forward the concept of plant species with extremely small populations (PSESP) in 2005, according to China’s current national conditions and the practice of biodiversity protection [12–15]. That a species is threatened by human activities and interference is a necessary qualifying condition to determine whether that species meets the definition of PSESP, and human activities are also of significance when implementing rescuing protection for PSESP [12, 16].

Plant genome sequencing has grown rapidly in the past 20 years, and by the end of June 2023, the genome sequences of more than 1,000 higher plant taxa had been published [17]. Sequenced genomes can provide insights and evidence to better understand the genome biology and evolution of plants [18, 19]. Although the genomes of so many plant species have been studied, only a few studies have sequenced the genomes of threatened plant species (examples include *Acer yangbiense, Acanthochlamys bracteata, Beta patula, Cercidiphyllum japonicum, Davidia involucrata, Dracaena cambodiana, Ginkgo biloba, Kingdonia uniflora, Malania oleifera, Ostrya rehderiana*, and *Rhododendron griersonianum*) in order to focus on the conservation of these species [20–30].

Plant species in the family Magnoliaceae are hugely important in gardens and horticulture across the world [31, 32]. The Magnoliaceae is also one of the most highly threatened angiosperm groups. There are more than 300 species in this family, which are mainly distributed intermittently in the temperate, subtropical, and tropical regions of East and Southeast Asia, eastern North America, and Central and South America [33–35]. About 120 species of Magnoliaceae are known from China, and Southwest and South China are the centers of diversity for this family [36]. Global conservation assessments suggest that 147 magnoliaceous species are facing threats, accounting for 48% of the total assessed species in this family [35]. Similarly, 76 species of Chinese Magnoliaceae are threatened, representing more than 50% of the total number of threatened Magnoliaceae species globally [37]. At present, in-depth genome research has been conducted in only 4 species in the Magnoliaceae (*Liriodendron chinense, Magnolia biondii, Magnolia obovata*, and *Magnolia officinalis*), mainly to investigate the controversial evolutionary position of the magnoliids [38–41].

The evergreen tree *Magnolia sinica* (Law) Noot. (NCBI:txid86752) (Magnoliaceae) is a typical PSESP endemic to southeast Yunnan, where many threatened species are in urgent need of rescue and protection [12, 14]. In China, the species is often referred to as *Manglietiastrum sinicum* Y.W. Law and is known as Huagaimu in Chinese [34, 36, 42, 43]. It has been categorized as Critically Endangered on the *China Species Red List* [44], *The Red List of Magnoliaceae* [35, 45], and *The Threatened Species List of China's Higher Plants* [37]. *M. sinica* was proposed as a first-rank plant for national key protection in 1999 [46] and also in 2021 [47], and it was listed as 1 of 62 PSESP in Yunnan in 2010 and also as 1 of the 120 national PSESP of China in 2012, requiring the most urgent rescue conservation [14, 15]. Recent survey data revealed only 52 individuals remain in the wild, and comprehensive conservation research and protective action of *M. sinica* have been implemented, including reproductive and seed biology, genetic diversity studies based on SSR (Simple Sequence Repeat), sequencing of the chloroplast genome, *in situ* conservation, *ex situ* conservation, and reintroduction programs [48–53]. Although a great deal of protection and research action has been carried out, the lack of natural regeneration and genetic rescue still limits the protection of *M. sinica*. Therefore, the formulation of genetic rescue strategies for *M. sinica* will benefit greatly from the exploration of harmful cumulative mutations, population historical dynamics, and effective population size from the whole-genome level.

Here, we report a high-quality chromosome-scale genome sequence of *Magnolia sinica* and compare it with other relevant published genomic data. By exploring the evolution of the genome, as well as the genetic characteristics, demographic history, and genetic load of *M. sinica*, we have identified genomic factors that may contribute to the threats to this species, and on the basis of this, we propose further strategies for the conservation of *M. sinica*.

## Materials and Methods

### Collection of plant material

**Figure 1:**
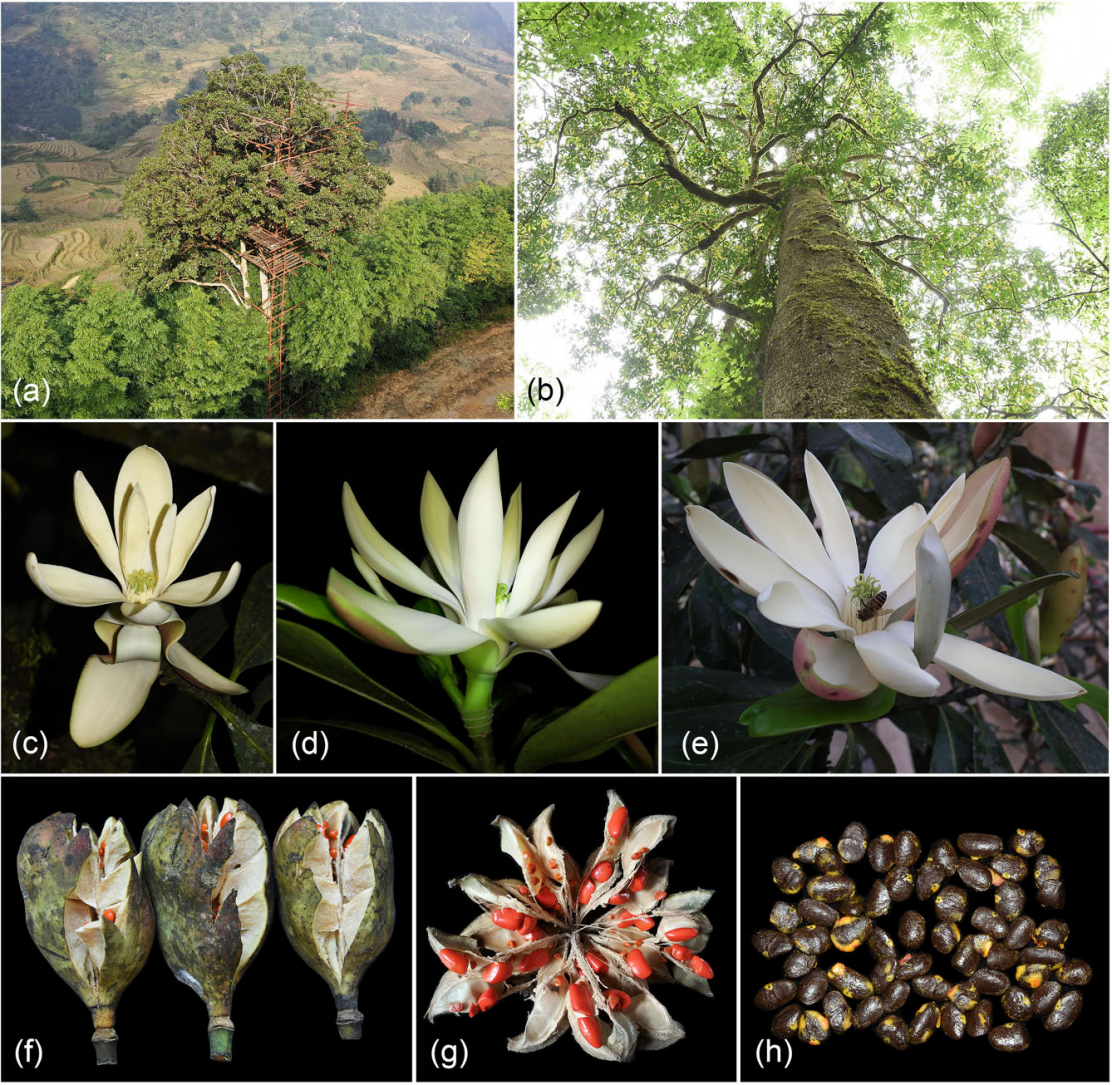
Habitat and morphological characters of *Magnolia sinica*. (A) Habitat severely affected by human interference. (B) Habit. (C–E) Flowers of different individuals. (F) Fruits. (G) Fruit completely opened. (H) Seeds without testa.

*Magnolia sinica* is only found scattered in several counties in southeast Yunnan (Fig. 1). Fresh young leaf material was collected for whole-genome sequencing from a single individual. This individual is conserved and growing *ex situ* at the Kunming Botanical Garden (KBG) but was originally introduced from Xichou County, southeast Yunnan. For transcriptome sequencing, leaf, stem and root samples were obtained from a 3-year-old seedling also at KBG, and fresh fruits were collected from the wild in Jinping County, Yunnan. Fresh leaves used for genome library preparation and other tissues used for transcriptome sequencing were immediately frozen in liquid nitrogen and stored at −78.5°C in dry ice until DNA or RNA extraction. The remaining 21 leaf samples for resequencing were collected from the original species habitat in Xichou, Maguan, and Jinping Counties from 2017 to 2019 (Supplementary Table S1). Other DNA materials from 8 further species in the Magnoliaceae was used for comparison of genetic diversity and investigation of the phylogenic relationships. These DNA materials were collected from specimens cultivated at KBG and the Germplasm Bank of Wild Species, Chinese Academy of Sciences (Supplementary Table S2). After the leaves were collected, they were quickly packed in silica gel desiccant and stored in silica gel until resequencing.

### Genome sequencing

Genomic DNA sequencing was performed using different sequencing platforms simultaneously to ensure accurate assembly. (1) For ONT (Oxford Nanopore Technologies) PromethION sequencing, total DNA was extracted using the cetyltrimethylammonium bromide (CTAB) method [54] using a genomic DNA extraction kit (cat. 13323, Qiagen). A NanoDrop One UV-Vis spectrophotometer (Thermo Fisher Scientific) was then used to check DNA purity and a Qubit 3.0 Fluorometer (Invitrogen) was used to accurately quantify the DNA. After purification, the adapters from the LSK109 Ligation kit (cat. SQK-LSK109; Oxford) were used for the ligation reaction, and finally the Qubit 3.0 Fluorometer (Invitrogen) was used to quantify the constructed DNA library. The DNA library was subsequently transferred to Nanopore PromethION (ONT) for sequencing 7 flow cells. (2) For Illumina sequencing, short-insert libraries were prepared using 2 μg genomic DNA, and 3 Illumina PCR-free libraries of 300 to 500 bp insertion size were constructed according to the standard manufacturer’s protocol using the DNAseq Library Index Kit (Hangzhou Kaitai Biotechnology, Co., Ltd.). The whole-genomic libraries were sequenced on an Illumina Hiseq X Ten platform (RRID:SCR_020131). (3) The Hi-C library was prepared by Beijing Ori-Gene Science and Technology Co., Ltd. High molecular weight genomic DNA (≥700 ng) was cross-linked *in situ*, extracted, and then digested with a restriction enzyme. The DNA ends were then marked with biotin-14-dCTP, and the crosslinked fragments were blunt-end ligated. Fragments were sheared to a size of 200 to 600 bp with sonication. The Hi-C libraries were amplified using 12 to 14 cycles of PCR and sequenced in the Illumina HiSeq X Ten platform. (4) Transcriptome sequencing was performed on a PacBio Sequel (Pacific Biosciences) platform (RRID:SCR_017989) using full-length isoform sequencing (iso-seq) [55]. High-quality RNA was extracted with a Qiagen kit while a series of RNA samples were tested: Nanodrop was used to assess RNA purity, Qubit was used to precisely quantify the RNA, and an Agilent 2100 Bioanalyzer was used to calculate RIN values and 28S/18S. Then, a SMARTer PCR cDNA synthesis kit (Clontech,Princeton, NJ, USA) was used to reverse transcribe the RNA into cDNA, the reverse transcription products were amplified using KAPA HiFi PCR kits (Roche Diagnostics, Switzerland), and the amplified products were used to construct a SMRTbell library using a SMRTbell template prep kit 1.0. The third-generation sequencer Sequel (Pacific Biosciences) was used to sequence the full-length cDNA to obtain high-quality transcriptome sequencing data.

### Genome assembly

We obtained ∼203 Gb (∼100×) ONT reads, ∼215 Gb (∼110×) Illumina Hiseq reads, ∼222 Gb Hi-C reads, and ∼24 Gb iso-seq reads (Supplementary Tables S3–S6). The *de novo* genome assembly was first performed upon ONT reads using different assembly strategies. Briefly, the long noisy ONT reads were first corrected with NextDenovo [56] and then assembled with SMARTDENOVO (RRID:SCR_017622) [57] and WTDBG (assembly v0.2), respectively [58] (Supplementary Tables S7–S9). Primary assembly v0.1 was selected as the optimal assembly due to the low error rate. Then, the Illumina sequencing reads were used to improve base-level accuracy of the assembly with Pilon [59]. The 2 draft assemblies (v0.1 as reference and v0.2 as query) were then merged using QuickMerge to improve continuity [60] and then polished again using pilon (Supplementary Tables S10–S12). The GetOrganelle software was used to assemble the mitochondrial (parameters: -R 50 -k 67,87,107,127 -F embplant_mt -w 125) and chloroplast (-R 15 -k 67,87,107,127 -F embplant_pt -w 125) genomes, respectively, and Bandage was used for manually adjustment [61, 62].

Hi-C reads were mapped to the draft assembly with Juicer, and a candidate chromosome-length assembly was generated automatically using the 3D-DNA pipeline to correct misjoins, order, and orientation and to anchor contigs [63, 64]. Manual review and refinement of the candidate assembly was performed in Juicebox Assembly Tools (JBAT) for quality control and interactive correction [65]. To reduce the influence of chromosome interactions and to further improve the chromosome-scale assembly, each chromosome was separately rescaffolded with 3D-DNA and then manually refined with Juicebox (RRID:SCR_021172). Finally, the chromosomal and unanchored sequences were generated, with the gap length set as 100 bp.

**Figure 2:**
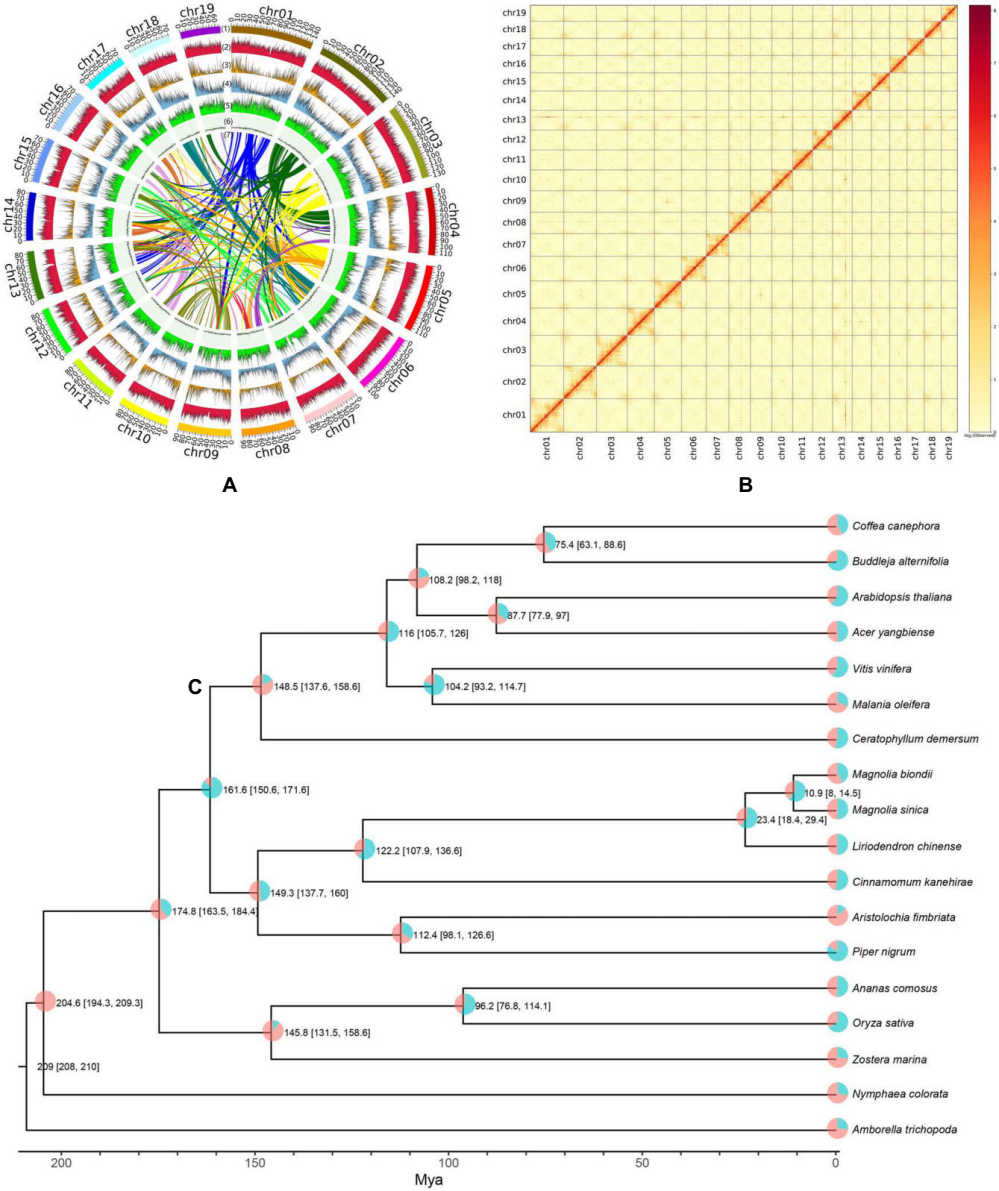
Genomic character and genome evolution of *Magnolia sinica*. (A) The genome features across 19 chromosomes of *M. sinica*. (1) Nineteen pseudochromosomes. (2) Class I transposable element (TE) density (including long terminal repeats [LTRs], long and short interspersed nuclear elements). (3) Class II TE (DNA and Heliron) density. (4) Coding gene (messenger RNA) density. (5) The density of single-nucleotide polymorphism (SNP) loci. (6) GC content. (7) collinear blocks. (B) Hi-C interaction heatmap for the *M. sinica* genome showing interactions among 19 chromosomes. (C) The phylogenetic tree of 18 species showing the proportions of the gene families that contracted and expanded (pink: contracted; blue-green: expanded; values at the nodes represent the time of differentiation and 95% CI).

To fill the assembly gaps, LR_Gapcloser (default parameters) was run for 2 rounds based on ONT reads, and then NextPolish (default parameters) was run for 3 rounds to polish the assembly based on Illumina reads [66, 67]. In order to eliminate redundancy and external source pollution: (i) Redundant was used to remove the redundant unanchored sequences (identity ≥0.98) [68]; (ii) unplaced contigs with a length of less than 5 kb were removed; (iii) the assembly was aligned with the NT database [69] using BLASTN, combined with coverage depth and GC content, to determine whether there was contamination from other species; and (iv) haplotigs or fragments with low average coverage depth (less than 75% of the peak depth) were removed with manual curation. The chromosomes were coded as chr01–chr19 according to their lengths (from long to short) (Fig. 2A, B). The numbers, lengths, and proportions of the chromosomes, unanchored sequences, and chloroplast and mitochondrial sequences are summarized in Supplementary Table S13.

### Assessment of genome assembly

The completeness of the final assembly was evaluated using BUSCO (RRID:SCR_015008) and LTR Assembly Index (LAI) [66, 70]. KAT was used to compare the genome assembly and the Illumina reads (Supplementary  Fig. S1). Bwa was used to map the Illumina reads to the genome, and Minimap2 was used to map the third-generation ONT and PacBio transcriptome (iso-seq) CCS reads to the genome [71, 72]. The nonprimary alignment was removed, so that each read only mapped once and the mapping ratio and coverage percentage were also calculated (Supplementary Table S14). The coverage depth of single-copy and multicopy core genes should be consistent with a Poisson distribution if without redundancy after checking (Supplementary Fig. S2). The second-generation reads were mapped to the genome with Bwa, and mutation sites were detected using SAMtools/BCFtools (RRID:SCR_005227) [73]. The single-base heterozygous sites were used to calculate the heterozygosity rate, and homozygous sites were used to calculate the error rate. Juicer was used to map the Hi-C data to the final genome assembly. The chromosome clustering heatmap of *M. sinica* was adequate, and there were no obvious chromosome assembly errors (Fig. 2A, B) [64].

### Genome annotation

The repeat libraries were generated by *de novo* identification of the repeat region family using the RepeatModeler software. LTR_retriever (RRID:SCR_017623) was also used to identify the intact long terminal repeat (LTR) retrotransposons, and then a second library was clustered and generated [72]. After combining these 2 libraries directly, we used RepeatMasker (RRID:SCR_012954) to identify repeated regions on the genome. Transcripts were generated following the process of isoseq3 [74] and annotated to the genome using the PASA pipeline (RRID:SCR_014656) [75]. The results were used to train an AUGUSTUS model for 5 rounds of optimization [76]. In total, 154,904 nonredundant protein sequences from *L. chinense* [38], *Cinnamomum kanehirae* [77, 78], *Piper nigrum* [79], *Amborella trichopoda* [80], and *Arabidopsis thaliana* [81] were used as evidence of homologous proteins for gene annotation.

Gene structure annotation was conducted using the Maker2 pipeline [82]. Briefly, AUGUSTUS (RRID:SCR_008417) was used to perform *ab initio* prediction of the genome with the repetitive regions masked out [76]. Transcripts were aligned with the genome using BLASTN (RRID:SCR_001598), and BLASTX (RRID:SCR_001653) was also used for aligning the protein evidence with the genome. Exonerate was used to optimize the alignments [83]. Based on the above 3 categories of evidence, hints files were generated, to allow AUGUSTUS to ultimately synthetically predict the gene models. Annotation edit distance (AED) scores of each gene model were calculated according to the transcript and homologous protein evidence within the pipeline. Finally, false annotations in the coding frame and overly short (≤50 AA) gene annotations were removed. tRNAScan-SE, Barrnap [84], and Rfamscan were used to annotate transfer RNA (tRNA), ribosomal RNA (rRNA), and other noncoding RNA, respectively [85]. BUSCO was used to evaluate the integrated annotated proteins [70].

The functions of protein-coding genes were annotated based on 3 strategies. First, genes were mapped with the eggNOG database using eggNOG-mapper to annotate gene function, including Gene Ontology (GO) and KEGG annotation [86]. Second, for assignment based on sequence conservation, a diamond search of the protein sequences from several protein databases was performed, including the databases Swiss-Prot, TrEMBL, NR, and the *Arabidopsis* database [87]. Lastly, for assignment based on domain conservation, InterProScan was used to examine conserved amino acid sequences, motifs, and domains of proteins by matching against subdatabases of several InterPro databases, including CDD, PANTHER, PRINTS, Pfam, and SMART [88].

### Gene family identification and phylogenetic analysis

OrthoFinder2 was used to infer orthogroups, with the parameters set to “-M msa” [89]. A protein alignment of 1,070 orthogroups with a minimum of 87.5% of species having single-copy genes in any orthogroup obtained from OrthoFinder2 was used to construct a phylogenetic tree using IQTREE, using a maximum likelihood method (the best model was JTT+F+R5,1,000 bootstrap replicates) [90]. In addition, ASTRAL was also used to infer the species tree based on 3,841 gene trees with genes in at least 70% taxa being single copy. MCMCTree, from the PAML package, was used to estimate species divergence time and the mutation rate in *M. sinica*, based on the codon alignment of 211 1:1 nonmissing single-copy orthologous genes [91]. Four fossil calibration time points were chosen: stem Nymphaeaceae (113 Mya: Millions of Years Ago), stem Poaceae (55.8 Mya), stem Lauraceae (104 Mya), and stem Santalales (65.5 Mya) [92, 93]. The root time of the phylogentic tree was set according to previous studies [92, 93]. Based on the time tree and 12,306 homologous gene families, CAFE was used to assess the expansion, contraction, and rapid evolution of the gene families [94].

Based on the orthologous and paralogous gene relationships inferred with OrthoFinder2, collinearity between and within species was analyzed using MCScanX_h [95]. According to the collinear homologous gene pairs, the protein sequences were first aligned with MUSCLE [96] and then transformed into codon alignment with PAL2NAL [97]. Ka and Ks were then calculated between homologous gene pairs using KaKs_Caculator v2.0 (YN model) [98, 99]. Polyploidization events and time were inferred based on collinearity in combination with the Ks value [99].

### Genome mapping and single-nucleotide polymorphism calling

A total of 43 samples, including 21 samples of *M. sinica* and 22 samples of a further 8 Magnoliaceae species, were sampled for whole-genome resequencing (Supplementary Tables S1, S2). A total of 5,687 million reads were produced across all samples. The raw data were filtered using fastp [100] to trim away the adaptors and low-quality regions. The cleaned reads were mapped to the reference genome using BWA-MAM [71] with the default parameters. The markdup model in SAMtools [73] was used to mark and to remove duplicate reads. To improve the accuracy of the subsequent analyses, we only retained bases with a quality score >20 and mapping quality >30 (as the filter parameters in ANGSD and Freebayes). We removed the sites with a mapping depth across all samples of <100 or >600 as well as the sites not mapped to chromosomes, using SAMtools. In total, 1,585,988,829 sites (dataset 1) from the BAM files were retained after quality control.

Freebayes (RRID:SCR_010761) [101] was used to process single-nucleotide polymorphisms (SNPs) calling for *M. sinica* and a total of 176,087,519 variable sites were obtained. The resulting SNP dataset was then filtered with vcftools (RRID:SCR_001235) [102] using the following criteria: (i) sites with a genotype quality <20 or genotypes with depth <5 were treated as missing, (ii) nonbiallelic and non-SNP sites, (iii) SNPs with missing rate >20% (dataset 2: 11,438,677 SNPs), and (iv) SNPs with minor allele frequency (MAF) <0.05 (dataset 3: 8,149,323 SNPs).

### Population genetics

PopLDdecay was used for linkage disequilibrium analysis across the *M. sinica* genome. The ThetaStat module in ANGSD (RRID:SCR_021865) v0.93 [103] was used to assess genome-wide diversity by calculating different estimators of *θ*, including *θ*_W_ (Watterson’s *θ*) [104] and *θπ* (nucleotide diversity), Tajima’s *D* [105], and Fu and Li’s *D* [106]. These statistics were calculated in a window size of 20 kb and a step size of 10 kb according to the result of LD decay, using dataset 1 generated previously. Individual heterozygosity was also calculated in ANGSD v0.93 for *M. sinica* in our research.

For population structure analysis, we first used PLINK (RRID:SCR_001757) [107] to remove linkage sites from dataset 3 with the parameter “–indep-pairwise 50 10 0.2,” and we obtained a total of 454,661 independent SNPs (dataset 4). Dataset 4 was further used to explore the population structure of *M. sinica* using the program Admixture v1.3.0 [108], and the most likely number of genetic clusters (ancestor numbers, *K*) was selected based on 10-fold cross-validation error (CV) value. Supplementary Fig. S3 contains a schematic diagram showing how these datasets were generated.

### Ancestral sequence reconstruction

We mapped data from several samples of other species of *Magnolia* and a sample of *Liriodendron* (Supplementary Table S15) to the *M. sinica* genome using BWA-MEM with the default parameters. At the same time, we used freebayes to call the genotype with the same filter parameters as the SNP calling described above, except that “–report-monomorphic” was used to keep monomorphic genotypes in the output. Phylogenetic trees were constructed using IQtree with the substitution model MFP+ASC and using *L. chinense* as the outgroup. We then used an empirical Bayesian method in IQtree [90] to reconstruct the ancestral state of each site of each chromosome; this method can produce accurate ancestral sequence reconstruction [109] and has been previously used to reconstruct ancestral state in other works [23, 110–112]. Finally, we reclassified the ancestral state according to the posterior probability of each site. Posterior probabilities ≥0.95 were classed as “high confidence”; lower probabilities were considered ambiguous and marked as “N.” The sequence from the crown group of *Magnolia* species was defined as the ancestral state.

### Inference of demographic history

A stairway plot was used to infer the demographic history of *M. sinica* [113]. The mutation rate was estimated as 1.2e-7 per locus per generation, which was constructed using MCMCTree based on the 4-fold degenerated sites (4D sites) of orthologous genes. The generation time was set as 30 years, based on the cultivation records of this species in KBG. Dataset 1 was further filtered by removing the sites within 5 kb of gene regions to ensure site neutrality, and 897,314,345 genomic sites were retained (dataset 5). The unfolded site frequency spectrum (SFS) for *M. sinica* was estimated using the functions doSaf and realSFS in ANGSD v 0.921 [103] with dataset 5 and the recommended filtering parameters “-minMapQ 30 -minQ 20.”

We also used the pairwise sequentially Markovian coalescent (PSMC) model to reconstruct the demographic history of *M. sinica* [114]. Using the BAM files (dataset 1) generated by BWA-MAM and the markdup model in SAMtools [73], we made a consensus fastq file for each sample using SAMtools and BCFtools with the parameter set to -C50 to downgrade the mapping quality for reads containing excessive mismatches. The script vcfutils.pl was used to keep the minimum read depth to 5× and the maximum read depth to 50× for all individuals. The consensus fastq file was converted into an input file for PSMC using fq2psmcfa with the parameter -q 20 set, to remove consensus calls with qualities ≤20. The PSMC analysis was run using default values for the upper limit to assign a date to the most recent common ancestor (-t 15) and theta/rho (-r 5). The atomic time interval pattern (-p) was set to “4+30*2+4+6+10.” We plotted the results using the same mutation rate and generation time as described above.

The contemporary effective population size of *M. sinica* was assessed using the linkage disequilibrium method in NeEstimator V2 [103] with the reduced dataset 4 (filtered by vcftools with –max missing 0.95 and –thin 60000) to ensure accuracy [115].

### Estimation of deleterious mutations and inbreeding

Accumulation of deleterious mutations is likely to impact species fitness. The Sorting Intolerant from Tolerant (SIFT) algorithm [116] was used to predict deleterious mutations, with the ancestral sequences reconstructed above as a reference. The TrEMBL plant database [117] was used to search for orthologous genes. After polarization of dataset 2, protein-coding variants of 8,896,099 retained SNPs were categorized as nonsynonymous or synonymous sites. Nonsynonymous sites were further divided into deleterious (SIFT score <0.05) and tolerated (SIFT score ≥0.05) based on their SIFT score [118]. We also calculated the derived allele frequency (DAF) of deleterious mutations.

In addition, frequency of runs of homozygosity (FROH) has been used as a robust estimate of genomic inbreeding [119] and was estimated following previous research [120, 121]. Briefly, runs of homozygosity (ROHs) were first identified based on dataset 2 using vcftools v0.1.17 with parameter “–LROH ” [102], and then FROH was calculated with the total length of ROH divided by the genome size of *M. sinica*.

## Results

### Genome sequencing and assembly

The libraries sequenced on the ONT PromethION platforms using 7 cells resulted in the generation of a total of 9.11 million reads with ∼202.85 Gb sequencing data (∼100×), with an average read length of 22 kb (the longest read was 194 kb, and N50 was 25 kb) ( Supplementary Table S3). A total of 1,432 million reads were generated with ca. 214.95 Gb (∼110×) data using the Illumina HiSeq platform (Supplementary Table S4). A total of 1,480 million reads with ca. 222.13 Gb data were produced with Hi-C sequencing (Supplementary Table S5). Through the optimal assembly method, the final size of the assembled *M. sinica* genome was 1.84 Gb, which was similar to the 1.9 Gb genome size estimated using *k*-mers (Supplementary Fig. S4, Supplementary Tables S10, S11). A total of 108 contigs (1.82 Gb, accounting for 99.08% of the whole genome) with an average size of 15 Mb were anchored onto the 19 chromosomes. The contig N50 of the *M. sinica* genome was ca. 45 Mb and the scaffold N50 was ca. 92 Mb, both of which were much higher than those of other previously reported magnolia genomes (Table 1) [37–40]. In addition, the mitochondrial and chloroplast genomes were assembled into circular DNA molecules of 856,922 bp and 160,070 bp, respectively. The LAI value was estimated to be 10.3 based on LTR, indicating that the gene integrity was relatively good (Supplementary Tables S11, S12). We also calculated that the heterozygosity rate in *M. sinica* was about 1.21% and that the error rate was about 0.0072%.

**Table 1:** Statistics of *Magnolia sinica* genome assembly and annotation

Parameter	*Magnolia sinica*
Total assembly size (bp)	1,839,595,854
GC content (%)	40.18
Total number of contigs	203
Maximum contig length (bp)	96,921,630
Minimum contig length (bp)	5,003
Contig N50 (bp)	44,871,976
Contig N90 (bp)	10,133,504
Total number of scaffolds	130
Maximum scaffold length (bp)	141,926,363
Minimum scaffold length (bp)	5,003
Scaffold N50 (bp)	92,164,922
Scaffold N90 (bp)	73,752,208
Gap number	73
Complete BUSCOs (%)	97.9
Complete single-copy BUSCOs (%)	94.3
Complete and duplicated BUSCOs (%)	3.6
Fragmented BUSCOs (%)	0.5
Missing BUSCOs (%)	1.6
Gene number	44,713
Protein-coding genes	43,473
LAI value	10.3

In total, 1,580 (97.9%) complete BUSCO genes, including 1,522 (94.3%) complete and single-copy genes and 58 (3.6%) complete and duplicated genes, were identified among the 1,614 total BUSCO groups. However, 8 (0.5%) genes were found to be fragmented and 26 (1.6%) genes were missing based on the BUSCO analysis (Supplementary Table S11).

### Genome annotation

A total of 2,329,558 repetitive sequences were identified in the *M. sinica* genome, with a total length of ∼1.05 Gb, and accounting for 56.99% of the genome. Of these, the highest proportion was LTR, accounting for 48.9% of the whole genome (Supplementary Table S16). The most abundant repeat element families were Copia (388,301, 14.88%) and Gypsy (759,932, 27.40%) (Supplementary Table S16). A total of 18 million subreads with ∼24.58 Gb data were generated from transcriptome sequencing, from which 43,473 protein-coding genes were annotated (Supplementary Tables S6, S17). The mean lengths of gene region, transcript, and coding DNA sequences were 11,297, 1,552, and 1,091, respectively (Supplementary Table S17). Moreover, 71 rRNA, 658 tRNA, and 511 noncoding RNA sequences were identified (Supplementary Table S18). A total of 38,041 genes were annotated using GO (14,360, 33.03%), KEGG (14,937, 34.36%), eggNOG (29,585, 68.05%), and COG (31,414, 72.26%). Based on sequence conservation, several protein databases, including Swiss-Prot (21,220, 48.81%), TrEMBL (31,720, 72.96%), NR (31,242, 71.87%), and *Arabidopsis thaliana* (25,007, 57.52%), were annotated with diamond. For assignment based on domain conservation, certain other databases, including Pfam (25,850, 59.46%), Coils (2,533, 5.83%), CDD (28,110, 64.70%), SMART (8,247, 18.97%), and others, were annotated with InterProScan (Supplementary Table S19).

### Analysis of phylogeny, collinearity, and whole-genome duplication

In order to investigate the early evolution of the core angiosperms, we identified 579,290 homologous genes belonging to 20,538 gene families from the 18 related genomes using OrthoFinder2 (Supplementary Fig. S5). A total of 1,266 expanded and 1,276 contracted gene families in *M. sinica* were identified and annotated (Fig 2C). A maximum likelihood tree was constructed using 1,070 orthogroups of 18 species. As shown in the ML phylogenetic tree (Fig. 2C), magnolias formed a sister relationship with both the eudicots and the Ceratophyllales, while the monocots were sister to the other core angiosperms. The Magnoliales and the Laurales were predicted to have diverged from the Piperales at ca. 149.3 Mya (137.7–160), a result that was slightly different from that of a whole-genome study of black pepper, in which the differentiation time was estimated at 175 to 187 Mya [79]. The Magnoliales were predicted to have diverged from the Laurales at ca. 122.2 Mya. In the Magnoliales, the estimated differentiation time of the genera *Magnolia* and *Liriodendron* was predicted to be 23.4 Mya, and within *Magnolia*, the closely related species *M. sinica* and *M. biondii* were estimated to have diverged ca. 10.9 Mya.

A total of 7,807 colinear gene pairs on 779 colinear blocks were inferred within the *M. sinica* genome. The collinearity depth ratio between *M. sinica* and *L. chinense* was 1:1 (Supplementary Fig. S6), indicating that the 2 species have no species-specific whole-genome duplication (WGD) events. Collinearity between these 2 species and earlier differentiated dicotyledons such as grapes was always 2:3 (Supplementary Figs. S7, S8), indicating that *M. sinica* and *L. chinense* experienced a WGD event after differentiation from the eudicots, which is consistent with the conclusions of the study investigating *L. chinense* [38]. Similarly, the collinearity with the early angiosperms *Amborella trichopoda* and *Nymphaea tetragona* was 2:1 and 2:2 (Supplementary Figs. S9, S10), respectively, which indicates that *M. sinica* and *L. chinense* only experienced a single shared WGD event after their differentiation from these plants. From the paralogous collinearity block in *M. sinica*, it can be seen that this WGD event occurred at a Ks value of about 0.75. Based on the chromosome tree analysis, the Magnoliaceae and the Lauraceae shared a WGD event, but this is not shared with pepper. After differentiation from other species, the Magnoliaceae (*M. sinica* and *L. chinense*) experienced a single WGD event, the Lauraceae (*Cinnamomum kanehirae*) experienced 2 WGD events, and pepper experienced 3 WGD events.

### Genome-wide diversity and population structure

After filtering out low-quality reads and adapter sequences, 5,386 million reads remained for processing (Supplementary Table S20). The sequencing depth of *M. sinica* samples ranged from 8.8× to 12.6×, with a mean value of 10.5×, and were between 10.8× and 14.3× for the other 8 Magnoliaceae species (Supplementary Table S20). The mapping rates of *M. sinica* ranged from 90.80% to 99.70%, with a mean value of 97.63%, and were 95.30% to 99.53% for the other 8 Magnoliaceae species (Supplementary Table S20).

The mean heterozygosity rate of *M. sinica* was 1.29% ± 0.07% (Supplementary Table S21), ranging from 1.12% to 1.38%, and the trees with the lowest and the highest heterozygosity rates were both found in the XZQ population. The MAD population had the lowest heterozygosity (1.19%), while the DLS population had the highest heterozygosity (1.32%).

Nucleotide diversity in *M. sinica* was estimated using 2 parameters. Watterson’s *θ* (*θ*w) and genome-wide diversity (*θ*π) of *M. sinica* were calculated as 0.01416 and 0.01494, respectively (Supplementary Table S22). When compared with other species, *M. sinica* was found to have higher genetic diversity (Supplementary Table S23) and was approximately 12-fold higher than that of *L. chinense* (0.00123) [38].

**Figure 3:**
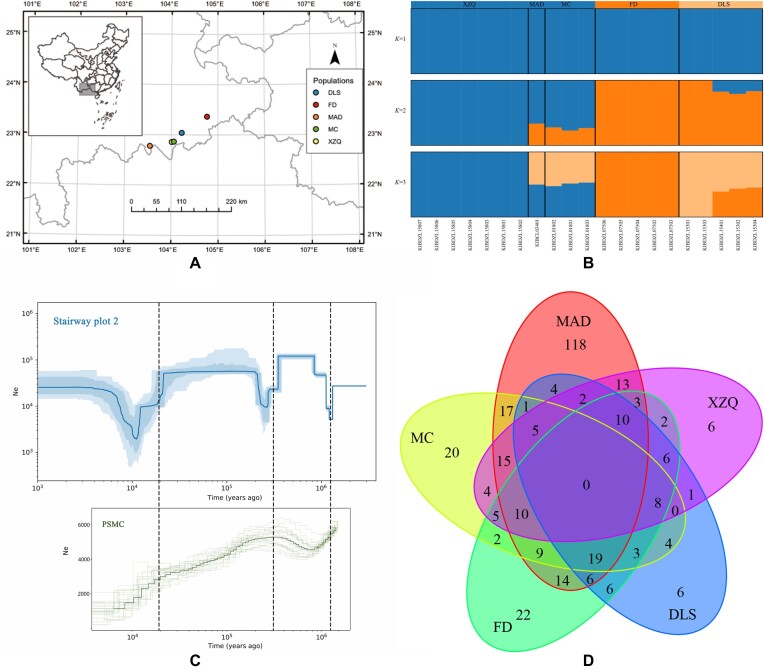
Distribution map, population structure, demographic history, and Venn diagram of *Magnolia sinica*. (A) Distribution map showing the locations of the 5 subpopulations in Yunnan. (B) Plots of the population structure of 21 *M. sinica* individuals from 5 subpopulations ( *K*), from *K* = 1 to *K* = 3. (C) The demographic history of *M. sinica* inferred in stairway plot 2 (with a generation time of 30 years and a mutation rate of 1.2e-7; the 95% confidence interval for the estimated effective population size is shown in a light blue color) and PSMC plot (with 21 samples of *M. sinica*, with the green line being the average effective population size). (D) Venn diagram showing distribution of shared and unique deleterious mutations among the 5 subpopulations of *M. sinica*. DLS, Dalishu population in Maguan County; FD, Fadou population in Xichou County; MAD, Maandi population in Jinping County; MC, Miechang population in Maguan County; XZQ, Xinzhaiqing population in Maguan County.

The population structure results showed that the CV error was smallest when there was an optimal number of clusters *K* = 1 (Supplementary Fig. S11), suggesting low genetic differentiation among populations of *M. sinica* (Fig. 3A). Low genetic differentiation among populations was further suggested by the low *F_st_* statistics between population pairs of *M. sinica*, which had a mean value of 0.133. We have given the structure results for *K* = 2 and *K* = 3 in Fig. 3B. At *K* = 2, all the populations of *M. sinica* could be separated into 2 components, including a blue component and an orange component, the DLS, MAD and MC populations appeared to have mixed ancestry between the XZQ and FD populations. When *K* = 3, the DLS population appeared to be genetically mixed with the MAD, MC and FD populations. Both the XZQ and FD populations were genetically “pure” from the other *M. sinica* populations. The MAD and MC populations were genetically similar irrespective of *K*.

### Demographic history

The demographic history of *M. sinica* inferred by stairway plot 2 indicates 3 significant population declines, 2 of which were also detected by PSMC (Fig. 3C). In the scenario inferred from stairway plot 2, the earliest population decline occurred at 1.3 Mya and continued until 1.1 Mya. For the scenarios inferred by the PSMC, the earliest population decline occurred at 1.5 Mya and continued until 0.8 Mya. After this, the population of *M. sinica* is predicted to have experienced a period of recovery in both scenarios. The second population decline occurred at about 0.3 Ma in both scenarios. After that, the population of *M. sinica* exhibited recovery in the scenario inferred by stairway plot 2 but experienced a continuing decline in PSMC. The latest population bottleneck in both scenarios occurred at about 20 Ka (One thousand years) ago and continued until 10 Ka, when the effective population size of *M. sinica* dropped to 1,936 in the stairway plot and 1,784 in PSMC. However, after 10 Ka, the effective size of the *M. sinica* population recovered in the stairway plot but showed continuous decline in PSMC. The contemporary effective population size of *M. sinica* estimated by NeEstimator was 10.9 (3.3–43.7 jackknife CI).

### Genetic load and genomic inbreeding coefficient

In total, 1,196,374,340 high-confidence loci were obtained and used as ancestral sequences to predict deleterious mutations; 16,131,74,385, and 36,827 sites were predicted to be deleterious, synonymous, and tolerated, respectively, in the 21 resequenced *M. sinica* individuals (Supplementary Table S24). The mean value of derived homozygous deleterious alleles (HoDA) was 249, ranging from 190 to 298, with the lowest found in the MC population, which had a mean number of 207 (190–216), and the highest was found in the XZQ popilation, which had a mean number of 258 (220–298) (Supplementary Table S25). The MAD population also harbors a very high number of HoDA (246), and this population had the highest proportion of private HoDA (118, 48%) when compared with other populations (Fig. 3D, Supplementary Table S25). None of the HoDA was shared among all 5 of these populations. An average of 2,607 heterozygous deleterious alleles (HeDA) were detected in *M. sinica*, ranging from 2,136 to 2,967. The highest number of HeDA was found in the XZQ population, which had a mean value of 2,593 (2,136–2,967) (Supplementary Table S25), while the lowest number of HeDA was found in the MAD population (2,430). The MAD population shared the highest HeDA with the MC population and the lowest HeDA with the XZQ population. None of the HeDA was shared among all 5 of the populations. The derived allele frequency (DAF) of approximately 32.35% of the deleterious mutations was <0.05, and all these rare deleterious mutations were heterozygous. Only ∼7.1% (1,147/16,131) of the deleterious mutations were homozygous (DAF >0.05) (Supplementary Fig. S12).

At the population level, the mean value of FROH in *M. sinica* was 0.11 ± 0.04, ranging from 0.08 to 0.16, with the lowest value found in the DLS population and the highest value found in the MAD population. At the individual level, 1 individual (KIBDZL15801) from the XZQ population showed the lowest level of inbreeding and had the lowest FROH value (0.06). The individual (KIBDZL15803) with the largest FROH value (0.21) was also found in XZQ population (Supplementary Table S25).

## Discussion

To date, only 4 species in the Magnoliaceae (*L. chinense, M. officinalis, M. obovata*, and *M. biondii*) have been the objects of in-depth genomic research, and this has been mainly from the perspective of confirming the phylogeny of the angiosperms, investigation of species differentiation, and the biosynthesis of terpenoids. To date, no species in the family Magnoliaceae have been studied at a genome-wide level from the perspective of conservation [38–41]. From the aspect of conservation genomics, we report high-quality whole-genomic data from *M. sinica* (1.84 Gb with contigs N50 of ca. 45 Mb). This is superior to the data available from *L. chinense* (1.74 Gb with contigs N50 of ∼1.43 Mb) [38], *M. officinalis* (1.68 Gb, with contigs N50 of 0.22 Mb) [40], *M. obovata* (1.64 Gb, with contigs N50 of 1.71 Mb) [41], and *M. biondii* (2.22 Gb with contigs N50 of 0.27 Mb) [39].

The early evolution of the core angiosperms has been studied with whole-genome analysis of certain species of Magnoliids and Chloranthales [39, 77, 120, 122–125]. However, the phylogenetic relationships between the Magnoliids on the early branch of the angiosperm lineage and the eudicots and monocots have been controversial and not fully resolved [124, 125]. Our genome-level phylogenetic tree suggests that the magnolias form a sister group to the eudicots and the Ceratophyllales, while the monocots are sister to the other core angiosperms. This is consistent with the results of a study into Chloranthales [120, 124], but inconsistent with the relevant results of *M. biondii, M. hypoleuca*, and *M. officinalis* [39–41]. The evolutionary history of the angiosperms was accompanied by frequent WGD events. However, evidence of WGD events was inferred from dot plots and Ks, which is insufficient to demonstrate whether any 2 species very close to differentiation share a WGD event. In our study, we concatenated homologous genes to construct a chromosome-level synteny tree to make our inferences more reliable. Our inference results suggest that WGD events also occurred after the differentiation of the magnoliids from other groups, which is in agreement with other studies [125].

Genetic diversity is essential to allow species evolution in response to environmental changes and has been predicted to be positively correlated with species fitness and evolutionary potential [126]. We found that *M. sinica* had relatively high genetic diversity, which is consistent with previous research based on SSR markers [49]. This high diversity could be explained by the fact that, as a tree species, *M. sinica* has a long life span (ca. 30 years). De Kort et al. [127] compared the genetic diversity of 164 annuals, 1,405 perennials, 308 shrubs, and 2,337 trees and found that although species-level diversity is lower for long-lived or low-fecundity species than for short-lived or high-fecundity species, population-level genetic diversity is usually higher for long-living plants, as they may respond more slowly to reduced gene flow. Another reason for this high diversity could be that *M. sinica* is found in southern subtropical monsoon broadleaved evergreen forests [5, 48]. Species around the equator are expected to have higher population-level genetic diversity than other species. This is because in theoretical prediction analyses, the abundant precipitation around the equator shows a significant relative contribution to population genetic diversity, although the exact mechanisms and extent of this are still unknown [127]. Moreover, the pollinator-dependent pollination system may contribute to the high genetic diversity in *M. sinica* [49].

*M. sinica* has low genetic differentiation between subpopulations, which could be attributed to higher gene flow among subpopulations, despite the fragmented distribution of the species [49]. The species has an outcrossing mating system, which is pollinator dependent, and 2 species of beetles appear to be effective pollinators [5, 48]. Previous research has demonstrated that some beetles can fly up to 12 km [128]. Long-distance pollen-mediated gene flow among populations may decrease population genetic differentiation [129]. The smaller FROH and lower inbreeding load in *M. sinica* compared with *Acer yangbiense* may also indicate the existence of certain gene flow among its isolated populations [121] or from other populations that we have not found. As most of the reported populations of *M. sinica* are found on the borders of China with other countries, it is not unreasonable to suggest that other unreported individuals or populations exist outside China.

Southeast Yunnan is an important biodiversity hotspot [130] and is shielded by the Ailao Mountains from the climate fluctuations caused by glaciation and the uplift of the Himalayas and the Hengduan Mountains [131]. From the geological point of view, there is no evidence that southeast Yunnan was affected by the Quaternary ice age, and simulations of climate data suggest that this area was not seriously affected by the global temperature drop [132]. In our results, stairway plot 2 detected major population declines, which is similar to the inferred demographic history of the sympatric *Magnolia fistulosa* [133]. Each *M. sinica* population decline inferred in the stairway plot could be verified in PSMC (Fig. 3C). However, the demographic history of *M. sinica* inferred by stairway plot 2 shows population rebound after each decline, which was not obvious in the PSMC analysis. Moreover, the stairway plot can estimate very recent events, while PSMC estimates only up to 10,000 years ago (Fig. 3C). The earliest inferred population decline occurred 1.0 to 1.2 Ma, which is consistent with the mid-Pleistocene transition [134]. Population declines at a similar time are also reflected in other sympatric species such as *Acer yangbiense* [121] and *Buddleja alternifolia* [120]. The second population decline occurred at 0.3 Ma, during which global temperature experienced a general decline [135]. The latest population decline occurred at ca. 20 Ka and may have been caused by the Last Glacial Maximum (19.0–26.5 Ka) [136]. Multiple population declines may have resulted in a narrow distribution of *M. sinica*, and the stable population sizes from about 1 Ka inferred in the stairway plot may be a result of the very recent large-scale anthropogenic land development and land-use changes in the habitat of *M. sinica* and is likely to have been responsible for the extremely rare status of this species [27]; this is also consistent with the characteristics of high genetic diversity and low genetic differentiation of this species. The value of genetic differentiation among populations separated in recently tends to be lower than those isolated from histprical, especially for species with long generation times [137]. *M. sinica* has a pollinator-dependent outcrossing mating system, which may contribute to its high genetic diversity, while high gene flow among populations may maintain links between populations of this species and may contribute to its low genetic differentiation. The recent reduction in population size due to anthropogenic activities has led to an isolation state of the populations, leading to the high genetic diversity and low genetic differentiation now observed in the fragmented populations of this endangered tree species. Similar patterns have been reported in *Michelia coriacea*, another species in the Magnoliaceae [138].

The MAD population only sampled a single individual with a higher level of inbreeding (FROH = 0.16), lower heterozygosity rate (1.19%), and higher homozygous deleterious allele number (246) than other populations. Gene flow has been proposed as a potential strategy to sustain small and isolated populations by masking of deleterious alleles [139]. We found that the DLS population had a higher heterozygosity rate (1.32%) and shared few homozygous deleterious mutations with the tree from the MAD population. The DLS population could therefore serve as source material for breeding, which could be used to mask homozygous deleterious mutations in future MAD population individuals. Methods such as population reinforcement, hand pollination to assist pollen flow (by collecting pollen from the DLS population and pollinating the MAD population), or the transplantation of seedlings from the DLS population into MAD could also be considered. Similarly, an individual (KIBDZL15801) in the XZQ population also had a higher heterozygosity rate (1.37%) and a smaller number of HoDA (220) than the MAD population. Pollen from KIBDZL15801 could therefore be used to assist gene flow to KIBDZL15803 and KIBDZL15807, 2 other individuals from the XZQ population with lower heterozygosity rates (1.12% and 1.16%, respectively) and higher numbers of HoDA (298 and 286, respectively).

The identification of a management unit (MU) is essential for the management of natural populations [140]. The FD population was genetically pure, and had no admixture with other populations even when *K* = 2 and *K* = 3. This could be attributed to its distance from the other populations (about 66–145 km), which may decrease opportunities for pollen flow. Similarly, population XZQ was also found to be genetically pure at *K* = 2 and *K* = 3. We therefore suggest that the FD and XZQ populations should be treated as 2 separate evolutionarily significant units (ESUs). The MAD and MC populations were genetically similar at all values of *K*, and we suggest that they be treated as another ESU. Importantly, however, the MAD and MC populations are found outside of any existing nature reserves, and it is therefore necessary to include these populations in a nature reserve or to establish specific conservation regions to protect them.

The main threats currently faced by *M. sinica* are as follows: (i) substantial reduction and loss of the original habitat, leading to severe habitat fragmentation and population isolation; (ii) the large-scale planting of *Amomum tsaoko* under forest cover, which means that *M. sinica* is unable to regenerate naturally in the wild, and there are no seedlings; and (iii) excessive artificial seed collection. Fortunately, since 2005, because this plant is a critically endangered flagship species, comprehensive scientific research, including reproductive and seed biology, conservation genetics, and protection measures including field investigations, *in situ* conservation, *ex situ* conservation, and reintroduction, has been gradually implemented [14, 48, 50, 51, 53]. At present, in addition to the existing protection measures, strengthening of the management of nature reserves and reduction of the disturbance by human activities in the original habitats of wild populations are urgently needed. In particular, it is necessary to stop the large-scale planting of commercial crops (*A. tsaoko*) under these forests, which is important to restore their natural regeneration in the wild. Unlike most of the severely threatened species, *M. sinica* has high genetic diversity and low genetic differentiation, which is also consistent with research into other endangered species in the Magnoliaceae [133, 141,142]. However, considering that the generation time of *M. sinica* can be as long as 30 years, the isolation of the various populations, the serious habitat fragmentation, and that there are very few wild individuals, we still need to consider potential future inbreeding depression. More artificial outcrossing strategies should be designed in the future to reduce the loss of genetic diversity caused by inbreeding, and these strategies should be considered instead of collecting seeds and simply breeding more individuals [26]. Our genomic study into *M. sinica* provides an example of high genetic diversity and low genetic differentiation in a long-lived tree species and informs the future formation and maintenance of conservation strategies necessary for the survival of such a PSESP.

## Abbreviations

AED: annotation edit distance; BLAST: Basic Local Alignment Search Tool; BUSCO: Benchmarking Universal Single-Copy Orthologues; CBD COP 15: 15th Conference of the Parties, Convention on Biological Diversity; DAF: derived allele frequency; ESTs: expressed sequence tags; FROH: frequency of runs of homozygosity; GO: Gene Ontology; HeDA: heterozygous deleterious alleles; HoDA: homozygous deleterious alleles; JBAT: Juicebox Assembly Tools; KBG: Kunming Botanical Garden; KEGG: Kyoto Encyclopedia of Genes and Genomes; LAI: LTR Assembly Index; LTR: long terminal repeat retrotransposons; MAF: minor allele frequency; ONT: Oxford Nanopore Technologies; PSESP: Plant Species with Extremely Small Populations; PSMC: pairwise sequentially Markovian coalescent; ROH: run of homozygosity; SFS: site frequency spectrum; SIFT: Sorting Intolerant from Tolerant; SMRT: single molecule real time; *θ*_W_: Watterson’s *θ; θπ*: nucleotide diversity; WGD: whole-genome duplication.

## Supplementary Material

giad110_GIGA-D-23-00060_Original_Submission

giad110_GIGA-D-23-00060_Revision_1

giad110_Response_to_Reviewer_Comments_Original_Submission

giad110_Reviewer_1_Report_Original_Submission Jinhui Chen -- 4/20/2023 Reviewed

giad110_Reviewer_1_Report_Revision_1 Jinhui Chen -- 10/24/2023 Reviewed

giad110_Reviewer_2_Report_Original_Submission Damien Hinsinger -- 5/3/2023 Reviewed

giad110_Supplemental_Files

## Data Availability

The genome assembly, annotations, and other supporting data are available via the *GigaScience* database, GigaDB [143]. The raw sequence data have been deposited in the Short Read Archive under NCBI BioProject ID PRJNA774088. The raw data, genome assembly, and gene annotation have also been deposited at National Genomics Data Center, China National Center for Bioinformation under BioProject accession number PRJCA015437.

## Additional Files

**Supplementary Fig. S1**. *K*-mer spectrum analysis.

**Supplementary Fig. S2**. Evaluation of the distribution of coverage depth over the whole genome and the BUSCO core gene region with Illumina and ONT data.

**Supplementary Fig. S3**. A schematic diagram showing how these datasets were generated.

**Supplementary Fig. S4**. *K*-mer frequency distribution diagram.

**Supplementary Fig. S5**. Maximum likelihood (ML) phylogeny of *M. sinica* and related taxa showing bootstrap values.

**Supplementary Fig. S6**. The collinearity between *M. sinica* and *L. chinense*.

**Supplementary Fig. S7**. The collinearity between *M. sinica* and *V. vinifera*.

**Supplementary Fig. S8**. The collinearity between *L. chinense* and *V. vinifera*.

**Supplementary Fig. S9**. The collinearity between *A. trichopoda* and *M. sinica*.

**Supplementary Fig. S10**. The collinearity between *N. colorata* and *M. sinica*.

**Supplementary Fig. S11**. Cross-validation error (CV) based on Admixture output.

**Supplementary Fig. S12**. Deleterious allele frequency distribution of homozygous deleterious SNPs.

**Supplementary Table S1**. Collection information for the 21 resequenced samples of *M. sinica*.

**Supplementary Table S2**. Collection information for the other 8 Magnoliaceae resequencing samples.

**Supplementary Table S3**. WGS-ONT sequencing statistics.

**Supplementary Table S4**. WGS-Illumina sequencing statistics.

**Supplementary Table S5**. HiC sequencing statistics.

**Supplementary Table S6**. Iso-seq sequencing statistics.

**Supplementary Table S7**. Assembly statistics (V0.1).

**Supplementary Table S8**. Assembly statistics (V0.2).

**Supplementary Table S9**. Assembly statistics (V0.3).

**Supplementary Table S10**. Assembly statistics (V1.0).

**Supplementary Table S11**. Assembly statistics (V1.1).

**Supplementary Table S12**. Statistics of all assemblies.

**Supplementary Table S13**. Information pertaining to the chromosomes, unanchored sequences, chloroplasts, and mitochondria.

**Supplementary Table S14**. The mapping ratio and coverage percentage of resequencing data.

**Supplementary Table S15**. Sequences used to construct ancestral states.

**Supplementary Table S16**. Repetitive sequences statistics.

**Supplementary Table S17**. Final gene set statistics.

**Supplementary Table S18**. Statistics of the source of integration annotation.

**Supplementary Table S19**. Gene annotation statistics.

**Supplementary Table S20**. Genome mapping statistics of sequencing data.

**Supplementary Table S21**. Statistics of heterozygsity rate.

**Supplementary Table S22**. Mean population fixation index and corresponding spatial distance.

**Supplementary Table S23**. Genome-wide diversity of woody species.

**Supplementary Table S24**. SIFT prediction of deleterious mutations.

**Supplementary Table S25**. Genetic load of 21 individuals of *M. sinia*.
