# Supplementary material for: The chromosome-scale genome of Magnolia sinica (Magnoliaceae) provides insights into the conservation of plant species with extremely small populations (PSESP)
Source: Gigascience. 2024 Jan 11;13:giad110. doi: 10.1093/gigascience/giad110 (PMC10999834; doi:10.1093/gigascience/giad110)
Supplement: giad110_GIGA-D-23-00060_Original_Submission [file giad110_giga-d-23-00060_original_submission.pdf]

GigaScience

The chromosome-scale genome of Magnolia sinica (Magnoliaceae) provides insights into the conservation of plant species with extremely small populations (PSESP)

--Manuscript Draft--

**Manuscript Number:**	GIGA-D-23-00060
**Full Title:**	The chromosome-scale genome of Magnolia sinica (Magnoliaceae) provides insights into the conservation of plant species with extremely small populations (PSESP)
**Article Type:**	Research
**Funding Information:**	National Science & Technology Basic Resources Investigation Program of China (2017FY100100) — Prof. Weibang Sun<br>National Natural Science Foundation of China (NSFC) – Yunnan Joint Fund (U1302262) — Prof. Weibang Sun<br>Yunnan Fundamental Research Projects (202101AT070173) — Dr. Lei Cai<br>National Natural Science Foundation of China (NSFC) (32101407) — Dr. Lei Cai
**Abstract:**	Magnolia sinica (Magnoliaceae) is one of the most highly threatened trees endemic to Southeast Yunnan, China. In this study, we generated for the first time a high-quality chromosome-scale genome sequence from M. sinica, by combining Illumina and PacBio data with Hi-C mapping methods. The final assembled genome size of M. sinica was 1.84 Gb, with a contig N50 of ca. 45 Mb and scaffold N50 of 92 Mb. Identified repeats constituted approximately 57% of the genome, and 43,473 protein-coding genes were predicted with high support. Phylogenetic analysis showed that the magnolias form a sister clade with the eudicots and the order Ceratophyllales, while the monocots are sister to the other core angiosperms. A total of 21 individuals from the five remnant populations of M. sinica, as well as 22 specimens belonging to eight related Magnoliaceae species, were resequenced. The results showed that M. sinica had higher genetic diversity (θw = 0.01126 and $\theta \pi$ = 0.01158) than other related species in the Magnoliaceae. However, population structure analysis suggested that the genetic differentiation among the five M. sinica populations was very low. Analyses of the demographic history of the species using different models consistently revealed that two bottleneck events occurred. The contemporary effective population size of M. sinica was estimated to be 10.9. Additionally, different patterns of genetic loads (inbreeding and numbers of deleterious mutations) suggested constructive conservation strategies for these five different populations of M. sinica. Overall, this high-quality genome could be a valuable genomic resource for conservation of M. sinica.
**Corresponding Author:**	Yongpeng Ma<br><br>CHINA
**Corresponding Author Secondary Information:**	
**Corresponding Author's Institution:**	
**Corresponding Author's Secondary Institution:**	
**First Author:**	Lei Cai, Ph.D.
**First Author Secondary Information:**	
**Order of Authors:**	Lei Cai, Ph.D.<br>Detuan Liu<br>Fengmao Yang

Powered by Editorial Manager® and ProduXion Manager® from Aries Systems Corporation

	Rengang Zhang
	Quanzheng Yun
	Zhiling Dao, PhD
	Yongpeng Ma
	Weibang Sun, PhD
**Order of Authors Secondary Information:**	
**Additional Information:**	
**Question**	**Response**
Are you submitting this manuscript to a special series or article collection?	No
**Experimental design and statistics**<br><br>Full details of the experimental design and statistical methods used should be given in the Methods section, as detailed in our Minimum Standards Reporting Checklist. Information essential to interpreting the data presented should be made available in the figure legends.<br><br>Have you included all the information requested in your manuscript?	Yes
**Resources**<br><br>A description of all resources used, including antibodies, cell lines, animals and software tools, with enough information to allow them to be uniquely identified, should be included in the Methods section. Authors are strongly encouraged to cite Research Resource Identifiers (RRIDs) for antibodies, model organisms and tools, where possible.<br><br>Have you included the information requested as detailed in our Minimum Standards Reporting Checklist?	Yes
**Availability of data and materials**<br><br>All datasets and code on which the conclusions of the paper rely must be	Yes

either included in your submission or deposited in publicly available repositories (where available and ethically appropriate), referencing such data using a unique identifier in the references and in the “Availability of Data and Materials” section of your manuscript.<br><br>Have you have met the above requirement as detailed in our Minimum Standards Reporting Checklist?	

Powered by Editorial Manager® and ProduXion Manager® from Aries Systems Corporation

Main document
Click here to access/download;Manuscript;001 The whole genome of Magnolia sinica 202300306.pdf

The chromosome-scale genome of *Magnolia sinica* (Magnoliaceae) provides insights into the conservation of plant species with extremely small populations (PSESP)

Lei Cai[1,†], Detuan Liu[1,†], Fengmao Yang[1], Rengang Zhang[2], Quanzheng Yun[2], Zhiling Dao[1], Yongpeng Ma[1,*], Weibang Sun[1,3*]

[1] Yunnan Key Laboratory for Integrative Conservation of Plant Species with Extremely Small Populations/ Key Laboratory for Plant Diversity and Biogeography of East Asia, Kunming Institute of Botany, Chinese Academy of Sciences, Kunming, Yunnan, China

[2] Department of Bioinformatics, Ori (Shandong) Gene Science and Technology Co., Ltd., Weifang, Shandong, China

3 Kunming Botanical Garden, Kunming Institute of Botany, Chinese Academy of Sciences, Kunming, 650201, China

Correspondence address. Yongpeng Ma, E-mail: mayongpeng@mail.kib.ac.cn; http://orcid.org/0000-0002-7725-3677; Weibang Sun, E-mail: wbsun@mail.kib.ac.cn; https://orcid.org/0000-0002-7195-2215

[†]These authors contributed equally to this work.

Abstract

Magnolia sinica (Magnoliaceae) is one of the most highly threatened trees endemic to Southeast Yunnan, China. In this study, we generated for the first time a high-quality chromosome-scale genome sequence from *M. sinica*, by combining Illumina and PacBio data with Hi-C mapping methods. The final assembled genome size of *M. sinica* was 1.84 Gb, with a contig N50 of ca. 45

Mb and scaffold N50 of 92 Mb. Identified repeats constituted approximately 57% of the genome, and 43,473 protein-coding genes were predicted with high support. Phylogenetic analysis showed that the magnolias form a sister clade with the eudicots and the order Ceratophyllales, while the monocots are sister to the other core angiosperms. A total of 21 individuals from the five remnant populations of *M. sinica*, as well as 22 specimens belonging to eight related Magnoliaceae species, were resequenced. The results showed that *M. sinica* had higher genetic diversity (θw = 0.01126 and $\theta\pi$ = 0.01158) than other related species in the Magnoliaceae. However, population structure analysis suggested that the genetic differentiation among the five *M. sinica* populations was very low. Analyses of the demographic history of the species using different models consistently revealed that two bottleneck events occurred. The contemporary effective population size of *M. sinica* was estimated to be 10.9. Additionally, different patterns of genetic loads (inbreeding and numbers of deleterious mutations) suggested constructive conservation strategies for these five different populations of *M. sinica*. Overall, this high-quality genome could be a valuable genomic resource for conservation of *M. sinica*.

Keywords: *Magnolia sinica*, PSESP, genome sequencing, deleterious mutation, population demographic, conservation

1 Introduction

The reduction of species diversity is of global concern, and has been closely linked with climate change and human activity. The conservation of biodiversity is therefore a hot topic [1–6]. The resolution of the recently convened CBD COP 15 (15th Conference of the Parties, Convention on Biological Diversity) supports biodiversity conservation issues of global concern, and one of the

goals (so called “30 × 30”) requires that at least 30% of the land, fresh water and oceans on Earth be protected in some form by 2030. In addition, identification of geographic areas with high concentrations of endemic and rare species diversity is an important step in protecting biodiversity [7]. The Mountains of Southwest China is one of the world's biodiversity hotspots, and is also affected by climate change and human disturbance, meaning that it is also an area at very serious risk of species extinction [8, 9]. Study and protection of the threatened species in this region is therefore of particular importance and urgency [10, 11]. In order to rescue the most highly threatened species and reduce their risks of extinction in this region, Chinese scholars put forward the concept of Plant Species with Extremely Small Populations (PSESP) in 2005, according to China's current national conditions and the practice of biodiversity protection [12–15]. That a species is threatened by human activities and interference is a necessary qualifying condition to determine whether that species meets the definition of PSESP, and human activities are also of significance when implementing rescuing protection for PSESPs [12, 16].

Plant genome sequencing has grown rapidly in the past 20 years, and the genomes sequences of more than 980 higher plant taxa have been published to date (www.plabipd.de/plant_genomes_pa.ep). Sequenced genomes can provide insights and evidence to better understand the genome biology and evolution of plants [17, 18]. Although the genomes of so many plant species have been studied, only few studies have sequenced the genomes of threatened plant species (examples include *Acer yangbiense*, *Acanthochlamys bracteata*, *Beta patula, Cercidiphyllum japonicum*, *Davidia involucrata*, *Dracaena cambodiana*, *Ginkgo biloba*, *Kingdonia uniflora*, *Ostrya rehderiana* and *Rhododendron griersonianum*) in order to focus on the conservation of these species [19–28].

Plant species in the family Magnoliaceae are hugely important in gardens and horticulture across the world [29, 30]. The Magnoliaceae is also one of the most highly threatened angiosperm groups. There are more than 300 species in this family, which are mainly distributed intermittently in the temperate, subtropical and tropical regions of East and Southeast Asia, East North America and central and South America [31–33]. About 120 species of Magnoliaceae are known from China, and Southwest and South China are the centers of diversity for this family [34]. Global conservation assessments suggest that 147 magnoliaceous species are facing threats, accounting for 48% of the total assessed species in this family [33]. Similarly, 76 species of Chinese Magnoliaceae are threatened, representing more than 50% of the total number of threatened Magnoliaceae species globally [35]. At present, in-depth genome research has only been conducted in three species in the Magnoliaceae (*Liriodendron chinense*, *Magnolia biondii* and *M. officinalis*), mainly to investigate the controversial evolutionary position of the magnoliids [36–38].

The evergreen tree *Magnolia sinica* (Law) Noot. (Magnoliaceae) is a typical PSESP endemic to Southeast Yunnan, where many threatened species are in urgent need of rescue and protection [12, 14]. In China, the species is often referred to as *Manglietiastrum sinicum* Y.W. Law and is known as Huagaimu in Chinese [32, 34, 39, 40]. It has also been subsequently categorized as Critically Endangered on the *China Species Red List* [41], *The Red List of Magnoliaceae* [33, 42] and *The Threatened Species List of China's Higher Plants* [35]. *M. sinica* was proposed as a first-rank plant for national key protection in 1999 [43] and also in 2021 (www.forestry.gov.cn/main/3457/20210915/143259505655181.html), and was listed as one of 62 PSESPs in Yunnan in 2010, and also as one of the 120 national PSESPs of China in 2012, requiring the most urgent rescue conservation [14, 15]. Recent survey data revealed only 52 individuals

remaining in the wild, and comprehensive conservation research and protection action of *M. sinica* have been implemented, including reproductive and seed biology, genetic diversity studies based on SSR, sequencing of the chloroplast genome, investigation of the soil microbiome, *in situ* conservation, *ex situ* conservation and reintroduction [44–49].

Here, we report the high-quality chromosome-scale genome sequence of *Magnolia sinica*, and compared it with other relevant published genomic data. By exploring the evolution of the genome, and the genetic characteristics, demographic history and genetic load of *M. sinica*, we have identified genomic factors that may contribute to the threats to this species, and, on the basis of this, we therefore propose conservation strategies for *M. sinica*.

2 Materials and methods

2.1 Collection of plant material

Magnolia sinica is only scattered in several counties in southeast Yunnan (Figures 1 & 3a). Fresh young leaf materials were collected for whole-genome sequencing from a single individual. This individual was conserved *ex situ* at the Kunming Botanical Garden (KBG), but was originally introduced from Xichou County, Southeast Yunnan. For transcriptome sequencing, leaf, stem and root samples were obtained from a three-year-old seedling also at KBG, and fresh fruits were collected from the wild in Jinping County, Yunnan. Fresh leaves used for genome library preparation, and other tissues used for transcriptome sequencing, were immediately frozen in liquid nitrogen and were stored at -80 °C in dry ice until DNA or RNA extraction. The remaining 21 leaf samples for re-sequencing were collected from the original species habitat in Xichou, Maguan and Jinping Counties from 2017 to 2019 (Table S1). Other DNA material from eight further species in the Magnoliaceae was used for comparison of genetic diversity and investigation of the phylogenic

relationships. This DNA material was collected from specimens cultivated at KBG and the Germplasm Bank of Wild Species, Chinese Academy of Sciences (Table S2). After the leaves were collected, they were quickly packed in silica gel desiccant and stored in silica gel until re-sequencing.

2.2 Genome sequencing

Genomic DNA sequencing was performed using different sequencing platforms simultaneously to insure accurate assembly. (1) For ONT (Oxford Nanopore Technologies) PromethION sequencing, total DNA was extracted using the cetyltrimethylammonium bromide (CTAB) method [50] using a QIAGEN® Genomic DNA Extraction kit (cat. no. 13323, Qiagen, Hilden, Germany). A NanoDrop™ One UV-Vis spectrophotometer (Thermo Fisher Scientific, USA) was then used to check DNA purity and a Qubit® 3.0 Fluorometer (Invitrogen, USA) was used to accurately quantify the DNA. After purification, the adapters from the LSK109 Ligation kit (cat. no. SQK-LSK109, Oxford) were used for the ligation reaction, and finally the Qubit® 3.0 Fluorometer (Invitrogen, USA) was used to quantify the constructed DNA library. The DNA library was subsequently transferred to Nanopore GridION X5/PromethION (ONT, UK) for sequencing. (2) For Illumina sequencing, short-insert libraries of 300–500 bp were prepared using 2 μg of genomic DNA, and three Illumina PCR-free libraries were constructed according to the standard manufacturer's protocol using the DNAseq Library Index Kit (Hangzhou Kaitai Biotechnology, Co., Ltd., Hangzhou, China). The whole-genomic libraries were sequenced on an Illumina Hiseq X Ten platform. (3) The Hi-C library was prepared by Beijing Ori-Gene Science and Technology Co., Ltd., Beijing, China. High molecular weight genomic DNA (≥700 ng) was cross-linked *in situ*, extracted and then digested with a restriction enzyme. The DNA ends were then marked with biotin-14-dCTP, the crosslinked fragments were blunt-end ligated. Fragments were sheared to a size of 200–600 bp

with sonication. The Hi-C libraries were amplified using 12–14 cycles of PCR, and were sequenced in Illumina HiSeq X Ten platform. (4) Transcriptome sequencing was performed on a PacBio Sequel (Pacific Biosciences, Menlo Park, CA, USA) platform using full-length isoform sequencing (iso-seq) [51]. High-quality RNA was extracted with a Qiagen kit while a series of RNA samples were tested: Nanodrop was used to assess RNA purity, Qubit was used to precisely quantify the RNA, and an Agilent 2100 Bioanalyzer was used to calculate RIN values and 28S/18S. Then a SMARTer ® PCR cDNA Synthesis Kit was used to reverse transcribe the qualifying RNA into cDNA, The reverse transcription products were amplified using KAPA HiFi PCR Kits, and the amplified products were used to construct a SMRTbell library using a SMRTbell template prep kit 1.0. The third-generation sequencer Sequel was used to sequence the full-length cDNA to obtain high-quality original transcriptome sequencing data.

2.3 Genome assembly

We obtained ~203 G (~100×) ONT reads, ~215 G (~110×) Illumina Hiseq reads, ~222 G Hi-C reads, and ~24 G iso-seq reads (Table S3–S6). The *de novo* genome assembly was first performed on ONT reads using different assembly strategies, then using the Overlap-Layout-Consensus method [52] (Table S7–9). Primary assembly v0.1 was selected as the optimal assembly due to the low error rate. Then, the Illumina sequencing reads were modified using Pilon [53] to improve single-base-pair accuracy. The two draft assemblies were then merged using QuickMerge to improve continuity [54] and then polished again (Table S10–12). GetOrganelle software was used to assemble the mitochondrial and chloroplast genomes [55].

Hi-C reads were mapped to the draft assembly with Juicer, and a candidate chromosome-length assembly was generated automatically using the 3d-DNA pipeline to correct mis-joins, order,

orientation, and to anchor contigs [56, 57]. Manual review and refinement of the candidate assembly was performed in Juicebox Assembly Tools (JBAT) for quality control and interactive correction [58]. To reduce the influence of chromosome interactions and to further improve the chromosome scale assembly, each chromosome was separately re-scaffolded with 3d-DNA, and was then manually refined with Juicebox. Finally, the chromosome frame and scattered sequences were generated, with the gap length set as 100 bp.

To fill the assembly gaps, LR_Gapcloser was run for two rounds based on ONT reads, and then NextPolish was run for three rounds to polish the assembly based on Illumina reads [59, 60]. In order to eliminate redundancy and external source pollution: 1) Redundans was used to remove the redundant scattered sequences (identity ≥ 0.98) [61]; 2) Unplaced contigs with a length of less than 5 kb were removed; 3) The assembly was aligned with the NT library using BLASTn combined with coverage depth, to determine whether there was contamination from other species; and 4) Haplotigs or fragments with low average coverage depth were removed. The chromosomes were coded as chr01-chr19 according to their lengths (from long to short) (Fig 2a, b). The numbers, lengths and proportions of the chromosomes, scattered sequences, chloroplasts and mitochondria are summarized in Table S13.

2.4 Assessment of genome assembly

The completeness of the final assembly was evaluated using BUSCO (Benchmarking Universal Single-copy Orthologues) and LAI (the LTR Assembly Index) [59, 62]. Bwa was used to map the Illumina reads to the genome and Minimap2 was used to map the third-generation ONT and PacBio transcriptome(iso-seq) CCS reads to the genome [63, 64]. The non-primary alignment was filtered out, so that each read only mapped once and the mapping ratio and coverage percentage were also

calculated (Table S14). The coverage depth of single-copy and multi-copy core genes should be consistent with a Poisson distribution if without redundancy after checking (Fig S1). The second-generation reads were compared to the genome with Bowtie2, and mutation sites were detected using SAMtools/BCFtools [65]. The single base heterozygous sites were used to calculate the heterozygosity rate, and the single base homozygous sites were used to calculate the error rate. Juicer was used to map the Hi-C data to the final genome assembly. The chromosome clustering effect of *Magnolia sinica* was good, and there was no obvious chromosome assembly error (Figure 2a, 2b) [57].

2.5 Genome annotation

The repeat libraries were generated by *de novo* identification of the repeat region family using the RepeatModeler software. LTR_retriever was also used to identify the intact LTR (long terminal repeat retrotransposons), and then a second library was clustered and generated [64]. After combining these two libraries, we used RepeatMasker to identify repeated regions on the genome. Transcripts were generated following the official process of isoseq3 (https://github.com/ylipacbio/IsoSeq3) and were annotated to the genome using PASA pipeline [66]. The results were used to train an AUGUSTUS model for five rounds of optimization [67]. 154,904 merged non-redundant protein sequences from *Liriodendron chinense* [36], *Cinnamomum kanehirae* [68, 69], *Piper nigrum* [70], *Amborella trichopoda* [71] and *Arabidopsis thaliana* [72] were used as evidence of homologous proteins for gene annotation.

Gene structure annotation was conducted using Maker2 [73], and AUGUSTUS was used to perform ab initio prediction of the genome with the repetitive regions masked out [67]. Expressed sequence tags (ESTs) were aligned with the genome using BLASTN and T BLASTN, and BLASTN

was also used for the comparison of the protein evidence with the genome. Exonerate was used to optimize the previous alignments [74]. Based on the above evidence, hints files were generated, and AUGUSTUS was then used to integrate the prediction gene model. AED (annotation edit distance) scores of each gene model were calculated according to the EST evidence and the UTR annotations. Finally, false annotations in the coding frame and too short (≤50 AA) gene annotations in the coding frame were removed. Software including tRNAScan-SE, Barrnap (https://github.com/tseemann/barrnap), and Rfamscan were used to annotate tRNA, rRNA and various ncRNA, respectively [75]. BUSCO was used to evaluate integrated annotated protein [62].

The functions of protein coding genes were annotated based on three strategies. Firstly, genes were matched with the eggNOG homologous gene database using eggNOG-mapper to annotate gene function, including GO and KEGG annotation [76]. Secondly, for assignment based on sequence conservation, a diamond search of the peptide sequences from several protein databases was performed, including the databases Swiss-Prot, TrEMBL, NR, *Arabidopsis* database and others [77]. Lastly, for assignment based on domain conservation, InterProScan was used to examine conserved amino acid sequences, motifs and domains of proteins by matching against sub databases of several Interpro databases, including CDD, PANTHER, PRINTS, Pfam, SMART and others [78].

2.6 Gene family identification and phylogenetic analysis

OrthoFinder2 was used to infer orthogroups, with the parameters set to "-M msa" [79]. A protein alignment of 1070 single-copy genes obtained from OrthoFinder2 was used to construct a phylogenetic tree using IQTREE, using a maximum likelihood method (the best model was JTT+F+R5, 1000 bootstrap replicates) [80]. In addition, ASTRAL was used to infer the species tree based on 3841 gene trees for comparison. MCMCTree, from the PAML package, was used to

estimate species divergence time and the mutation rate of *Magnolia sinica*, based on the codon alignment of 211 single-copy orthologous genes [81]. Four fossil calibration time points were chosen: stem Nymphaeaceae (113 Mya), stem Poaceae (55.8 Mya), stem Lauraceae (104 Mya), and stem Santalales (65.5 Mya). The root time of the phylogentic tree was set according to previous studies [82, 83]. Based on the time tree and 12306 homologous gene families, CAFE was used to assess the expansion, contraction and rapid evolution of the gene families [84].

Based on the orthologous and paralogous gene relationships inferred with OrthoFinder2, collinearity between and within species was analyzed using MCScanX_h [85]. According to the collinear homologous gene pairs obtained by MCScanX, the protein sequences were first aligned with MUSCLE [86], and then transformed into codon alignment with PAL2NAL [87]. Ka and Ks were then calculated between homologous gene pairs using KaKs_Caculator v2.0 (YN model) [88, 89]. Polyploidization events and time were inferred based on collinearity in combination with the Ks value [89].

2.7 Genome mapping and SNP calling

A total of 43 samples, including 21 samples of *Magnolia sinica* and 22 samples of a further eight *Magnolia* species, were sampled for whole genome resequencing (Table S1, S2). A total of 5,687 million reads were produced across all samples. The raw data were filtered using fastp [90] to trim away the adaptors and low-quality regions. The cleaned reads were mapped to the reference genome using BWA-MAM [63] with the default parameters. The markdUp model in SAMtools [65] was used to mark and to remove duplicate reads. To improve the accuracy of the subsequent analyses, we only retained bases with a quality score > 20 and mapping quality > 30. We removed the duplicated sites, sites with a mapping depth of < 100 or > 600 as well as the sites not mapped to

chromosomes. 1,585,988,829 sites (Datasets1) from the BAM files were retained after quality control.

Freebayes [91] was used to process SNPs calling for *Magnolia sinica* and a total of 176,087,519 variable sites were obtained. The resulting SNP dataset was then filtered using vcftools [92] using the following criteria: 1) sites with a genotype quality < 20 or genotypes with depth < 5 were treated as missing; 2) non-biallelic SNP sites; 3) the SNPs with missing rate > 20% (Datasets2: 11,438,677 SNPs); 4) sites with minor allele frequency (MAF) < 0.05 (Dataset 3: 3,580,172 SNPs).

2.8 Population genetics

PopLDdecay was used for linkage disequilibrium analysis across the *Magnolia sinica* genome. The ThetaStat module in ANGSD v0.93 [93] was used to assess genome wide diversity by calculating different estimators of Theta, including θW (Watterson's theta) [94] and $\theta\pi$ (nucleotide diversity), and Tajima's *D* [95], and Fu and Li's *D* [96]. These statistics were calculated in a window size of 20 kb and a step size of 10 kb according to the result of LD Decay, using Dataset1. Individual heterozygosity was also calculated in ANGSD v0.93 for *M. sinica* in our research.

For population structure analysis, we first used PLINK [97] to remove linkage sites from Dataset 4 with the parameter "--indep-pairwise 50 10 0.2", and we obtained a total of 454,661 independent SNPs (Dataset 5). Dataset 5 was further used to explore the population structure of *M. sinica* using the program Admixture v1.3.0 [98], and the most likely number of genetic clusters (ancestor numbers, K) was selected based on 10-fold cross-validation error (CV) value.

2.9 Ancestral sequence reconstruction

We mapped data from several samples of other species of *Magnolia* and a sample of *Liriodendron* (Table S15) to *Magnolia sinica* using BWA-MEM with the default parameters. At the

same time, we used freebayes to call the genotype with the same filter parameters as the SNP calling described above, except that “--report-monomorphic” was used to keep monomorphic genotypes in the output. Phylogenetic trees were constructed using IQtree with the substitution model MFP+ASC and using *Liriodendron chinense* as the outgroup. We then used an empirical Bayesian method in IQtree [80] to reconstruct the ancestral state of each chromosome. Finally, we reclassified the ancestral state according to each site’s posterior probability. Posterior probabilities ≥ 0.95 were classed as “high confidence”; lower probabilities were considered to be ambiguous and were marked as "N". The sequence from the crown group *Magnolia* species were defined as ancestral.

2.10 Inference of demographic history

A Stairway plot was used to infer the demographic history of *Magnolia sinica* [99]. The mutation rate was estimated as 1.2e-7 per locus per generation and the Stairway plot was constructed using MCMCTree based on the four-fold degenerated sites (4DTv sites) of orthologous family genes. Generation time was set as 30 years, based on the cultivation records of this species in KBG. Dataset 1 was further filtered by removing the sites within 5 kb of gene regions to ensure site neutrality. The unfolded Site Frequency Spectrum (SFS) for *M. sinica* was estimated using the functions doSaf and realSFS in ANGSD v 0.921 [93] with neutral sites and the recommended filtering parameters “-minMapQ 30 -minQ 20”.

We also used the Pairwise Sequentially Markovian Coalescent (PSMC) model to reconstruct the demographic history of *M. sinica* [100]. Using the BAM files generated by BWA-MAM and the markdup model in SAMtools [65], we made a consensus fastq file for each sample using SAMtools and BCFtools with the parameter set to -C50 to downgrade the mapping quality for reads containing excessive mismatches. The script vcfutils.pl was used to keep the minimum read depth to 5× and

the maximum read depth to 50 for all individuals. The consensus fastq file was converted into an input file for PSMC using fq2psmcfa with the parameter -q 20 set, to remove consensus calls with qualities ≤ 20. The PSMC analysis was run using default values for the upper limit to assign a date to most recent common ancestor (-t 15) and theta/rho (-r 5). The atomic time interval pattern (-p) was set to "4+30*2+4+6+10". We plotted the results using the same mutation rate and generation time as described above.

The contemporary effective population size of *Magnolia sinica* was assessed using the linkage disequilibrium method in NeEstimator V2 [93] with the reduced Dataset 5 (filtered by vcftools with --max missing 0.95 and --thin 60000) to ensure accuracy [101].

2.11 Estimation of deleterious mutations and inbreeding

Accumulation of deleterious mutations is likely to impact species fitness. The Sorting Intolerant from Tolerant (SIFT) algorithm [102] was used to predict deleterious mutations, with the ancestral sequences reconstructed above as a reference. The TrEMBL plant database [103] was used to search for orthologous genes. After polarization, protein-coding variants of Dataset2 were categorized as nonsynonymous and synonymous sites. Nonsynonymous sites were further divided into deleterious (SIFT score <0.05), and tolerated (SIFT score ≥0.05) based on their SIFT score [104]. We also calculated the derived allele frequency (DAF) of deleterious mutations. The deleterious mutations were annotated by performing gene ontology (GO) analysis and Kyoto Encyclopedia of Genes and Genomes (KEGG) analysis. GO terms and KEGG terms for the candidate genes with significant p value (<0.05) were retained.

In addition, frequency of runs of homozygosity (FROH) has been used as a robust estimate of genomic inbreeding [105] and was estimated following previous research [106, 107]. Briefly, runs

of homozygosity (ROH) were first identified using vcftools v0.1.17 [92], then FROH was calculated with the total length of ROH divided by the genome size of *M. sinica*.

Results

3.1 Genome sequencing and assembly

The libraries sequenced on the ONT PromethION platforms using 6 cells resulted in the generation of a total of 9.11 million reads with ~202.85 Gb sequencing data (~100×), with an average read length of 22 kb (the longest read was 194 kb, and N50 was 25 kb) (Table S3). A total of 1,432 million reads were generated with ca. 214.95 Gb (~110×) data using the Illumina HiSeq platform (Table S4). A total of 1,480 million reads with ca. 222.13 Gb data were produced with Hi-C sequencing (Table S5). Through the optimal assembly method, the final size of the assembled *Magnolia sinica* genome was 1.84 Gb, which was similar to the 1.9 Gb genome size estimated using k-mers (Figure S2, Table S10, S11). A total of 108 contigs (1.82 Gb, accounting for 99.08% of the whole genome) with an average size of 15 Mb were anchored onto the 19 chromosomes. The contigs N50 of the *M. sinica* genome was ca. 45 Mb and the scaffold N50 ca. 92 Mb, much higher than those of other previously reported magnolia genomes (Table 1) [36–38]. In addition, the mitochondrial and chloroplast genomes were assembled into circular DNA molecules of 856,922 bp and 160,070 bp, respectively. The complete core genes (including single- and multi-copy genes) account for 90.5% of the genome while the missing genes account for 6.7%, and the LAI value was estimated to be 10.3 based on LTR, indicating that the gene integrity was relatively good (Table S11, S12). We also calculated that the heterozygosity rate in *M. sinica* was about 1.21%, and that the error rate was about 0.0072%.

3.2 Genome annotation

A total of 2,329,558 repetitive sequences were identified in the *M. sinica* genome, with a total length of ~1.05 Gb, and accounting for 56.99 % genome. Of these, the highest proportion was LTR, accounting for 48.9% of the whole genome (Table S16). The most abundant repeat element families were Copia (388,301, 14.88 %) and Gypsy (759,932, 27.40 %) (Table S16). A total of 18 million subreads with ~24.58 Gb data were generated from transcriptome sequencing, from which 43,473 protein-coding genes were annotated (Table S6, S17). The mean lengths of gene region, transcript, and coding DNA sequences were 11,297, 1,552, and 1,091, respectively (Table S17). Moreover, 71 rRNA, 658 tRNA, and 511 ncRNA sequences were identified (Table S18). A total of 43,473 genes were annotated using GO (14,360, 33.03 %), KEGG (14,937, 34.36 %), eggnog (29,585, 68.05 %) and COG (31,414, 72.26 %). Based on sequence conservation, several protein databases, including Swiss-Prot (21,220, 48.81 %), TrEMBL (31,720, 72.96 %), NR (31,242, 71.87 %) and *Arabidopsis thaliana* (25,007, 57.52 %) were annotated with BLAT. For assignment based on domain conservation, certain other databases of *M. sinica* were annotated with IntreProScan. (Table S19)

1,303 (90.49 %) complete BUSCO genes, including 1,249 (86.74 %) complete and single-copy genes and 54 (3.75 %) complete and duplicated genes were identified among the 1,440 total BUSCO groups. However, 40 (2.78 %) genes were found to be fragmented and 97 (6.74 %) genes were missing based on the BUSCO analysis (Table S11).

3.3 Analysis of phylogeny, collinearity and WGD

In order to investigate the early evolution of the core angiosperms, we identified 579,290 homologous genes belong to 20,538 gene families from the 18 related genomes using OrthoFinder2. A total of 1,266 expanded and 1,276 contracted gene families in *Magnolia sinica* were identified and annotated (Fig 2c). A maximum likelihood tree was constructed using 1,070 orthogroups of 18

species. As shown in the ML phylogenetic tree (Fig 2c), magnolias formed a sister relationship with both the eudicots and the Ceratophyllales, while the monocots were sister to the other core angiosperms. The Magnoliales and the Laurales were predicted to have diverged from the Piperales at ca. 149.3 Ma (137.7–160), a result which was slightly different from that of a whole-genome study of black pepper, in which the differentiation time was estimated at 175–187 Ma [70]. The Magnoliales were predicted to have diverged from the Laurales at ca. 122.2 Ma. In the Magnoliales, the estimated differentiation time of the genera *Magnolia* and *Liriodendron* was predicted to be 23.4 Ma, and within Magnolia, the closely related species *M. sinica* and *M. biondii* are estimated to have diverged ca. 10.9 Ma.

A total of 7,807 colinear gene pairs on 779 colinear blocks were inferred within the *Magnolia sinica* genome. The collinearity between *M. sinica* and *Liriodendron chinense* was 1:1 (Figure S3), indicating that the two species have no species-specific whole-genome duplication (WGD) events. Collinearity between these two species and with earlier differentiated dicotyledons such as grapes was always 2:3 (Figure S4, S5), indicating that *M. sinica* and *L. chinense* experienced a WGD event after differentiation from the eudicots which is consistent with the conclusions of the *L. chinense* [36]. Similarly, the collinearity with the early angiosperms *Amborella trichopoda* and *Nymphaea tetragona* was 2:1 and 2:2 (Figure S6, S7), respectively, which indicates that *M. sinica* and *L. chinense* only experienced a single shared WGD event after their differentiation from these plants. From the paralogous collinearity block in *M. sinica*, it can be seen that this WGD event occurred at a Ks value of about 0.75. Based on the chromosome tree analysis, the Magnoliaceae and the Lauraceae share a WGD event, but this is not shared with pepper. After differentiation from other species, the Magnoliaceae (*M. sinica* and *L. chinense*) experienced a single WGD event, the

Lauraceae (*Cinnamomum kanehirae*) experienced two WGD events, and pepper experienced three WGD events.

3.4 Genome wide diversity and population structure

After filtering out low quality reads and adapter sequences, 5,386 million reads remained for processing (Table S20). The sequencing depth of *Magnolia sinica* samples ranged from 8.8× to 12.6×, with a mean value of 10.5×, and were between 10.8×–14.3× for the other eight *Magnolia* species (Table S20). The mapping rates of *M. sinica* ranged from 90.80% to 99.70%, with a mean value of 97.63 %, and were 95.30%–99.53% for the other eight *Magnolia* species (Table S20).

The mean heterozygosity rate of *M. sinica* was (1.29 ± 0.07) % (Table S21), ranging from 1.12 % to 1.38 %, and the trees with the lowest and the highest heterozygosity rates were both found in the XZQ population. The MAD population had the lowest heterozygosity (1.19 %), while the DLS population had the highest heterozygosity (1.32 %).

Nucleotide diversity in *M. sinica* was estimated using two parameters. Watterson's theta (θw) and genome wide diversity (θπ) of *M. sinica* were calculated as 0.01416 and 0.01494, respectively (Table S22). When compared with other species, *M. sinica* was found to have higher genetic diversity (Table S23), and was approximately 12 folds higher than that of *Liriodendron chinense* (0.00123), a species from Magnoliaceae estimated using BioPerl [36].

The population structure results showed that the CV error was smallest when there was an optimal number of clusters K = 1 (Figure S8), suggesting low genetic differentiation among populations of *M. sinica.* Low genetic differentiation among populations was further suggested by the low F_{st} statistics between population pairs of *M. sinica*, which had a mean value of 0.133. We have given the structure results for $K = 2$ and $K = 3$ in Figure 3b. At $K = 2$, all the populations of *M.*

sinica could be separated into three components, including an XZQ component (blue), the component (orange) from the FD population, and two individuals (KIBDZL15301 and KIBDZL15303) from the DLS population, as well as a mixture component. When *K* = 3, the FD population was further separated into two components, including an FD component and a mixture component. Both the XZQ and FD populations were genetically "pure" from the other *M. sinica* populations. The MAD and MC populations were genetically similar irrespective of *K*.

3.5 Demographic history

The demographic histories of *Magnolia sinica* inferred by Stairway plot2 indicate three significant population declines, two of which were also detected by PSMC (Figure 3c). In the scenario inferred from Stairway plot2, the earliest population decline occurred at 1.3 Ma and continued until 1.1 Ma. For the scenarios inferred by the PSMC, the earliest population decline occurred at 1.5 Ma and continued until 0.8 Ma. After this, the population of *M. sinica* is predicted to have experienced a period of recovery in both scenarios. The second population decline occurred at about 0.3 Ma in both scenarios. After that, the population of *M. sinica* exhibited recovery in the scenario inferred by Stairway plot2, but experienced a continuing decline in PSMC. The latest population bottleneck in both scenarios occurred at about 20 Ka and continued until 10 Ka, when the effective population size of *M. sinica* dropped to 1,936 in the Stairway plot and 1784 in PSMC. However, after 10 ka, the effective size of the *M. sinica* population recovered in Stairway plot, but showed continuous decline in PSMC. The contemporary effective population size of *M. sinica* estimated by NeEstimator V2 was 10.9 (3.3–43.7 Jackknife CI).

3.6 Genetic load and genomic inbreeding coefficient

1,196,374,340 high confidence loci were obtained and used as ancestral sequences to predict

deleterious mutations. 16,131, 74,385 and 36,827 sites were predicted to be deleterious, synonymous and tolerated, respectively, in the 21 re-sequenced *Magnolia sinica* individuals (Table S24). The mean value of derived homozygous deleterious alleles (HoDA) was 249, ranging from 190 to 298, with the lowest found in the MC population, which had a mean number of 207 (190–216), and the highest found in XZQ, which had a mean number of 258 (220–298) (Table S25). The MAD population also harbors a very high number of HoDA (246), and this population had highest proportion of private HoDA (118, 48%) when compared with other populations (Figure 3d, Table S25). None of the HoDA was shared among all five of these populations. An average of 2,607 heterozygous deleterious alleles (HeDA) was detected in *M. sinica*, ranging from 2,136 to 2,967. The highest number of HeDA was found in the XZQ population, which had a mean value of 2,593 (2,136–2,967) (Table S25), while the lowest number of HeDA was found in the MAD population (2,430). The MAD population shared the highest HeDA with the MC population, and shared the lowest HeDA with XZQ. None of the HeDA was shared among all five of the populations (Table S25). The derived allele frequency (DAF) of approximately 32.35% of the deleterious mutations was < 0.05, and all these rare deleterious mutations were heterozygous. Only ~7.1% (1147/16131) of the deleterious mutations were homozygous (DAF > 0.05) (Figure S9).

At the population level, the mean value of FROH in *M. sinica* was 0.11 ± 0.04, ranging from 0.08 to 0.16, with the lowest value found in the DLS population, and the highest value found in MAD. At the individual level, one individual (KIBDZL15801) from XZQ population showed the lowest levels of inbreeding, and had the lowest FROH value (0.06). The individual (KIBDZL15803) with the largest FROH value (0.21) was also found in XZQ population (Table S25).

GO analysis annotated many gene terms of deleterious mutations involved in the lipid

metabolism, lipid biosynthesis, lipid translocation, oxidation of lipid, lipid transport, membrane lipid biosynthetic, and galactolipid biosynthetic pathways. KEGG analysis also annotated some metabolic pathways of deleterious mutations related to lipids, including glycerolipid metabolism, sphingolipid metabolism, Steroid biosynthesis, alpha-Linolenic acid metabolism, and glycerophospholipid metabolism (Table S26, S27).

4. DISCUSSION

To date, only three species in the Magnoliaceae (*Liriodendron chinense*, *Magnolia officinalis* and *M. biondii*) have been the objects of in-depth genomic research, and this has been mainly from the perspective of confirming the phylogeny of the angiosperms, investigation of species differentiation and the biosynthesis of terpenoids. To date, no species in the family Magnoliaceae have been studied at a genome-wide level from the perspective of conservation [36–38]. From the aspect of conservation genomics, we report high-quality whole-genomic data from *M. sinica* (1.84 Gb with contigs N50 of ca. 45 Mb). This is superior to the data available from *Liriodendron chinense* (1.74 Gb with contigs N50 of ~1.43 Mb) [36], *Magnolia officinalis* (1.68 Gb, with contigs N50 of 0.22 Mb) [38] and *M. biondii* (2.22 Gb with contigs N50 of 0.27 Mb) [37].

The early evolution of the core angiosperms has been studied with whole-genome analysis of certain species of Magnoliids and Chloranthales [37, 68, 106, 108–111]. However, the phylogenetic relationships between the Magnoliids on the early branch of the angiosperm lineage and the eudicots and monocots have been controversial and not fully resolved [110, 111]. Our genome level phylogenetic tree suggests that the magnolias form a sister group to the eudicots and the Ceratophyllales, while the monocots are sister to the other core angiosperms. This is consistent with the results of a study into Chloranthales [106, 110], but inconsistent with the relevant results of

Magnolia biondii and *M. officinalis* [37, 38]. The evolutionary history of the angiosperms was accompanied by frequent WGD events. However, evidence of WGD events was inferred from dot plots and Ks, which is insufficient to demonstrate whether any two species very close to differentiation share a WGD event. In our study, we concatenated homologous genes to construct a chromosome-level tree to make our inferences more reliable. Our inference results suggest that WGD events also occurred after the differentiation of the magnoliids from other groups, which is in agreement with other studies [111].

Genetic diversity is essential to allow species evolution in response to environmental changes, and has been predicted to be positively correlated with species fitness and evolutionary potential [112]. We found that *M. sinica* had relatively high genetic diversity, which is consistent with previous research based on SSR markers [45]. This high diversity could be explained by the fact that, as a tree species, *M. sinica* has a long life span (ca. 30 years). De Kort et al. (2021) [114] compared the genetic diversity of 164 annuals, 1,405 perennials, 308 shrubs and 2,337 trees, and found that although species level diversity is lower for long-lived or low-fecundity species than for short-lived or high-fecundity species, population level genetic diversity is usually higher for long-living plants, as they may respond more slowly to reduced gene flow. Another reason for this high diversity could be that *M. sinica* is found in southern subtropical monsoon broadleaved evergreen forests [44]. Species around the equator are expected to have higher population-level genetic diversity than other species. This is because in theoretical prediction analyses, the abundant precipitation around the equator shows a significant relative contribution to population genetic diversity, although the exact mechanisms and extent of this are still unknown [113]. Moreover, the pollinator-dependent pollination system may contribute to the high genetic diversity in *M. sinica*

[45].

Magnolia sinica has low genetic differentiation between subpopulations, which could be attributed to higher gene flow among subpopulations, despite the fragmented distribution of the species [45]. The species has an outcrossing mating system, which is pollinator dependent, and two species of beetles appear to be effective pollinators [44]. Previous research has demonstrated that some beetles can fly up to 12 km [114]. Long-distance pollen-mediated gene flow among populations may decrease population genetic differentiation [115]. The smaller FROH and lower inbreeding load in *M. sinica* compared with *Acer yangbiense* may also indicate the existence of certain gene flow among its isolated populations [107], or from other populations which we have not found. As most of the reported populations of *M. sinica* are found on the borders of China with other countries, it is not unreasonable to suggest that other unreported individuals or populations exist outside China.

Southeast Yunnan is an important biodiversity hotspot [116], and is shielded by Ailao Mountain from the climate fluctuations caused by glaciation and the uplift of the Himalayas and the Hengduan Mountains [117]. From the geological point of view, there is no evidence that Southeast Yunnan was affected by the Quaternary ice age, and simulations of climate data suggest that this area was not seriously affected by the global temperature drop [118]. In our results, Stairway plot2 detected major population declines, which is similar to the inferred demographic history of the sympatric *Magnolia fistulosa* [119]. Each *M. sinica* population decline inferred in the Stairway plot could be verified in PSMC (Figure 3c). However, the demographic history of *M. sinica* inferred by Stairway plot2 shows population rebound after each decline, which was not obvious in the PSMC analysis. Moreover, the Stairway plot can estimate very recent events, while PSMC estimates only up to 10,000 years ago

(Figure 3c). The earliest inferred population decline occurred 1.0–1.2 Ma, which is consistent with the mid-Pleistocene transition [120]. Population declines at a similar time are also reflected in other sympatric species such as *Acer yangbiense* [107], and *Buddleja alternifolia* [106]. The second population decline occurred at 0.3 Ma, during which global temperature experienced a general decline [121]. The latest population decline occurred at ca. 20 Ka, and may have been caused by the Last Glacial Maximum (19.0–26.5 Ka) [122]. Multiple population declines may have resulted in a narrow distribution of *M. sinica*, and the stable population sizes from about 1 ka inferred in the Stairway plot may be as a result of the very recent large-scale anthropogenic land development and land use changes in the habitat of *M. sinica*, and is likely to have been responsible for the extremely rare status of this species [26], this is also consistent with the characteristics of high genetic diversity and low genetic differentiation of this species.

The MAD population contains only a single remnant individual with a higher level of inbreeding (FROH = 0.16), lower heterozygosity rate (1.19%) and higher homozygous deleterious allele number (246) than other populations. Gene flow has been proposed as a potential strategy to sustain small and isolated populations, by masking of deleterious alleles [123]. We found that the DLS population had a higher heterozygosity rate (1.32%) and shared few homozygous deleterious mutations with the MAD population. The DLS population could therefore serve as source material for breeding, which could be used to mask homozygous deleterious mutations in MAD population. Methods such as population reinforcement, hand pollination to assist pollen flow (by collecting pollen from DLS population and pollinating the MAD population), or the transplantation of seedlings from the DLS population into MAD could be considered. Similarly, an individual (KIBDZL15801) in the XZQ population also had a higher heterozygosity rate (1.37%), and a

smaller number of HoDA (220). Pollen from KIBDZL15801 could therefore be used to assist gene flow to KIBDZL15803 and KIBDZL15807, two individuals with lower heterozygosity rates (1.12 % and 1.16 %, respectively) and higher numbers of HoDA (298 and 286, respectively).

Identification of a management unit (MU) is essential for the management of natural populations [124]. The FD population was genetically pure, and had no admixture with other populations even when $K = 2$ and $K = 3$. This could be attributed to its distance from the other populations (about 66–145 km), which may decrease opportunities for pollen flow. Similarly, population XZQ was also found to be genetically pure at $K = 2$ and $K = 3$. We therefore suggest that the FD and XZQ populations be treated as two separate evolutionarily significant units (ESU). The MAD and MC populations were genetically similar at all values of K, and we suggest that they be treated as another ESU. Importantly, however, the MAD and MC populations are found outside any existing nature reserves, and it is therefore necessary to include these populations in a nature reserve or to establish specific conservation regions to protect them.

The main threats currently faced by *Magnolia sinica* are as follows: (1) Substantial reduction and loss of the original habitats leading to severe habitat fragmentation and population isolation; (2) The large-scale planting of *Amomum tsaoko* under forest cover means that *M. sinica* is unable to regenerate naturally in the wild, and there are no seedlings; (3) Excessive artificial seed collection. Fortunately, since 2005, because this plant is a critically endangered flagship species, comprehensive scientific research, including reproductive and seed biology, conservation genetics, and protection measures including field investigations, *in situ* conservation, *ex situ* conservation, and reintroduction have been gradually implemented [14, 44, 46, 47, 49]. At present, in addition to the existing protection measures, strengthening of the management of nature reserves and reduction

of the disturbance by human activities in the original habitats of wild populations are urgently needed. In particular, it is necessary to stop the large-scale planting of commercial crops (*Amomum tsaoko*) under these forests, which is important to restore their natural regeneration in the wild. Unlike most of the severely threatened species, *M. sinica* has high genetic diversity and low genetic differentiation which is also consistent with research into other endangered species in the Magnoliaceae [119, 125–127]. However, considering that the generation time of *M. sinica* can be as long as 30 years, the isolation of the various populations, the serious habitat fragmentation, and that there are very few wild individuals, we still need to consider potential future inbreeding depression. More artificial outcrossing strategies should be designed in the future to reduce the loss of genetic diversity caused by inbreeding, and that these strategies should be considered instead of collecting seeds and simply breeding more individuals [25]. Our genomic study into *M. sinica* provides an example of high genetic diversity and low genetic differentiation in a long-lived tree species and informs the future formation and maintenance of conservation strategies necessary for the survival of such a PSESP.

Data availability statement

The genome assembly, annotations, and other supporting data are available via the GigaScience database GigaDB. The raw sequence data have been deposited in the Short Read Archive under NCBI BioProject ID PRJNA774088.

Additional Files

Figure S1. Evaluate the distribution of the coverage depth of the whole genome and BUSCO core gene region with the data of ilumina and ont.

Figure S2. Kmer frequency distribution diagram.

Figure S3. The collinearity between *M. sinica* and *Liriodendron chinense*.

Figure S4. The collinearity between *M. sinica* and *Vitis vinifera.*

Figure S5. The collinearity between *Liriodendron chinense* and *Vitis vinifera*.

Figure S6. The collinearity between *Amborella trichopoda* and *M. sinica*.

Figure S7. The collinearity between *Nymphaea colorata* and *M. sinica*.

Figure S8. Cross validation error (CV) based on Admixture output.

Figure S9. Deleterious allele frequency distribution of homozygous deleterious SNPs. The density on the left of y axis is the number of alleles in a given allele frequency.

Table S1. Collection information of 21 re-sequenced samples of Magnolia sinica.

Table S2. Collection information of other eight Magnoliaceae samples for re-sequencing.

Table S3. WGS-ONT sequencing statics.

Table S4. WGS-Illumina sequencing statics.

Table S5. HiC sequencing statics.

Table S6. Iso-Seq sequencing statics.

Table S7. Assembly statics (V0.1).

Table S8. Assembly statics (V0.2).

Table S9. Assembly statics (V0.3).

Table S10. Assembly statics (V1.0).

Table S11. Assembly statics (V1.1).

Table S12. Statistics of all assemblies.

Table S13. The information of the chromosomes, scattered sequences, chloroplasts and

mitochondria.

Table S14. The mapping ratio and coverage percentage.

Table S15. Sequences used to construct ancestral sequences.

Table S16. The repetitive sequences statics.

Table S17. The final gene gff3 statics.

Table S18. Statistics of the source of integration annotation.

Table S19. Gene annotation statistics.

Table S20. Genome mapping statistics.

Table S21. Statistics of heterozygsity rate.

Table S22. Mean population fixation index and corresponding spatial distance.

Table S23. Genome wide diversity of woody speices.

Table S24. SIFT (Sorting Intolerant From Tolerant) prediction of deleterious mutations.

Table S25. Geneticl load of 21 individuals of Magnolia sinia.

Table S26. GO (gene ontology) enrichment of the deleterious mutations.

Table S27. KEGG pathway of the deleterious mutations.

Abbreviations

AED: annotation edit distance; Blast: Basic Local Alignment Search Tool; BUSCO: Benchmarking Universal Single-copy Orthologues; CBD COP 15: 15th Conference of the Parties, Convention on Biological Diversity; DAF: derived allele frequency; ESTs: Expressed sequence tags; FROH: frequency of runs of homozygosity; GO: gene ontology; HeDA: heterozygous deleterious alleles; HoDA: homozygous deleterious alleles; JBAT: Juicebox Assembly Tools; KBG : Kunming

Botanical Garden; KEGG: Kyoto Encyclopedia of Genes and Genomes; ONT: Oxford Nanopore Technologies; LAI: the LTR Assembly Index; LTR: long terminal repeat retrotransposons; MAF: minor allele frequency; PSESP: Plant Species with Extremely Small Populations; PSMC: Pairwise Sequentially Markovian Coalescent; ROH: runs of homozygosity; θW: Watterson's theta; $\theta\pi$: nucleotide diversity; SFS: Site Frequency Spectrum; SIFT: Sorting Intolerant from Tolerant; SMRT: Single Molecule Real-Time; WGD: whole-genome duplication.

Competing interests

The authors declare no competing interests.

Authors' contributions

Y.P.M. and W.B.S. conceived and designed the study; R.G.Z., L.C., D.T.L. F.M.Y. and Q.Z.Y. analyzed the data; L.C., D.T.L. and F.M.Y. wrote the manuscript; Y.P.M., Z.L.D. and W.B. S. revised the manuscript. All authors reviewed and approved the final manuscript.

Acknowledgements

We thank Li-Dan Tao, Pin Zhang, Jia-Jun Yang, Rong-Li Liao for their help in collecting materials, and we thank Li-Sen Qian for helping to write the R script.

Funding

This work was supported by the National Science & Technology Basic Resources Investigation Program of China (Grant No. 2017FY100100); Yunnan Fundamental Research Projects (Grant No. 202101AT070173); National Natural Science Foundation of China (NSFC) (Grant No. 32101407); and the National Natural Science Foundation of China (NSFC) – Yunnan Joint Fund (Grant No. U1302262).

References

1. Berry PM, Fabók V, Blicharska M, et al. Why conserve biodiversity? A multi-national exploration of stakeholders' views on the arguments for biodiversity conservation. *Biodivers Conserv.* 2018;**27**(7):1741–62. doi: 10.1007/s10531-016-1173-z.
2. Chen GS. Analysis on biodiversity conservation in the pluralistic vision. *Advanced Materials Research*. 2012;**518-523**:4980–4. doi: 10.4028/www.scientific.net/AMR.518-523.4980.
3. Isbell F, Gonzalez A, Loreau M, et al. Linking the influence and dependence of people on biodiversity across scales. *Nature*. 2017;**546**(7656):65–72. doi: 10.1038/nature22899.
4. Johnson CN, Balmford A, Brook BW, et al. Biodiversity losses and conservation responses in the Anthropocene. *Science*. 2017;**356**(6335):270–5. doi: 10.1126/science.aam9317.
5. Meng HH, Zhou SS, Li L, et al. Conflict between biodiversity conservation and economic growth: insight into rare plants in tropical China. *Biodivers Conserv*. 2019;**28**(2):523–37. doi: 10.1007/s10531-018-1661-4.
6. Wang W, Feng CT, Liu FZ, et al. Biodiversity conservation in China: A review of recent studies and practices. *Environ Sci Ecotech*. 2020;**2**:100025. doi: 10.1016/j.ese.2020.100025.
7. Zhang YZ, Qian LS, Spalink D, et al. Spatial phylogenetics of two topographic extremes of the Hengduan Mountains in southwestern China and its implications for biodiversity conservation. *Plant Divers*. 2021;**43**(3):181–91. doi: 10.1016/j.pld.2020.09.001.
8. McBeath J and McBeath† JH. Biodiversity conservation in China: policies and practice. *J. Int. Wildl. Law Policy*. 2006;**9**(4):293–317. doi: 10.1080/13880290601039238.
9. Xu Jc and Wilkes A. Biodiversity impact analysis in northwest Yunnan, southwest China.

Biodivers Conserv. 2004;**13**(5):959–83. doi: 10.1023/B:BIOC.0000014464.80847.02.

10. Wyse Jackson P and Kennedy K. The Global Strategy for Plant Conservation: a challenge and opportunity for the international community. *Trends Plant Sci.* 2009;**14**(11):578–80. doi: 10.1016/j.tplants.2009.08.011.

11. López-Pujol J, Zhang FM and Ge S. Plant biodiversity in China: richly varied, endangered, and in need of conservation. *Biodivers Conserv*. 2006;**15**(12):3983–4026. doi: 10.1007/s10531-005-3015-2.

12. Ma YP, Chen G, Grumbine RE, et al. Conserving plant species with extremely small populations (PSESP) in China. *Biodivers Conserv*. 2013;**22**(3):803–9. doi: 10.1007/s10531-013-0434-3.

13. Sun WB, Ma YP and Blackmore S. How a new conservation action concept has accelerated plant conservation in China. *Trends Plant Sci*. 2019;**24**(1):4–6. doi: 10.1016/j.tplants.2018.10.009.

14. Sun WB, Yang J and Dao ZL. Study and Conservation of Plant Species with Extremely Small Populations (PSESP) in Yunnan Province, China. Beijing: Science Press; 2019.

15. Yang J, Cai L, Liu DT, et al. China's conservation program on Plant Species with Extremely Small Populations (PSESP): Progress and perspectives. *Biol Conserv*. 2020;**244**:108535. doi: 10.1016/j.biocon.2020.108535.

16. Sun WB. List of Yunnan Protected Plant Species with Extremely Small Populations (2021). Kunming: Yunnan Science and Technology Press; 2021.

17. Marks RA, Hotaling S, Frandsen PB, et al. Representation and participation across 20 years of plant genome sequencing. *Nat Plants*. 2021;**7**(12):1571–8. doi: 10.1038/s41477-021-

01031-8.

18. Zanini SF, Bayer PE, Wells R, et al. Pangenomics in crop improvement—from coding structural variations to finding regulatory variants with pangenome graphs. *Plant Genome*. 2022;**15**(1):e20177. doi: 10.1002/tpg2.20177.

19. Chen Y, Ma T, Zhang LS, et al. Genomic analyses of a "living fossil": The endangered dove-tree. *Mol Ecol Resour*. 2020;**20**(3):756–69. doi: 10.1111/1755-0998.13138.

20. Ding XP, Mei WL, Huang SZ, et al. Genome survey sequencing for the characterization of genetic background of *Dracaena cambodiana* and its defense response during dragon's blood formation. *PLoS One*. 2018;**13**(12):e0209258. doi: 10.1371/journal.pone.0209258.

21. Liu HL, Wang XB, Wang GB, et al. The nearly complete genome of *Ginkgo biloba* illuminates gymnosperm evolution. *Nat Plants*. 2021;**7**(6):748–56. doi: 10.1038/s41477-021-00933-x.

22. Ma H, Liu YB, Liu DT, et al. Chromosome-level genome assembly and population genetic analysis of a critically endangered rhododendron provide insights into its conservation. *Plant J*. 2021;**107**(5):1533–45. doi: 10.1111/tpj.15399.

23. Rodríguez del Río Á, Minoche AE, Zwickl NF, et al. Genomes of the wild beets *Beta patula* and *Beta vulgaris* ssp. *maritima*. *Plant J*. 2019;**99**(6):1242–53. doi: 10.1111/tpj.14413.

24. Sun YX, Deng T, Zhang AD, et al. Genome sequencing of the endangered *Kingdonia uniflora* (Circaeasteraceae, Ranunculales) reveals potential mechanisms of evolutionary specialization. *iScience*. 2020;**23**(5):101124. doi: 10.1016/j.isci.2020.101124.

25. Yang YZ, Ma T, Wang ZF, et al. Genomic effects of population collapse in a critically endangered ironwood tree *Ostrya rehderiana*. *Nat Commun*. 2018;**9**(1):5449. doi:

10.1038/s41467-018-07913-4.

26. Yang J, Wariss HM, Tao LD, et al. *De novo* genome assembly of the endangered *Acer yangbiense*, a plant species with extremely small populations endemic to Yunnan Province, China. *GigaScience*. 2019;**8**(7):giz085. doi: 10.1093/gigascience/giz085.

27. Xu B, Liao M, Deng HN, et al. Chromosome-level *de novo* genome assembly and whole-genome resequencing of the threatened species *Acanthochlamys bracteata* (Velloziaceae) provide insights into alpine plant divergence in a biodiversity hotspot. *Mol Ecol Resour*. 2022;**22**(4):1582–95. doi: 10.1111/1755-0998.13562.

28. Zhu SS, Chen J, Zhao J, et al. Genomic insights on the contribution of balancing selection and local adaptation to the long-term survival of a widespread living fossil tree, *Cercidiphyllum japonicum*. *New Phytol*. 2020;**228**(5):1674–89. doi: 10.1111/nph.16798.

29. Dong SS, Wang YL, Xia NH, et al. Plastid and nuclear phylogenomic incongruences and biogeographic implications of *Magnolia* s.l. (Magnoliaceae). *J Syst Evol*. 2022;**60**(1):1–15. doi: 10.1111/jse.12727.

30. Wang YB, Liu BB, Nie ZL, et al. Major clades and a revised classification of *Magnolia* and Magnoliaceae based on whole plastid genome sequences via genome skimming. *J Syst Evol*. 2020;**58**(5):673–95. doi: 10.1111/jse.12588.

31. Azuma H, García-Franco JG, Rico-Gray V, et al. Molecular phylogeny of the Magnoliaceae: the biogeography of tropical and temperate disjunctions. *Am J Bot*. 2001;**88**(12):2275–85. doi: 10.2307/3558389.

32. Figlar RB and Nooteboom HP. Notes on Magnoliaceae IV. *Blumea-Biodiversity, Evolution and Biogeography of Plants*. 2004;**49**(1):87–100. doi: 10.3767/000651904X486214.

33. Rivers M, Beech E, Murphy L, et al. The red list of Magnoliaceae-revised and extended. Richmond: Botanic Gardens Conservation International; 2016.

34. Xia NH, Liu YH, Nooteboom HP. Magnoliaceae. In: Wu ZY, Raven PH , editors. *Flora of China*, Vol. 7. Beijing: Science Press & St. Louis: Missouri Botanical Garden Press; 2008. p. 48–91.

35. Qin HN, Yang Y, Dong SY, et al. Threatened species list of China's higher plants. *Biodiv Sci*. 2017;**25**(7):696–744. doi: 10.17520/biods.2017144.

36. Chen JH, Hao ZD, Guang XM, et al. *Liriodendron* genome sheds light on angiosperm phylogeny and species–pair differentiation. *Nat Plants*. 2019;**5**:18–25. doi: 10.1038/s41477-018-0323-6.

37. Dong SS, Liu M, Liu Y, et al. The genome of *Magnolia biondii* Pamp. provides insights into the evolution of Magnoliales and biosynthesis of terpenoids. *Hortic Res*. 2021;**8**:38. doi: 10.1038/s41438-021-00471-9.

38. Yin YP, Peng F, Zhou LJ, et al. The chromosome-scale genome of *Magnolia officinalis* provides insight into the evolutionary position of magnoliids. *iScience*. 2021;**24**(9):102997. doi: 10.1016/j.isci.2021.102997.

39. Yuh-wu L. A new genus of Magnoliaceae from China. *J Syst Evol.* 1979;**17**:72–4.

40. Sun WB, Zhou Y, Li XY, et al. Population reinforcing program for *Magnolia sinica*, a critically endangered endemic tree in southeast Yunnan province, China. In: Maschinski J and Haskins KE, editors. Plant Reintroduction in a Changing Climate. Washington: Island Press; 2012. p. 65–69.

41. Wang S and Xie Y. China Species Red List (Vol 1). Beijing: Higher Education Press; 2004.

42. Cicuzza D, Newton A and Oldfield S. The red list of Magnoliaceae. Cambridge: Lavenham Press; 2007.

43. Anon. List of National Key Protected Wild Plants (First Group). *The Order of National Forestry Bureau and Agriculture Ministry of China 4*, 2–13.

44. Chen Y, Chen G, Yang J, et al. Reproductive biology of *Magnolia sinica* (Magnoliaecea), a threatened species with extremely small populations in Yunnan, China. *Plant Divers*. 2016;**38**(5):253–8. doi: 10.1016/j.pld.2016.09.003.

45. Chen Y. Conservation biology of *Manglietiastrum sinicum* Law (Magnoliaceae), a plant species with extremely small populations. University of Chinese Academy of Sciences; 2017.

46. Lin L, Cai L, Fan L, et al. Seed dormancy, germination and storage behavior of *Magnolia sinica*, a plant species with extremely small populations of Magnoliaceae. *Plant Divers*. 2022;**44**(1):94–100. doi: 10.1016/j.pld.2021.06.009.

47. Song EJ, Park S, Sun WB, et al. Complete chloroplast genome sequence of *Magnolia sinica* (Y.W.Law) Noot. (magnoliaceae), A critically endangered species with extremely small populations in Magnoliaceae. *Mitochondrial DNA B*. 2019;**4**(1):242–3. doi: 10.1080/23802359.2018.1546141.

48. Su DF, Shen QQ, Yang JY, et al. Comparison of the bulk and rhizosphere soil prokaryotic communities between wild and reintroduced *Manglietiastrum sinicum* plants, a threatened species with extremely small populations. *Curr Microbiol.* 2021;**78**(11):3877–90. doi: 10.1007/s00284-021-02653-z.

49. Wang B, Ma YP, Chen G, et al. Rescuing *Magnolia sinica* (Magnoliaceae), a Critically

Endangered species endemic to Yunnan, China. *Oryx*. 2016;**50**(3):446–9. doi: 10.1017/S0030605315000435.

50. Doyle JJ and Doyle JL. A rapid DNA isolation procedure for small quantities of fresh leaf tissue. *Phytochem. bull*. 1987;**19**(1):11–5.

51. Gonzalez-Garay ML. Introduction to isoform sequencing using pacific biosciences technology (Iso-Seq). In: Wu JQ, editor. Transcriptomics and Gene Regulation. Dordrecht: Springer Netherlands; 2016. p. 141–60.

52. Myers EW, Sutton GG, Delcher AL, et al. A whole-genome assembly of *Drosophila*. *Science*. 2000;**287**(5461):2196–204. doi: 10.1126/science.287.5461.2196.

53. Walker BJ, Abeel T, Shea T, et al. Pilon: an integrated tool for comprehensive microbial variant detection and genome assembly improvement. *PLoS One*. 2014;**9**(11):e112963. doi: 10.1371/journal.pone.0112963.

54. Chakraborty M, Baldwin-Brown JG, Long AD, et al. Contiguous and accurate *de novo* assembly of metazoan genomes with modest long read coverage. *Nucleic Acids Res*. 2016;**44**(19):e147–e. doi: 10.1093/nar/gkw654.

55. Jin JJ, Yu WB, Yang JB, et al. GetOrganelle: a fast and versatile toolkit for accurate de novo assembly of organelle genomes. *Genome Biol*. 2020;**21**(1):241. doi: 10.1186/s13059-020-02154-5.

56. Dudchenko O, Batra SS, Omer AD, et al. De novo assembly of the *Aedes aegypti* genome using Hi-C yields chromosome-length scaffolds. *Science*. 2017;**356**(6333):92–5. doi: 10.1126/science.aal3327.

57. Durand NC, Shamim MS, Machol I, et al. Juicer provides a One-Click system for analyzing

loop-resolution Hi-C experiments. *Cell Syst.* 2016;**3**(1):95–8. doi: 10.1016/j.cels.2016.07.002.

58. Durand NC, Robinson JT, Shamim MS, et al. Juicebox provides a visualization system for Hi-C contact maps with unlimited zoom. *Cell Syst.* 2016;**3**(1):99–101. doi: 10.1016/j.cels.2015.07.012.

59. Hu J, Fan JP, Sun ZY, et al. NextPolish: a fast and efficient genome polishing tool for long-read assembly. *Bioinformatics*. 2019;**36**(7):2253–5. doi: 10.1093/bioinformatics/btz891.

60. Xu GC, Xu TJ, Zhu R, et al. LR_Gapcloser: a tiling path-based gap closer that uses long reads to complete genome assembly. *GigaScience*. 2018;**8**(1):giy157. doi: 10.1093/gigascience/giy157.

61. Pryszcz LP and Gabaldón T. Redundans: an assembly pipeline for highly heterozygous genomes. *Nucleic Acids Res*. 2016;**44**(12):e113. doi: 10.1093/nar/gkw294.

62. Simão FA, Waterhouse RM, Ioannidis P, et al. BUSCO: assessing genome assembly and annotation completeness with single-copy orthologs. *Bioinformatics*. 2015;**31**(19):3210–2. doi: 10.1093/bioinformatics/btv351.

63. Li H. Aligning sequence reads, clone sequences and assembly contigs with BWA-MEM. *ArXiv.* 2013:1303.3997. doi: 10.48550/arXiv.1303.3997.

64. Ou SJ and Jiang N. LTR_retriever: a highly accurate and sensitive program for identification of long terminal repeat retrotransposons. *Plant Physiol.* 2018;**176**(2):1410–22. doi: 10.1104/pp.17.01310.

65. Li H, Handsaker B, Wysoker A, et al. The Sequence Alignment/Map format and SAMtools. *Bioinformatics*. 2009;**25**(16):2078–9. doi: 10.1093/bioinformatics/btp352.

66. Haas BJ, Delcher AL, Mount SM, et al. Improving the *Arabidopsis* genome annotation using maximal transcript alignment assemblies. *Nucleic Acids Res*. 2003;**31**(19):5654–66. doi: 10.1093/nar/gkg770.

67. Stanke M, Diekhans M, Baertsch R, et al. Using native and syntenically mapped cDNA alignments to improve *de novo* gene finding. *Bioinformatics*. 2008;**24**(5):637–44. doi: 10.1093/bioinformatics/btn013.

68. Chaw SM, Liu YC, Wu YW, et al. Stout camphor tree genome fills gaps in understanding of flowering plant genome evolution. *Nat Plants.* 2019;**5**(1):63–73. doi: 10.1038/s41477-018-0337-0.

69. Soltis DE and Soltis PS. Nuclear genomes of two magnoliids. *Nat Plants.* 2019;**5**(1):6–7. doi: 10.1038/s41477-018-0344-1.

70. Hu LS, Xu ZP, Wang MJ, et al. The chromosome-scale reference genome of black pepper provides insight into piperine biosynthesis. *Nat Commun*. 2019;**10**(1):4702. doi: 10.1038/s41467-019-12607-6.

71. Albert VA, Barbazuk WB, dePamphilis CW, et al. The *Amborella* genome and the evolution of flowering plants. *Science.* 2013;**342**(6165):1241089. doi: 10.1126/science.1241089.

72. The Arabidopsis Genome Initiative. Analysis of the genome sequence of the flowering plant *Arabidopsis thaliana*. *Nature*. 2000;**408**(6814):796–815. doi:10.1038/35048692.

73. Cantarel BL, Korf I, Robb SM, et al. MAKER: an easy-to-use annotation pipeline designed for emerging model organism genomes. *Genome Res*. 2008;**18**(1):188–96. doi: 10.1101/gr.6743907.

74. Slater GSC and Birney E. Automated generation of heuristics for biological sequence

comparison. *Bmc Bioinformatics*. 2005;**6**(1):31. doi: 10.1186/1471-2105-6-31.

75. Lowe TM and Eddy SR. tRNAscan-SE: a program for improved detection of transfer RNA genes in genomic sequence. *Nucleic Acids Res.* 1997;**25**(5):955–64. doi: 10.1093/nar/25.5.955.

76. Huerta-Cepas J, Forslund K, Coelho LP, et al. Fast genome-wide functional annotation through orthology assignment by eggNOG-Mapper. *Mol Biol Evol.* 2017;**34**(8):2115–22. doi: 10.1093/molbev/msx148.

77. Buchfink B, Xie C and Huson DH. Fast and sensitive protein alignment using DIAMOND. *Nat Methods*. 2015;**12**(1):59–60. doi:10.1038/nmeth.3176.

78. Jones P, Binns D, Chang H-Y, et al. InterProScan5: genome-scale protein function classification. *Bioinformatics.* 2014;**30**(9):1236–40. doi: 10.1093/bioinformatics/btu031.

79. Emms DM and Kelly S. OrthoFinder: phylogenetic orthology inference for comparative genomics. *Genome Biol.* 2019;**20**(1):238. doi: 10.1186/s13059-019-1832-y.

80. Nguyen L-T, Schmidt HA, von Haeseler A, et al. IQ-TREE: a fast and effective stochastic algorithm for estimating maximum-likelihood phylogenies. *Mol Biol Evol.* 2015;**32**(1):268–74. doi: 10.1093/molbev/msu300.

81. Yang ZH. PAML 4: phylogenetic analysis by maximum likelihood. *Mol Biol Evol.* 2007;**24**(8):1586–91. doi: 10.1093/molbev/msm088.

82. Li HT, Yi TS, Gao LM, et al. Origin of angiosperms and the puzzle of the Jurassic gap. *Nat Plants.* 2019;**5**(5):461–70. doi: 10.1038/s41477-019-0421-0.

83. Magallón S, Gómez-Acevedo S, Sánchez-Reyes LL, et al. A metacalibrated time-tree documents the early rise of flowering plant phylogenetic diversity. *New Phytol.*

2015;**207**(2):437–53. doi: 10.1111/nph.13264.

84. Hahn MW, De Bie T, Stajich JE, et al. Estimating the tempo and mode of gene family evolution from comparative genomic data. *Genome Res.* 2005;**15**(8):1153–60. doi: 10.1101/gr.3567505.

85. Wang YP, Tang HB, DeBarry JD, et al. MCScanX: a toolkit for detection and evolutionary analysis of gene synteny and collinearity. *Nucleic Acids Res*. 2012;**40**(7):e49. doi: 10.1093/nar/gkr1293.

86. Edgar RC. MUSCLE: multiple sequence alignment with high accuracy and high throughput. *Nucleic Acids Res.* 2004;**32**(5):1792–7. doi: 10.1093/nar/gkh340.

87. Suyama M, Torrents D and Bork P. PAL2NAL: robust conversion of protein sequence alignments into the corresponding codon alignments. *Nucleic Acids Res.* 2006;**34**(suppl_2):609–12. doi: 10.1093/nar/gkl315.

88. Yang ZH and Nielsen R. Estimating synonymous and nonsynonymous substitution rates under realistic evolutionary models. *Mol Biol Evol.* 2000;**17**(1):32–43. doi: 10.1093/oxfordjournals.molbev.a026236.

89. Zhang Z, Li J, Zhao XQ, et al. KaKs_Calculator: calculating Ka and Ks through model selection and model averaging. *Genom Proteom Bioinf.* 2006;**4**(4):259–63. doi: 10.1016/S1672-0229(07)60007-2.

90. Chen SF, Zhou YQ, Chen YR, et al. Fastp: an ultra-fast all-in-one FASTQ preprocessor. *Bioinformatics.* 2018;**34**(17):884–90. doi: 10.1093/bioinformatics/bty560.

91. Garrison E and Marth G. Haplotype-based variant detection from short-read sequencing. *ArXiv*. 2012:1207.3907. doi: 10.48550/arXiv.1207.3907.

92. Danecek P, Auton A, Abecasis G, et al. The variant call format and VCFtools. *Bioinformatics*. 2011;**27**(15):2156–8. doi: 10.1093/bioinformatics/btr330.

93. Korneliussen TS, Albrechtsen A and Nielsen R. ANGSD: analysis of next generation sequencing data. *Bmc Bioinformatics*. 2014;**15**(1):356. doi: 10.1186/s12859-014-0356-4.

94. Watterson GA. On the number of segregating sites in genetical models without recombination. *Theor Popul Biol.* 1975;**7**(2):256–76. doi: 10.1016/0040-5809(75)90020-9.

95. Tajima F. Statistical method for testing the neutral mutation hypothesis by DNA polymorphism. *Genetics*. 1989;**123**(3):585–95. doi: 10.1093/genetics/123.3.585.

96. Fu YX and Li WH. Statistical tests of neutrality of mutations. *Genetics*. 1993;**133**(3):693–709. doi: 10.1093/genetics/133.3.693.

97. Purcell S, Neale B, Todd-Brown K, et al. PLINK: a tool set for whole-genome association and population-based linkage analyses. *Am J Hum Genet*. 2007;**81**(3):559–75. doi: 10.1086/519795.

98. Alexander DH, Novembre J and Lange K. Fast model-based estimation of ancestry in unrelated individuals. *Genome Res*. 2009;**19**(9):1655–64. doi: 10.1101/gr.094052.109.

99. Liu XM and Fu YX. Stairway Plot 2: demographic history inference with folded SNP frequency spectra. *Genome Biol*. 2020;**21**(1):280. doi: 10.1186/s13059-020-02196-9.

100. Li H and Durbin R. Inference of human population history from individual whole-genome sequences. *Nature*. 2011;**475**(7357):493–6. doi: 10.1038/nature10231.

101. Do C, Waples RS, Peel D, et al. NeEstimator v2: re-implementation of software for the estimation of contemporary effective population size (Ne) from genetic data. *Mol Ecol Resour.* 2014;**14**(1):209–14. doi: 10.1111/1755-0998.12157.

102. Sim N-L, Kumar P, Hu J, et al. SIFT web server: predicting effects of amino acid substitutions on proteins. *Nucleic Acids Res.* 2012;**40**(W1):452–7. doi: 10.1093/nar/gks539.

103. Boeckmann B, Bairoch A, Apweiler R, et al. The SWISS-PROT protein knowledgebase and its supplement TrEMBL in 2003. *Nucleic Acids Res.* 2003;**31**(1):365–70. doi: 10.1093/nar/gkg095.

104. Bortoluzzi C, Bosse M, Derks MFL, et al. The type of bottleneck matters: Insights into the deleterious variation landscape of small managed populations. *Evol Appl.* 2020;**13**(2):330–41. doi: 10.1111/eva.12872.

105. Kirin M, McQuillan R, Franklin CS, et al. Genomic Runs of Homozygosity record population history and consanguinity. *PLoS One*. 2010;**5**(11):e13996. doi: 10.1371/journal.pone.0013996.

106. Ma YP, Wariss HM, Liao RL, et al. Genome-wide analysis of butterfly bush (*Buddleja alternifolia*) in three uplands provides insights into biogeography, demography and speciation. *New Phytol.* 2021;**232**(3):1463–76. doi: 10.1111/nph.17637.

107. Ma YP, Liu DT, Wariss HM, et al. Demographic history and identification of threats revealed by population genomic analysis provide insights into conservation for an endangered maple. *Mol Ecol.* 2022;**31**(3):767–79. doi: 10.1111/mec.16289.

108. Chen YC, Li Z, Zhao YX, et al. The *Litsea* genome and the evolution of the laurel family. *Nat Commun.* 2020;**11**(1):1675. doi: 10.1038/s41467-020-15493-5.

109. Chen SP, Sun WH, Xiong YF, et al. The *Phoebe* genome sheds light on the evolution of magnoliids. *Hortic Res*. 2020;**7**:146. doi: 10.1038/s41438-020-00368-z.

110. Guo X, Fang DM, Sahu SK, et al. *Chloranthus* genome provides insights into the early

diversification of angiosperms. *Nat Commun*. 2021;**12**(1):6930. doi: 10.1038/s41467-021-26922-4.

111. Qin LY, Hu YH, Wang JP, et al. Insights into angiosperm evolution, floral development and chemical biosynthesis from the *Aristolochia fimbriata* genome. *Nat Plants.* 2021;**7**(9):1239–53. doi: 10.1038/s41477-021-00990-2.

112. Reed DH and Frankham R. Correlation between fitness and genetic diversity. *Conserv Biol.* 2003;**17**(1):230–7. doi: 10.1046/j.1523-1739.2003.01236.x.

113. De Kort H, Prunier JG, Ducatez S, et al. Life history, climate and biogeography interactively affect worldwide genetic diversity of plant and animal populations. *Nat Commun.* 2021;**12**(1):516. doi: 10.1038/s41467-021-20958-2.

114. Lessio F, Pisa CG, Picciau L, et al. An immunomarking method to investigate the flight distance of the Japanese beetle. *Entomol Gen*. 2022;**42**(1):45–56. doi: 10.1127/entomologia/2021/1117.

115. Gamba D and Muchhala N. Global patterns of population genetic differentiation in seed plants. *Mol Ecol.* 2020;**29**(18):3413–28. doi: 10.1111/mec.15575.

116. Li R and Yue J. A phylogenetic perspective on the evolutionary processes of floristic assemblages within a biodiversity hotspot in eastern Asia. *J Syst Evol.* 2020;**58**(4):413–22. doi: 10.1111/jse.12539.

117. Zhang KY. A preliminary study on the climatic characteristic and the formation factors in southern Yunnan. *Acta Meteorol Sin.* 1963;**33**(2):218–230.

118. Qian H and Ricklefs RE. Diversity of temperate plants in east Asia. *Nature*. 2001;**413**(6852):130–. doi: 10.1038/35093169.

119. Yang FM, Cai L, Dao ZL, et al. Genomic data reveals population genetic and demographic history of *Magnolia fistulosa* (Magnoliaceae), a Plant Species With Extremely Small Populations in Yunnan Province, China. *Front Plant Sci.* 2022;**13**:811312. doi: 10.3389/fpls.2022.811312.

120. Clark PU, Archer D, Pollard D, et al. The middle Pleistocene transition: characteristics, mechanisms, and implications for long-term changes in atmospheric pCO2. *Quat Sci Rev.* 2006;**25**(23):3150–84. doi: 10.1016/j.quascirev.2006.07.008.

121. Sun YB and An ZS. Late Pliocene-Pleistocene changes in mass accumulation rates of eolian deposits on the central Chinese Loess Plateau. *J Geophys Res-Atmos.* 2005;**110**(D23):D23101. doi: 10.1029/2005JD006064.

122. Clark PU, Dyke AS, Shakun JD, et al. The Last Glacial Maximum. *Science.* 2009;**325**(5941):710–4. doi: 10.1126/science.1172873.

123. Khan A, Patel K, Shukla H, et al. Genomic evidence for inbreeding depression and purging of deleterious genetic variation in Indian tigers. *Proc Natl Acad Sci.* 2021;**118**(49):e2023018118. doi: 10.1073/pnas.2023018118.

124. Palsbøll PJ, Bérubé M and Allendorf FW. Identification of management units using population genetic data. *Trends Ecol Evol.* 2007;**22**(1):11–6. doi: 10.1016/j.tree.2006.09.003.

125. Deng YW, Liu TT, Xie YQ, et al. High genetic diversity and low differentiation in *Michelia shiluensis*, an Endangered magnolia species in South China. *Forests.* 2020;**11**(4):469. doi: 10.3390/f11040469.

126. Yu HH, Yang ZL, Sun B, et al. Genetic diversity and relationship of endangered plant

Magnolia officinalis (Magnoliaceae) assessed with ISSR polymorphisms. *Biochem Syst Ecol.* 2011;**39**(2):71–8. doi: 10.1016/j.bse.2010.12.003.

127. Zhao XF, Ma YP, Sun WB, et al. High genetic diversity and low differentiation of *Michelia coriacea* (Magnoliaceae), a critically endangered endemic in southeast Yunnan, China. *Int J Mol Sci.* 2012;**13**(4):4396–411. doi: 10.3390/ijms13044396.

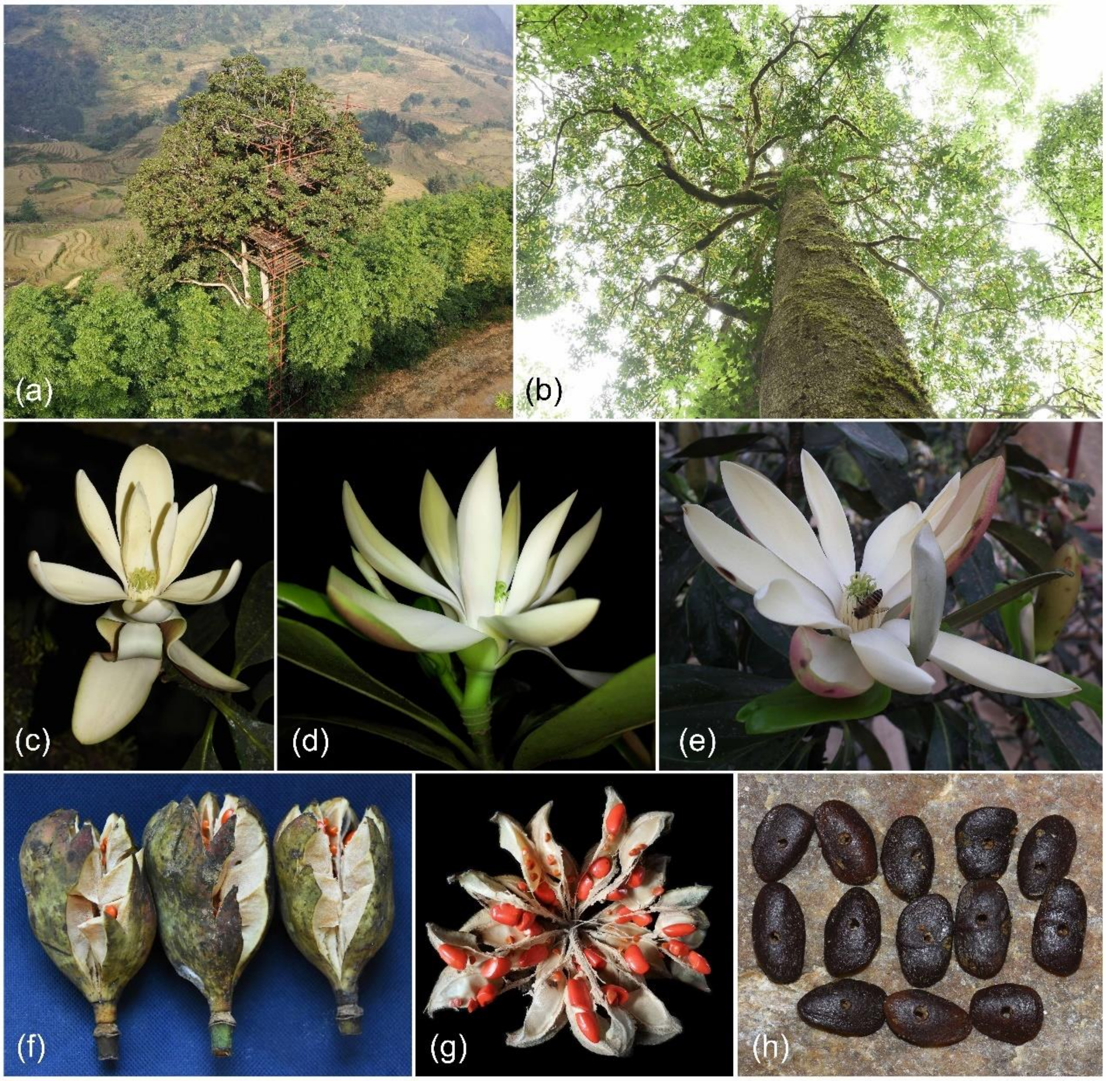

FIGURE 1 Habitat and morphological characters of *Magnolia sinica*. (a) Habitat. (b) Habit. (c-e) Flowers. (f) Fruits. (g) Fruit completely opened. (h) Seeds without testa.

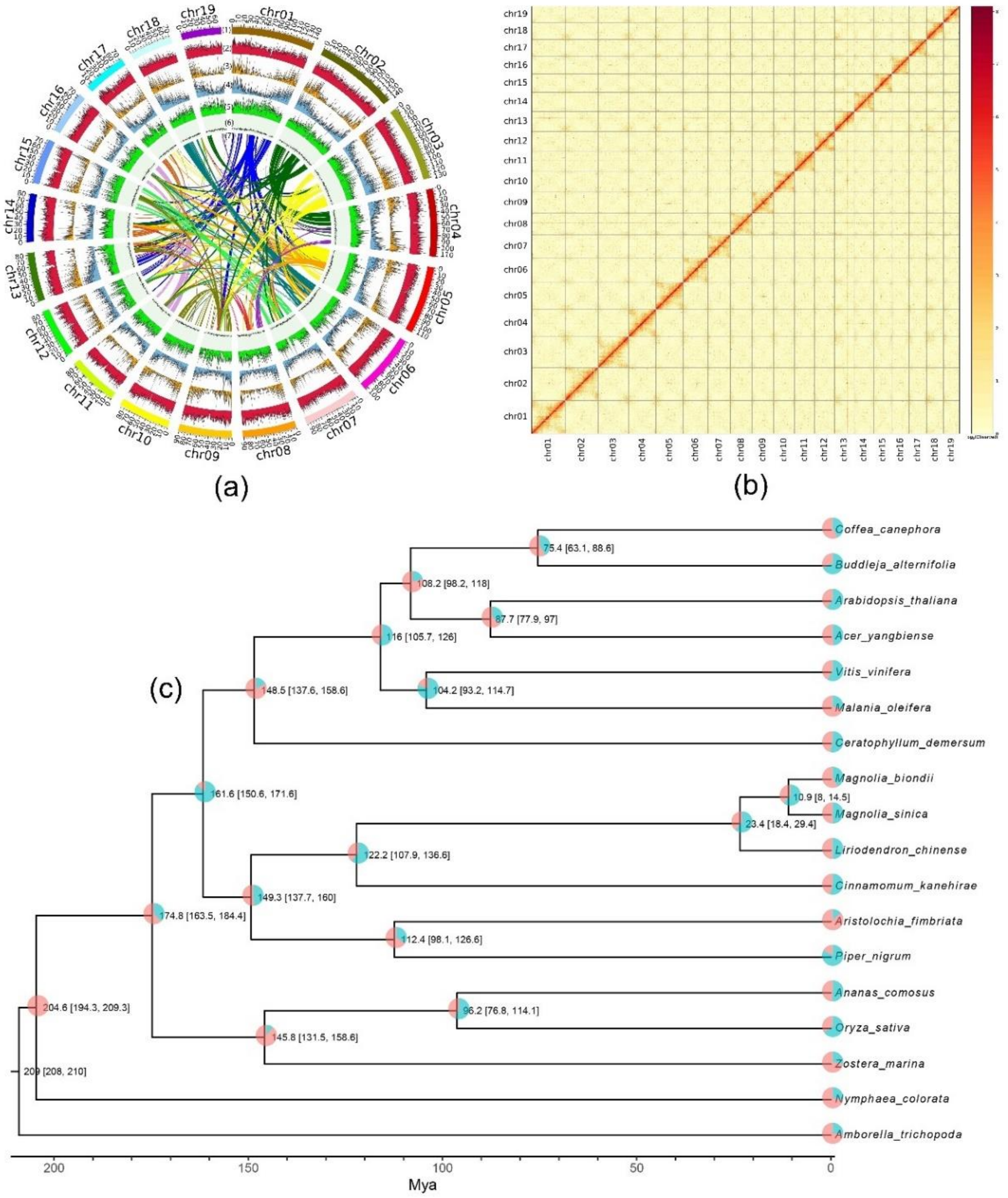

FIGURE 2 Genomic landscape of *Magnolia sinica* chromosomes, Hi-C heatmap and related phylogenetic tree. **(a)** The genome features across 19 chromosomes of *M. sinica*. (1) 19 pseudochromosomes. (2) Class I transposable element (TE) density (long terminal repeats, LTRs, long and short interspersed nuclear elements). (3) Class II TE (DNA and Heliron) density. (4) Coding gene (messenger RNA) density. (5) The density of single-nucleotide polymorphism (SNP) loci. (6) GC content. (7) collinear blocks. **(b)** Hi-C interaction heatmap for the *M. sinica* genome showing interactions among 19 chromosomes. **(c)** The phylogenetic tree of 18 species showing the

proportions of the gene families that contracted and expanded (pink: contracted; blue-green: expanded; Values at the nodes represent the time of differentiation and 95 % CI).

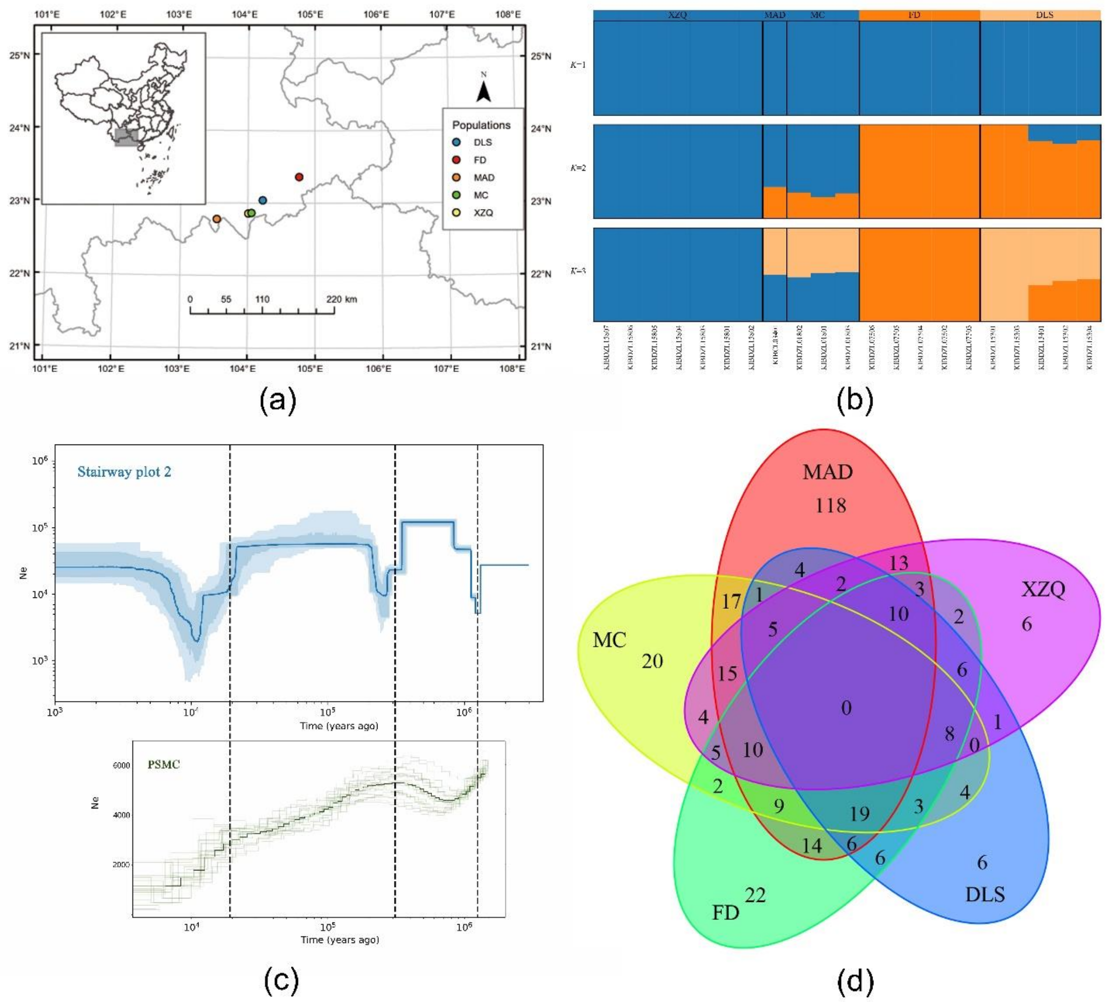

FIGURE 3 (a) Distribution map showing the locations of the five subpopulations in Yunnan. **(b)** Plots of the population structure of 21 *Magnolia sinica* individuals from five provenances for different numbers of subpopulations (K), from K = 1 to K = 3. **(c)** The demographic history of *M. sinica* inferred in Stairway plot2 (with a generation time of 30 years, and a mutation rate of 1.2e-7. The 95% confidence interval for the estimated effective population size is shown in a light blue color) and PSMC plot (with 21 samples of *M. sinica*, with the blue line being the average effective population size). **(d)** Venn diagram showing distribution of shared and unique deleterious mutations among the five subpopulations of *M. sinica*.

MAD, Maandi population in Jinping County; FD, Fadou population in Xichou County; XZQ, Xinzhaiqing population in Maguan County; DLS, Dalishu population in Maguan County; MC, Miechang population in Maguan County.

Table 1 Statistics of *Magnolia sinica* genome assembly and annotation

**Parameter**	***Magnolia sinica***
Total assembly size (bp)	1,839,595,854
GC content (%)	40.18
Total number of contings	203
Maximum conting length (bp)	96,921,630
Minimum conting length (bp)	5,003
Conting N50 (bp)	44,871,976
Conting N90 (bp)	10,133,504
Total number of scaffolds	130
Maximum scaffold length (bp)	141,926,363
Minimum scaffold length (bp)	5,003
Scaffold N50 (bp)	92,164,922
Scaffold N90 (bp)	73,752,208
Gap number	73
Complete BUSCOs (%)	90.5
Complete single-copy BUSCOs (%)	86.7
Complete and duplicated BUSCOs (%)	3.8
Fragmented BUSCOs (%)	2.8
Missing BUSCOs (%)	6.7
Gene number	44,713
Protein-coding genes	43,473
LAI value	10.3

Click here to access/download;Table;Table 1.docx

Table 1 Statistics of *Magnolia sinica* genome assembly and annotation

**Parameter**	***Magnolia sinica***
Total assembly size (bp)	1,839,595,854
GC content (%)	40.18
Total number of contings	203
Maximum conting length (bp)	96,921,630
Minimum conting length (bp)	5,003
Conting N50 (bp)	44,871,976
Conting N90 (bp)	10,133,504
Total number of scaffolds	130
Maximum scaffold length (bp)	141,926,363
Minimum scaffold length (bp)	5,003
Scaffold N50 (bp)	92,164,922
Scaffold N90 (bp)	73,752,208
Gap number	73
Complete BUSCOs (%)	90.5
Complete single-copy BUSCOs (%)	86.7
Complete and duplicated BUSCOs (%)	3.8
Fragmented BUSCOs (%)	2.8
Missing BUSCOs (%)	6.7
Gene number	44,713
Protein-coding genes	43,473
LAI value	10.3

Figure 1

Click here to access/download;Figure;FIGURE 1.pdf

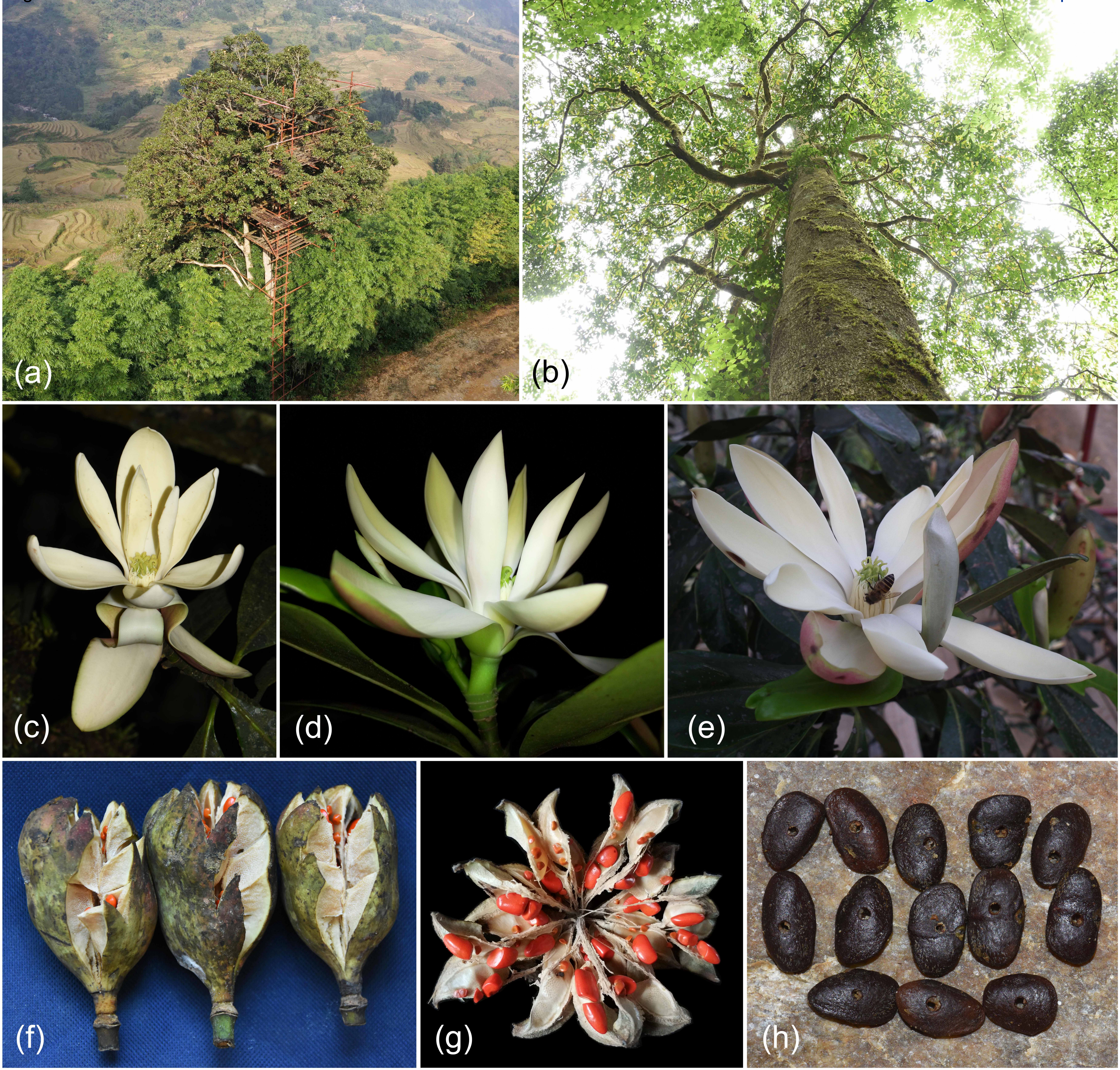

Figure 2

Click here to access/download;Figure;FIGURE 2.pdf

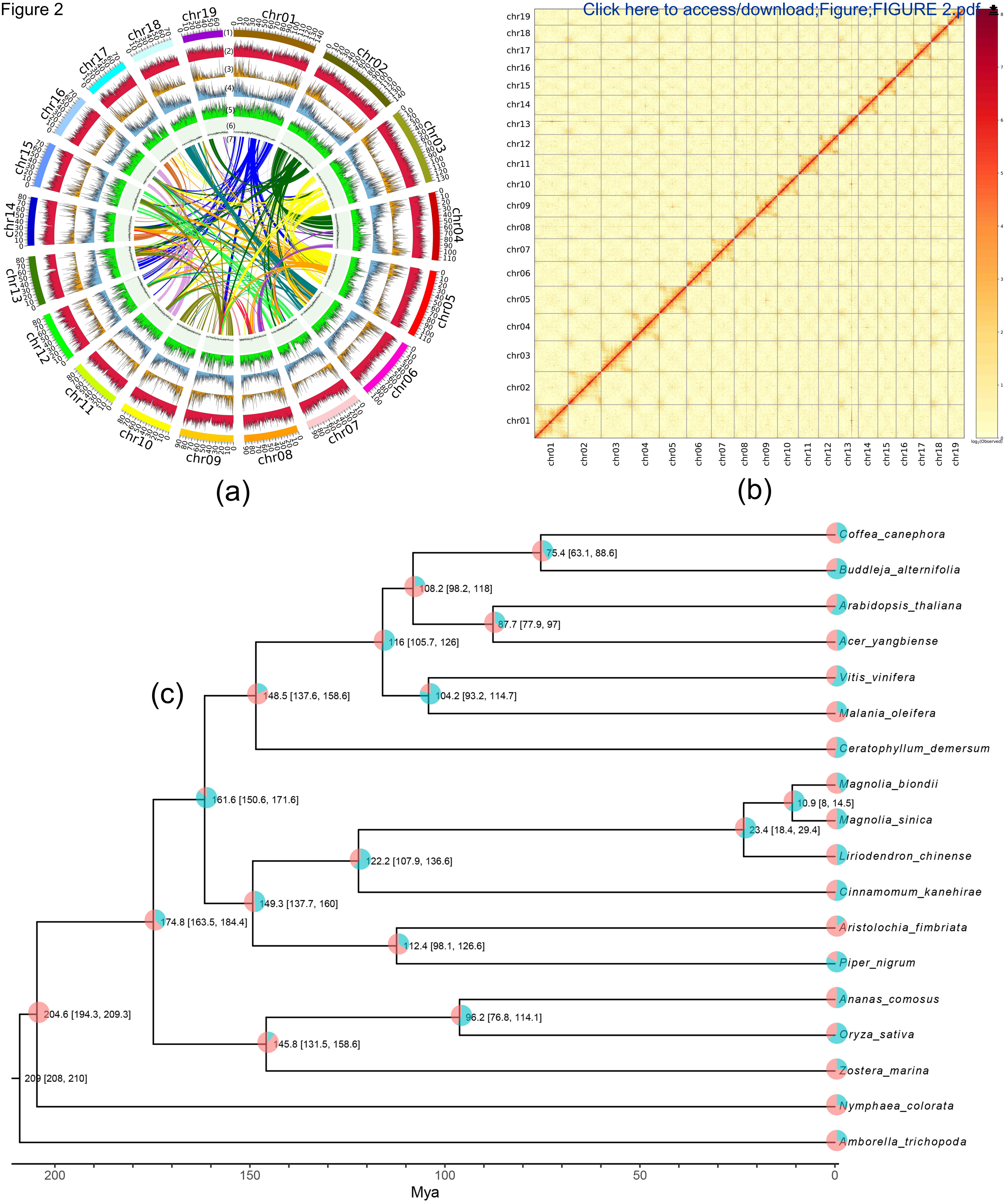

Figure 3

Click here to access/download;Figure;FIGURE 3.pdf

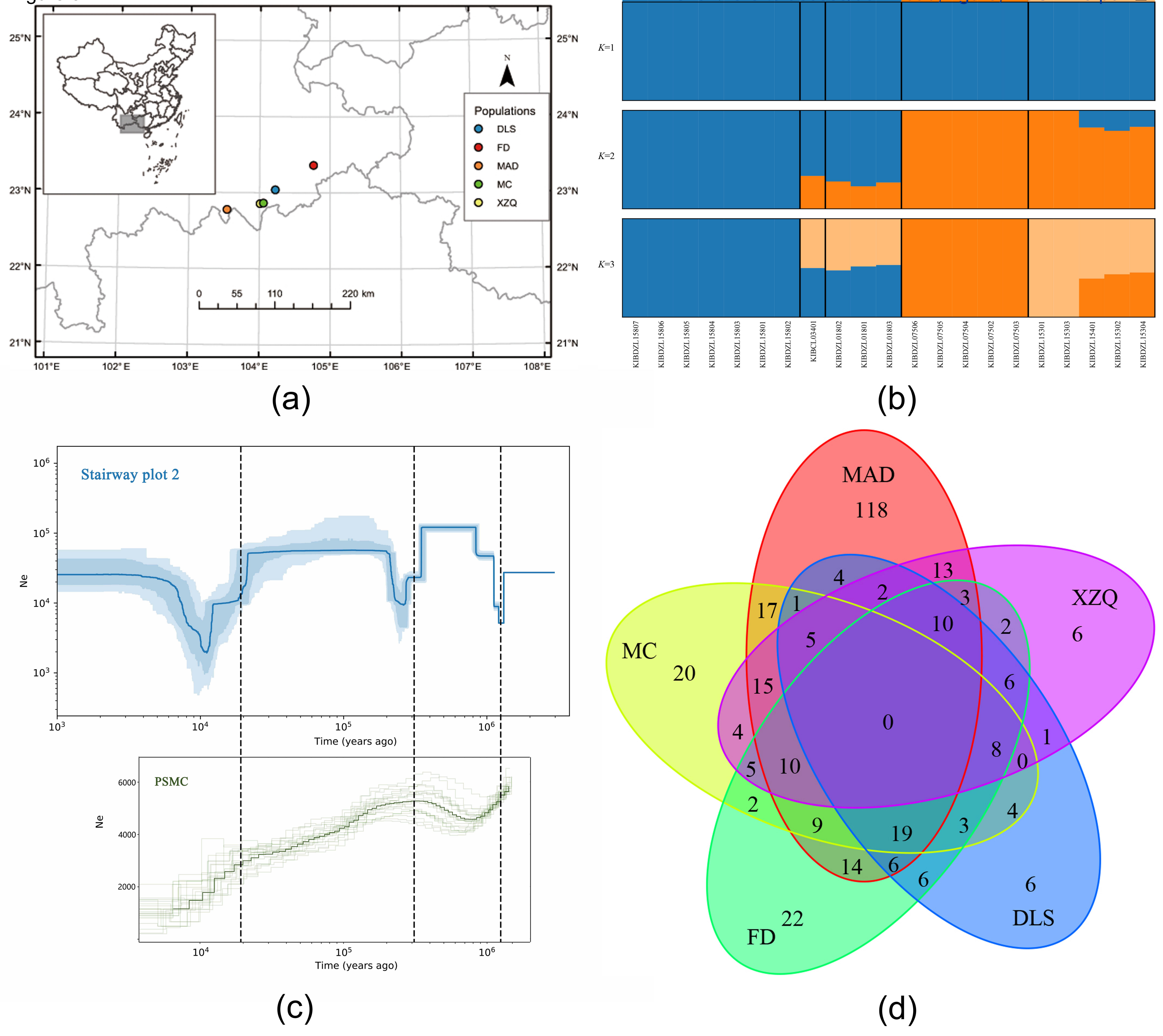

Supplementary file-Tables

Click here to access/download
Supplementary Material
Supplementary file-Tables.xlsx

Click here to access/download
Supplementary Material
Supplementary file-Figures 20230306.pdf
