# Supplementary material for: The chromosome-scale genome of Magnolia sinica (Magnoliaceae) provides insights into the conservation of plant species with extremely small populations (PSESP)
Source: Gigascience. 2024 Jan 11;13:giad110. doi: 10.1093/gigascience/giad110 (PMC10999834; doi:10.1093/gigascience/giad110)
Supplement: giad110_GIGA-D-23-00060_Revision_1 [file giad110_giga-d-23-00060_revision_1.pdf]

GigaScience

The chromosome-scale genome of Magnolia sinica (Magnoliaceae) provides insights into the conservation of plant species with extremely small populations (PSESP)

--Manuscript Draft--

Manuscript Number:	GIGA-D-23-00060R1
Full Title:	The chromosome-scale genome of Magnolia sinica (Magnoliaceae) provides insights into the conservation of plant species with extremely small populations (PSESP)
Article Type:	Research
Funding Information:	National Science & Technology Basic Resources Investigation Program of China (2017FY100100) — Prof. Weibang Sun<br>Yunnan Fundamental Research Projects (202101AT070173) — Dr. Lei Cai<br>National Natural Science Foundation of China (NSFC) (32101407) — Dr. Lei Cai<br>National Natural Science Foundation of China (NSFC) – Yunnan Joint Fund (U1302262) — Prof. Weibang Sun
Abstract:	Magnolia sinica (Magnoliaceae) is a highly threatened tree endemic to Southeast Yunnan, China. In this study, we generated for the first time a high-quality chromosome-scale genome sequence from M. sinica, by combining Illumina and ONT data with Hi-C scaffolding methods. The final assembled genome size of M. sinica was 1.84 Gb, with a contig N50 of ca. 45 Mb and scaffold N50 of 92 Mb. Identified repeats constituted approximately 57% of the genome, and 43,473 protein-coding genes were predicted. Phylogenetic analysis show that the magnolias form a sister clade with the eudicots and the order Ceratophyllales, while the monocots are sister to the other core angiosperms. In our study, a total of 21 individuals from the five remnant populations of M. sinica, as well as 22 specimens belonging to eight related Magnoliaceae species, were resequenced. The results showed that M. sinica had higher genetic diversity ($\theta w = 0.01126$ and $\theta \pi = 0.01158$) than other related species in the Magnoliaceae. However, population structure analysis suggested that the genetic differentiation among the five M. sinica populations was very low. Analyses of the demographic history of the species using different models consistently revealed that two bottleneck events occurred. The contemporary effective population size of M. sinica was estimated to be 10.9. The different patterns of genetic loads (inbreeding and numbers of deleterious mutations) suggested constructive strategies for the conservation of these five different populations of M. sinica. Overall, this high-quality genome will be a valuable genomic resource for conservation of M. sinica.
Corresponding Author:	Yongpeng Ma<br><br>CHINA
Corresponding Author Secondary Information:	
Corresponding Author's Institution:	
Corresponding Author's Secondary Institution:	
First Author:	Lei Cai, Ph.D.
First Author Secondary Information:	
Order of Authors:	Lei Cai, Ph.D.<br>Detuan Liu<br>Fengmao Yang<br>Rengang Zhang

Powered by Editorial Manager® and ProduXion Manager® from Aries Systems Corporation

	Quanzheng Yun
	Zhiling Dao, PhD
	Yongpeng Ma
	Weibang Sun, PhD
**Order of Authors Secondary Information:**	
**Response to Reviewers:**	Comments to the editor and reviewers<br>Dear the Editor of GigaScience,<br>Thank you very much for editing this manuscript entitled “The chromosome-scale genome of Magnolia sinica (Magnoliaceae) provides insights into the conservation of plant species with extremely small populations (PSESP)” and making suggestions. We are also very grateful for the efforts of the two reviewers. We have revised the manuscript carefully according to their comments and have made responses listed below.<br>We have accepted most of the comments from the two reviewers, made revisions to the errors that occurred, added some relevant analyses, and have responded to and explained a small portion of the questions. 1) We have added discussions of the coexistence of high genetic diversity and low genetic differentiation to the manuscript in the DISCUSSION part. 2) We have added relevant supplementary figures with bootstrap values in the phylogenetic tree (Figure S5). 3) We have added parameters and we have added KAT analysis. 4) We have released all the data produced to date (https://www.ncbi.nlm.nih.gov/bioproject/PRJNA774088). 5) We have explained why the whole genome sequencing and transcriptome sequencing (RNA-seq) analyses did not use material from the same individual, and also explained why only 21 individuals were re sequenced. Please review the specific revisions and responses.<br>We resubmit the revised manuscript and we hope this version is now suitable for the publication in GigaScience. If you have any further questions or requirements, please do not hesitate to contact the corresponding author (MYP).<br>Yours sincerely,<br>Yongpeng Ma (corresponding authors on behalf of all authors).<br>26th JULY 2023<br><br>Reviewer #1: In this paper, authors reported the first genome of a critically endangered species Magnolia sinica. This large tree is widely known as "giant pandas in plants" due to its extremely rare individuals in wild, thus is under the first-class state protection in China. Here, authors obtained a high-quality chromosome-level genome assembly via combining Illumina, PacBio and Hi-C sequencing data.<br>Authors mainly focus on the population resequencing, showing a high genetic diversity of M. sinica population but a low genetic differentiation among subpopulations. Authors provide some explanations for each result. I wonder if author can discuss the potential connections between these two observed phenomenons. In addition, authors detected many deleterious mutations which were mostly related to lipids. Authors didn't mention this result in the DISCUSSION part. Are these deleterious mutations related to lipids results of or reasons for the endangered status of this species? Authors may provide further discussions or even conclusive evidences to clearly elucidate point of view this issue.<br>Response: Thank you for your suggestion. We now added discussions of coexistence of high genetic diversity and low genetic differentiation to the manuscript in the DISCUSSION part as below:<br>“M. sinica has a pollinator-dependent outcrossing mating system, which may contribute to its high genetic diversity; while high gene flow among populations may maintain links between populations of this species, and may contribute to its low genetic differentiation. The recent reduction in population size due to anthropogenic activities has led to isolation of the populations, leading to the high genetic diversity and low genetic differentiation now observed in the fragmented populations of this endangered tree species. Similar patterns have been reported in Michelia coriacea, another species in the Magnoliaceae [131].”<br>Regarding the deleterious mutations related to lipids, we could not conclude whether they were the results of or the reasons for the endangered status of Magnolia sinica, and we have therefore deleted the parts of the GO and KEGG anotations and enrichment analysis regarding deleterious mutations from the manuscript.

Powered by Editorial Manager® and ProduXion Manager® from Aries Systems Corporation

Reference

Zhao X, Ma Y, Sun W, et al. (2012) High genetic diversity and low differentiation of Michelia coriacea (Magnoliaceae), a critically endangered endemic in southeast Yunnan, China. International Journal of Molecular Sciences, 13(4): 4396–4411.

Minor concerns:

1. Introduction part: authors should point out what's the major limitations of the current protection of Huagaimu. And how a reference genome helps to overcome such limitations.

Response: Thank you. We have added the first part in the manuscript. And, the second part was included in last paragraph of the introduction as below.

“Although a great deal of protection and research action has been carried out, the lack of natural regeneration and genetic rescue still limits the protection of M. sinica. Therefore, the formulation of genetic rescue strategies for M. sinica will benefit greatly from the exploration of harmful cumulative mutations, population historical dynamics and effective population size from the whole genome level.

Here, we report a high-quality chromosome-scale genome sequence of Magnolia sinica, and compare it with other relevant published genomic data. By exploring the evolution of the genome, as well as the genetic characteristics, demographic history and genetic load of M. sinica, we have identified genomic factors that may contribute to the threats to this species, and, on the basis of this, we propose further strategies for the conservation of M. sinica.”

2. Magnolia sinica was first occurred in Line 79 in the main text and it should be written as M. sinica afterwards.

Response: Thank you. We have checked and revised this.

3. Line 206: "integrated annotated protein" should be "integrated annotated proteins".

Response: Thank you. We have revised this.

4. Line 222-224: References were needed here.

Response: Thank you. We have added relevant references.

5. Line 253: "θW" should be "θw".

Response: Thank you. We have revised this.

6. Fig. 2c, there shouldn't be a "_" within species name. And, bootstrap values should be indicated in the phylogenetic tree. In addition, Fig. 2 contained different results with no obvious connections. I do recommend to layout the content of this figure, focusing on one particular theme.

Response: Thank you. We now deleted the "_" within species name. We have added a relevant supplementary figure with the bootstrap values in the phylogenetic tree, please check (Figure S5). Because of the large number of figures in the manuscript, we have tried to save space and have given the figures (genomic character and genome evolution), where related figures are merged into one plate and explanations are provided separately.

7. No title was found in Fig. 3. Authors should give a strong title that reflects the major finding of this figure.

Response: Thank you. We have added a title (Distribution map, population structure, demographic history and Venn diagram of Magnolia sinica) for this Figure 3.

Reviewer #2: This manuscript described the assembly and analyses of the chromosome-scale genome assembly for Magnolia sinica, an endangered Magnoliaceae species. Despite the authors provided a useful piece of work, it can still be greatly improved. In particular, it needs a thorough proofing to clarify many points in the Material & Methods section, as well as in results.

However, a major interrogation is the rational of resequencing only 21 M. sinica and 22 other Magnolia, while there is only 52 remaining M. sinica in the wild. I think it would have shown a much complete picture to generate data for all (known) individuals in the species.

Response: Thank you for your questions. In 2019, we only re-sequenced the materials that we had collected (21 samples). These materials included samples from all populations and covered the full range of the Magnolia sinica distribution, representing >40% of all M. sinica individuals. Because the collection of these materials took a lot of money and time, considering the cost of re-collection and the expensive re-sequencing costs at the time, we were unable to collect material from more individuals. Furthermore, based on the preliminary analysis of our sequencing data, we found that there were no significant differences (such as genetic diversity or genetic structure) compared to previous population studies based on SSR (Chen 2017, in Chinese).

Therefore, we only sequenced 21 individuals of M. sinica from that time.
The phylogenetic position of M. sinica has always been controversial, so we chose to sequence 22 samples from other eight Magnolia species. We have provided the relevant chloroplast tree (attached figure 1 chloroplast tree) and SNPs tree (attached figure 2 SNP_tree) as attachments at the bottom of this file.

I noticed several mistakes in the description of used data and methods. For example:
(1) line 21 the authors mentioned using Pacbio data for genome assembly, but from the Material & Methods, they used only ONT data to generate long reads for assembly
Response: We have revised this mistake.
(2) they mentioned a QiaGen kit that seems to not exist in Material & Methods line 149 they mentioned using Pilon to modifiy - correct? - Illumina reads; should be the opposite
Response: The reagent kit with product number 13323, Qiagen, is available. Genomic DNA kit (cat. no. 13323. Qiagen, Hilden, Germany). Please check: https://www.qiagen.com/us/products/discovery-and-translational-research/dna-rna-purification/dna-purification/genomic-dna/blood-and-cell-culture-dna-kits.
We have corrected the description of correcting with Illumina reads.
(3) Parameters used for pipelines are missing in several part of the manuscript
Also, the usually used metrics and quality assessment methods were not used here; I would appreciate to get a Merqury / KAT/ GenomeScope analysis in addition to the BUSCO and LAI.
Response: We have added parameters and a KAT analysis.

Also, I don't really understand why the authors performed RNAseq for annotation from a different individual, instead of using the same individual as for the genome assembly.
Response: Thank you. We understand your concern regarding this issue, unfortunately we faced some challenges during this project. In 2019, when we started sequencing, leaf samples were initially sent to a company in dry ice for genome sequencing. Later in 2020, when we collected multiple tissues for RNA-seq, it became very difficult to send samples rapidly in dry ice because of special policies (special periods of COVID-19). Therefore, for simplicity, we decided to directly send a living seedling (including leaf, stem, root tissues, but excluding other tissues such as flowers) and fresh fruits at room temperature (without dry ice) for RNA-seq. Therefore, the RNAseq and genome assembly analyses were conducted using different individuals. However, because we used the PacBio platform to sequence the full-length cDNA, the variations between individuals should have very limited negative effects on gene annotation. In fact, 99.5% PacBio CCS reads were mapped to the genome.
The ancestral sequence reconstruction part appeared quite weak with the method used, not taking into account the emergence of potentially large Structural Variations (SVs) across the chromosomes during their evolutions. I would suggest, if the authors want to keep this part to use a more robust approach (e.g. based on Salse, 2021 approach)
Response: Thank you for your suggestion. We agree that the emergence of SV may influence the reconstruction of ancestral state. However, SV is difficult to detect from our short reseqencing reads. Here we used an empirical Bayesian method based on posterior probability of the sites to reconstruct ancestral sequence. This method can produce accurate reconstruction of the ancestral sequence (Hanson-Smith et al. 2010) and has been previously used to reconstruct the ancestral state in other works (Cristofari et al., 2016; Salojärvi et al., 2017; Ma et al., 2021; Fukushima et al., 2023). We apologize for not being able to find the article by “Salse, 2021”. After explaining our method above, if it is necessary to use Salse's approach, could you please provide us more information about it and give us another chance to revise it?
References
Cristofari R, Bertorelle G, Ancel A, et al. Full circumpolar migration ensures evolutionary unity in the Emperor penguin. Nat Commun. 2016;7:11842. doi: org/10.1038/ncomms11842.
Fukushima K, Pollock DD. Detecting macroevolutionary genotype–phenotype associations using error-corrected rates of protein convergence. Nat Ecol Evol. 2023;7: 155–170. doi: org/10.1038/s41559-022-01932-7.
Hanson-Smith V, Kolaczkowski B, Thornton JW. Robustness of Ancestral Sequence Reconstruction to Phylogenetic Uncertainty. Mol Biol Evol. 2010;27 (9):1988–1999. Doi: org/10.1093/molbev/msq081.

	Ma H, Liu YB, Liu DT, et al. Chromosome-level genome assembly and population genetic analysis of a critically endangered rhododendron provide insights into its conservation. Plant J. 2021;107(5):1533–45. doi: 10.1111/tpj.15399.<br>Salojärvi J, Smolander OP, Nieminen K. et al. Genome sequencing and population genomic analyses provide insights into the adaptive landscape of silver birch. Nat Genet. 2017;49:904–912. doi: org/10.1038/ng.3862.<br>The data accessibility is also questionable, as the authors mentioned the BioProject PRJNA774088, that is already cited by a published paper, but not accessible<br>Response: We apologize that the data were not released earlier. The data have now been completely released (https://www.ncbi.nlm.nih.gov/bioproject/PRJNA774088). A copy of the data can be found in China National Center for Bioinformation (https://ngdc.cncb.ac.cn/bioproject/browse/PRJCA015437).<br><br>Specific comments:<br>-Line 21 : Only ONT data were combined with short reads to assemble the genome ;<br>Response: Sorry, we have revised this mistake.<br>-Line 59 : please add the date when the database have been accessed ;<br>Response: Thank you. We have corrected this and added the access dates.<br>-Line 93-97 : this seems more adequate for a Data Notes than for a research article ;<br>Response: Thank you, this is indeed only a partial summary. Here, we not only reported the high-quality chromosome-scale genome sequence of Magnolia sinica and re-sequenced 21 samples of the same species and 22 samples from other species, but also investigated genome evolution, genome-wide diversity, and population structure of this species, inferred its demographic history, and estimated its genetic load and inbreeding level. We further discussed the possible reason for its high genetic diversity but low genetic differentiation, the climatic, tectonic and anthropogenic explanation of its demographic history, the likely genetic basis of the extremely small populations, and provided conservation measures based on our findings. We think it is worthy of a research article.<br>-Line 107 : dry ice temperature is -78.5°C<br>Response: We have revised this mistake.<br>-Line 118 : this kit does not exist (the reference number is for an other kit)<br>Response: We have revised this. The Genomic DNA kit (cat. no. 13323. Qiagen, Hilden, Germany) is available, and this kit can also extract genomic DNA from diverse materials. The kit was also used to extract plant DNA after treatment of CTAB.<br>-Line 121 : more details are needed for the library construction method. What was the DNA input ? any modification from the ONT protocol ? barcoded library or not ?<br>Response: The DNA input was total genomic DNA. The ONT protocol was not modified, and the library was not barcoded.<br>-Line 124 : please choose the machine the library was run on (or precise which library was run on which machine) ; how many flowcells ?<br>Response: PromethION was used yielding 7 flowcells. This has been added to the manuscript.<br>-Line 126 : what fragment size for the Illumina library<br>Response: We have added insertion size of 300–500 bp.<br>-Line 130 : what was considered as "high molecular weight DNA" ?<br>Response: This refers to longer and more complete DNA with high “molecular weight”.<br>-Line 147: please precise what assembly strategies did you used (= assemblers ?)<br>Response: Thank you, we have added a descriptions of the assembly method.<br>-Line 148 : this reference is for the Celera assembler only, did you use it ?<br>Response: No. We have revised the text.<br>-Line 149 : short reads were used to correct long reads, not the opposite ;<br>Response: Thank you, this has been revised.<br>-Line 151 : how they were polished ?<br>Response: The method has been added.<br>-Line 151 : please described the parameters used in GetOrganelles to assemble both the mitochondrial genome and plastome<br>Response: The parameters have been added.<br>-Line 159 : "scaffolded" instead of "scattered" ?<br>Response: This has been revised as “un-anchored” meaning contigs that were not anchored onto chromosomes.<br>-Line 161 : what parameters for LR_Gapcloser and NextPolish ?<br>Response: The parameters have been added.<br>-Line 163 : Redundant (typo)

Powered by Editorial Manager® and ProduXion Manager® from Aries Systems Corporation

Response: It has been revised.
-Line 165 : what is the NT library ?
Response: The NT library is NT database from NCBI for BLAST (https://ftp.ncbi.nlm.nih.gov/blast/db/). We have revised this in the text for clarification.
-Line 167 : how low was a coverage considered ?
Response: We have revised this in the text.
-Line 172-183 : see above for addition of QC pipelines results
Response: We have added KAT analysis.
-Line 189 : how these two libraries were combined ?
Response: We concatenated the two libraries (fasta files) directly using the Linux command `cat`.
-Line 194 : Considering Magnoliaceae position in angiosperms, I think it could be useful to add at least one monocots in the annotation process (e.g. the wheat or maize, or rice genome)
Response: Thank you for your suggestion. We tested this by adding the wheat genome, and found only 551 new genes (1.3% more than before) predicted by the MAKER2 pipeline. We also tested it with the Aristolochia fimbriata (Piperales) genome as evidence, and 1419 genes (3.3% more) were newly identified. It appears that more protein evidences would certainly produce more genes, but considering the improvements (1.3-3.3% more genes) are quite limited and would not significantly affect our downstream conclusions regarding comparative and conservation genomics, we chose to not include the update in the revision.
-Line 201 : Augustus is usually used as an ab initio annotator ; please specify more in details how you used it the integrate previous annotations
Response: Yes, Augustus is an ab initio annotator, but it supports biological evidence (hint file from transcript and protein alignments) as input for better prediction. This step is integrated in the MAKER2 pipeline. We have revised the text for a clearer description.
-Line 217, 220, 222 : why there is a discrepancy between the single-copy gene numbers ?
Response: We used different cutoffs to allow for missing data. For the ASTRAL method, more genes are better with high ILS (incomplete lineage sorting) level, and missing data are more tolerated (References below), so we used more genes with higher missing rate (30%). For the IQTREE method, missing data are moderately tolerated, so we used the dataset with moderate missing rate (12.5%; the dataset was generated in OrthoFinder2 to infer a species tree in its pipeline). MCMCtree uses only non-missing data by default, so we just included 1:1 orthologous single-copy genes (with none missing). Different dataset may provide cross-validations to reduce sampling bias. We have added detailed descriptions.
References:
Molloy E K, Warnow T. To Include or Not to Include: The Impact of Gene Filtering on Species Tree Estimation Methods [J]. Syst. Biol., 2017, 67 (2): 285–303 [http://doi.org/10.1093/sysbio/syx077]
Shekhar S, Roch S, Mirarab S. Species Tree Estimation Using ASTRAL: How Many Genes Are Enough? [J]. IEEE/ACM Transactions on Computational Biology and Bioinformatics, 2018, 15 (5): 1738–1747 [http://doi.org/10.1109/TCBB.2017.2757930]

-Line 235 : Why not using the 52 M. sinica individuals (see above) ?
Response: Thank you for your questions. In 2019, we only re-sequenced the materials that we had collected (21 samples). These materials included samples from all populations, and covered the full range of the Magnolia sinica distribution, representing >40% of all M. sinica individuals. Because the collection of these materials took a lot of money and time, considering the cost of re-collection and the expensive re-sequencing costs at the time, we were unable to collect material from more individuals. Furthermore, based on the preliminary analysis of our sequencing data, we found that there were no significant differences (such as genetic diversity or genetic structure) compared to previous population studies based on SSR (Chen 2017, in Chinese). Therefore, we only sequenced 21 individuals of M. sinica from that time.
-Line 241 : sequences with quality score <20 should not be found in the clean reads (from line 238)
Response: After filtering with fastp, the proportion of sequences with a quality score <20 decreases, however, there are still some bases with a quality score <20. Fastp trims reads using a sliding window, but did not trim all bases with a quality score <20. Thus, we excluded the potentially retained bases with quality score <20 in downstream

Powered by Editorial Manager® and ProduXion Manager® from Aries Systems Corporation

analysis (ANGSD and freebayes).
-Line 242 : considering a sequencing depth ranging from 8.8X to 12.6X for M. sinica (max 14.3X for other Magnolia), it seems unrealistic to remove sites with a mapping depth <100X
Response: The depth of sites refers to the sum of all samples, but not average depth across samples. The distribution of the depth of sites is as follows. The peak value is at 331x, so empirically the upper limit is set to 600x, about twice that of the peak, and the lower limit is about 1/3 of the peak. We have revised the text to make this clear.

-Line 243 : please specify how these sites were retained
Response: We have described this in more detail in the paper.
-Line 248 : why the authors did not use the widely used 10% missing data threshold?
Response: Thank you for your question. We wanted to balance the threshold and the number of SNPs. Considering that there are many species, a stricter threshold would lead to fewer SNPs, which may be not have been sufficient for downstream analyses. In fact, the threshold of 20% or higher has also been used in previous studies (References below).
References:
Liu S, Zhang L, Sang Y et. al. Demographic History and Natural Selection Shape Patterns of Deleterious Mutation Load and Barriers to Introgression across Populus Genome [J]. Mol. Biol. Evol., 2022, 39 (2) [http://doi.org/10.1093/molbev/msac008]
Dai F, Zhuo X, Luo G et. al. Genomic Resequencing Unravels the Genetic Basis of Domestication, Expansion, and Trait Improvement in Morus Atropurpurea [J]. Adv. Sci., 2023 [http://doi.org/10.1002/advs.202300039]
Wang P, Zhou G, Jian J et. al. Whole-genome assembly and resequencing reveal genomic imprint and key genes of rapid domestication in narrow-leafed lupin [J]. Plant J., 2021, 105 (5): 1192–1210 [http://doi.org/10.1111/tpj.15100]
Ma Z, Zhang Y, Wu L et. al. High-quality genome assembly and resequencing of modern cotton cultivars provide resources for crop improvement [J]. Nat. Genet., 2021 [http://doi.org/10.1038/s41588-021-00910-2]
-Line 249 : due to both the relatively low number of indiviuals and the large part of the sampling made of other Magnolia species, such a classic MAF value would results in removing SNPs present in 1 or 2 samples, making them potentially diagnostic of a given species
Response: We did not aim to make diagnostic of a given species, so the species-specific SNPs were not necessary for our analyses. In the phylogenetic tree based on the filtered SNPs (attached figure 2 SNP_tree), each species has formed a separate monophyletic clade, suggesting that our filtering with the classic MAF value did not obscure the relationships among these species.
-Line 250 and following : Please described more in details, but concisely, how these different datasets are made, and how they are each useful (at least more useful than only one or two datasets)
Response: We apologized for the imprecise and incorrect descriptions. We have revised this and have also added an additional schematic diagram to the supplementary figures to illustrate it.
-Line 309 : please add the parameters used
Response: Thank you, we have added these.
-Line 319 : did the authors considered flow cytometry to get a (more) accurate estimate of the genome size ? Considering the patrimonial value of the species, it could be valuable.
Response: Thank you. At that time, the Genome size of Magnolia sinica was estimated by k-mer analysis of the Illumina sequencing data. This method is widely used and is sufficiently accurate, so we felt that we did not need to use an experimental method based on Flow Cytometry.
-Line 327 : Did the authors compared the LAI value obtained here with other Magnolia genome assemblies ?
Response: Thank you. We could not compare the relevant LAI values of several Magnolia species because of the other three genomic articles did not calculate this value.
-Line 335-336 : Please add values for gene annotations from transcriptomic, ab initio and similarity approaches separately, then indicate how many were supported, filtered and so on, with the final value.
Response: The MAKER annotation pipeline used in the study does not generate individual gene annotations; instead, it only produced intermediate alignments of

Powered by Editorial Manager® and ProduXion Manager® from Aries Systems Corporation

evidence. Here we compared these intermediate alignments to the final gene set. Please refer to the attached table for details.

-Line 343 : what is "certain other databases of M. sinica" ?
Response: Thank you, we have revised this and added the annotated percentages from several different databases, and these can be found in Supplementary Table 19. “certain other databases, including Pfam (25,850, 59.46%), Coils (2,533, 5.83%), CDD (28,110, 64.70%), SMART (8,247, 18.97%) and others were annotated with IntrerProScan. (Table S19)”.
-Line 343 : InterProScan (typo)
Response: It has been revised.
-Line 344 : 90 % BUSCO value seems very low for a modern assembly. What could explain such a low value ?
Response: Thank you. This was because previously we used an old version of BUSCO (v2). In the revision, we have used the last version BUSCO5 and the value improved significantly (97.9%). We have revised this text.
-Line 357-361 : How is it different from (or similar with) the other studies ?
Response: We have discussed the relationship between our research results and those from other studies in the discussion section.
-Line 381 : what could explain the very low mapping rate (~90%) of M. sinica against itself (same species) ?
Response: They are the same species according to the SNP tree and the chloroplast tree, so the low mapping rate of this individuals could be attributed to sequencing artifacts.
-Line 391 : the end of the sentence does not make sense.
Response: Thank you, we have deleted this.
-Line 440- 445 : Are these values significant ?
Response: Yes, these terms were significant, and we revised the expressions.
-Line 447-448 : There is also M. obovata / M. hypoleuca
Response: Thank you, we have added these.
-Line 631 : Is this script available ?
Response: Thank you, it is available, we still have this script. If you would like it, you are welcome to apply to write to the provided communication email and you will receive it soon.
-Table 1. contigs (typo)
Response: Thank you, we have revised this.

attached figure 1 chloroplast_tree attached figure 2 SNP_tree

Reference

Cristofari R, Bertorelle G, Ancel A, et al. Full circumpolar migration ensures evolutionary unity in the Emperor penguin. Nat Commun. 2016;7:11842. doi: org/10.1038/ncomms11842.
Dai F, Zhuo X, Luo G et. al. Genomic Resequencing Unravels the Genetic Basis of Domestication, Expansion, and Trait Improvement in Morus atropurpurea [J]. Adv. Sci., 2023 [http://doi.org/10.1002/advs.202300039]
Fukushima K, Pollock DD. Detecting macroevolutionary genotype–phenotype associations using error-corrected rates of protein convergence [J]. Nat Ecol Evol. 2023;7: 155–170. doi: org/10.1038/s41559-022-01932-7.
Hanson-Smith V, Kolaczkowski B, Thornton JW. Robustness of Ancestral Sequence Reconstruction to Phylogenetic Uncertainty [J]. Mol Biol Evol. 2010;27 (9):1988–1999. Doi: org/10.1093/molbev/msq081.
Liu S, Zhang L, Sang Y et. al. Demographic History and Natural Selection Shape Patterns of Deleterious Mutation Load and Barriers to Introgression across Populus Genome [J]. Mol. Biol. Evol., 2022, 39 (2). [http://doi.org/10.1093/molbev/msac008]
Ma H, Liu YB, Liu DT, et al. Chromosome-level genome assembly and population genetic analysis of a critically endangered rhododendron provide insights into its conservation [J]. Plant J. 2021;107(5):1533–45. doi: 10.1111/tpj.15399.
Ma Z, Zhang Y, Wu L et. al. High-quality genome assembly and resequencing of modern cotton cultivars provide resources for crop improvement [J]. Nat. Genet., 2021 [http://doi.org/10.1038/s41588-021-00910-2]
Molloy E K, Warnow T. To Include or Not to Include: The Impact of Gene Filtering on Species Tree Estimation Methods [J]. Syst. Biol., 2017, 67 (2): 285–303 [http://doi.org/10.1093/sysbio/syx077]

Powered by Editorial Manager® and ProduXion Manager® from Aries Systems Corporation

	Salojärvi J, Smolander OP, Nieminen K. et al. Genome sequencing and population genomic analyses provide insights into the adaptive landscape of silver birch [J]. Nat Genet. 2017;49:904–912. doi: org/10.1038/ng.3862.<br>Shekhar S, Roch S, Mirarab S. Species Tree Estimation Using ASTRAL: How Many Genes Are Enough? [J]. IEEE/ACM Transactions on Computational Biology and Bioinformatics, 2018, 15 (5): 1738–1747 [http://doi.org/10.1109/TCBB.2017.2757930]<br>Wang P, Zhou G, Jian J et. al. Whole-genome assembly and resequencing reveal genomic imprint and key genes of rapid domestication in narrow-leafed lupin [J]. Plant J., 2021, 105 (5): 1192–1210 [http://doi.org/10.1111/tpj.15100]<br>Zhao XF, Ma YP, Sun WB, et al. High genetic diversity and low differentiation of Michelia coriacea (Magnoliaceae), a critically endangered endemic in southeast Yunnan, China [J]. Int J Mol Sci. 2012;13(4):4396–411. doi:https://doi.org/10.3390/ijms13044396.
Additional Information:	
Question	Response
Are you submitting this manuscript to a special series or article collection?	No
**Experimental design and statistics**<br><br>Full details of the experimental design and statistical methods used should be given in the Methods section, as detailed in our Minimum Standards Reporting Checklist. Information essential to interpreting the data presented should be made available in the figure legends.<br><br>Have you included all the information requested in your manuscript?	Yes
**Resources**<br><br>A description of all resources used, including antibodies, cell lines, animals and software tools, with enough information to allow them to be uniquely identified, should be included in the Methods section. Authors are strongly encouraged to cite Research Resource Identifiers (RRIDs) for antibodies, model organisms and tools, where possible.<br><br>Have you included the information requested as detailed in our Minimum Standards Reporting Checklist?	Yes
**Availability of data and materials**	Yes

All datasets and code on which the conclusions of the paper rely must be either included in your submission or deposited in publicly available repositories (where available and ethically appropriate), referencing such data using a unique identifier in the references and in the "Availability of Data and Materials" section of your manuscript.<br><br>Have you have met the above requirement as detailed in our Minimum Standards Reporting Checklist?	

Manuscript

Click here to access/download;Manuscript;001 The whole genome of Magnolia sinica-final.docx

The chromosome-scale genome of *Magnolia sinica* (Magnoliaceae) provides insights into the conservation of plant species with extremely small populations (PSESP)

Lei Cai[1,†], Detuan Liu[1, 2,†], Fengmao Yang[1, 2], Rengang Zhang[1, 2], Quanzheng Yun[3], Zhiling Dao[1], Yongpeng Ma[1,*], Weibang Sun[1,*]

[1] Yunnan Key Laboratory for Integrative Conservation of Plant Species with Extremely Small Populations/ Key Laboratory for Plant Diversity and Biogeography of East Asia, Kunming Institute of Botany, Chinese Academy of Sciences, Kunming, Yunnan, China

[2] University of Chinese Academy of Sciences, 100049 Beijing, China

[3] Department of Bioinformatics, Ori (Shandong) Gene Science and Technology Co., Ltd., Weifang, Shandong, China

Correspondence address. Yongpeng Ma, E-mail: mayongpeng@mail.kib.ac.cn; http://orcid.org/0000-0002-7725-3677; Weibang Sun, E-mail: wbsun@mail.kib.ac.cn; https://orcid.org/0000-0002-7195-2215

[†]These authors contributed equally to this work.

Lei Cai [0000-0002-9251-2745];

Detuan Liu [0000-0002-2295-3799];

Fengmao Yang [0000-0001-8034-1398];

Rengang Zhang [0000-0002-8028-9208];

Zhiling Dao [0000-0002-9785-6336];

Yongpeng Ma [0000-0002-7725-3677];

Weibang Sun [0000-0002-7195-2215].

Abstract

Magnolia sinica (Magnoliaceae) is a highly threatened tree endemic to Southeast Yunnan, China. In this study, we generated for the first time a high-quality chromosome-scale genome sequence from *M. sinica*, by combining Illumina and ONT data with Hi-C scaffolding methods. The final assembled genome size of *M. sinica* was 1.84 Gb, with a contig N50 of ca. 45 Mb and scaffold N50 of 92 Mb. Identified repeats constituted approximately 57% of the genome, and 43,473 protein-coding genes were predicted. Phylogenetic analysis show that the magnolias form a sister clade with the eudicots and the order Ceratophyllales, while the monocots are sister to the other core angiosperms. In our study, a total of 21 individuals from the five remnant populations of *M. sinica*, as well as 22 specimens belonging to eight related Magnoliaceae species, were resequenced. The results showed that *M. sinica* had higher genetic diversity (θw = 0.01126 and $\theta\pi$ = 0.01158) than other related species in the Magnoliaceae. However, population structure analysis suggested that the genetic differentiation among the five *M. sinica* populations was very low. Analyses of the demographic history of the species using different models consistently revealed that two bottleneck events occurred. The contemporary effective population size of *M. sinica* was estimated to be 10.9. The different patterns of genetic loads (inbreeding and numbers of deleterious mutations) suggested constructive strategies for the conservation of these five different populations of *M. sinica*. Overall, this high-quality genome will be a valuable genomic resource for conservation of *M. sinica*.

Keywords: *Magnolia sinica*, PSESP, genome sequencing, deleterious mutation, population demographic, conservation

1 Introduction

The reduction of species diversity is of global concern, and has been closely linked with climate change and human activity. The conservation of biodiversity is therefore a hot topic [1–6]. The resolution of the recently convened CBD COP 15 (15th Conference of the Parties, Convention on Biological Diversity) supports biodiversity conservation issues of global concern, and one of the goals (so called "30 × 30") requires that at least 30% of the land, fresh water and oceans on Earth be protected in some form by 2030. In addition, identification of geographic areas with high concentrations of endemic and rare species diversity is an important step in protecting biodiversity [7]. The Mountains of Southwest China is one of the world's biodiversity hotspots, and is also affected by climate change and human disturbance, meaning that it is also an area at very high risk of species extinction [8, 9]. The study and protection of the threatened species in this region are therefore of particular importance and urgency [10, 11]. In order to rescue the most highly threatened species and reduce their risks of extinction in this region, Chinese scholars put forward the concept of Plant Species with Extremely Small Populations (PSESP) in 2005, according to China's current national conditions and the practice of biodiversity protection [12–15]. That a species is threatened by human activities and interference is a necessary qualifying condition to determine whether that species meets the definition of PSESP, and human activities are also of significance when implementing rescuing protection for PSESPs [12, 16].

Plant genome sequencing has grown rapidly in the past 20 years, and by the end of June 2023, the genomes sequences of more than 1000 higher plant taxa had been published [17]. Sequenced genomes can provide insights and evidence to better understand the genome biology and evolution of plants [18, 19]. Although the genomes of so many plant species have been studied, only a few

studies have sequenced the genomes of threatened plant species (examples include *Acer yangbiense*, *Acanthochlamys bracteata*, *Beta patula, Cercidiphyllum japonicum*, *Davidia involucrata*, *Dracaena cambodiana*, *Ginkgo biloba*, *Kingdonia uniflora*, *Malania oleifera*, *Ostrya rehderiana* and *Rhododendron griersonianum*) in order to focus on the conservation of these species [20–30].

Plant species in the family Magnoliaceae are hugely important in gardens and horticulture across the world [31, 32]. The Magnoliaceae is also one of the most highly threatened angiosperm groups. There are more than 300 species in this family, which are mainly distributed intermittently in the temperate, subtropical and tropical regions of East and Southeast Asia, East North America and central and South America [33–35]. About 120 species of Magnoliaceae are known from China, and Southwest and South China are the centers of diversity for this family [36]. Global conservation assessments suggest that 147 magnoliaceous species are facing threats, accounting for 48% of the total assessed species in this family [35]. Similarly, 76 species of Chinese Magnoliaceae are threatened, representing more than 50% of the total number of threatened Magnoliaceae species globally [37]. At present, in-depth genome research has only been conducted in four species in the Magnoliaceae (*Liriodendron chinense*, *Magnolia biondii*, *M. obovata* and *M. officinalis*), mainly to investigate the controversial evolutionary position of the magnoliids [38–41].

The evergreen tree *Magnolia sinica* (Law) Noot. (NCBI:txid86752) (Magnoliaceae) is a typical PSESP endemic to Southeast Yunnan, where many threatened species are in urgent need of rescue and protection [12, 14]. In China, the species is often referred to as *Manglietiastrum sinicum* Y.W. Law and is known as Huagaimu in Chinese [34, 36, 42, 43]. It has been categorized as Critically Endangered on the *China Species Red List* [44], *The Red List of Magnoliaceae* [35, 45] and *The Threatened Species List of China's Higher Plants* [37]. *M. sinica* was proposed as a first-rank plant

for national key protection in 1999 [46] and also in 2021 [47], and was listed as one of 62 PSESPs in Yunnan in 2010, and also as one of the 120 national PSESPs of China in 2012, requiring the most urgent rescue conservation [14, 15]. Recent survey data revealed only 52 individuals remaining in the wild, and comprehensive conservation research and protection action of *M. sinica* have been implemented, including reproductive and seed biology, genetic diversity studies based on SSR, sequencing of the chloroplast genome, investigation of the soil microbiome, *in situ* conservation, *ex situ* conservation and reintroduction programs [48–53]. Although great deal of protection and research action has been carried out, the lack of natural regeneration and genetic rescue still limits the protection of *M. sinica*. Therefore, the formulation of genetic rescue strategies for *M. sinica* will benefit greatly from the exploration of harmful cumulative mutations, population historical dynamics and effective population size from the whole genome level.

Here, we report a high-quality chromosome-scale genome sequence of *Magnolia sinica*, and compare it with other relevant published genomic data. By exploring the evolution of the genome, as well as the genetic characteristics, demographic history and genetic load of *M. sinica*, we have identified genomic factors that may contribute to the threats to this species, and, on the basis of this, we propose further strategies for the conservation of *M. sinica*.

2 Materials and methods

2.1 Collection of plant material

Magnolia sinica is only found scattered in several counties in southeast Yunnan (Figures 1 & 3a). Fresh young leaf material was collected for whole-genome sequencing from a single individual. This individual is conserved and growing *ex situ* at the Kunming Botanical Garden (KBG), but was originally introduced from Xichou County, Southeast Yunnan. For transcriptome sequencing, leaf,

stem and root samples were obtained from a three-year-old seedling also at KBG, and fresh fruits were collected from the wild in Jinping County, Yunnan. Fresh leaves used for genome library preparation, and other tissues used for transcriptome sequencing, were immediately frozen in liquid nitrogen and were stored at -78.5 °C in dry ice until DNA or RNA extraction. The remaining 21 leaf samples for re-sequencing were collected from the original species habitat in Xichou, Maguan and Jinping Counties from 2017 to 2019 (Table S1). Other DNA material from eight further species in the Magnoliaceae was used for comparison of genetic diversity and investigation of the phylogenic relationships. This DNA material was collected from specimens cultivated at KBG and the Germplasm Bank of Wild Species, Chinese Academy of Sciences (Table S2). After the leaves were collected, they were quickly packed in silica gel desiccant and stored in silica gel until re-sequencing.

2.2 Genome sequencing

Genomic DNA sequencing was performed using different sequencing platforms simultaneously to insure accurate assembly. (1) For ONT (Oxford Nanopore Technologies) PromethION sequencing, total DNA was extracted using the cetyltrimethylammonium bromide (CTAB) method [54] using a genomic DNA extraction kit (cat. no. 13323, Qiagen, Hilden, Germany). A NanoDrop™ One UV-Vis spectrophotometer (Thermo Fisher Scientific, USA) was then used to check DNA purity and a Qubit® 3.0 Fluorometer (Invitrogen, USA) was used to accurately quantify the DNA. After purification, the adapters from the LSK109 Ligation kit (cat. no. SQK-LSK109, Oxford) were used for the ligation reaction, and finally the Qubit® 3.0 Fluorometer (Invitrogen, USA) was used to quantify the constructed DNA library. The DNA library was subsequently transferred to NanoporePromethION (ONT, UK) for sequencing seven flow cells. (2) For Illumina sequencing, short-insert libraries were prepared using 2 μg of genomic DNA, and three

Illumina PCR-free libraries of 300–500 bp insertion size were constructed according to the standard manufacturer's protocol using the DNAseq Library Index Kit (Hangzhou Kaitai Biotechnology, Co., Ltd., Hangzhou, China). The whole-genomic libraries were sequenced on an Illumina Hiseq X Ten platform (RRID:SCR_020131). (3) The Hi-C library was prepared by Beijing Ori-Gene Science and Technology Co., Ltd., Beijing, China. High molecular weight genomic DNA (≥700 ng) was cross-linked *in situ*, extracted and then digested with a restriction enzyme. The DNA ends were then marked with biotin-14-dCTP, and the crosslinked fragments were blunt-end ligated. Fragments were sheared to a size of 200–600 bp with sonication. The Hi-C libraries were amplified using 12–14 cycles of PCR, and were sequenced in Illumina HiSeq X Ten platform. (4) Transcriptome sequencing was performed on a PacBio Sequel (Pacific Biosciences, Menlo Park, CA, USA) platform (RRID:SCR_017989) using full-length isoform sequencing (iso-seq) [55]. High-quality RNA was extracted with a Qiagen kit while a series of RNA samples were tested: Nanodrop was used to assess RNA purity, Qubit was used to precisely quantify the RNA, and an Agilent 2100 Bioanalyzer was used to calculate RIN values and 28S/18S. Then a SMARTer ® PCR cDNA Synthesis Kit was used to reverse transcribe the RNA into cDNA, The reverse transcription products were amplified using KAPA HiFi PCR Kits, and the amplified products were used to construct a SMRTbell library using a SMRTbell template prep kit 1.0. The third-generation sequencer Sequel was used to sequence the full-length cDNA to obtain high-qualitytranscriptome sequencing data.

2.3 Genome assembly

We obtained ~203 Gb (~100×) ONT reads, ~215 Gb (~110×) Illumina Hiseq reads, ~222b G Hi-C reads, and ~24 Gb iso-seq reads (Table S3–S6). The *de novo* genome assembly was first performed upon ONT reads using different assembly strategies. Briefly, the long noisy ONT reads

were first corrected with NextDenovo [56] and then assembled with SMARTDENOVO (RRID:SCR_017622) [57] and WTDBG (assembly v0.2), respectively [58] (Table S7–9). Primary assembly v0.1 was selected as the optimal assembly due to the low error rate. Then, the Illumina sequencing reads were used to improve base-level accuracy of the assembly with Pilon [59]. The two draft assemblies (v0.1 as reference and v0.2 as query) were then merged using QuickMerge to improve continuity [60] and then polished again using pilon (Table S10–12). The GetOrganelle software was used to assemble the mitochondrial (parameters:-R 50 -k 67,87,107,127 -F embplant_mt -w 125) and chloroplast (-R 15 -k 67,87,107,127 -F embplant_pt -w 125) genomes, respectively , and Bandage was used for manually adjustment [61, 62].

Hi-C reads were mapped to the draft assembly with Juicer, and a candidate chromosome-length assembly was generated automatically using the 3d-DNA pipeline to correct mis-joins, order; and orientation, and to anchor contigs [63, 64]. Manual review and refinement of the candidate assembly was performed in Juicebox Assembly Tools (JBAT) for quality control and interactive correction [65]. To reduce the influence of chromosome interactions and to further improve the chromosome scale assembly, each chromosome was separately re-scaffolded with 3d-DNA, and was then manually refined with Juicebox (RRID:SCR_021172). Finally, the chromosomal and unanchored sequences were generated, with the gap length set as 100 bp.

To fill the assembly gaps, LR_Gapcloser (default parameters) was run for two rounds based on ONT reads, and then NextPolish (default parameters) was run for three rounds to polish the assembly based on Illumina reads [66, 67]. In order to eliminate redundancy and external source pollution: 1) Redundant was used to remove the redundant unanchored sequences (identity ≥ 0.98) [68]; 2) Unplaced contigs with a length of less than 5 kb were removed; 3) The assembly was aligned

with the NT database [69] using BLASTN combined with coverage depth and GC content, to determine whether there was contamination from other species; and 4) Haplotigs or fragments with low average coverage depth (less than 75% of the peak depth) were removed with manual curation. The chromosomes were coded as chr01-chr19 according to their lengths (from long to short) (Fig 2a, b). The numbers, lengths and proportions of the chromosomes, unanchored sequences, and chloroplast and mitochondrial sequences are summarized in Table S13.

2.4 Assessment of genome assembly

The completeness of the final assembly was evaluated using BUSCO (RRID:SCR_015008) and LAI (LTR Assembly Index) [66, 70]. KAT was used to compare the genome assembly and the Illumina reads (Fig S1). Bwa was used to map the Illumina reads to the genome and Minimap2 was used to map the third-generation ONT and PacBio transcriptome(iso-seq) CCS reads to the genome [71, 72]. The non-primary alignment was removed, so that each read only mapped once and the mapping ratio and coverage percentage were also calculated (Table S14). The coverage depth of single-copy and multi-copy core genes should be consistent with a Poisson distribution if without redundancy after checking (Fig S2). The second-generation reads were mapped to the genome with Bwa, and mutation sites were detected using SAMtools/BCFtools (RRID:SCR_005227) [73]. The single base heterozygous sites were used to calculate the heterozygosity rate, and homozygous sites were used to calculate the error rate. Juicer was used to map the Hi-C data to the final genome assembly. The chromosome clustering heatmap of *M. sinica* was adequate, and there was no obvious chromosome assembly errors (Figure 2a, 2b) [64].

2.5 Genome annotation

The repeat libraries were generated by *de novo* identification of the repeat region family using

the RepeatModeler software. LTR_retriever (RRID:SCR_017623) was also used to identify the intact LTR (long terminal repeat retrotransposons), and then a second library was clustered and generated [72]. After combining these two libraries directly, we used RepeatMasker (RRID:SCR_012954) to identify repeated regions on the genome. Transcripts were generated following the process of isoseq3 [74] and were annotated to the genome using the PASA pipeline (RRID:SCR_014656) [75]. The results were used to train an AUGUSTUS model for five rounds of optimization [76]. 154,904 non-redundant protein sequences from *Liriodendron chinense* [38], *Cinnamomum kanehirae* [77, 78], *Piper nigrum* [79], *Amborella trichopoda* [80] and *Arabidopsis thaliana* [81] were used as evidence of homologous proteins for gene annotation.

Gene structure annotation was conducted using the Maker2 pipeline [82]. Briefly, AUGUSTUS (RRID:SCR_008417) was used to perform *ab initio* prediction of the genome with the repetitive regions masked out [76]. Transcripts were aligned with the genome using BLASTN (RRID:SCR_001598), and BLASTX (RRID:SCR_001653) was also used for aligning the protein evidence with the genome. Exonerate was used to optimize the alignments [83]. Based on the above three categories of evidence, hints files were generated, to allow AUGUSTUS to ultimately synthetically predict the gene models. AED (annotation edit distance) scores of each gene model were calculated according to the transcript and homologous protein evidence within the pipeline. Finally, false annotations in the coding frame and overly short (≤50 AA) gene annotations were removed. The software tRNAScan-SE, Barrnap [84] and Rfamscan was used to annotate tRNA, rRNA and other non-coding RNA, respectively [85]. BUSCO was used to evaluate the integrated annotated proteins [70].

The functions of protein coding genes were annotated based on three strategies. Firstly, genes

were mapped with the eggNOG database using eggNOG-mapper to annotate gene function, including GO and KEGG annotation [86]. Secondly, for assignment based on sequence conservation, a diamond search of the protein sequences from several protein databases was performed, including the databases Swiss-Prot, TrEMBL, NR, and the *Arabidopsis* database [87]. Lastly, for assignment based on domain conservation, InterProScan was used to examine conserved amino acid sequences, motifs and domains of proteins by matching against sub databases of several InterPro databases, including CDD, PANTHER, PRINTS, Pfam, and SMART [88].

2.6 Gene family identification and phylogenetic analysis

OrthoFinder2 was used to infer orthogroups, with the parameters set to "-M msa" [89]. A protein alignment of 1070 orthogroups with minimum of 87.5% of species having single-copy genes in any orthogroup obtained from OrthoFinder2 was used to construct a phylogenetic tree using IQTREE, using a maximum likelihood method (the best model was JTT+F+R5, 1000 bootstrap replicates) [90]. In addition, ASTRAL was also used to infer the species tree based on 3841 gene trees with genes in at least 70% taxa being single-copy. MCMCTree, from the PAML package, was used to estimate species divergence time and the mutation rate in *M. sinica*, based on the codon alignment of 211 1:1 non-missing single-copy orthologous genes [91]. Four fossil calibration time points were chosen: stem Nymphaeaceae (113 Mya), stem Poaceae (55.8 Mya), stem Lauraceae (104 Mya), and stem Santalales (65.5 Mya) [92, 93]. The root time of the phylogentic tree was set according to previous studies [92, 93]. Based on the time tree and 123, 06 homologous gene families, CAFE was used to assess the expansion, contraction and rapid evolution of the gene families [94].

Based on the orthologous and paralogous gene relationships inferred with OrthoFinder2, collinearity between and within species was analyzed using MCScanX_h [95]. According to the

collinear homologous gene pairs, the protein sequences were first aligned with MUSCLE [96], and then transformed into codon alignment with PAL2NAL [97]. Ka and Ks were then calculated between homologous gene pairs using KaKs_Caculator v2.0 (YN model) [98, 99]. Polyploidization events and time were inferred based on collinearity in combination with the Ks value [99].

2.7 Genome mapping and SNP calling

A total of 43 samples, including 21 samples of *M. sinica* and 22 samples of a further eight *Magnolia* species, were sampled for whole genome resequencing (Table S1, S2). A total of 5,687 million reads were produced across all samples. The raw data were filtered using fastp [100] to trim away the adaptors and low-quality regions. The cleaned reads were mapped to the reference genome using BWA-MAM [71] with the default parameters. The markdup model in SAMtools [73] was used to mark and to remove duplicate reads. To improve the accuracy of the subsequent analyses, we only retained bases with a quality score > 20 and mapping quality > 30 (as the filter parameters in ANGSD and Freebayes). We removed the sites with a mapping depth across all samples of < 100 or > 600 as well as the sites not mapped to chromosomes, using SAMtools. 1,585,988,829 sites (Dataset 1) from the BAM files were retained after quality control.

Freebayes (RRID:SCR_010761) [101] was used to process SNPs calling for *M. sinica* and a total of 176,087,519 variable sites were obtained. The resulting SNP dataset was then filtered with vcftools (RRID:SCR_001235) [102] using the following criteria: 1) sites with a genotype quality < 20 or genotypes with depth < 5 were treated as missing; 2) non-biallelic and non-SNP sites; 3) SNPs with missing rate > 20% (Dataset 2: 11,438,677 SNPs); 4) SNPs with minor allele frequency (MAF) < 0.05 (Dataset 3: 8,149,323 SNPs).

2.8 Population genetics

PopLDdecay was used for linkage disequilibrium analysis across the *M. sinica* genome. The ThetaStat module in ANGSD (RRID:SCR_021865) v0.93 [103] was used to assess genome wide diversity by calculating different estimators of θ, including θ_W (Watterson's θ) [104] and $\theta\pi$ (nucleotide diversity), and Tajima's *D* [105], and Fu and Li's *D* [106]. These statistics were calculated in a window size of 20 kb and a step size of 10 kb according to the result of LD decay, using Dataset 1 generated previously. Individual heterozygosity was also calculated in ANGSD v0.93 for *M. sinica* in our research.

For population structure analysis, we first used PLINK (RRID:SCR_001757) [107] to remove linkage sites from Dataset 3 with the parameter "--indep-pairwise 50 10 0.2", and we obtained a total of 454,661 independent SNPs (Dataset 4). Dataset 4 was further used to explore the population structure of *M. sinica* using the program Admixture v1.3.0 [108], and the most likely number of genetic clusters (ancestor numbers, K) was selected based on 10-fold cross-validation error (CV) value. Fig S3 contains a schematic diagram showing how these datasets were generated.

2.9 Ancestral sequence reconstruction

We mapped data from several samples of other species of *Magnolia* and a sample of *Liriodendron* (Table S15) to the *M. sinica* genome using BWA-MEM with the default parameters. At the same time, we used freebayes to call the genotype with the same filter parameters as the SNP calling described above, except that "--report-monomorphic" was used to keep monomorphic genotypes in the output. Phylogenetic trees were constructed using IQtree with the substitution model MFP+ASC and using *Liriodendron chinense* as the outgroup. We then used an empirical Bayesian method in IQtree [90] to reconstruct the ancestral state of each site of each chromosome; this method can produce accurate ancestral sequence reconstruction [109] and has been previously

used to reconstruct ancestral state in other works [23, 110–112]. Finally, we reclassified the ancestral state according to the posterior probability of each site. Posterior probabilities ≥ 0.95 were classed as “high confidence”; lower probabilities were considered to be ambiguous and were marked as "N". The sequence from the crown group of *Magnolia* species were defined as ancestral state.

2.10 Inference of demographic history

A Stairway plot was used to infer the demographic history of *M. sinica* [113]. The mutation rate was estimated as 1.2e-7 per locus per generation which was constructed using MCMCTree based on the four-fold degenerated sites (4D sites) of orthologous genes. The generation time was set as 30 years, based on the cultivation records of this species in KBG. Dataset 1 was further filtered by removing the sites within 5 kb of gene regions to ensure site neutrality, and 897,314,345 genomic sites were retained (Dataset 5). The unfolded Site Frequency Spectrum (SFS) for *M. sinica* was estimated using the functions doSaf and realSFS in ANGSD v 0.921 [103] with Dataset 5 and the recommended filtering parameters “-minMapQ 30 -minQ 20”.

We also used the Pairwise Sequentially Markovian Coalescent (PSMC) model to reconstruct the demographic history of *M. sinica* [114]. Using the BAM files (Dataset 1) generated by BWA-MAM and the markdup model in SAMtools [73], we made a consensus fastq file for each sample using SAMtools and BCFtools with the parameter set to -C50 to downgrade the mapping quality for reads containing excessive mismatches. The script vcfutils.pl was used to keep the minimum read depth to 5× and the maximum read depth to 50 for all individuals. The consensus fastq file was converted into an input file for PSMC using fq2psmcfa with the parameter -q 20 set, to remove consensus calls with qualities ≤ 20. The PSMC analysis was run using default values for the upper limit to assign a date to most recent common ancestor (-t 15) and theta/rho (-r 5). The atomic time

interval pattern (-p) was set to "4+30*2+4+6+10". We plotted the results using the same mutation rate and generation time as described above.

The contemporary effective population size of *M. sinica* was assessed using the linkage disequilibrium method in NeEstimator V2 [103] with the reduced Dataset 4 (filtered by vcftools with --max missing 0.95 and --thin 60000) to ensure accuracy [115].

2.11 Estimation of deleterious mutations and inbreeding

Accumulation of deleterious mutations is likely to impact species fitness. The Sorting Intolerant from Tolerant (SIFT) algorithm [116] was used to predict deleterious mutations, with the ancestral sequences reconstructed above as a reference. The TrEMBL plant database [117] was used to search for orthologous genes. After polarization of Dataset 2, protein-coding variants of 8,896,099 retained SNPs were categorized as nonsynonymous or synonymous sites. Nonsynonymous sites were further divided into deleterious (SIFT score <0.05), and tolerated (SIFT score ≥0.05) based on their SIFT score [118]. We also calculated the derived allele frequency (DAF) of deleterious mutations.

In addition, frequency of runs of homozygosity (FROH) has been used as a robust estimate of genomic inbreeding [119] and was estimated following previous research [120, 121]. Briefly, runs of homozygosity (ROH) were first identified based on Dataset2 using vcftools v0.1.17 with parameter "--LROH " [102], then FROH was calculated with the total length of ROH divided by the genome size of *M. sinica*.

Results

3.1 Genome sequencing and assembly

The libraries sequenced on the ONT PromethION platforms using seven cells resulted in the

generation of a total of 9.11 million reads with ~202.85 Gb sequencing data (~100×), with an average read length of 22 kb (the longest read was 194 kb, and N50 was 25 kb) (Table S3). A total of 1,432 million reads were generated with ca. 214.95 Gb (~110×) data using the Illumina HiSeq platform (Table S4). A total of 1,480 million reads with ca. 222.13 Gb data were produced with Hi-C sequencing (Table S5). Through the optimal assembly method, the final size of the assembled *M. sinica* genome was 1.84 Gb, which was similar to the 1.9 Gb genome size estimated using k-mers (Figure S4, Table S10, S11). A total of 108 contigs (1.82 Gb, accounting for 99.08% of the whole genome) with an average size of 15 Mb were anchored onto the 19 chromosomes. The contigs N50 of the *M. sinica* genome was ca. 45 Mb and the scaffold N50 ca. 92 Mb, both of which were much higher than those of other previously reported magnolia genomes (Table 1) [37–40]. In addition, the mitochondrial and chloroplast genomes were assembled into circular DNA molecules of 856,922 bp and 160,070 bp, respectively. The LAI value was estimated to be 10.3 based on LTR, indicating that the gene integrity was relatively good (Table S11, S12). We also calculated that the heterozygosity rate in *M. sinica* was about 1.21%, and that the error rate was about 0.0072%.

1,580 (97.9 %) complete BUSCO genes, including 1,522 (94.3 %) complete and single-copy genes and 58 (3.6 %) complete and duplicated genes were identified among the 1,614 total BUSCO groups. However, 8 (0.5 %) genes were found to be fragmented and 26 (1.6 %) genes were missing based on the BUSCO analysis (Table S11).

3.2 Genome annotation

A total of 2,329,558 repetitive sequences were identified in the *M. sinica* genome, with a total length of ~1.05 Gb, and accounting for 56.99 % genome. Of these, the highest proportion was LTR, accounting for 48.9% of the whole genome (Table S16). The most abundant repeat element families

were Copia (388,301, 14.88 %) and Gypsy (759,932, 27.40 %) (Table S16). A total of 18 million subreads with ~24.58 Gb data were generated from transcriptome sequencing, from which 43,473 protein-coding genes were annotated (Table S6, S17). The mean lengths of gene region, transcript, and coding DNA sequences were 11,297, 1,552, and 1,091, respectively (Table S17). Moreover, 71 rRNA, 658 tRNA, and 511 ncRNA sequences were identified (Table S18). A total of 38,041 genes were annotated using GO (14,360, 33.03 %), KEGG (14,937, 34.36 %), eggNOG (29,585, 68.05 %) and COG (31,414, 72.26 %). Based on sequence conservation, several protein databases, including Swiss-Prot (21,220, 48.81 %), TrEMBL (31,720, 72.96 %), NR (31,242, 71.87 %) and *Arabidopsis thaliana* (25,007, 57.52 %) were annotated with diamond. For assignment based on domain conservation, certain other database, including Pfam (25,850, 59.46%), Coils (2,533, 5.83%), CDD (28,110, 64.70%), SMART (8,247, 18.97%) and others were annotated with InterProScan. (Table S19)

3.3 Analysis of phylogeny, collinearity and WGD

In order to investigate the early evolution of the core angiosperms, we identified 579,290 homologous genes belong to 20,538 gene families from the 18 related genomes using OrthoFinder2 (Fig S5). A total of 1,266 expanded and 1,276 contracted gene families in *M. sinica* were identified and annotated (Fig 2c). A maximum likelihood tree was constructed using 1,070 orthogroups of 18 species. As shown in the ML phylogenetic tree (Fig 2c), magnolias formed a sister relationship with both the eudicots and the Ceratophyllales, while the monocots were sister to the other core angiosperms. The Magnoliales and the Laurales were predicted to have diverged from the Piperales at ca. 149.3 Ma (137.7–160), a result which was slightly different from that of a whole-genome study of black pepper, in which the differentiation time was estimated at 175–187 Ma [79]. The

Magnoliales were predicted to have diverged from the Laurales at ca. 122.2 Ma. In the Magnoliales, the estimated differentiation time of the genera *Magnolia* and *Liriodendron* was predicted to be 23.4 Ma, and within Magnolia, the closely related species *M. sinica* and *M. biondii* are estimated to have diverged ca. 10.9 Ma.

A total of 7,807 colinear gene pairs on 779 colinear blocks were inferred within the *M. sinica* genome. The collinearity depth ratio between *M. sinica* and *Liriodendron chinense* was 1:1 (Figure S6), indicating that the two species have no species-specific whole-genome duplication (WGD) events. Collinearity between these two species and earlier differentiated dicotyledons such as grapes was always 2:3 (Figure S7, S8), indicating that *M. sinica* and *L. chinense* experienced a WGD event after differentiation from the eudicots which is consistent with the conclusions of the study investigating *L. chinense* [38]. Similarly, the collinearity with the early angiosperms *Amborella trichopoda* and *Nymphaea tetragona* was 2:1 and 2:2 (Figure S9, S10), respectively, which indicates that *M. sinica* and *L. chinense* only experienced a single shared WGD event after their differentiation from these plants. From the paralogous collinearity block in *M. sinica*, it can be seen that this WGD event occurred at a Ks value of about 0.75. Based on the chromosome tree analysis, the Magnoliaceae and the Lauraceae share a WGD event, but this is not shared with pepper. After differentiation from other species, the Magnoliaceae (*M. sinica* and *L. chinense*) experienced a single WGD event, the Lauraceae (*Cinnamomum kanehirae*) experienced two WGD events, and pepper experienced three WGD events.

3.4 Genome wide diversity and population structure

After filtering out low quality reads and adapter sequences, 5,386 million reads remained for processing (Table S20). The sequencing depth of *M. sinica* samples ranged from 8.8× to 12.6×, with

a mean value of 10.5×, and were between 10.8–14.3× for the other eight *Magnolia* species (Table S20). The mapping rates of *M. sinica* ranged from 90.80% to 99.70%, with a mean value of 97.63 %, and were 95.30%–99.53% for the other eight *Magnolia* species (Table S20).

The mean heterozygosity rate of *M. sinica* was (1.29 ± 0.07) % (Table S21), ranging from 1.12 % to 1.38 %, and the trees with the lowest and the highest heterozygosity rates were both found in the XZQ population. The MAD population had the lowest heterozygosity (1.19 %), while the DLS population had the highest heterozygosity (1.32 %).

Nucleotide diversity in *M. sinica* was estimated using two parameters. Watterson's θ (θw) and genome wide diversity ($\theta\pi$) of *M. sinica* were calculated as 0.01416 and 0.01494, respectively (Table S22). When compared with other species, *M. sinica* was found to have higher genetic diversity (Table S23), and was approximately 12 folds higher than that of *Liriodendron chinense* (0.00123) [38].

The population structure results showed that the CV error was smallest when there was an optimal number of clusters K = 1 (Figure S11), suggesting low genetic differentiation among populations of *M. sinica.* Low genetic differentiation among populations was further suggested by the low F_{st} statistics between population pairs of *M. sinica*, which had a mean value of 0.133. We have given the structure results for $K = 2$ and $K = 3$ in Figure 3b. At $K = 2$, all the populations of *M. sinica* could be separated into three components, including an XZQ component (blue), the component (orange) from the FD population, and two individuals (KIBDZL15301 and KIBDZL15303) from the DLS population, as well as a mixture component. When $K = 3$, the FD population was further separated into two components, including an FD component and a mixture component. Both the XZQ and FD populations were genetically “pure” from the other *M. sinica*

populations. The MAD and MC populations were genetically similar irrespective of *K*.

3.5 Demographic history

The demographic history of *M. sinica* inferred by Stairway plot2 indicate three significant population declines, two of which were also detected by PSMC (Figure 3c). In the scenario inferred from Stairway plot2, the earliest population decline occurred at 1.3 Ma and continued until 1.1 Ma. For the scenarios inferred by the PSMC, the earliest population decline occurred at 1.5 Ma and continued until 0.8 Ma. After this, the population of *M. sinica* is predicted to have experienced a period of recovery in both scenarios. The second population decline occurred at about 0.3 Ma in both scenarios. After that, the population of *M. sinica* exhibited recovery in the scenario inferred by Stairway plot2, but experienced a continuing decline in PSMC. The latest population bottleneck in both scenarios occurred at about 20 Ka and continued until 10 Ka, when the effective population size of *M. sinica* dropped to 1,936 in the Stairway plot and 1,784 in PSMC. However, after 10 ka, the effective size of the *M. sinica* population recovered in Stairway plot, but showed continuous decline in PSMC. The contemporary effective population size of *M. sinica* estimated by NeEstimator was 10.9 (3.3–43.7 Jackknife CI).

3.6 Genetic load and genomic inbreeding coefficient

1,196,374,340 high confidence loci were obtained and used as ancestral sequences to predict deleterious mutations. 16,131, 74,385 and 36,827 sites were predicted to be deleterious, synonymous and tolerated, respectively, in the 21 re-sequenced *M. sinica* individuals (Table S24). The mean value of derived homozygous deleterious alleles (HoDA) was 249, ranging from 190 to 298, with the lowest found in the MC population, which had a mean number of 207 (190–216), and the highest found in XZQ, which had a mean number of 258 (220–298) (Table S25). The MAD

population also harbors a very high number of HoDA (246), and this population had highest proportion of private HoDA (118, 48%) when compared with other populations (Figure 3d, Table S25). None of the HoDA was shared among all five of these populations. An average of 2,607 heterozygous deleterious alleles (HeDA) was detected in *M. sinica*, ranging from 2,136 to 2,967. The highest number of HeDA was found in the XZQ population, which had a mean value of 2,593 (2,136–2,967) (Table S25), while the lowest number of HeDA was found in the MAD population (2,430). The MAD population shared the highest HeDA with the MC population, and shared the lowest HeDA with XZQ. None of the HeDA was shared among all five of the populations (Table S25). The derived allele frequency (DAF) of approximately 32.35% of the deleterious mutations was < 0.05, and all these rare deleterious mutations were heterozygous. Only ~7.1% (1147/16131) of the deleterious mutations were homozygous (DAF > 0.05) (Figure S12).

At the population level, the mean value of FROH in *M. sinica* was 0.11 ± 0.04, ranging from 0.08 to 0.16, with the lowest value found in the DLS population, and the highest value found in MAD. At the individual level, one individual (KIBDZL15801) from the XZQ population showed the lowest levels of inbreeding, and had the lowest FROH value (0.06). The individual (KIBDZL15803) with the largest FROH value (0.21) was also found in XZQ population (Table S25).**4.**

DISCUSSION

To date, only four species in the Magnoliaceae (*Liriodendron chinense*, *Magnolia officinalis*, *M. obovata* and *M. biondii*) have been the objects of in-depth genomic research, and this has been mainly from the perspective of confirming the phylogeny of the angiosperms, investigation of species differentiation and the biosynthesis of terpenoids. To date, no species in the family Magnoliaceae have been studied at a genome-wide level from the perspective of conservation [38–

41]. From the aspect of conservation genomics, we report high-quality whole-genomic data from *M. sinica* (1.84 Gb with contigs N50 of ca. 45 Mb). This is superior to the data available from *Liriodendron chinense* (1.74 Gb with contigs N50 of ~1.43 Mb) [38], *Magnolia officinalis* (1.68 Gb, with contigs N50 of 0.22 Mb) [40], *M. obovata* (1.64 Gb, with contigs N50 of 1.71 Mb) [41] and *M. biondii* (2.22 Gb with contigs N50 of 0.27 Mb) [39].

The early evolution of the core angiosperms has been studied with whole-genome analysis of certain species of Magnoliids and Chloranthales [39, 77, 120, 122–125]. However, the phylogenetic relationships between the Magnoliids on the early branch of the angiosperm lineage and the eudicots and monocots have been controversial and not fully resolved [124, 125]. Our genome level phylogenetic tree suggests that the magnolias form a sister group to the eudicots and the Ceratophyllales, while the monocots are sister to the other core angiosperms. This is consistent with the results of a study into Chloranthales [120, 124], but inconsistent with the relevant results of *M. biondii*, *M. hypoleuca* and *M. officinalis* [39–41]. The evolutionary history of the angiosperms was accompanied by frequent WGD events. However, evidence of WGD events was inferred from dot plots and Ks, which is insufficient to demonstrate whether any two species very close to differentiation share a WGD event. In our study, we concatenated homologous genes to construct a chromosome-level synteny tree to make our inferences more reliable. Our inference results suggest that WGD events also occurred after the differentiation of the magnoliids from other groups, which is in agreement with other studies [125].

Genetic diversity is essential to allow species evolution in response to environmental changes, and has been predicted to be positively correlated with species fitness and evolutionary potential [126]. We found that *M. sinica* had relatively high genetic diversity, which is consistent with

previous research based on SSR markers [49]. This high diversity could be explained by the fact that, as a tree species, *M. sinica* has a long life span (ca. 30 years). De Kort et al. (2021) [127] compared the genetic diversity of 164 annuals, 1,405 perennials, 308 shrubs and 2,337 trees, and found that although species level diversity is lower for long-lived or low-fecundity species than for short-lived or high-fecundity species, population level genetic diversity is usually higher for long-living plants, as they may respond more slowly to reduced gene flow. Another reason for this high diversity could be that *M. sinica* is found in southern subtropical monsoon broadleaved evergreen forests [5, 48]. Species around the equator are expected to have higher population-level genetic diversity than other species. This is because in theoretical prediction analyses, the abundant precipitation around the equator shows a significant relative contribution to population genetic diversity, although the exact mechanisms and extent of this are still unknown [128]. Moreover, the pollinator-dependent pollination system may contribute to the high genetic diversity in *M. sinica* [49].

M. sinica has low genetic differentiation between subpopulations, which could be attributed to higher gene flow among subpopulations, despite the fragmented distribution of the species [49]. The species has an outcrossing mating system, which is pollinator dependent, and two species of beetles appear to be effective pollinators [5, 48]. Previous research has demonstrated that some beetles can fly up to 12 km [128]. Long-distance pollen-mediated gene flow among populations may decrease population genetic differentiation [129]. The smaller FROH and lower inbreeding load in *M. sinica* compared with *Acer yangbiense* may also indicate the existence of certain gene flow among its isolated populations [121], or from other populations which we have not found. As most of the reported populations of *M. sinica* are found on the borders of China with other countries, it is not

unreasonable to suggest that other unreported individuals or populations exist outside China.

Southeast Yunnan is an important biodiversity hotspot [130], and is shielded by the Ailao Mountains from the climate fluctuations caused by glaciation and the uplift of the Himalayas and the Hengduan Mountains [131]. From the geological point of view, there is no evidence that Southeast Yunnan was affected by the Quaternary ice age, and simulations of climate data suggest that this area was not seriously affected by the global temperature drop [132]. In our results, Stairway plot2 detected major population declines, which is similar to the inferred demographic history of the sympatric *Magnolia fistulosa* [133]. Each *M. sinica* population decline inferred in the Stairway plot could be verified in PSMC (Figure 3c). However, the demographic history of *M. sinica* inferred by Stairway plot2 shows population rebound after each decline, which was not obvious in the PSMC analysis. Moreover, the Stairway plot can estimate very recent events, while PSMC estimates only up to 10,000 years ago (Figure 3c). The earliest inferred population decline occurred 1.0–1.2 Ma, which is consistent with the mid-Pleistocene transition [134]. Population declines at a similar time are also reflected in other sympatric species such as *Acer yangbiense* [121], and *Buddleja alternifolia* [120]. The second population decline occurred at 0.3 Ma, during which global temperature experienced a general decline [135]. The latest population decline occurred at ca. 20 Ka, and may have been caused by the Last Glacial Maximum (19.0–26.5 Ka) [136]. Multiple population declines may have resulted in a narrow distribution of *M. sinica*, and the stable population sizes from about 1 ka inferred in the Stairway plot may be as a result of the very recent large-scale anthropogenic land development and land use changes in the habitat of *M. sinica*, and is likely to have been responsible for the extremely rare status of this species [27], this is also consistent with the characteristics of high genetic diversity and low genetic differentiation of this

species. Genetic differentiation tends to be lower among populations separated in recently than those isolated from historical, especially for species with long generation times [137]. *M. sinica* has a pollinator-dependent outcrossing mating system, which may contribute to its high genetic diversity; while high gene flow among populations may maintain links between populations of this species, and may contribute to its low genetic differentiation. The recent reduction in population size due to anthropogenic activities has led to isolation state of the populations, leading to the high genetic diversity and low genetic differentiation now observed in the fragmented populations of this endangered tree species. Similar patterns have been reported in *Michelia coriacea*, another species in the Magnoliaceae [138].

The MAD population contains only a single remnant individual with a higher level of inbreeding (FROH = 0.16), lower heterozygosity rate (1.19%) and higher homozygous deleterious allele number (246) than other populations. Gene flow has been proposed as a potential strategy to sustain small and isolated populations, by masking of deleterious alleles [139]. We found that the DLS population had a higher heterozygosity rate (1.32%) and shared few homozygous deleterious mutations with tree from the MAD population. The DLS population could therefore serve as source material for breeding, which could be used to mask homozygous deleterious mutations in future MAD population individuals. Methods such as population reinforcement, hand pollination to assist pollen flow (by collecting pollen from the DLS population and pollinating the MAD population), or the transplantation of seedlings from the DLS population into MAD could also be considered. Similarly, an individual (KIBDZL15801) in the XZQ population also had a higher heterozygosity rate (1.37%), and a smaller number of HoDA (220) than the MAD population. Pollen from KIBDZL15801 could therefore be used to assist gene flow to KIBDZL15803 and KIBDZL15807,

two other individuals from the XZQ population with lower heterozygosity rates (1.12 % and 1.16 %, respectively) and higher numbers of HoDA (298 and 286, respectively).

The identification of a management unit (MU) is essential for the management of natural populations [140]. The FD population was genetically pure, and had no admixture with other populations even when $K = 2$ and $K = 3$. This could be attributed to its distance from the other populations (about 66–145 km), which may decrease opportunities for pollen flow. Similarly, population XZQ was also found to be genetically pure at $K = 2$ and $K = 3$. We therefore suggest that the FD and XZQ populations be treated as two separate evolutionarily significant units (ESU). The MAD and MC populations were genetically similar at all values of K, and we suggest that they be treated as another ESU. Importantly, however, the MAD and MC populations are found outside any existing nature reserves, and it is therefore necessary to include these populations in a nature reserve or to establish specific conservation regions to protect them.

The main threats currently faced by *Magnolia sinica* are as follows: (1) Substantial reduction and loss of the original habitat leading to severe habitat fragmentation and population isolation; (2) The large-scale planting of *Amomum tsaoko* under forest cover means that *M. sinica* is unable to regenerate naturally in the wild, and there are no seedlings; (3) Excessive artificial seed collection. Fortunately, since 2005, because this plant is a critically endangered flagship species, comprehensive scientific research, including reproductive and seed biology, conservation genetics, and protection measures including field investigations, *in situ* conservation, *ex situ* conservation, and reintroduction have been gradually implemented [14, 48, 50, 51, 53]. At present, in addition to the existing protection measures, strengthening of the management of nature reserves and reduction of the disturbance by human activities in the original habitats of wild populations are urgently

needed. In particular, it is necessary to stop the large-scale planting of commercial crops (*Amomum tsaoko*) under these forests, which is important to restore their natural regeneration in the wild. Unlike most of the severely threatened species, *M. sinica* has high genetic diversity and low genetic differentiation which is also consistent with research into other endangered species in the Magnoliaceae [133, 141–143]. However, considering that the generation time of *M. sinica* can be as long as 30 years, the isolation of the various populations, the serious habitat fragmentation, and that there are very few wild individuals, we still need to consider potential future inbreeding depression. More artificial outcrossing strategies should be designed in the future to reduce the loss of genetic diversity caused by inbreeding, and that these strategies should be considered instead of collecting seeds and simply breeding more individuals [26]. Our genomic study into *M. sinica* provides an example of high genetic diversity and low genetic differentiation in a long-lived tree species and informs the future formation and maintenance of conservation strategies necessary for the survival of such a PSESP.

Data availability statement

The genome assembly, annotations, and other supporting data are available via the *GigaScience* database GigaDB [144]. The raw sequence data have been deposited in the Short Read Archive under NCBI BioProject ID PRJNA774088. The raw data, genome assembly and gene annotation have also been deposited at National Genomics Data Center, China National Center for Bioinformation under BioProject accession number PRJCA015437.

Additional Files

Figure S1. K-mer spectrum analysis.

Figure S2. Evaluation of the distribution of coverage depth over the whole genome and the BUSCO core gene region with Illumina and ONT data.

Figure S3. A schematic diagram showing how these datasets were generated.

Figure S4. Kmer frequency distribution diagram.

Figure S5. Maximum-likelihood (ML) phylogeny of *Magnolia sinica* and related taxa showing bootstrap values. Bar, substitutions per site.

Figure S6. The collinearity between *M. sinica* and *Liriodendron chinense*.

Figure S7. The collinearity between *M. sinica* and *Vitis vinifera.*

Figure S8. The collinearity between *Liriodendron chinense* and *Vitis vinifera*.

Figure S9. The collinearity between *Amborella trichopoda* and *M. sinica*.

Figure S10. The collinearity between *Nymphaea colorata* and *M. sinica*.

Figure S11. Cross validation error (CV) based on Admixture output.

Figure S12. Deleterious allele frequency distribution of homozygous deleterious SNPs.

Table S1. Collection information for the 21 re-sequenced samples of *Magnolia sinica*.

Table S2. Collection information for the other eight Magnoliaceae re-sequencing samples.

Table S3. WGS-ONT sequencing statistics.

Table S4. WGS-Illumina sequencing statistics.

Table S5. HiC sequencing statistics.

Table S6. Iso-Seq sequencing statistics.

Table S7. Assembly statistics (V0.1).

Table S8. Assembly statistics (V0.2).

Table S9. Assembly statistics (V0.3).

Table S10. Assembly statistics (V1.0).

Table S11. Assembly statistics (V1.1).

Table S12. Statistics of all assemblies.

Table S13. Information pertaining to the chromosomes, unanchored sequences, chloroplasts and mitochondria.

Table S14. The mapping ratio and coverage percentage of re-sequencing data.

Table S15. Sequences used to construct ancestral states.

Table S16. Repetitive sequences statistics.

Table S17. Final gene set statistics.

Table S18. Statistics of the source of integration annotation.

Table S19. Gene annotation statistics.

Table S20. Genome mapping statistics of sequencing data.

Table S21. Statistics of heterozygsity rate.

Table S22. Mean population fixation index and corresponding spatial distance.

Table S23. Genome wide diversity of woody species.

Table S24. SIFT (Sorting Intolerant From Tolerant) prediction of deleterious mutations.

Table S25. Genetic load of 21 individuals of *Magnolia sinia*.

Abbreviations

AED: annotation edit distance; Blast: Basic Local Alignment Search Tool; BUSCO: Benchmarking Universal Single-copy Orthologues; CBD COP 15: 15th Conference of the Parties, Convention on

Biological Diversity; DAF: derived allele frequency; ESTs: Expressed sequence tags; FROH: frequency of runs of homozygosity; GO: gene ontology; HeDA: heterozygous deleterious alleles; HoDA: homozygous deleterious alleles; JBAT: Juicebox Assembly Tools; KBG : Kunming Botanical Garden; KEGG: Kyoto Encyclopedia of Genes and Genomes; ONT: Oxford Nanopore Technologies; LAI: LTR Assembly Index; LTR: long terminal repeat retrotransposons; MAF: minor allele frequency; PSESP: Plant Species with Extremely Small Populations; PSMC: Pairwise Sequentially Markovian Coalescent; ROH: runs of homozygosity; θ_W: Watterson's θ; $\theta\pi$: nucleotide diversity; SFS: Site Frequency Spectrum; SIFT: Sorting Intolerant from Tolerant; SMRT: Single Molecule Real-Time; WGD: whole-genome duplication.

Competing interests

The authors declare no competing interests.

Authors' contributions

Y.P.M. and W.B.S. conceived and designed the study; R.G.Z., L.C., D.T.L. F.M.Y. and Q.Z.Y. analyzed the data; L.C., D.T.L. and F.M.Y. wrote the manuscript; Y.P.M., Z.L.D. and W.B.S. revised the manuscript. All authors reviewed and approved the final manuscript.

Acknowledgments

We thank Li-Dan Tao, Pin Zhang, Jia-Jun Yang, Rong-Li Liao for their help in collecting materials, and we thank Li-Sen Qian for helping to write the R script.

Funding

This work was supported by the National Science & Technology Basic Resources Investigation Program of China (Grant No. 2017FY100100); Yunnan Fundamental Research Projects (Grant No. 202101AT070173); National Natural Science Foundation of China (NSFC) (Grant No. 32101407);

and the National Natural Science Foundation of China (NSFC) – Yunnan Joint Fund (Grant No. U1302262).

References

1. Berry PM, Fabók V, Blicharska M, et al. Why conserve biodiversity? A multi-national exploration of stakeholders' views on the arguments for biodiversity conservation. *Biodivers Conserv.* 2018;**27**(7):1741–62. doi: 10.1007/s10531-016-1173-z.

2. Chen GS. Analysis on biodiversity conservation in the pluralistic vision. *Advanced Materials Research*. 2012;**518-523**:4980–4. doi: 10.4028/www.scientific.net/AMR.518-523.4980.

3. Isbell F, Gonzalez A, Loreau M, et al. Linking the influence and dependence of people on biodiversity across scales. *Nature*. 2017;**546**(7656):65–72. doi: 10.1038/nature22899.

4. Johnson CN, Balmford A, Brook BW, et al. Biodiversity losses and conservation responses in the Anthropocene. *Science*. 2017;**356**(6335):270–5. doi: 10.1126/science.aam9317.

5. Meng HH, Zhou SS, Li L, et al. Conflict between biodiversity conservation and economic growth: insight into rare plants in tropical China. *Biodivers Conserv*. 2019;**28**(2):523–37. doi: 10.1007/s10531-018-1661-4.

6. Wang W, Feng CT, Liu FZ, et al. Biodiversity conservation in China: A review of recent studies and practices. *Environ Sci Ecotech*. 2020;**2**:100025. doi: 10.1016/j.ese.2020.100025.

7. Zhang YZ, Qian LS, Spalink D, et al. Spatial phylogenetics of two topographic extremes of the Hengduan Mountains in southwestern China and its implications for biodiversity conservation. *Plant Divers*. 2021;**43**(3):181–91. doi: 10.1016/j.pld.2020.09.001.

8. McBeath J and McBeath JH. Biodiversity conservation in China: policies and practice. *J. Int. Wildl. Law Policy*. 2006;**9**(4):293–317. doi: 10.1080/13880290601039238.

9. Xu JC and Wilkes A. Biodiversity impact analysis in northwest Yunnan, southwest China. *Biodivers Conserv*. 2004;**13**(5):959–83. doi: 10.1023/B:BIOC.0000014464.80847.02.

10. Wyse Jackson P and Kennedy K. The Global Strategy for Plant Conservation: a challenge and opportunity for the international community. *Trends Plant Sci.* 2009;**14**(11):578–80. doi: 10.1016/j.tplants.2009.08.011.

11. López-Pujol J, Zhang FM and Ge S. Plant biodiversity in China: richly varied, endangered, and in need of conservation. *Biodivers Conserv*. 2006;**15**(12):3983–4026. doi: 10.1007/s10531-005-3015-2.

12. Ma YP, Chen G, Grumbine RE, et al. Conserving plant species with extremely small populations (PSESP) in China. *Biodivers Conserv*. 2013;**22**(3):803–9. doi: 10.1007/s10531-013-0434-3.

13. Sun WB, Ma YP and Blackmore S. How a new conservation action concept has accelerated plant conservation in China. *Trends Plant Sci*. 2019;**24**(1):4–6. doi: 10.1016/j.tplants.2018.10.009.

14. Sun WB, Yang J and Dao ZL. Study and Conservation of Plant Species with Extremely Small Populations (PSESP) in Yunnan Province, China. Beijing: Science Press; 2019.

15. Yang J, Cai L, Liu DT, et al. China's conservation program on Plant Species with Extremely Small Populations (PSESP): Progress and perspectives. *Biol Conserv*. 2020;**244**:108535. doi: 10.1016/j.biocon.2020.108535.

16. Sun WB. List of Yunnan Protected Plant Species with Extremely Small Populations (2021).

Kunming: Yunnan Science and Technology Press; 2021.

17. The Published Plant Genomes. Phylogenetic relationships for flowering plants with genomes sequenced and published. 2023. https://www.plabipd.de/plant_genomes_pa.ep

18. Marks RA, Hotaling S, Frandsen PB, et al. Representation and participation across 20 years of plant genome sequencing. *Nat Plants*. 2021;**7**(12):1571–8. doi: 10.1038/s41477-021-01031-8.

19. Zanini SF, Bayer PE, Wells R, et al. Pangenomics in crop improvement—from coding structural variations to finding regulatory variants with pangenome graphs. *Plant Genome*. 2022;**15**(1):e20177. doi: 10.1002/tpg2.20177.

20. Chen Y, Ma T, Zhang LS, et al. Genomic analyses of a "living fossil": The endangered dove-tree. *Mol Ecol Resour.* 2020;**20**(3):756–69. doi: 10.1111/1755-0998.13138.

21. Ding XP, Mei WL, Huang SZ, et al. Genome survey sequencing for the characterization of genetic background of *Dracaena cambodiana* and its defense response during dragon's blood formation. *PLoS One*. 2018;**13**(12):e0209258. doi: 10.1371/journal.pone.0209258.

22. Liu HL, Wang XB, Wang GB, et al. The nearly complete genome of *Ginkgo biloba* illuminates gymnosperm evolution. *Nat Plants*. 2021;**7**(6):748–56. doi: 10.1038/s41477-021-00933-x.

23. Ma H, Liu YB, Liu DT, et al. Chromosome-level genome assembly and population genetic analysis of a critically endangered rhododendron provide insights into its conservation. *Plant J.* 2021;**107**(5):1533–45. doi: 10.1111/tpj.15399.

24. Rodríguez del Río Á, Minoche AE, Zwickl NF, et al. Genomes of the wild beets *Beta patula* and *Beta vulgaris* ssp. *maritima*. *Plant J.* 2019;**99**(6):1242–53. doi: 10.1111/tpj.14413.

25. Sun YX, Deng T, Zhang AD, et al. Genome sequencing of the endangered *Kingdonia uniflora* (Circaeasteraceae, Ranunculales) reveals potential mechanisms of evolutionary specialization. *iScience*. 2020;**23**(5):101124. doi: 10.1016/j.isci.2020.101124.
26. Yang YZ, Ma T, Wang ZF, et al. Genomic effects of population collapse in a critically endangered ironwood tree *Ostrya rehderiana*. *Nat Commun*. 2018;**9**(1):5449. doi: 10.1038/s41467-018-07913-4.
27. Yang J, Wariss HM, Tao LD, et al. *De novo* genome assembly of the endangered *Acer yangbiense*, a plant species with extremely small populations endemic to Yunnan Province, China. *GigaScience*. 2019;**8**(7):giz085. doi: 10.1093/gigascience/giz085.
28. Xu B, Liao M, Deng HN, et al. Chromosome-level *de novo* genome assembly and whole-genome resequencing of the threatened species *Acanthochlamys bracteata* (Velloziaceae) provide insights into alpine plant divergence in a biodiversity hotspot. *Mol Ecol Resour*. 2022;**22**(4):1582–95. doi: 10.1111/1755-0998.13562.
29. Zhu SS, Chen J, Zhao J, et al. Genomic insights on the contribution of balancing selection and local adaptation to the long-term survival of a widespread living fossil tree, *Cercidiphyllum japonicum*. *New Phytol*. 2020;**228**(5):1674–89. doi: 10.1111/nph.16798.
30. Yang T, Zhang R, Tian X et. al. The chromosome-level genome assembly and genes involved in biosynthesis of nervonic acid of *Malania oleifera*. *Sci. Data*. 2023,**10**(1):298. doi.org/10.1038/s41597-023-02218-8.
31. Dong SS, Wang YL, Xia NH, et al. Plastid and nuclear phylogenomic incongruences and biogeographic implications of *Magnolia* s.l. (Magnoliaceae). *J Syst Evol*. 2022;**60**(1):1–15. doi: 10.1111/jse.12727.

32. Wang YB, Liu BB, Nie ZL, et al. Major clades and a revised classification of *Magnolia* and Magnoliaceae based on whole plastid genome sequences via genome skimming. *J Syst Evol*. 2020;**58**(5):673–95. doi: 10.1111/jse.12588.

33. Azuma H, García-Franco JG, Rico-Gray V, et al. Molecular phylogeny of the Magnoliaceae: the biogeography of tropical and temperate disjunctions. *Am J Bot*. 2001;**88**(12):2275–85. doi: 10.2307/3558389.

34. Figlar RB and Nooteboom HP. Notes on Magnoliaceae IV. *Blumea-Biodiversity, Evolution and Biogeography of Plants*. 2004;**49**(1):87–100. doi: 10.3767/000651904X486214.

35. Rivers M, Beech E, Murphy L, et al. The red list of Magnoliaceae-revised and extended. Richmond: Botanic Gardens Conservation International; 2016.

36. Xia NH, Liu YH, Nooteboom HP. Magnoliaceae. In: Wu ZY, Raven PH , editors. *Flora of China*, Vol. 7. Beijing: Science Press & St. Louis: Missouri Botanical Garden Press; 2008. p. 48–91.

37. Qin HN, Yang Y, Dong SY, et al. Threatened species list of China's higher plants. *Biodiv Sci*. 2017;**25**(7):696–744. doi: 10.17520/biods.2017144.

38. Chen JH, Hao ZD, Guang XM, et al. *Liriodendron* genome sheds light on angiosperm phylogeny and species–pair differentiation. *Nat Plants*. 2019;**5**:18–25. doi: 10.1038/s41477-018-0323-6.

39. Dong SS, Liu M, Liu Y, et al. The genome of *Magnolia biondii* Pamp. provides insights into the evolution of Magnoliales and biosynthesis of terpenoids. *Hortic Res*. 2021;**8**:38. doi: 10.1038/s41438-021-00471-9.

40. Yin YP, Peng F, Zhou LJ, et al. The chromosome-scale genome of *Magnolia officinalis*

provides insight into the evolutionary position of magnoliids. *iScience*. 2021;**24**(9):102997. doi: 10.1016/j.isci.2021.102997.

41. Zhou L, Hou F, Wang L, et al. The genome of *Magnolia hypoleuca* provides a new insight into cold tolerance and the evolutionary position of magnoliids. *Front. Plant Sci.* 2023;**14**:1108701. doi: 10.3389/fpls.2023.1108701.

42. Yuh-wu L. A new genus of Magnoliaceae from China. *J Syst Evol.* 1979;**17**:72–4.

43. Sun WB, Zhou Y, Li XY, et al. Population reinforcing program for *Magnolia sinica*, a critically endangered endemic tree in southeast Yunnan province, China. In: Maschinski J and Haskins KE, editors. Plant Reintroduction in a Changing Climate. Washington: Island Press; 2012. p. 65–69.

44. Wang S and Xie Y. China Species Red List (Vol 1). Beijing: Higher Education Press; 2004.

45. Cicuzza D, Newton A and Oldfield S. The red list of Magnoliaceae. Cambridge: Lavenham Press; 2007.

46. Anon. List of National Key Protected Wild Plants (First Group). *The Order of National Forestry Bureau and Agriculture Ministry of China 4*, 2–13.

47. National Forestry and Grassland Administration and Ministry of Agriculture and Rural Affairs of PRC. List of National Key Protected Wild Plants. 2021. https://www.forestry.gov.cn/main/3457/20210915/143259505655181.html

48. Chen Y, Chen G, Yang J, et al. Reproductive biology of *Magnolia sinica* (Magnoliaecea), a threatened species with extremely small populations in Yunnan, China. *Plant Divers*. 2016;**38**(5):253–8. doi: 10.1016/j.pld.2016.09.003.

49. Chen Y. Conservation biology of Manglietiastrum sinicum Law (Magnoliaceae), a plant

species with extremely small populations. PhD Thesis. University of Chinese Academy of Sciences; 2017.

50. Lin L, Cai L, Fan L, et al. Seed dormancy, germination and storage behavior of *Magnolia sinica*, a plant species with extremely small populations of Magnoliaceae. *Plant Divers*. 2022;**44**(1):94–100. doi: 10.1016/j.pld.2021.06.009.

51. Song EJ, Park S, Sun WB, et al. Complete chloroplast genome sequence of *Magnolia sinica* (Y.W.Law) Noot. (magnoliaceae), A critically endangered species with extremely small populations in Magnoliaceae. *Mitochondrial DNA B*. 2019;**4**(1):242–3. doi: 10.1080/23802359.2018.1546141.

52. Su DF, Shen QQ, Yang JY, et al. Comparison of the bulk and rhizosphere soil prokaryotic communities between wild and reintroduced *Manglietiastrum sinicum* plants, a threatened species with extremely small populations. *Curr Microbiol.* 2021;**78**(11):3877–90. doi: 10.1007/s00284-021-02653-z.

53. Wang B, Ma YP, Chen G, et al. Rescuing *Magnolia sinica* (Magnoliaceae), a Critically Endangered species endemic to Yunnan, China. *Oryx*. 2016;**50**(3):446–9. doi: 10.1017/S0030605315000435.

54. Doyle JJ and Doyle JL. A rapid DNA isolation procedure for small quantities of fresh leaf tissue. *Phytochem. bull*. 1987;**19**(1):11–5.

55. Gonzalez-Garay ML. Introduction to isoform sequencing using pacific biosciences technology (Iso-Seq). In: Wu JQ, editor. Transcriptomics and Gene Regulation. Dordrecht: Springer Netherlands; 2016. p. 141–60.

56. Nextomics. NextDenovo: Fast and accurate de novo assembler for long reads. GitHub. 2020.

https://github.com/Nextomics/NextDenovo

57. Liu H, Wu S, Li A, Ruan J. SMARTdenovo: a de novo assembler using long noisy reads. GigaByte. 2021 Mar 8;2021:gigabyte15. doi: 10.46471/gigabyte.15.

58. Ruan J, Li H. Fast and accurate long-read assembly with wtdbg2. *Nat methods*, 2020;**17**(2):155–158. doi.org/10.1038/s41592-019-0669-3.

59. Walker BJ, Abeel T, Shea T, et al. Pilon: an integrated tool for comprehensive microbial variant detection and genome assembly improvement. *PLoS One*. 2014;**9**(11):e112963. doi: 10.1371/journal.pone.0112963.

60. Chakraborty M, Baldwin-Brown JG, Long AD, et al. Contiguous and accurate *de novo* assembly of metazoan genomes with modest long read coverage. *Nucleic Acids Res*. 2016;**44**(19):e147–e. doi: 10.1093/nar/gkw654.

61. Jin JJ, Yu WB, Yang JB, et al. GetOrganelle: a fast and versatile toolkit for accurate de novo assembly of organelle genomes. *Genome Biol*. 2020;**21**(1):241. doi: 10.1186/s13059-020-02154-5.

62. Wick RR, Schultz M B, Zobel J, et. al. Bandage: interactive visualization of de novo genome assemblies. *Bioinformatics*. 2015; **31** (20): 3350–3352. doi: org/10.1093/bioinformatics/btv383.

63. Dudchenko O, Batra SS, Omer AD, et al. De novo assembly of the *Aedes aegypti* genome using Hi-C yields chromosome-length scaffolds. *Science*. 2017;**356**(6333):92–5. doi: 10.1126/science.aal3327.

64. Durand NC, Shamim MS, Machol I, et al. Juicer provides a One-Click system for analyzing loop-resolution Hi-C experiments. *Cell Syst*. 2016;**3**(1):95–8. doi:

10.1016/j.cels.2016.07.002.

65. Durand NC, Robinson JT, Shamim MS, et al. Juicebox provides a visualization system for Hi-C contact maps with unlimited zoom. *Cell Syst.* 2016;**3**(1):99–101. doi: 10.1016/j.cels.2015.07.012.

66. Hu J, Fan JP, Sun ZY, et al. NextPolish: a fast and efficient genome polishing tool for long-read assembly. *Bioinformatics*. 2019;**36**(7):2253–5. doi: 10.1093/bioinformatics/btz891.

67. Xu GC, Xu TJ, Zhu R, et al. LR_Gapcloser: a tiling path-based gap closer that uses long reads to complete genome assembly. *GigaScience*. 2018;**8**(1):giy157. doi: 10.1093/gigascience/giy157.

68. Pryszcz LP and Gabaldón T. Redundans: an assembly pipeline for highly heterozygous genomes. *Nucleic Acids Res*. 2016;**44**(12):e113. doi: 10.1093/nar/gkw294.

69. NT database. NCBI. 2020. https://ftp.ncbi.nlm.nih.gov/blast/db/

70. Simão FA, Waterhouse RM, Ioannidis P, et al. BUSCO: assessing genome assembly and annotation completeness with single-copy orthologs. *Bioinformatics*. 2015;**31**(19):3210–2. doi: 10.1093/bioinformatics/btv351.

71. Li H. Aligning sequence reads, clone sequences and assembly contigs with BWA-MEM. *ArXiv.* 2013:1303.3997. doi: 10.48550/arXiv.1303.3997.

72. Ou SJ and Jiang N. LTR_retriever: a highly accurate and sensitive program for identification of long terminal repeat retrotransposons. *Plant Physiol.* 2018;**176**(2):1410–22. doi: 10.1104/pp.17.01310.73.

73. Danecek P, Bonfield JK, Liddle J, et al. Twelve years of SAMtools and BCFtools. Gigacience. 2021,10(2):giab008. doi: 10.1093/gigascience/giab008.

74. Li Y. IsoSeq3 - Scalable De Novo Isoform Discovery from Single-Molecule PacBio Reads. GitHub. 2018. https://github.com/ylipacbio/IsoSeq3

75. Haas BJ, Delcher AL, Mount SM, et al. Improving the *Arabidopsis* genome annotation using maximal transcript alignment assemblies. *Nucleic Acids Res*. 2003;**31**(19):5654–66. doi: 10.1093/nar/gkg770.

76. Stanke M, Diekhans M, Baertsch R, et al. Using native and syntenically mapped cDNA alignments to improve *de novo* gene finding. *Bioinformatics*. 2008;**24**(5):637–44. doi: 10.1093/bioinformatics/btn013.

77. Chaw SM, Liu YC, Wu YW, et al. Stout camphor tree genome fills gaps in understanding of flowering plant genome evolution. *Nat Plants.* 2019;**5**(1):63–73. doi: 10.1038/s41477-018-0337-0.

78. Soltis DE and Soltis PS. Nuclear genomes of two magnoliids. *Nat Plants.* 2019;**5**(1):6–7. doi: 10.1038/s41477-018-0344-1.

79. Hu LS, Xu ZP, Wang MJ, et al. The chromosome-scale reference genome of black pepper provides insight into piperine biosynthesis. *Nat Commun*. 2019;**10**(1):4702. doi: 10.1038/s41467-019-12607-6.

80. Albert VA, Barbazuk WB, dePamphilis CW, et al. The *Amborella* genome and the evolution of flowering plants. *Science.* 2013;**342**(6165):1241089. doi: 10.1126/science.1241089.

81. The Arabidopsis Genome Initiative. Analysis of the genome sequence of the flowering plant *Arabidopsis thaliana*. *Nature*. 2000;**408**(6814):796–815. doi:10.1038/35048692.

82. Cantarel BL, Korf I, Robb SM, et al. MAKER: an easy-to-use annotation pipeline designed for emerging model organism genomes. *Genome Res*. 2008;**18**(1):188–96. doi:

10.1101/gr.6743907.

83. Slater GSC and Birney E. Automated generation of heuristics for biological sequence comparison. *Bmc Bioinformatics*. 2005;**6**(1):31. doi: 10.1186/1471-2105-6-31.

84. Seemann T. Barrnap: Bacterial ribosomal RNA predictor. GitHub. 2018. https://github.com/tseemann/barrnap

85. Lowe TM and Eddy SR. tRNAscan-SE: a program for improved detection of transfer RNA genes in genomic sequence. *Nucleic Acids Res.* 1997;**25**(5):955–64. doi: 10.1093/nar/25.5.955.

86. Huerta-Cepas J, Forslund K, Coelho LP, et al. Fast genome-wide functional annotation through orthology assignment by eggNOG-Mapper. *Mol Biol Evol.* 2017;**34**(8):2115–22. doi: 10.1093/molbev/msx148.

87. Buchfink B, Xie C and Huson DH. Fast and sensitive protein alignment using DIAMOND. *Nat Methods.* 2015;**12**(1):59–60. doi:10.1038/nmeth.3176.

88. Jones P, Binns D, Chang H-Y, et al. InterProScan5: genome-scale protein function classification. *Bioinformatics.* 2014;**30**(9):1236–40. doi: 10.1093/bioinformatics/btu031.

89. Emms DM and Kelly S. OrthoFinder: phylogenetic orthology inference for comparative genomics. *Genome Biol.* 2019;**20**(1):238. doi: 10.1186/s13059-019-1832-y.

90. Nguyen L-T, Schmidt HA, von Haeseler A, et al. IQ-TREE: a fast and effective stochastic algorithm for estimating maximum-likelihood phylogenies. *Mol Biol Evol.* 2015;**32**(1):268–74. doi: 10.1093/molbev/msu300.

91. Yang ZH. PAML 4: phylogenetic analysis by maximum likelihood. *Mol Biol Evol.* 2007;**24**(8):1586–91. doi: 10.1093/molbev/msm088.

92. Li HT, Yi TS, Gao LM, et al. Origin of angiosperms and the puzzle of the Jurassic gap. *Nat Plants*. 2019;**5**(5):461–70. doi: 10.1038/s41477-019-0421-0.

93. Magallón S, Gómez-Acevedo S, Sánchez-Reyes LL, et al. A metacalibrated time-tree documents the early rise of flowering plant phylogenetic diversity. *New Phytol.* 2015;**207**(2):437–53. doi: 10.1111/nph.13264.

94. Hahn MW, De Bie T, Stajich JE, et al. Estimating the tempo and mode of gene family evolution from comparative genomic data. *Genome Res.* 2005;**15**(8):1153–60. doi: 10.1101/gr.3567505.

95. Wang YP, Tang HB, DeBarry JD, et al. MCScanX: a toolkit for detection and evolutionary analysis of gene synteny and collinearity. *Nucleic Acids Res*. 2012;**40**(7):e49. doi: 10.1093/nar/gkr1293.

96. Edgar RC. MUSCLE: multiple sequence alignment with high accuracy and high throughput. *Nucleic Acids Res.* 2004;**32**(5):1792–7. doi: 10.1093/nar/gkh340.

97. Suyama M, Torrents D and Bork P. PAL2NAL: robust conversion of protein sequence alignments into the corresponding codon alignments. *Nucleic Acids Res.* 2006;**34**(suppl_2):609–12. doi: 10.1093/nar/gkl315.

98. Yang ZH and Nielsen R. Estimating synonymous and nonsynonymous substitution rates under realistic evolutionary models. *Mol Biol Evol*. 2000;**17**(1):32–43. doi: 10.1093/oxfordjournals.molbev.a026236.

99. Zhang Z, Li J, Zhao XQ, et al. KaKs_Calculator: calculating Ka and Ks through model selection and model averaging. *Genom Proteom Bioinf.* 2006;**4**(4):259–63. doi: 10.1016/S1672-0229(07)60007-2.

100. Chen SF, Zhou YQ, Chen YR, et al. Fastp: an ultra-fast all-in-one FASTQ preprocessor. *Bioinformatics*. 2018;**34**(17):884–90. doi: 10.1093/bioinformatics/bty560.

101. Garrison E and Marth G. Haplotype-based variant detection from short-read sequencing. *ArXiv*. 2012:1207.3907. doi: 10.48550/arXiv.1207.3907.

102. Danecek P, Auton A, Abecasis G, et al. The variant call format and VCFtools. *Bioinformatics*. 2011;**27**(15):2156–8. doi: 10.1093/bioinformatics/btr330.

103. Korneliussen TS, Albrechtsen A and Nielsen R. ANGSD: analysis of next generation sequencing data. *Bmc Bioinformatics*. 2014;**15**(1):356. doi: 10.1186/s12859-014-0356-4.

104. Watterson GA. On the number of segregating sites in genetical models without recombination. *Theor Popul Biol*. 1975;**7**(2):256–76. doi: 10.1016/0040-5809(75)90020-9.

105. Tajima F. Statistical method for testing the neutral mutation hypothesis by DNA polymorphism. *Genetics*. 1989;**123**(3):585–95. doi: 10.1093/genetics/123.3.585.

106. Fu YX and Li WH. Statistical tests of neutrality of mutations. *Genetics*. 1993;**133**(3):693–709. doi: 10.1093/genetics/133.3.693.

107. Purcell S, Neale B, Todd-Brown K, et al. PLINK: a tool set for whole-genome association and population-based linkage analyses. *Am J Hum Genet*. 2007;**81**(3):559–75. doi: 10.1086/519795.

108. Alexander DH, Novembre J and Lange K. Fast model-based estimation of ancestry in unrelated individuals. *Genome Res*. 2009;**19**(9):1655–64. doi: 10.1101/gr.094052.109.

109. Hanson-Smith V, Kolaczkowski B, Thornton JW. Robustness of Ancestral Sequence Reconstruction to Phylogenetic Uncertainty. *Mol Biol Evol*. 2010;**27** (9):1988–1999. Doi: org/10.1093/molbev/msq081.

110. Cristofari R, Bertorelle G, Ancel A, et al. Full circumpolar migration ensures evolutionary unity in the Emperor penguin. *Nat Commun*. 2016;**7**:11842. doi: org/10.1038/ncomms11842.

111. Salojärvi J, Smolander OP, Nieminen K. et al. Genome sequencing and population genomic analyses provide insights into the adaptive landscape of silver birch. *Nat Genet*. 2017;**49**:904–912. doi: org/10.1038/ng.3862.

112. Fukushima K, Pollock DD. Detecting macroevolutionary genotype–phenotype associations using error-corrected rates of protein convergence. *Nat Ecol Evol*. 2023;**7**: 155–170. doi: org/10.1038/s41559-022-01932-7.

113. Liu XM and Fu YX. Stairway Plot 2: demographic history inference with folded SNP frequency spectra. *Genome Biol*. 2020;**21**(1):280. doi: 10.1186/s13059-020-02196-9.

114. Li H and Durbin R. Inference of human population history from individual whole-genome sequences. *Nature*. 2011;**475**(7357):493–6. doi: 10.1038/nature10231.

115. Do C, Waples RS, Peel D, et al. NeEstimator v2: re-implementation of software for the estimation of contemporary effective population size (Ne) from genetic data. *Mol Ecol Resour.* 2014;**14**(1):209–14. doi: 10.1111/1755-0998.12157.

116. Sim N-L, Kumar P, Hu J, et al. SIFT web server: predicting effects of amino acid substitutions on proteins. *Nucleic Acids Res*. 2012;**40**(W1):452–7. doi: 10.1093/nar/gks539.

117. Boeckmann B, Bairoch A, Apweiler R, et al. The SWISS-PROT protein knowledgebase and its supplement TrEMBL in 2003. *Nucleic Acids Res*. 2003;**31**(1):365–70. doi: 10.1093/nar/gkg095.

118. Bortoluzzi C, Bosse M, Derks MFL, et al. The type of bottleneck matters: Insights into the

deleterious variation landscape of small managed populations. *Evol Appl.* 2020;**13**(2):330–41. doi: 10.1111/eva.12872.

119. Kirin M, McQuillan R, Franklin CS, et al. Genomic Runs of Homozygosity record population history and consanguinity. *PLoS One*. 2010;**5**(11):e13996. doi: 10.1371/journal.pone.0013996.

120. Ma YP, Wariss HM, Liao RL, et al. Genome-wide analysis of butterfly bush (*Buddleja alternifolia*) in three uplands provides insights into biogeography, demography and speciation. *New Phytol.* 2021;**232**(3):1463–76. doi: 10.1111/nph.17637.

121. Ma YP, Liu DT, Wariss HM, et al. Demographic history and identification of threats revealed by population genomic analysis provide insights into conservation for an endangered maple. *Mol Ecol.* 2022;**31**(3):767–79. doi: 10.1111/mec.16289.

122. Chen YC, Li Z, Zhao YX, et al. The *Litsea* genome and the evolution of the laurel family. *Nat Commun.* 2020;**11**(1):1675. doi: 10.1038/s41467-020-15493-5.

123. Chen SP, Sun WH, Xiong YF, et al. The *Phoebe* genome sheds light on the evolution of magnoliids. *Hortic Res*. 2020;**7**:146. doi: 10.1038/s41438-020-00368-z.

124. Guo X, Fang DM, Sahu SK, et al. *Chloranthus* genome provides insights into the early diversification of angiosperms. *Nat Commun*. 2021;**12**(1):6930. doi: 10.1038/s41467-021-26922-4.

125. Qin LY, Hu YH, Wang JP, et al. Insights into angiosperm evolution, floral development and chemical biosynthesis from the *Aristolochia fimbriata* genome. *Nat Plants.* 2021;**7**(9):1239–53. doi: 10.1038/s41477-021-00990-2.

126. Reed DH and Frankham R. Correlation between fitness and genetic diversity. *Conserv Biol.*

2003;**17**(1):230–7. doi: 10.1046/j.1523-1739.2003.01236.x.

127. Lessio F, Pisa CG, Picciau L, et al. An immunomarking method to investigate the flight distance of the Japanese beetle. *Entomol Gen*. 2022;**42**(1):45–56. doi: 10.1127/entomologia/2021/1117.

128. De Kort H, Prunier JG, Ducatez S, et al. Life history, climate and biogeography interactively affect worldwide genetic diversity of plant and animal populations. *Nat Commun.* 2021;**12**(1):516. doi: 10.1038/s41467-021-20958-2.

129. Gamba D and Muchhala N. Global patterns of population genetic differentiation in seed plants. *Mol Ecol.* 2020;**29**(18):3413–28. doi: 10.1111/mec.15575.

130. Li R and Yue J. A phylogenetic perspective on the evolutionary processes of floristic assemblages within a biodiversity hotspot in eastern Asia. *J Syst Evol.* 2020;**58**(4):413–22. doi: 10.1111/jse.12539.

131. Zhang KY. A preliminary study on the climatic characteristic and the formation factors in southern Yunnan. *Acta Meteorol Sin.* 1963;**33**(2):218–230.

132. Qian H and Ricklefs RE. Diversity of temperate plants in east Asia. *Nature*. 2001;**413**(6852):130–. doi: 10.1038/35093169.

133. Yang FM, Cai L, Dao ZL, et al. Genomic data reveals population genetic and demographic history of *Magnolia fistulosa* (Magnoliaceae), a Plant Species with Extremely Small Populations in Yunnan Province, China. *Front Plant Sci.* 2022;**13**:811312. doi: 10.3389/fpls.2022.811312.

134. Clark PU, Archer D, Pollard D, et al. The middle Pleistocene transition: characteristics, mechanisms, and implications for long-term changes in atmospheric pCO2. *Quat Sci Rev.*

2006;**25**(23):3150–84. doi: 10.1016/j.quascirev.2006.07.008.

135. Sun YB and An ZS. Late Pliocene-Pleistocene changes in mass accumulation rates of eolian deposits on the central Chinese Loess Plateau. *J Geophys Res-Atmos.* 2005;**110**(D23):D23101. doi: 10.1029/2005JD006064.

136. Clark PU, Dyke AS, Shakun JD, et al. The Last Glacial Maximum. *Science.* 2009;**325**(5941):710–4. doi: 10.1126/science.1172873.

137. Segelbacher G, Höglund J, Storch I. From connectivity to isolation: genetic consequences of population fragmentation in capercaillie across Europe. *Mol Ecol*. 2003,**12**(7): 1773–1780. doi: org/10.1046/j.1365-294X.2003.01873.x.

138. Zhao X, Ma Y, Sun W, et al. High genetic diversity and low differentiation of *Michelia coriacea* (Magnoliaceae), a critically endangered endemic in southeast Yunnan, China. *Int J Mol Sci*. **13**(4): 4396–4411. doi: 10.3390/ijms13044396

139. Khan A, Patel K, Shukla H, et al. Genomic evidence for inbreeding depression and purging of deleterious genetic variation in Indian tigers. *Proc Natl Acad Sci.* 2021;**118**(49):e2023018118. doi: 10.1073/pnas.2023018118.

140. Palsbøll PJ, Bérubé M and Allendorf FW. Identification of management units using population genetic data. *Trends Ecol Evol.* 2007;**22**(1):11–6. doi: 10.1016/j.tree.2006.09.003.

141. Deng YW, Liu TT, Xie YQ, et al. High genetic diversity and low differentiation in *Michelia shiluensis*, an Endangered magnolia species in South China. *Forests.* 2020;**11**(4):469. doi: 10.3390/f11040469.

142. Yu HH, Yang ZL, Sun B, et al. Genetic diversity and relationship of endangered plant

Magnolia officinalis (Magnoliaceae) assessed with ISSR polymorphisms. *Biochem Syst Ecol.* 2011;**39**(2):71–8. doi: 10.1016/j.bse.2010.12.003.

143. Zhao XF, Ma YP, Sun WB, et al. High genetic diversity and low differentiation of *Michelia coriacea* (Magnoliaceae), a critically endangered endemic in southeast Yunnan, China. *Int J Mol Sci.* 2012;**13**(4):4396–411. doi: 10.3390/ijms13044396.

144. Cai L, Liu D, Yang F, et al. Supporting data for "The chromosome-scale genome of Magnolia sinica (Magnoliaceae) provides insights into the conservation of plant species with extremely small populations (PSESP)". GigaScience Database. 2023. http://dx.doi.org/10.5524/102474.

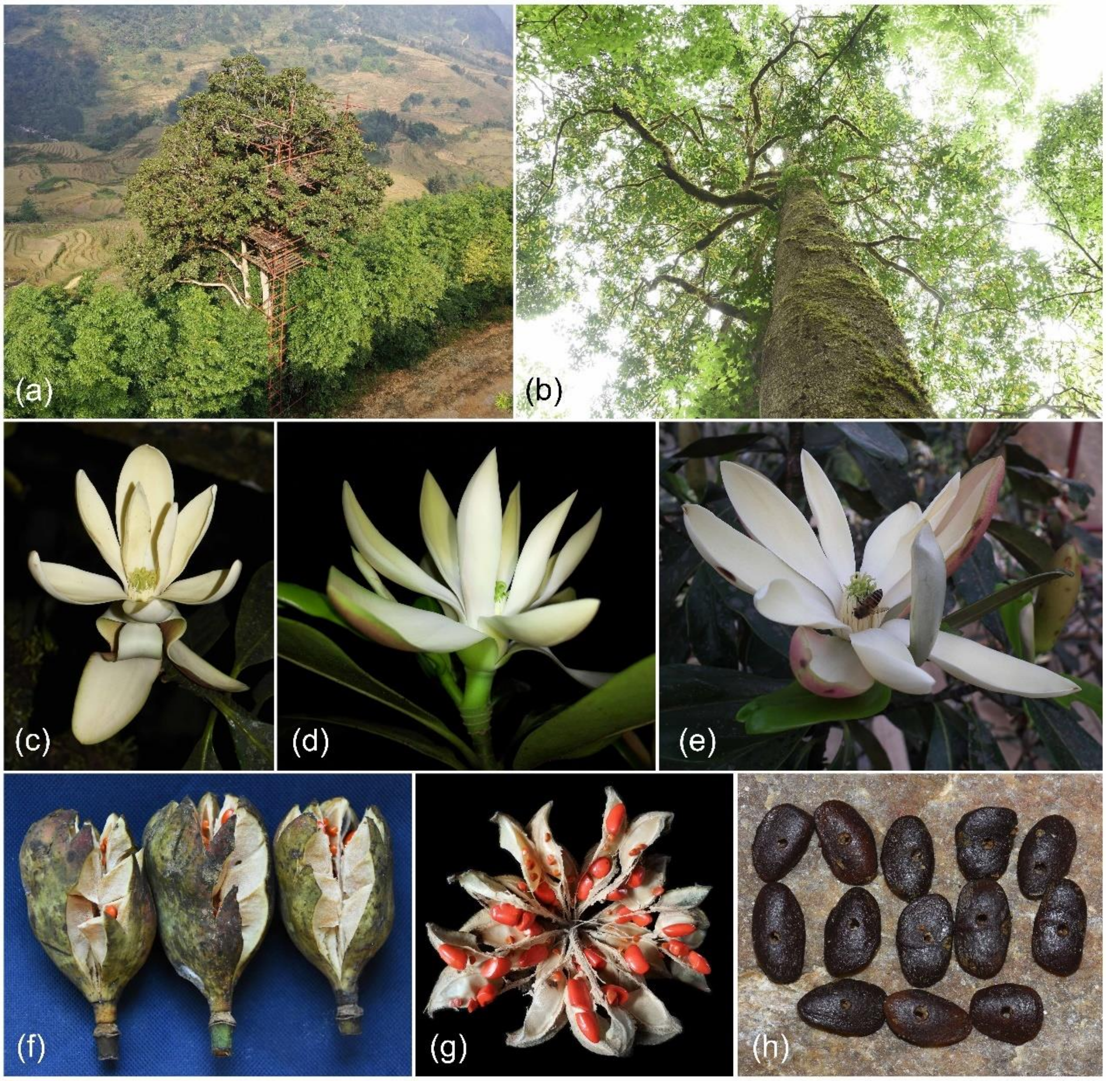

FIGURE 1 Habitat and morphological characters of *Magnolia sinica*. (a) Habitat. (b) Habit. (c-e) Flowers. (f) Fruits. (g) Fruit completely opened. (h) Seeds without testa.

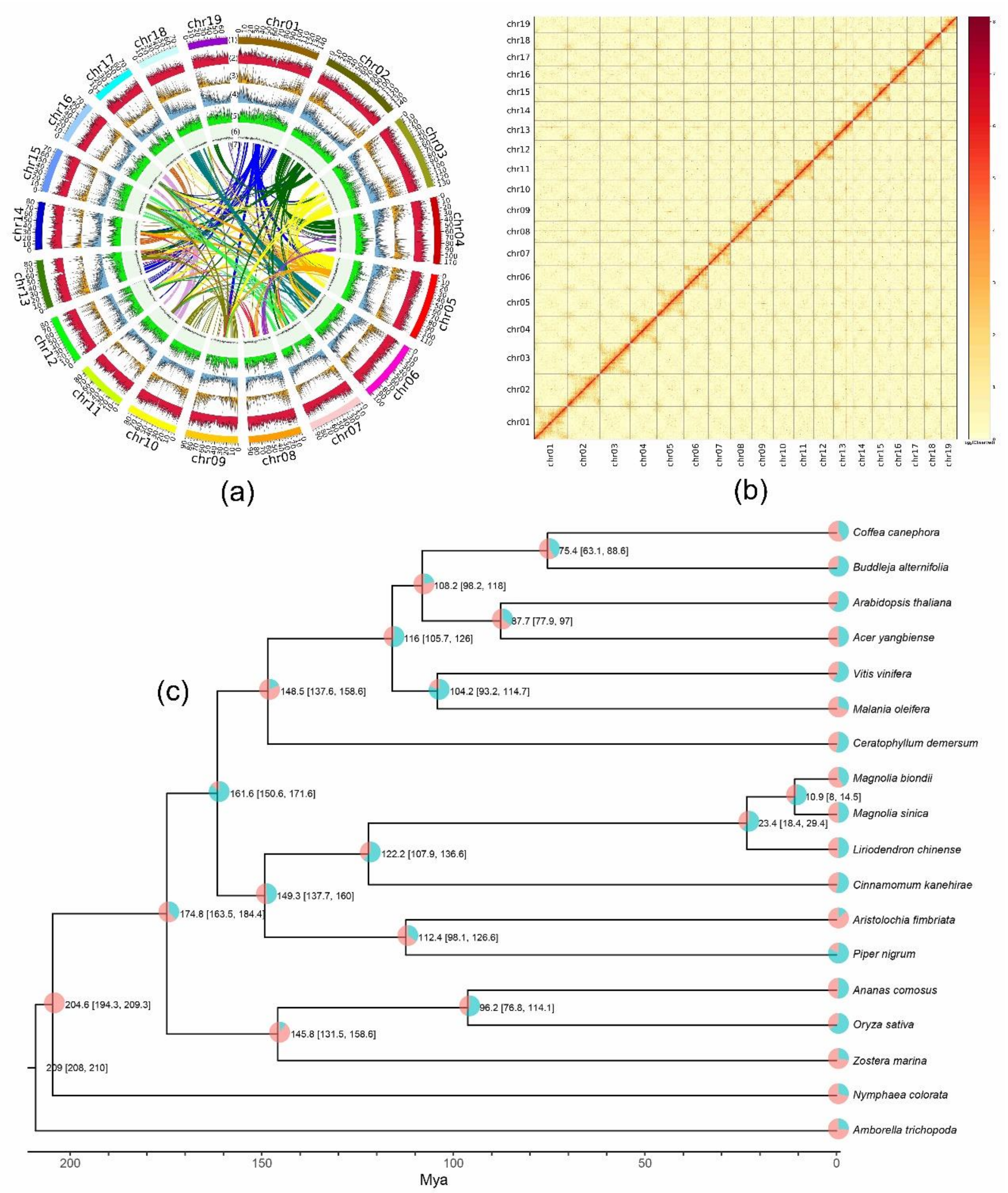

FIGURE 2 Genomic character and genome evolution of *Magnolia sinica*. **(a)** The genome features across 19 chromosomes of *M. sinica*. (1) 19 pseudochromosomes. (2) Class I transposable element (TE) density (including long terminal repeats; [LTRs], long and short interspersed nuclear elements).

(3) Class II TE (DNA and Heliron) density. (4) Coding gene (messenger RNA) density. (5) The density of single-nucleotide polymorphism (SNP) loci. (6) GC content. (7) collinear blocks. **(b)** Hi-C interaction heatmap for the *M. sinica* genome showing interactions among 19 chromosomes. **(c)** The phylogenetic tree of 18 species showing the proportions of the gene families that contracted and expanded (pink: contracted; blue-green: expanded; Values at the nodes represent the time of differentiation and 95 % CI).

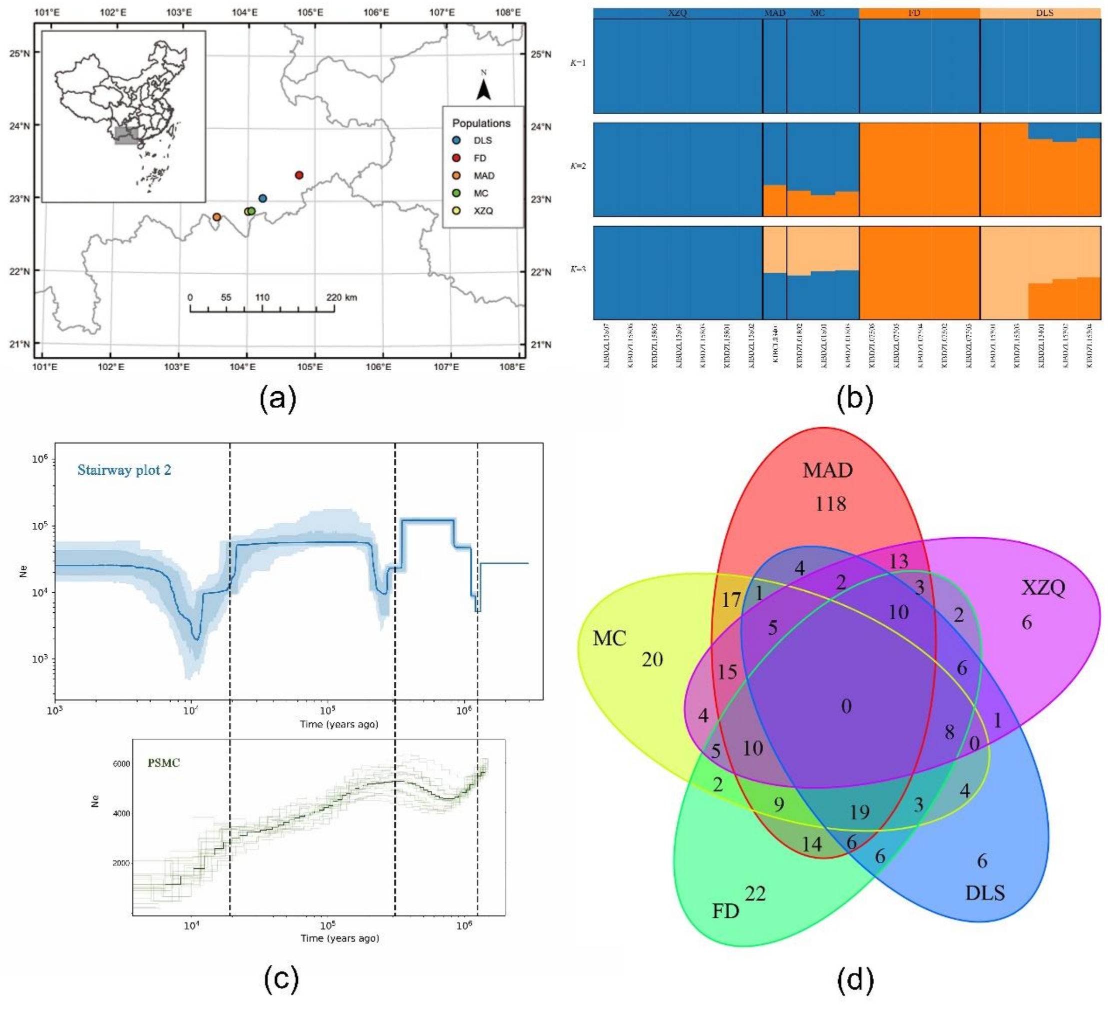

FIGURE 3 Distribution map, population structure, demographic history and Venn diagram of *Magnolia sinica*. **(a)** Distribution map showing the locations of the five subpopulations in Yunnan. **(b)** Plots of the population structure of 21 *Magnolia sinica* individuals from five provenances for different numbers of subpopulations (K), from K = 1 to K = 3. **(c)** The demographic history of *M.*

sinica inferred in Stairway plot2 (with a generation time of 30 years, and a mutation rate of 1.2e-7. The 95% confidence interval for the estimated effective population size is shown in a light blue color) and PSMC plot (with 21 samples of *M. sinica*, with the blue line being the average effective population size). **(d)** Venn diagram showing distribution of shared and unique deleterious mutations among the five subpopulations of *M. sinica.*

MAD, Maandi population in Jinping County; FD, Fadou population in Xichou County; XZQ, Xinzhaiqing population in Maguan County; DLS, Dalishu population in Maguan County; MC, Miechang population in Maguan County.

Table 1 Statistics of *Magnolia sinica* genome assembly and annotation

**Parameter**	***Magnolia sinica***
Total assembly size (bp)	1,839,595,854
GC content (%)	40.18
Total number of contigs	203
Maximum contig length (bp)	96,921,630
Minimum contig length (bp)	5,003
Contig N50 (bp)	44,871,976
Contig N90 (bp)	10,133,504
Total number of scaffolds	130
Maximum scaffold length (bp)	141,926,363
Minimum scaffold length (bp)	5,003
Scaffold N50 (bp)	92,164,922
Scaffold N90 (bp)	73,752,208
Gap number	73
Complete BUSCOs (%)	97.9
Complete single-copy BUSCOs (%)	94.3

Complete and duplicated BUSCOs (%)	3.6
Fragmented BUSCOs (%)	0.5
Missing BUSCOs (%)	1.6
Gene number	44,713
Protein-coding genes	43,473
LAI value	10.3

 Click here to access/download;Table;Table 1 20230728.docx

Table 1 Statistics of *Magnolia sinica* genome assembly and annotation

**Parameter**	***Magnolia sinica***
Total assembly size (bp)	1,839,595,854
GC content (%)	40.18
Total number of contigs	203
Maximum contig length (bp)	96,921,630
Minimum contig length (bp)	5,003
Contig N50 (bp)	44,871,976
Contig N90 (bp)	10,133,504
Total number of scaffolds	130
Maximum scaffold length (bp)	141,926,363
Minimum scaffold length (bp)	5,003
Scaffold N50 (bp)	92,164,922
Scaffold N90 (bp)	73,752,208
Gap number	73
Complete BUSCOs (%)	97.9
Complete single-copy BUSCOs (%)	94.3
Complete and duplicated BUSCOs (%)	3.6
Fragmented BUSCOs (%)	0.5
Missing BUSCOs (%)	1.6
Gene number	44,713
Protein-coding genes	43,473
LAI value	10.3

Figure 1

Click here to access/download;Figure;FIGURE 1.pdf

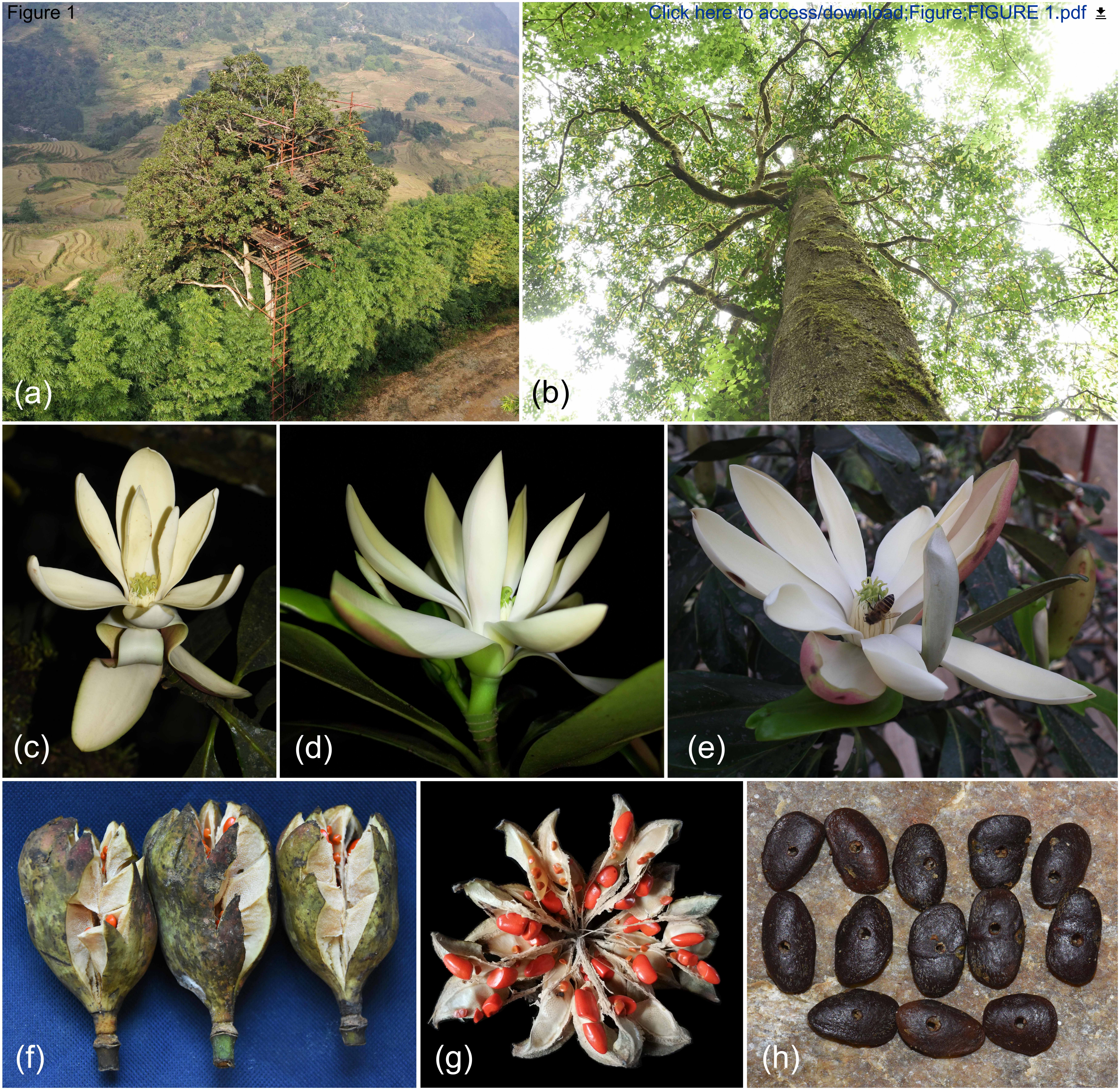

Figure 3

Click here to access/download;Figure;FIGURE 3.pdf

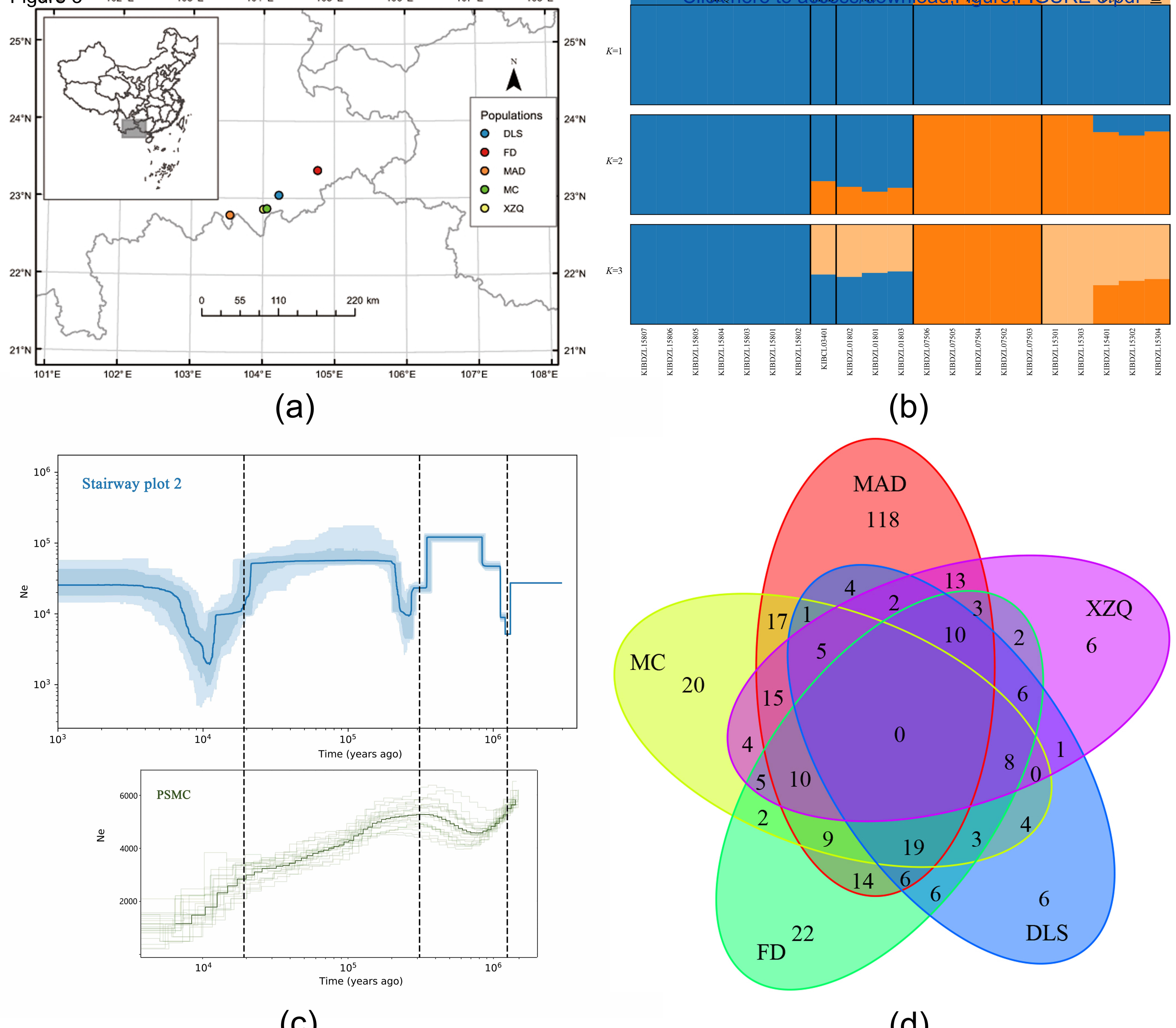

Figure 2

Click here to access/download;Figure;FIGURE 2.pdf

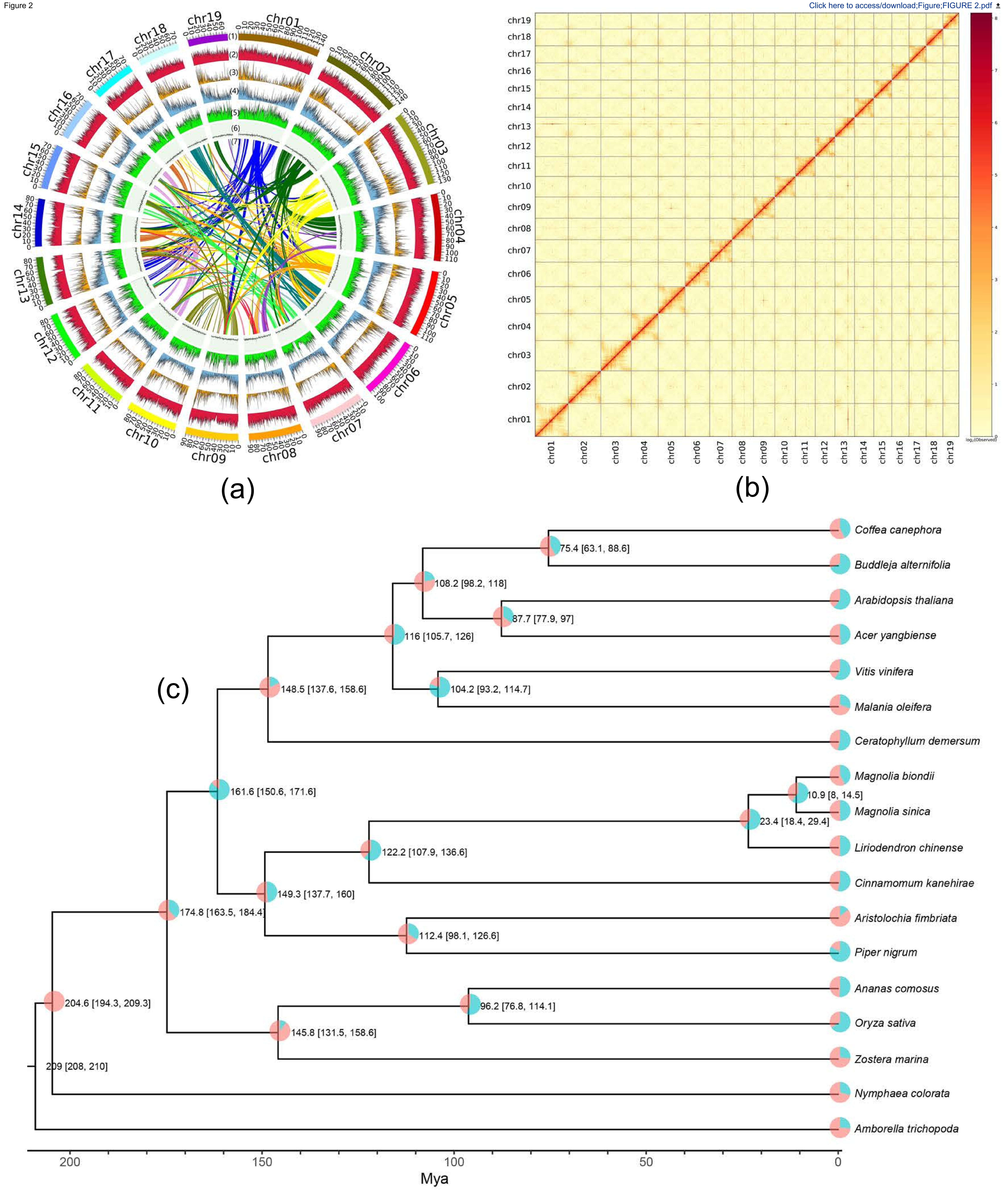

Click here to access/download
Supplementary Material
Supplementary file-Figures 20230728.docx

Click here to access/download
Supplementary Material
Supplementary file-Tables 20230728.xlsx

Response Click here to access/download;Personal Cover;Comments to the editor and reviewers 20230728.docx

Comments to the editor and reviewers

Dear the Editor of **GigaScience**,

Thank you very much for editing this manuscript entitled "*The chromosome-scale genome of Magnolia sinica (Magnoliaceae) provides insights into the conservation of plant species with extremely small populations (PSESP)*" and making suggestions. We are also very grateful for the efforts of the two reviewers. We have revised the manuscript carefully according to their comments and have made responses listed below.

We have accepted most of the comments from the two reviewers, made revisions to the errors that occurred, added some relevant analyses, and have responded to and explained a small portion of the questions. **1)** We have added discussions of the coexistence of high genetic diversity and low genetic differentiation to the manuscript in the DISCUSSION part. **2)** We have added relevant supplementary figures with bootstrap values in the phylogenetic tree (Figure S5). **3)** We have added parameters and we have added KAT analysis. **4)** We have released all the data produced to date (https://www.ncbi.nlm.nih.gov/bioproject/PRJNA774088). **5)** We have explained why the whole genome sequencing and transcriptome sequencing (RNA-seq) analyses did not use material from the same individual, and also explained why only 21 individuals were re sequenced. Please review the specific revisions and responses.

We resubmit the revised manuscript and we hope this version is now suitable for the publication in **GigaScience**. If you have any further questions or requirements, please do not hesitate to contact the corresponding author (MYP).

Yours sincerely,

Yongpeng Ma (corresponding authors on behalf of all authors).

26th JULY 2023

Reviewer #1: In this paper, authors reported the first genome of a critically endangered species Magnolia sinica. This large tree is widely known as "giant pandas in plants" due to its extremely rare individuals in wild, thus is under the first-class state protection in China. Here, authors obtained a high-quality chromosome-level genome assembly via combining Illumina, PacBio and Hi-C sequencing data. Authors mainly focus on the population resequencing, showing a high genetic diversity of M. sinica population but a low genetic differentiation among subpopulations. Authors provide some explanations for each result. I wonder if author can discuss the potential connections between these two observed phenomenons. In addition, authors detected many deleterious mutations which were mostly related to lipids. Authors didn't mention this result in the DISCUSSION part. Are these deleterious mutations related to lipids results of or reasons for the endangered status of this species? Authors may provide further discussions or even conclusive evidences to clearly elucidate point of view this issue.

Response: Thank you for your suggestion. We now added discussions of coexistence of high genetic diversity and low genetic differentiation to the manuscript in the DISCUSSION part as below:

"M. sinica has a pollinator-dependent outcrossing mating system, which may contribute to its high genetic diversity; while high gene flow among populations may maintain links between populations of this species, and may contribute to its low genetic differentiation. The recent reduction in population size due to anthropogenic activities has led to isolation of the populations, leading to the high genetic diversity and low genetic differentiation now observed in the fragmented populations of this endangered tree species. Similar patterns have been reported in Michelia coriacea, another species in the Magnoliaceae [131]."

Regarding the deleterious mutations related to lipids, we could not conclude whether they were the results of or the reasons for the endangered status of *Magnolia sinica*, and we have therefore deleted the parts of the GO and KEGG anotations and enrichment analysis regarding deleterious mutations from the manuscript.

Reference

Zhao X, Ma Y, Sun W, et al. (2012) High genetic diversity and low differentiation of *Michelia coriacea* (Magnoliaceae), a critically endangered endemic in southeast Yunnan, China. International Journal of Molecular Sciences, 13(4): 4396–4411.

Minor concerns:

1. Introduction part: authors should point out what's the major limitations of the current protection of Huagaimu. And how a reference genome helps to overcome such limitations.

Response: Thank you. We have added the first part in the manuscript. And, the second part was included in last paragraph of the introduction as below.

"Although a great deal of protection and research action has been carried out, the lack of natural regeneration and genetic rescue still limits the protection of *M. sinica*. Therefore, the formulation of genetic rescue strategies for *M. sinica* will benefit greatly from the exploration of harmful cumulative mutations, population historical dynamics and effective population size from the whole genome level.

Here, we report a high-quality chromosome-scale genome sequence of *Magnolia sinica*, and compare it with other relevant published genomic data. By exploring the evolution of the genome, as well as the genetic characteristics, demographic history and genetic load of *M. sinica*, we have identified genomic factors that may contribute to the threats to this species, and, on the basis of this, we propose further strategies for the conservation of *M. sinica*."

2. Magnolia sinica was first occurred in Line 79 in the main text and it should be written as M. sinica afterwards.

Response: Thank you. We have checked and revised this.

3. Line 206: "integrated annotated protein" should be "integrated annotated proteins".

Response: Thank you. We have revised this.

4. Line 222-224: References were needed here.

Response: Thank you. We have added relevant references.

5. Line 253: "θW" should be "θw".

Response: Thank you. We have revised this.

6. Fig. 2c, there shouldn't be a "_" within species name. And, bootstrap values should be indicated in the phylogenetic tree. In addition, Fig. 2 contained different results with no obvious connections. I do recommend to layout the content of this figure, focusing on one particular theme.

Response: Thank you. We now deleted the "_" within species name. We have added a relevant supplementary figure with the bootstrap values in the phylogenetic tree, please check **(Figure S5)**. Because of the large number of figures in the manuscript, we have tried to save space and have given the figures (genomic character and genome evolution), where related figures are merged into one plate and explanations are provided separately.

7. No title was found in Fig. 3. Authors should give a strong title that reflects the major finding of this figure.

Response: Thank you. We have added a title (Distribution map, population structure, demographic history and Venn diagram of *Magnolia sinica*) for this Figure 3.

Reviewer #2: This manuscript described the assembly and analyses of the chromosome-scale genome assembly for Magnolia sinica, an endangered Magnoliaceae species. Despite the authors provided a useful piece of work, it can still be greatly improved. In particular, it needs a thorough proofing to clarify many points in the Material & Methods section, as well as in results.

However, a major interrogation is the rational of resequencing only 21 M. sinica and 22 other Magnolia, while there is only 52 remaining M. sinica in the wild. I think it would have shown a much complete picture to generate data for all (known) individuals **in the species.**

Response: Thank you for your questions. In 2019, we only re-sequenced the materials that we had collected (21 samples). These materials included samples from all populations and covered the full range of the *Magnolia sinica* distribution, representing >40% of all *M. sinica* individuals. Because the collection of these

materials took a lot of money and time, considering the cost of re-collection and the expensive re-sequencing costs at the time, we were unable to collect material from more individuals. Furthermore, based on the preliminary analysis of our sequencing data, we found that there were no significant differences (such as genetic diversity or genetic structure) compared to previous population studies based on SSR (Chen 2017, in Chinese). Therefore, we only sequenced 21 individuals of *M. sinica* from that time.

The phylogenetic position of *M. sinica* has always been controversial, so we chose to sequence 22 samples from other eight *Magnolia* species. We have provided the relevant chloroplast tree **(attached figure 1 chloroplast tree)** and SNPs tree **(attached figure 2 SNP_tree)** as attachments at the bottom of this file.

I noticed several mistakes in the description of used data and methods. For example:

(1) line 21 the authors mentioned using Pacbio data for genome assembly, but from the Material & Methods, they used only ONT data to generate long reads for assembly

Response: We have revised this mistake.

(2) they mentioned a QiaGen kit that seems to not exist in Material & Methods line 149 they mentioned using Pilon to modifiy - correct? - Illumina reads; should be the opposite

Response: The reagent kit with product number 13323, Qiagen, is available. Genomic DNA kit (cat. no. 13323. Qiagen, Hilden, Germany). Please check: https://www.qiagen.com/us/products/discovery-and-translational-research/dna-rna-purification/dna-purification/genomic-dna/blood-and-cell-culture-dna-kits.

We have corrected the description of correcting with Illumina reads.

(3) Parameters used for pipelines are missing in several part of the manuscript

Also, the usually used metrics and quality assessment methods were not used here; I would appreciate to get a Merqury / KAT/ GenomeScope analysis in addition to the BUSCO and LAI.

Response: We have added parameters and a KAT analysis.

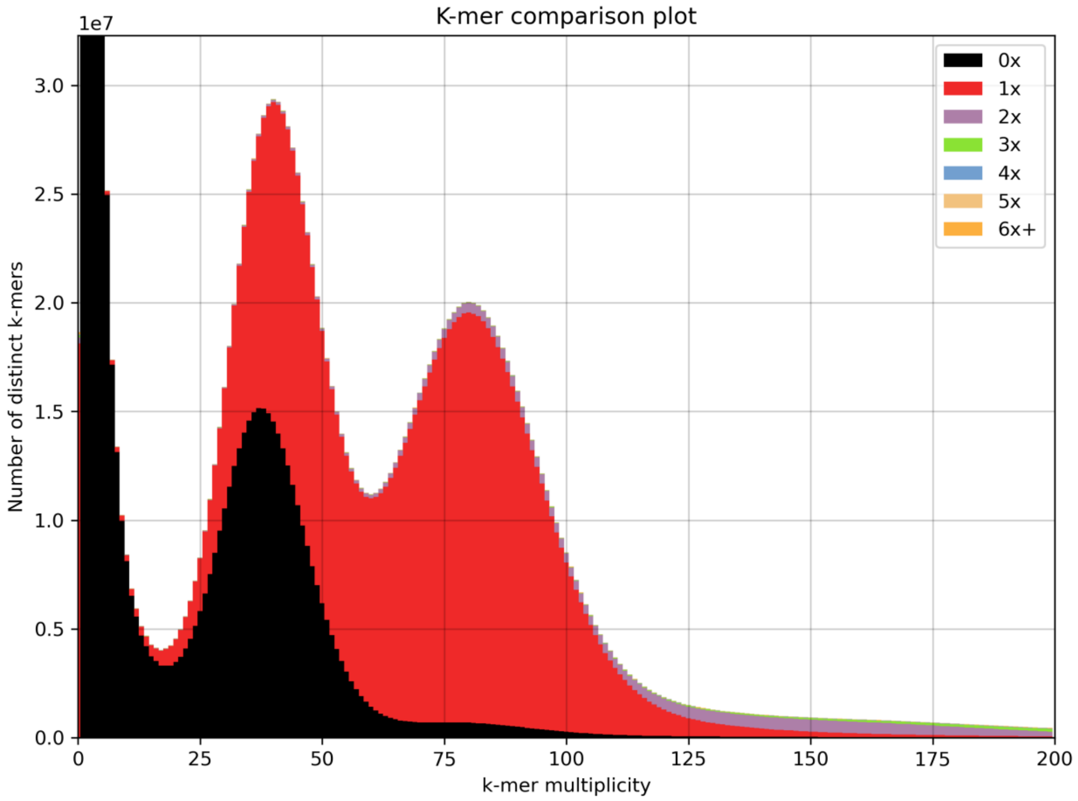

Also, I don't really understand why the authors performed RNAseq for annotation from a different individual, instead of using the same individual as for the genome assembly.

Response: Thank you. We understand your concern regarding this issue, unfortunately we faced some challenges during this project. In 2019, when we started sequencing, leaf samples were initially sent to a company in dry ice for genome sequencing. Later in 2020, when we collected multiple tissues for RNA-seq, it became very difficult to send samples rapidly in dry ice because of special policies (special periods of COVID-19). Therefore, for simplicity, we decided to directly send a living seedling (including leaf, stem, root tissues, but excluding other tissues such as flowers) and fresh fruits at room temperature (without dry ice) for RNA-seq. Therefore, the RNAseq and genome assembly analyses were conducted using different individuals. However, because we used the PacBio platform to sequence the full-length cDNA, the variations between individuals should have very limited negative effects on gene annotation. In fact, 99.5% PacBio CCS reads were mapped to the genome.

The ancestral sequence reconstruction part appeared quite weak with the method used, not taking into account the emergence of potentially large Structural Variations (SVs)

across the chromosomes during their evolutions. I would suggest, if the authors want to keep this part to use a more robust approach (e.g. based on Salse, 2021 approach)

Response: Thank you for your suggestion. We agree that the emergence of SV may influence the reconstruction of ancestral state. However, SV is difficult to detect from our short reseqencing reads. Here we used an empirical Bayesian method based on posterior probability of the sites to reconstruct ancestral sequence. This method can produce accurate reconstruction of the ancestral sequence (Hanson-Smith et al. 2010) and has been previously used to reconstruct the ancestral state in other works (Cristofari et al., 2016; Salojärvi et al., 2017; Ma et al., 2021; Fukushima et al., 2023). We apologize for not being able to find the article by "Salse, 2021". After explaining our method above, if it is necessary to use Salse's approach, could you please provide us more information about it and give us another chance to revise it?

References

Cristofari R, Bertorelle G, Ancel A, et al. Full circumpolar migration ensures evolutionary unity in the Emperor penguin. Nat Commun. 2016;7:11842. doi: org/10.1038/ncomms11842.

Fukushima K, Pollock DD. Detecting macroevolutionary genotype–phenotype associations using error-corrected rates of protein convergence. Nat Ecol Evol. 2023;7: 155–170. doi: org/10.1038/s41559-022-01932-7.

Hanson-Smith V, Kolaczkowski B, Thornton JW. Robustness of Ancestral Sequence Reconstruction to Phylogenetic Uncertainty. Mol Biol Evol. 2010;27 (9):1988–1999. Doi: org/10.1093/molbev/msq081.

Ma H, Liu YB, Liu DT, et al. Chromosome-level genome assembly and population genetic analysis of a critically endangered rhododendron provide insights into its conservation. Plant J. 2021;107(5):1533–45. doi: 10.1111/tpj.15399.

Salojärvi J, Smolander OP, Nieminen K. et al. Genome sequencing and population genomic analyses provide insights into the adaptive landscape of silver birch. Nat Genet. 2017;49:904–912. doi: org/10.1038/ng.3862.

The data accessibility is also questionable, as the authors mentioned the BioProject PRJNA774088, that is already cited by a published paper, but not accessible

Response: We apologize that the data were not released earlier. The data have now been completely released (https://www.ncbi.nlm.nih.gov/bioproject/PRJNA774088). A copy of the data can be found in China National Center for Bioinformation (https://ngdc.cncb.ac.cn/bioproject/browse/PRJCA015437).

Specific comments:

- Line 21 : Only ONT data were combined with short reads to assemble the genome ;

Response: Sorry, we have revised this mistake.

- Line 59 : please add the date when the database have been accessed ;

Response: Thank you. We have corrected this and added the access dates.

- Line 93-97 : this seems more adequate for a Data Notes than for a research article ;

Response: Thank you, this is indeed only a partial summary. Here, we not only reported the high-quality chromosome-scale genome sequence of *Magnolia sinica* and re-sequenced 21 samples of the same species and 22 samples from other species, but also investigated genome evolution, genome-wide diversity, and population structure of this species, inferred its demographic history, and estimated its genetic load and inbreeding level. We further discussed the possible reason for its high genetic diversity but low genetic differentiation, the climatic, tectonic and anthropogenic explanation of its demographic history, the likely genetic basis of the extremely small populations, and provided conservation measures based on our findings. We think it is worthy of a research article.

- Line 107 : dry ice temperature is -78.5°C

Response: We have revised this mistake.

- Line 118 : this kit does not exist (the reference number is for an other kit)

Response: We have revised this. The Genomic DNA kit (cat. no. 13323. Qiagen, Hilden, Germany) is available, and this kit can also extract genomic DNA from diverse materials. The kit was also used to extract plant DNA after treatment of CTAB.

- Line 121 : more details are needed for the library construction method. What was the DNA input ? any modification from the ONT protocol ? barcoded library or not ?

Response: The DNA input was total genomic DNA. The ONT protocol was not

modified, and the library was not barcoded.

- Line 124 : please choose the machine the library was run on (or precise which library was run on which machine) ; how many flowcells ?

Response: PromethION was used yielding 7 flowcells. This has been added to the manuscript.

- Line 126 : what fragment size for the Illumina library

Response: We have added insertion size of 300–500 bp.

- Line 130 : what was considered as "high molecular weight DNA" ?

Response: This refers to longer and more complete DNA with high “molecular weight”.

- Line 147: please precise what assembly strategies did you used (= assemblers ?)

Response: Thank you, we have added a descriptions of the assembly method.

- Line 148 : this reference is for the Celera assembler only, did you use it ?

Response: No. We have revised the text.

- Line 149 : short reads were used to correct long reads, not the opposite ;

Response: Thank you, this has been revised.

- Line 151 : how they were polished ?

Response: The method has been added.

- Line 151 : please described the parameters used in GetOrganelles to assemble both the mitochondrial genome and plastome

Response: The parameters have been added.

- Line 159 : "scaffolded" instead of "scattered" ?

Response: This has been revised as “un-anchored” meaning contigs that were not anchored onto chromosomes.

- Line 161 : what parameters for LR_Gapcloser and NextPolish ?

Response: The parameters have been added.

- Line 163 : Redundant (typo)

Response: It has been revised.

- Line 165 : what is the NT library ?

Response: The NT library is NT database from NCBI for BLAST (https://ftp.ncbi.nlm.nih.gov/blast/db/). We have revised this in the text for clarification.

- Line 167 : how low was a coverage considered ?

Response: We have revised this in the text.

- Line 172-183 : see above for addition of QC pipelines results

Response: We have added KAT analysis.

- Line 189 : how these two libraries were combined ?

Response: We concatenated the two libraries (fasta files) directly using the Linux command `cat`.

- Line 194 : Considering Magnoliaceae position in angiosperms, I think it could be useful to add at least one monocots in the annotation process (e.g. the wheat or maize, or rice genome)

Response: Thank you for your suggestion. We tested this by adding the wheat genome, and found only 551 new genes (1.3% more than before) predicted by the MAKER2 pipeline. We also tested it with the *Aristolochia fimbriata* (Piperales) genome as evidence, and 1419 genes (3.3% more) were newly identified. It appears that more protein evidences would certainly produce more genes, but considering the improvements (1.3-3.3% more genes) are quite limited and would not significantly affect our downstream conclusions regarding comparative and conservation genomics, we chose to not include the update in the revision.

- Line 201 : Augustus is usually used as an ab initio annotator ; please specify more in details how you used it the integrate previous annotations

Response: Yes, Augustus is an ab initio annotator, but it supports biological evidence (hint file from transcript and protein alignments) as input for better prediction. This step is integrated in the MAKER2 pipeline. We have revised the text for a clearer description.

- Line 217, 220, 222 : why there is a discrepancy between the single-copy gene numbers ?

Response: We used different cutoffs to allow for missing data. For the ASTRAL method, more genes are better with high ILS (incomplete lineage sorting) level, and missing data are more tolerated (References below), so we used more genes with higher missing rate (30%). For the IQTREE method, missing data are moderately tolerated, so we used the dataset with moderate missing rate (12.5%; the dataset was generated in

OrthoFinder2 to infer a species tree in its pipeline). MCMCtree uses only non-missing data by default, so we just included 1:1 orthologous single-copy genes (with none missing). Different dataset may provide cross-validations to reduce sampling bias. We have added detailed descriptions.

References:

Molloy E K, Warnow T. To Include or Not to Include: The Impact of Gene Filtering on Species Tree Estimation Methods [J]. Syst. Biol., 2017, 67 (2): 285–303 [http://doi.org/10.1093/sysbio/syx077]

Shekhar S, Roch S, Mirarab S. Species Tree Estimation Using ASTRAL: How Many Genes Are Enough? [J]. IEEE/ACM Transactions on Computational Biology and Bioinformatics, 2018, 15 (5): 1738–1747 [http://doi.org/10.1109/TCBB.2017.2757930]

- Line 235 : Why not using the 52 M. sinica individuals (see above) ?

Response: Thank you for your questions. In 2019, we only re-sequenced the materials that we had collected (21 samples). These materials included samples from all populations, and covered the full range of the *Magnolia sinica* distribution, representing >40% of all *M. sinica* individuals. Because the collection of these materials took a lot of money and time, considering the cost of re-collection and the expensive re-sequencing costs at the time, we were unable to collect material from more individuals. Furthermore, based on the preliminary analysis of our sequencing data, we found that there were no significant differences (such as genetic diversity or genetic structure) compared to previous population studies based on SSR (Chen 2017, in Chinese). Therefore, we only sequenced 21 individuals of *M. sinica* from that time.

- Line 241 : sequences with quality score <20 should not be found in the clean reads (from line 238)

Response: After filtering with fastp, the proportion of sequences with a quality score <20 decreases, however, there are still some bases with a quality score <20. Fastp trims reads using a sliding window, but did not trim all bases with a quality score <20. Thus,

we excluded the potentially retained bases with quality score <20 in downstream analysis (ANGSD and freebayes).

- Line 242 : considering a sequencing depth ranging from 8.8X to 12.6X for M. sinica (max 14.3X for other Magnolia), it seems unrealistic to remove sites with a mapping depth <100X

Response: The depth of sites refers to the sum of all samples, but not average depth across samples. The distribution of the depth of sites is as follows. The peak value is at 331x, so empirically the upper limit is set to 600x, about twice that of the peak, and the lower limit is about 1/3 of the peak. We have revised the text to make this clear.

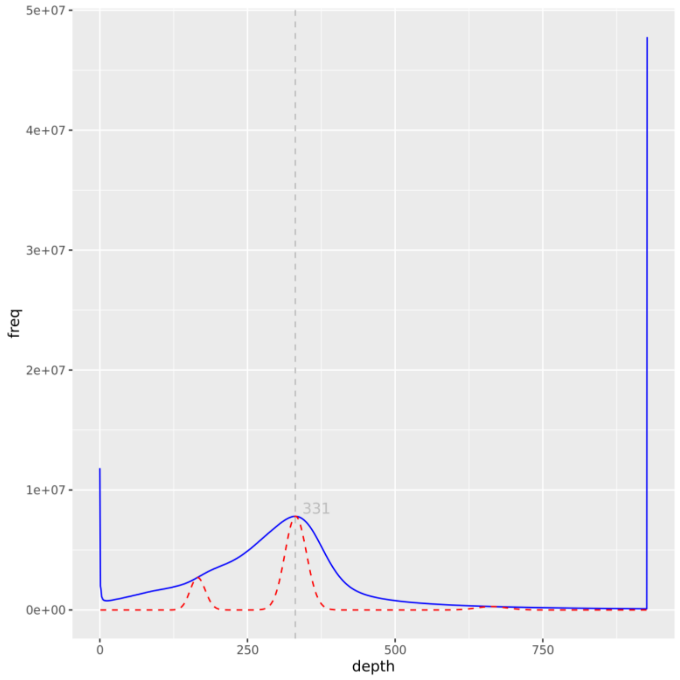

- Line 243 : please specify how these sites were retained

Response: We have described this in more detail in the paper.

- Line 248 : why the authors did not use the widely used 10% missing data threshold?

Response: Thank you for your question. We wanted to balance the threshold and the number of SNPs. Considering that there are many species, a stricter threshold would lead to fewer SNPs, which may be not have been sufficient for downstream analyses. In fact, the threshold of 20% or higher has also been used in previous studies (References below).

References:

Liu S, Zhang L, Sang Y et. al. Demographic History and Natural Selection Shape Patterns of Deleterious Mutation Load and Barriers to Introgression across Populus Genome [J]. Mol. Biol. Evol., 2022, 39 (2) [http://doi.org/10.1093/molbev/msac008]

Dai F, Zhuo X, Luo G et. al. Genomic Resequencing Unravels the Genetic Basis of Domestication, Expansion, and Trait Improvement in Morus Atropurpurea [J]. Adv. Sci., 2023 [http://doi.org/10.1002/advs.202300039]

Wang P, Zhou G, Jian J et. al. Whole-genome assembly and resequencing reveal genomic imprint and key genes of rapid domestication in narrow-leafed lupin [J]. Plant J., 2021, 105 (5): 1192–1210 [http://doi.org/10.1111/tpj.15100]

Ma Z, Zhang Y, Wu L et. al. High-quality genome assembly and resequencing of modern cotton cultivars provide resources for crop improvement [J]. Nat. Genet., 2021 [http://doi.org/10.1038/s41588-021-00910-2]

- Line 249 : due to both the relatively low number of indiviuals and the large part of the sampling made of other Magnolia species, such a classic MAF value would results in removing SNPs present in 1 or 2 samples, making them potentially diagnostic of a given species

Response: We did not aim to make diagnostic of a given species, so the species-specific SNPs were not necessary for our analyses. In the phylogenetic tree based on the filtered SNPs (attached figure 2 SNP_tree), each species has formed a separate monophyletic clade, suggesting that our filtering with the classic MAF value did not obscure the relationships among these species.

- Line 250 and following : Please described more in details, but concisely, how these different datasets are made, and how they are each useful (at least more useful than only one or two datasets)

Response: We apologized for the imprecise and incorrect descriptions. We have revised this and have also added an additional schematic diagram to the supplementary figures to illustrate it.

- Line 309 : please add the parameters used

Response: Thank you, we have added these.

- Line 319 : did the authors considered flow cytometry to get a (more) accurate estimate of the genome size ? Considering the patrimonial value of the species, it could be valuable.

Response: Thank you. At that time, the Genome size of *Magnolia sinica* was estimated by k-mer analysis of the Illumina sequencing data. This method is widely used and is sufficiently accurate, so we felt that we did not need to use an experimental method based on Flow Cytometry.

- Line 327 : Did the authors compared the LAI value obtained here with other Magnolia genome assemblies ?

Response: Thank you. We could not compare the relevant LAI values of several Magnolia species because of the other three genomic articles did not calculate this value.

- Line 335-336 : Please add values for gene annotations from transcriptomic, ab initio and similarity approaches separately, then indicate how many were supported, filtered and so on, with the final value.

Response: The MAKER annotation pipeline used in the study does not generate individual gene annotations; instead, it only produced intermediate alignments of evidence. Here we compared these intermediate alignments to the final gene set. Please refer to the attached table for details.

		intermediate	supporting final gene set
augustus_masked	match	68925	34751
blastn	expressed_sequence_match	1133759	28229
blastx	protein_match	1210717	37443
exonerate-est2genome	expressed_sequence_match	1825147	28718
exonerate-protein2genome	protein_match	1044717	36579

- Line 343 : what is "certain other databases of M. sinica" ?

Response: Thank you, we have revised this and added the annotated percentages from several different databases, and these can be found in Supplementary Table 19.

"certain other databases, including Pfam (25,850, 59.46%), Coils (2,533, 5.83%), CDD (28,110, 64.70%), SMART (8,247, 18.97%) and others were annotated with IntrerProScan. (Table S19)".

- Line 343 : InterProScan (typo)

Response: It has been revised.

- Line 344 : 90 % BUSCO value seems very low for a modern assembly. What could explain such a low value ?

Response: Thank you. This was because previously we used an old version of BUSCO (v2). In the revision, we have used the last version BUSCO5 and the value improved significantly (97.9%). We have revised this text.

- Line 357-361 : How is it different from (or similar with) the other studies ?

Response: We have discussed the relationship between our research results and those from other studies in the discussion section.

- Line 381 : what could explain the very low mapping rate (~90%) of M. sinica against itself (same species) ?

Response: They are the same species according to the SNP tree and the chloroplast tree, so the low mapping rate of this individuals could be attributed to sequencing artifacts.

- Line 391 : the end of the sentence does not make sense.

Response: Thank you, we have deleted this.

- Line 440- 445 : Are these values significant ?

Response: Yes, these terms were significant, and we revised the expressions.

- Line 447-448 : There is also M. obovata / M. hypoleuca

Response: Thank you, we have added these.

- Line 631 : Is this script available ?

Response: Thank you, it is available, we still have this script. If you would like it, you are welcome to apply to write to the provided communication email and you will receive it soon.

- Table 1. contigs (typo)

Response: Thank you, we have revised this.

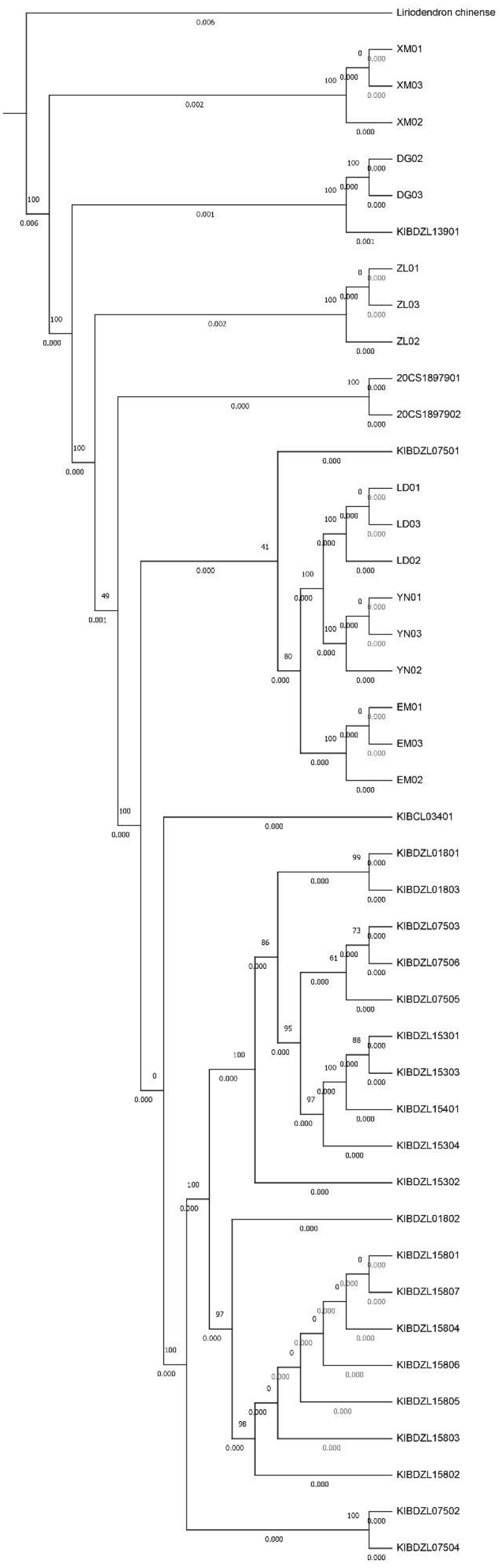

attached figure 1 chloroplast_tree

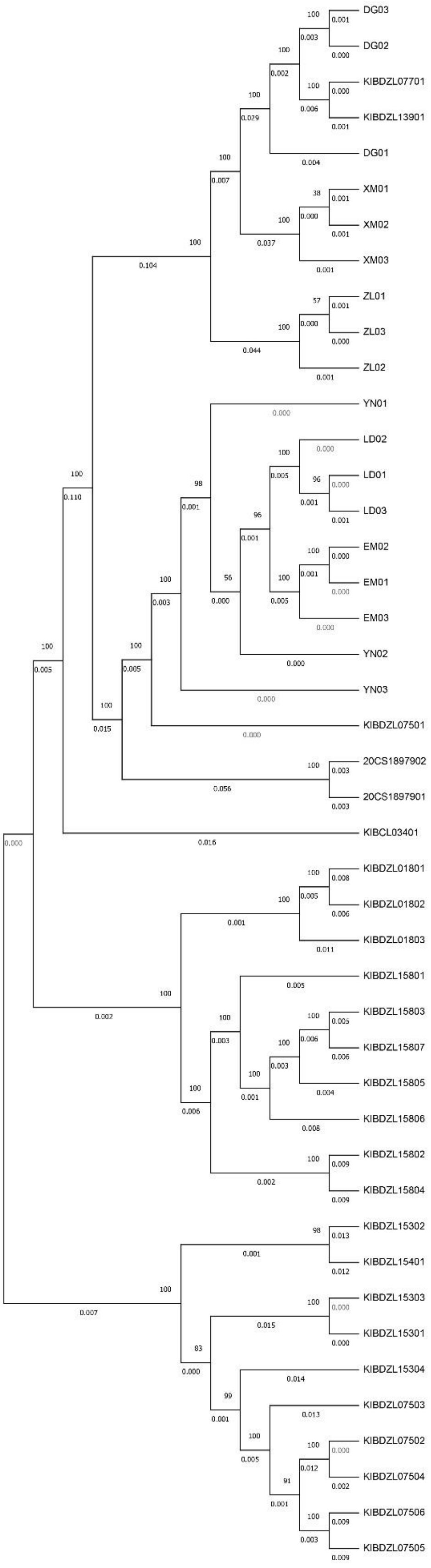

attached figure 2 SNP_tree

Reference

Cristofari R, Bertorelle G, Ancel A, et al. Full circumpolar migration ensures evolutionary unity in the Emperor penguin. *Nat Commun*. 2016;7:11842. doi: org/10.1038/ncomms11842.

Dai F, Zhuo X, Luo G et. al. Genomic Resequencing Unravels the Genetic Basis of Domestication, Expansion, and Trait Improvement in *Morus atropurpurea* [J]. *Adv. Sci*., 2023 [http://doi.org/10.1002/advs.202300039]

Fukushima K, Pollock DD. Detecting macroevolutionary genotype–phenotype associations using error-corrected rates of protein convergence [J]. *Nat Ecol Evol*. 2023;7: 155–170. doi: org/10.1038/s41559-022-01932-7.

Hanson-Smith V, Kolaczkowski B, Thornton JW. Robustness of Ancestral Sequence Reconstruction to Phylogenetic Uncertainty [J]. *Mol Biol Evol*. 2010;27 (9):1988–1999. Doi: org/10.1093/molbev/msq081.

Liu S, Zhang L, Sang Y et. al. Demographic History and Natural Selection Shape Patterns of Deleterious Mutation Load and Barriers to Introgression across *Populus* Genome [J]. *Mol. Biol. Evol*., 2022, 39 (2). [http://doi.org/10.1093/molbev/msac008]

Ma H, Liu YB, Liu DT, et al. Chromosome-level genome assembly and population genetic analysis of a critically endangered rhododendron provide insights into its conservation [J]. *Plant J*. 2021;107(5):1533–45. doi: 10.1111/tpj.15399.

Ma Z, Zhang Y, Wu L et. al. High-quality genome assembly and resequencing of modern cotton cultivars provide resources for crop improvement [J]. *Nat. Genet*., 2021 [http://doi.org/10.1038/s41588-021-00910-2]

Molloy E K, Warnow T. To Include or Not to Include: The Impact of Gene Filtering on Species Tree Estimation Methods [J]. *Syst. Biol*., 2017, 67 (2): 285–303 [http://doi.org/10.1093/sysbio/syx077]

Salojärvi J, Smolander OP, Nieminen K. et al. Genome sequencing and population genomic analyses provide insights into the adaptive landscape of silver birch [J]. *Nat Genet*. 2017;49:904–912. doi: org/10.1038/ng.3862.

Shekhar S, Roch S, Mirarab S. Species Tree Estimation Using ASTRAL: How Many Genes Are Enough? [J]. *IEEE/ACM Transactions on Computational Biology and Bioinformatics*, 2018, 15 (5): 1738–1747 [http://doi.org/10.1109/TCBB.2017.2757930]

Wang P, Zhou G, Jian J et. al. Whole-genome assembly and resequencing reveal genomic imprint and key genes of rapid domestication in narrow-leafed lupin [J]. *Plant J*., 2021, 105 (5): 1192–1210 [http://doi.org/10.1111/tpj.15100]

Zhao XF, Ma YP, Sun WB, et al. High genetic diversity and low differentiation of *Michelia coriacea* (Magnoliaceae), a critically endangered endemic in southeast Yunnan, China [J]. *Int J Mol Sci*. 2012;13(4):4396–411. doi:https://doi.org/10.3390/ijms13044396.
