# Supplementary material for: The chromosome-scale genome of Magnolia sinica (Magnoliaceae) provides insights into the conservation of plant species with extremely small populations (PSESP)
Source: Gigascience. 2024 Jan 11;13:giad110. doi: 10.1093/gigascience/giad110 (PMC10999834; doi:10.1093/gigascience/giad110)
Supplement: giad110_Supplemental_Files [file giad110_supplemental_files.zip › Supplementary file-Figures 20230728.docx]

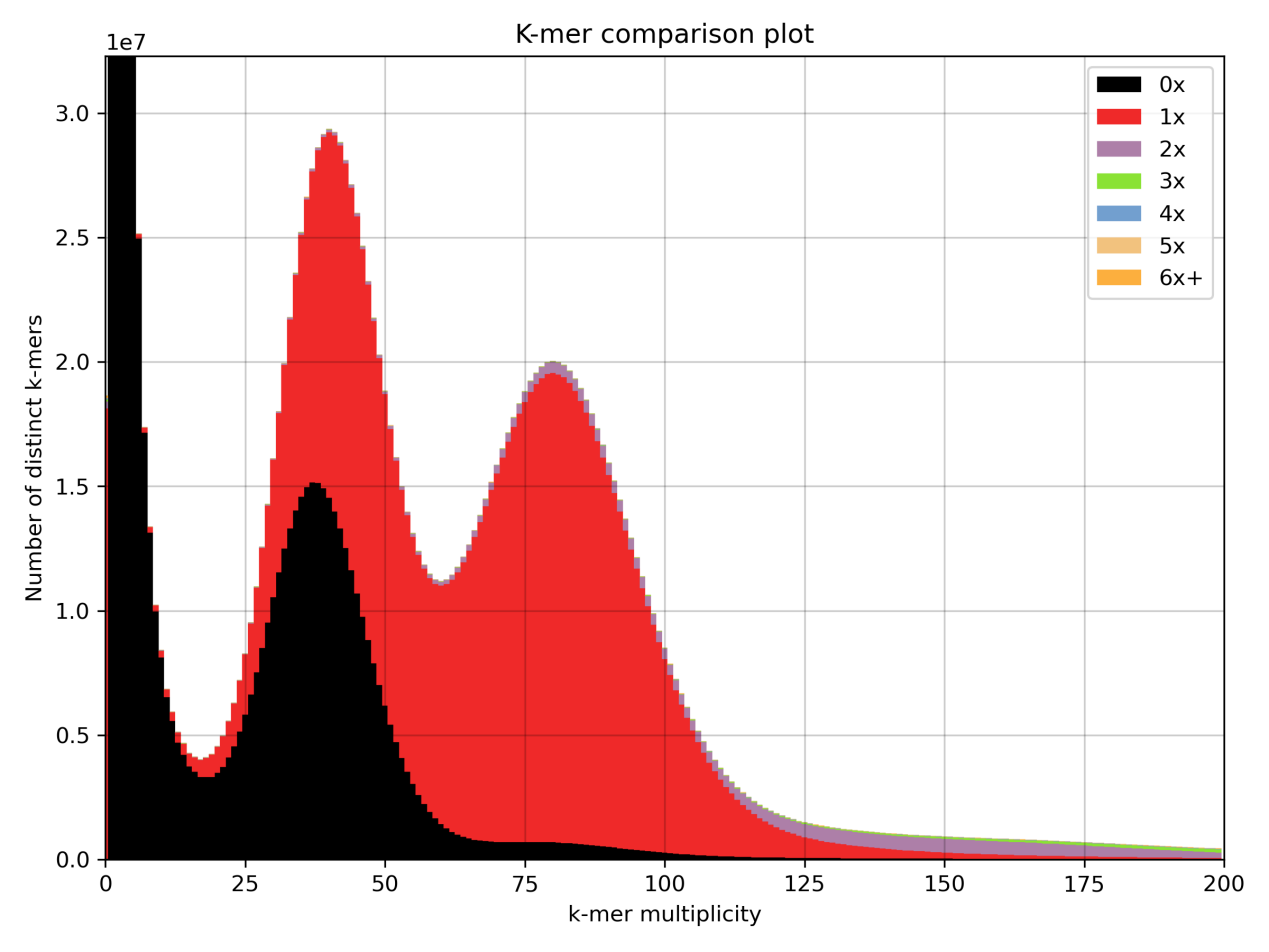


**Fig S1** K-mer spectrum analysis. Comparison of 27-mer spectra between Illumina reads and genome assembly.

| 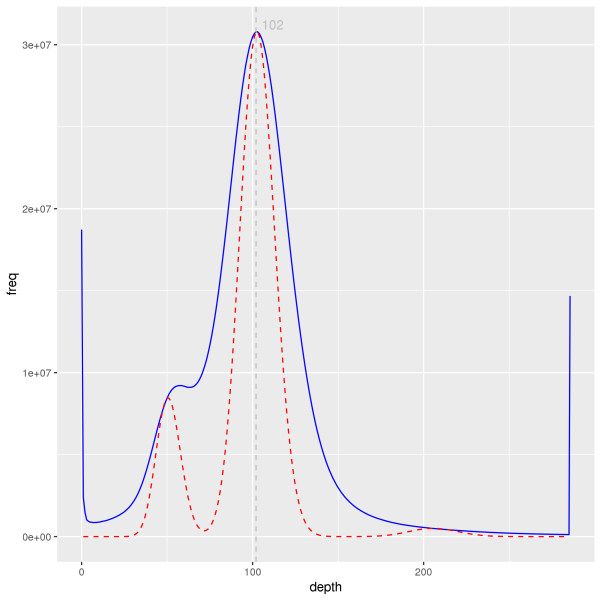 | 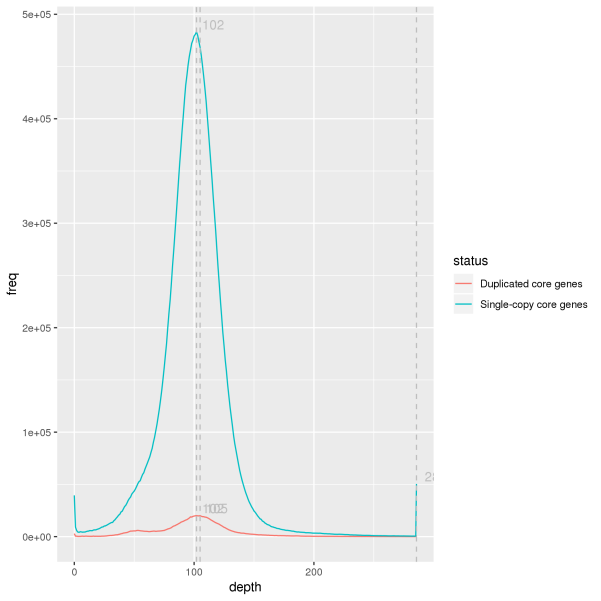 |
| --- | --- |
| 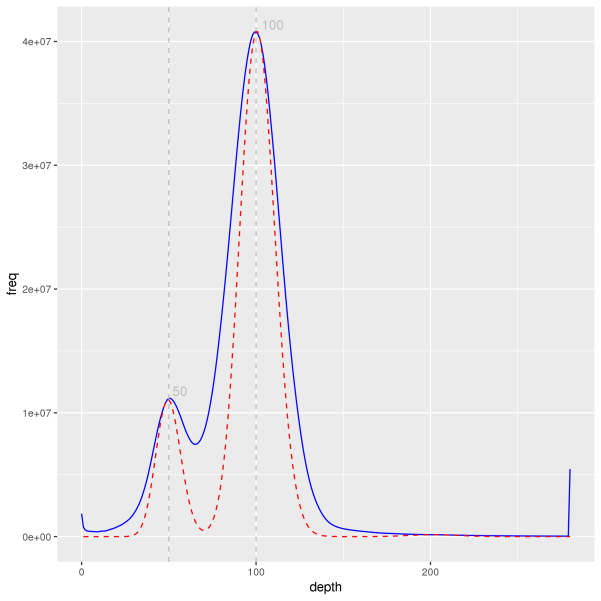 | 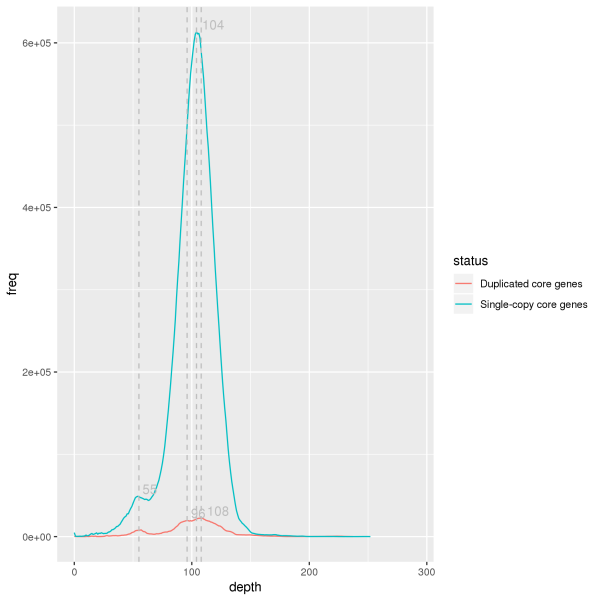 |

**Figure S2** Evaluation of the distribution of coverage depth over the whole genome and the BUSCO core gene region with Illumina and ONT data.


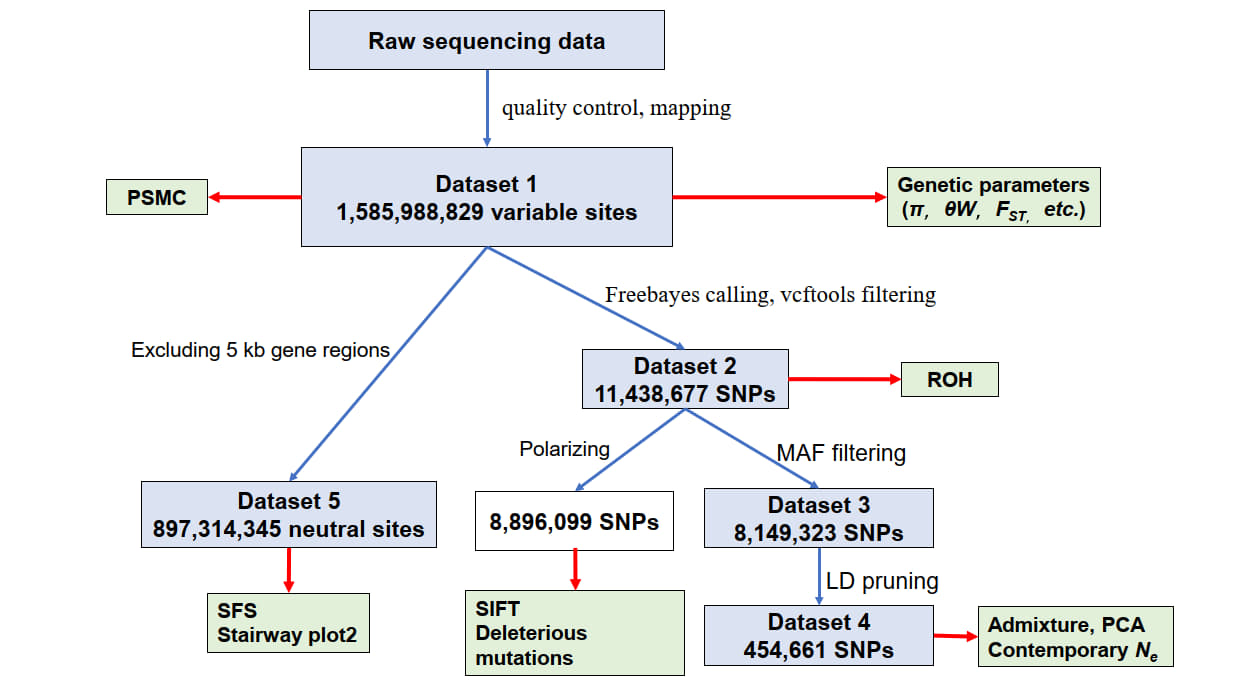


**Fig S3** A schematic diagram showing how these datasets were generated


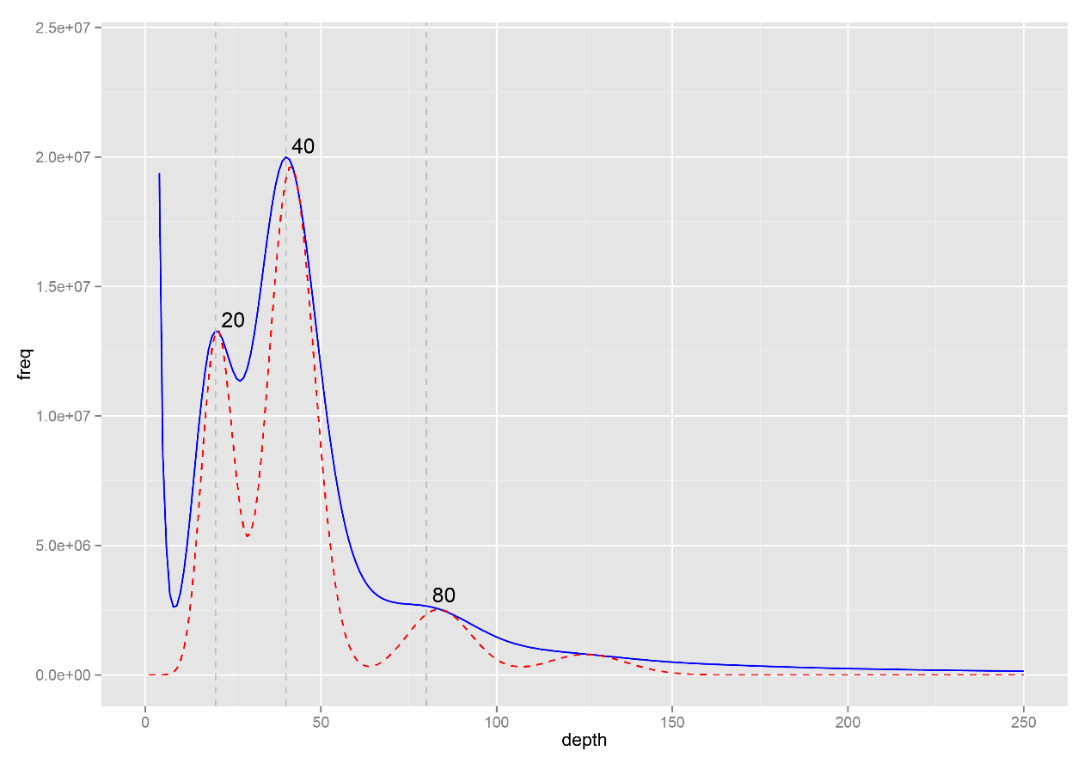


**Figure S4** Kmer frequency distribution diagram. The horizontal axis is the coverage depth, and the vertical axis is the corresponding kmer frequency. The blue solid line is 17bp-kmer distribution, with obvious main peak (d=40), heterozygous peak (d=20) and repetitive peak (double repetitive peak: d=80, etc.). The heterozygous peak is large, and the repetitive peak indicates that there may be genome replication events. The red dotted line is the theoretical Poisson distribution.


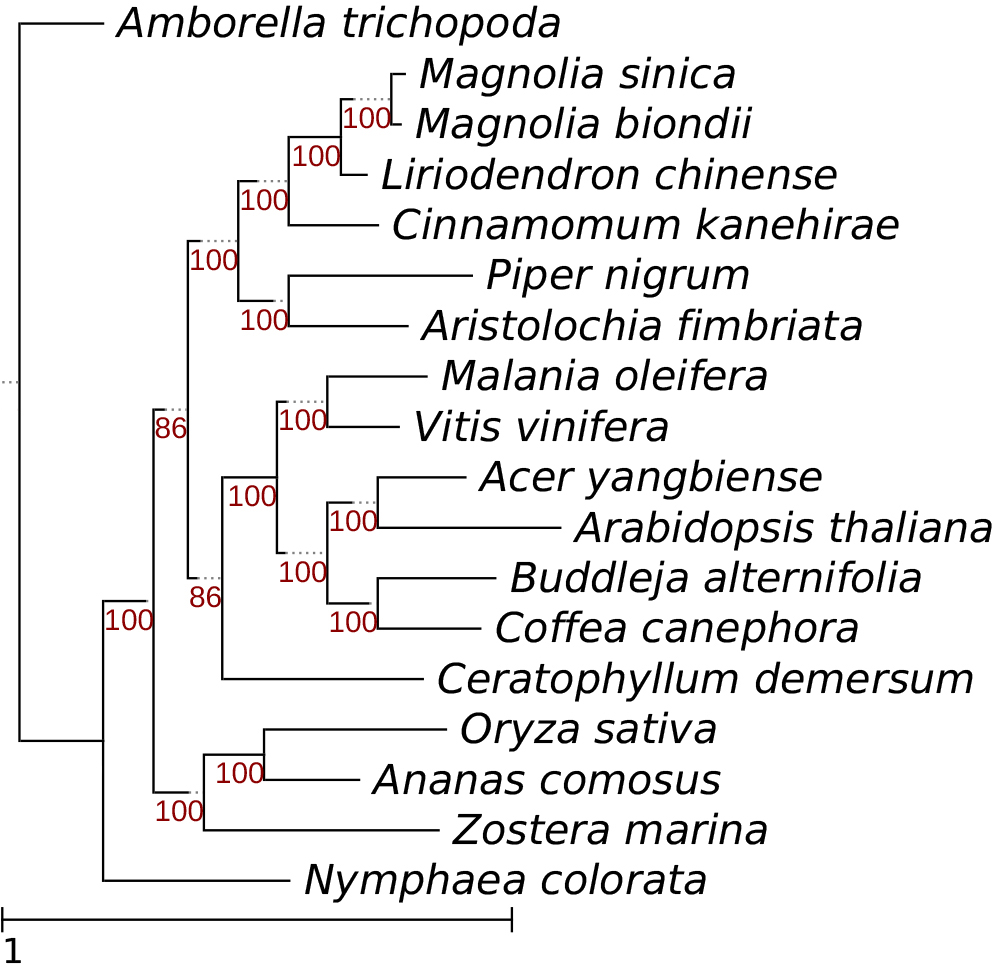


**Fig S5** ML phylogeny of *Magnolia sinica* and related taxa showing bootstrap values. Bar, substitution per site.


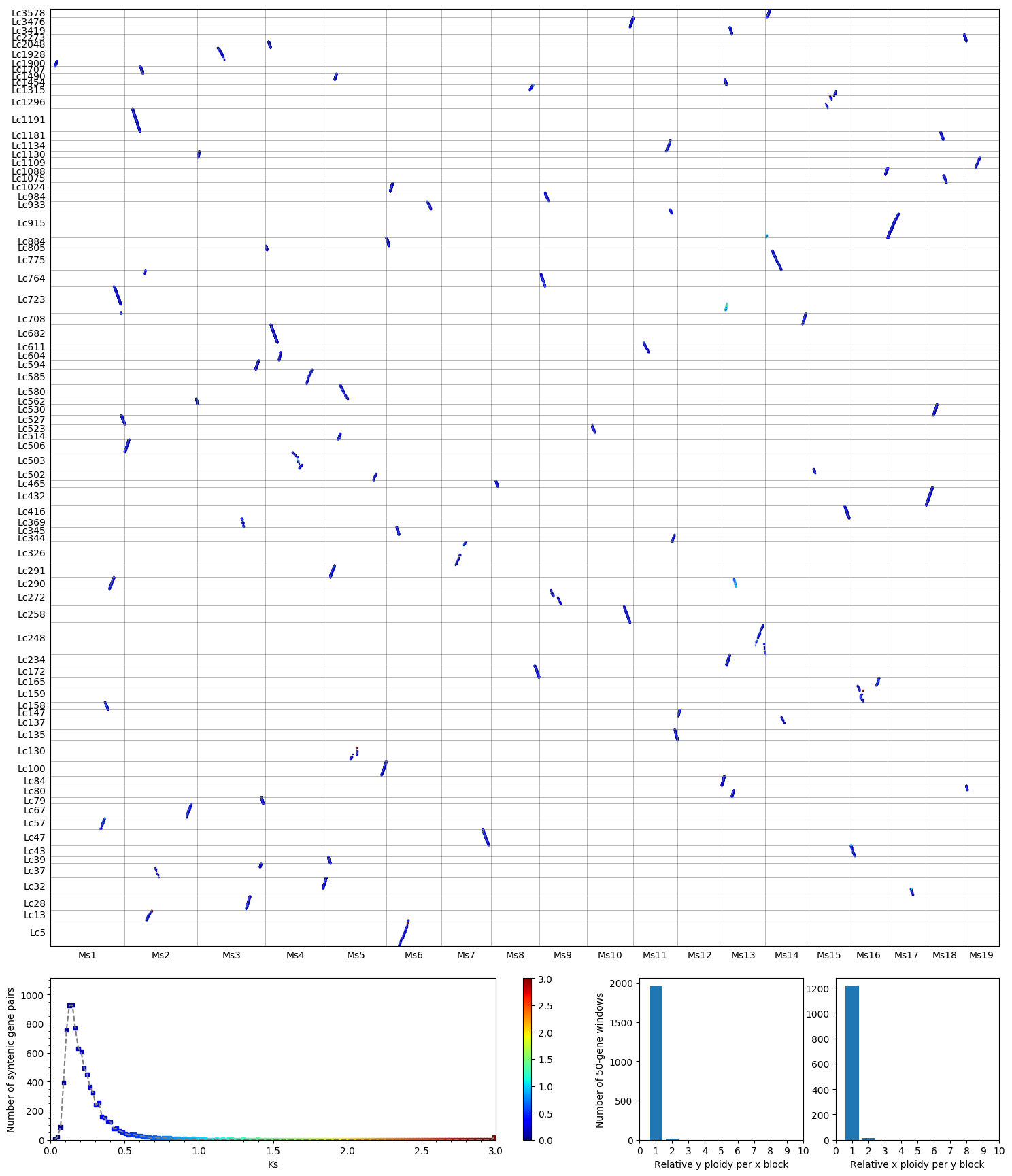


**Figure S6** The collinearity between *M. sinica* and *Liriodendron chinense* (1:1)


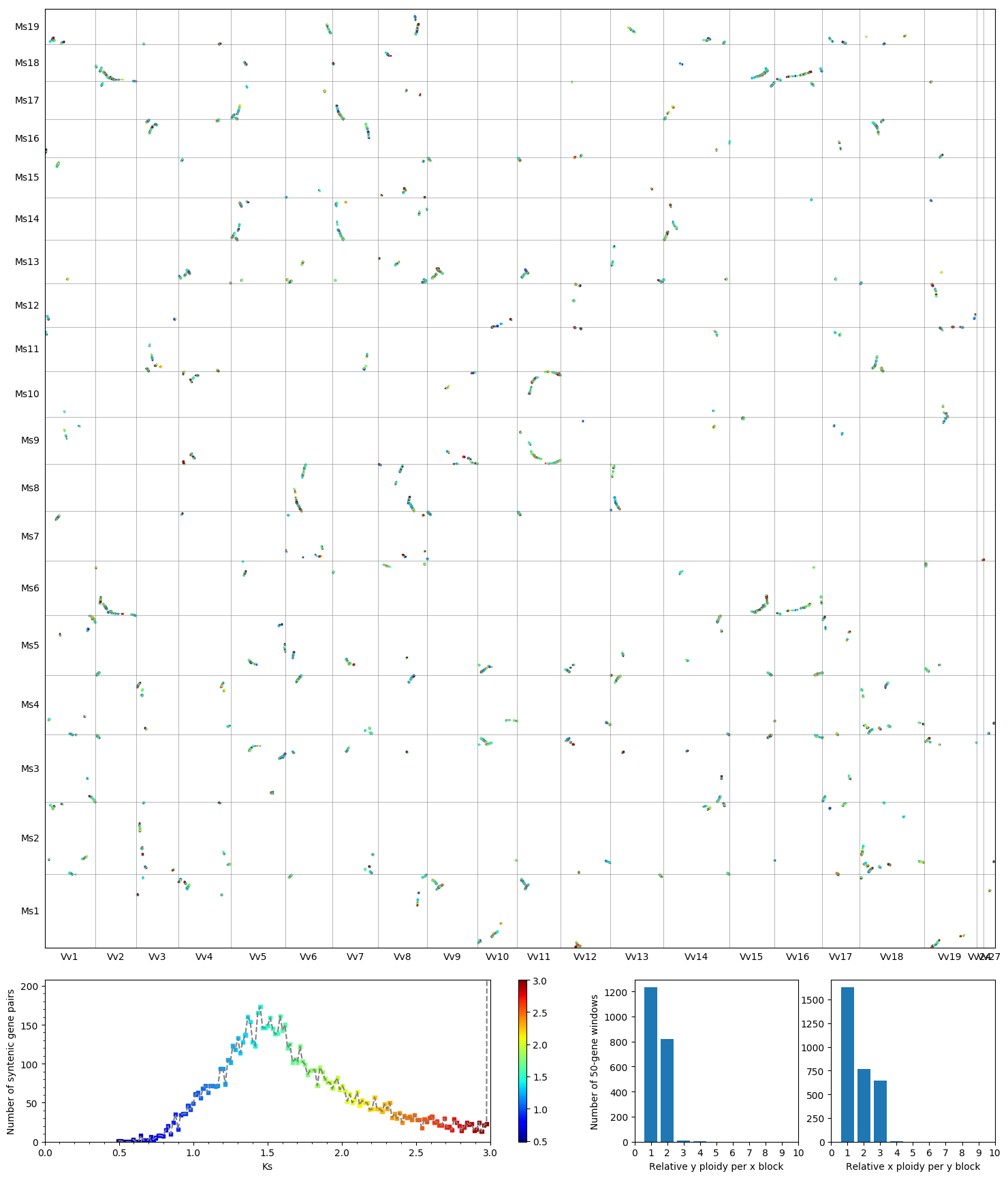


**Figure S7** The collinearity between *M. sinica* and *Vitis vinifera* (2:3)


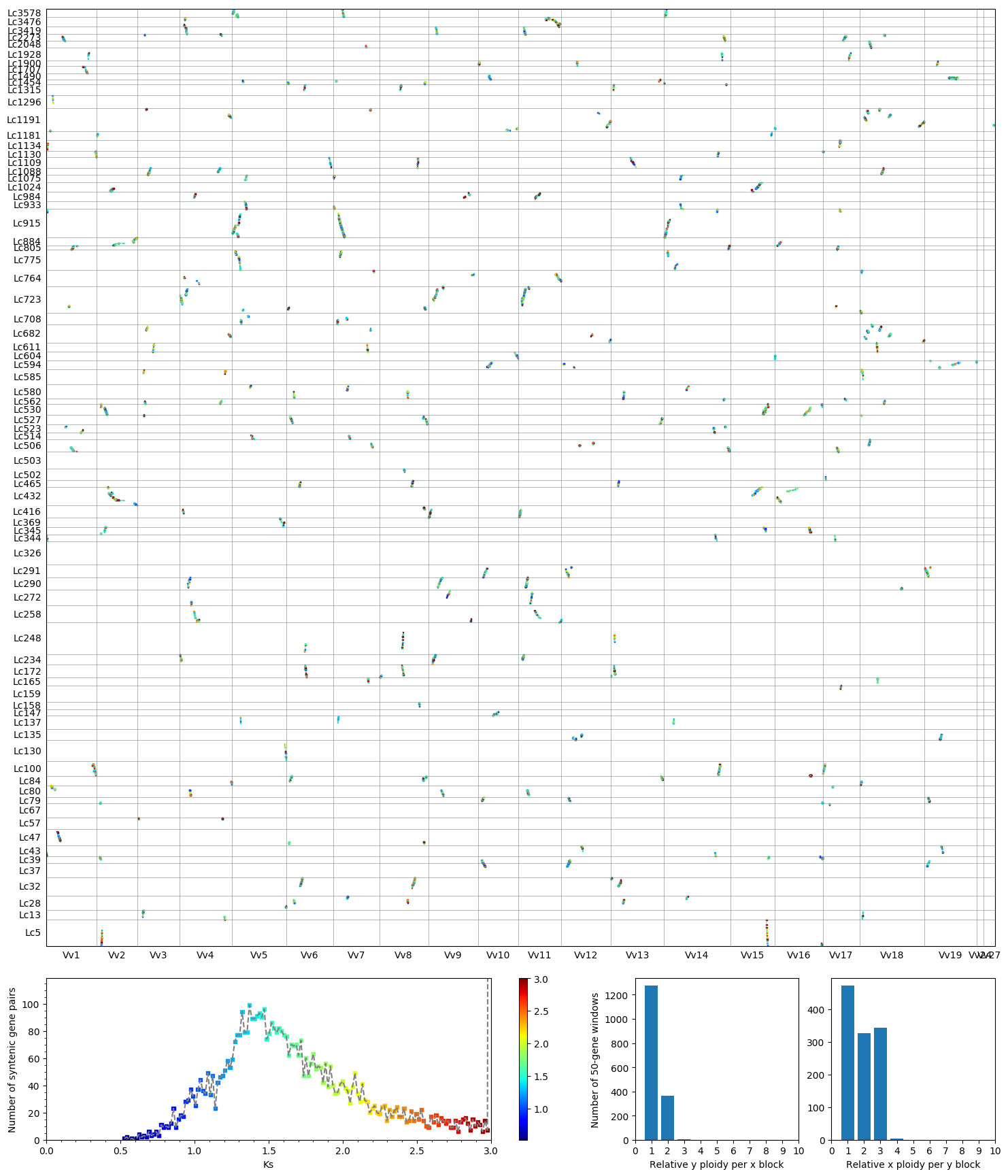


**Figure S8** The collinearity between *Liriodendron chinense* and *Vitis vinifera* (2:3)


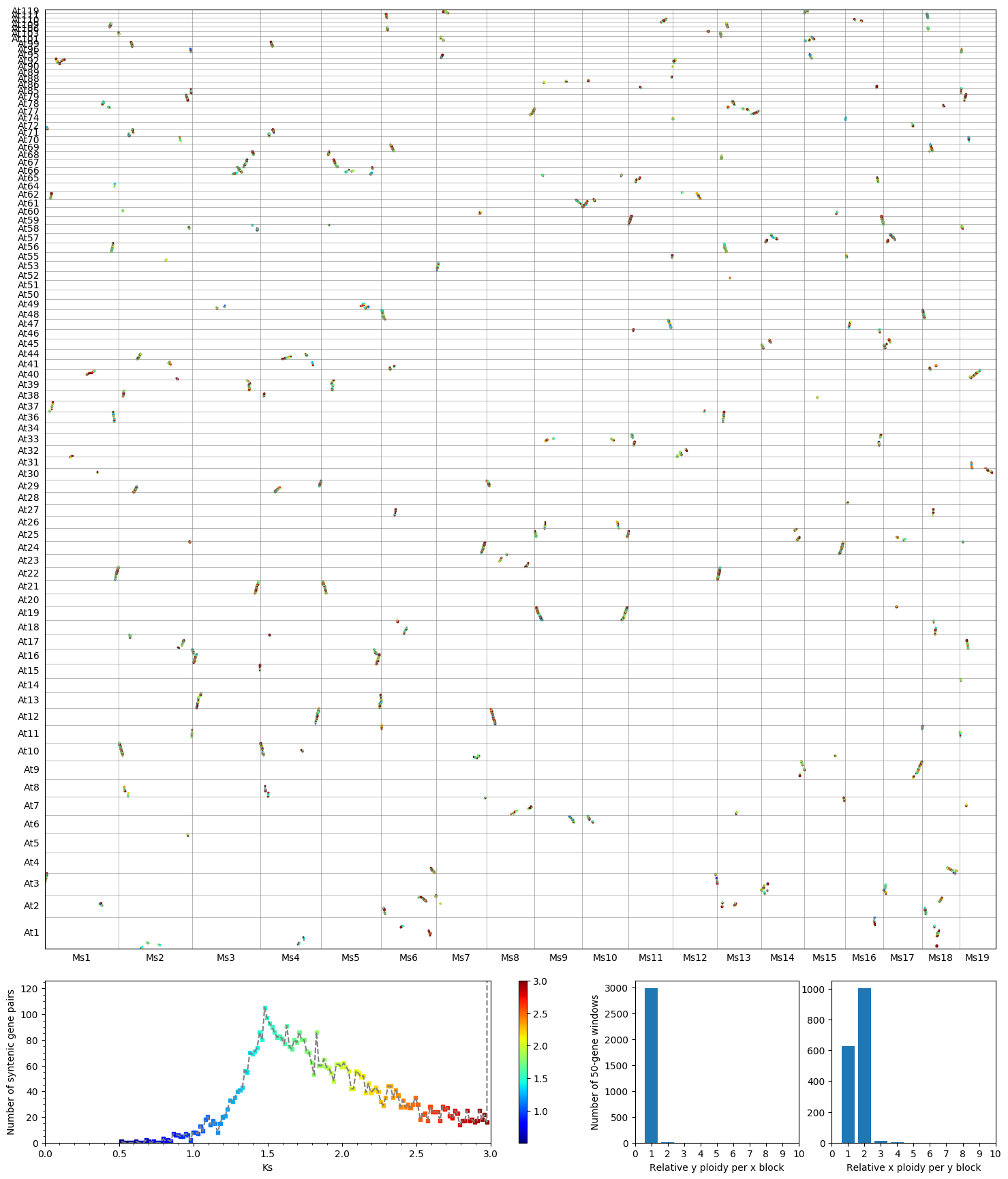


**Figure S9** The collinearity between *Amborella trichopoda* and *M. sinica*(1:2)


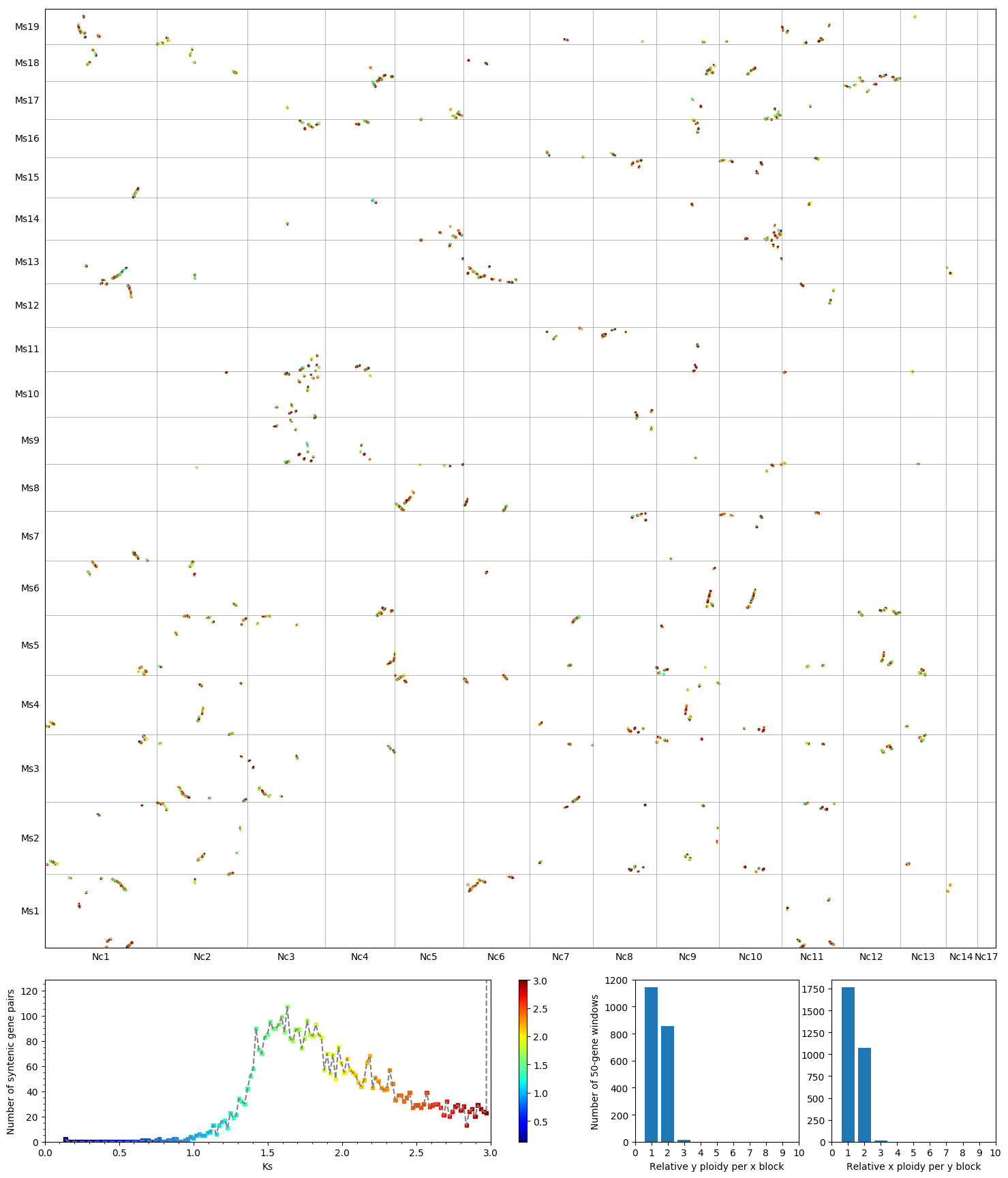


**Figure S10** The collinearity between *Nymphaea colorata* and *M. sinica*(2:2)


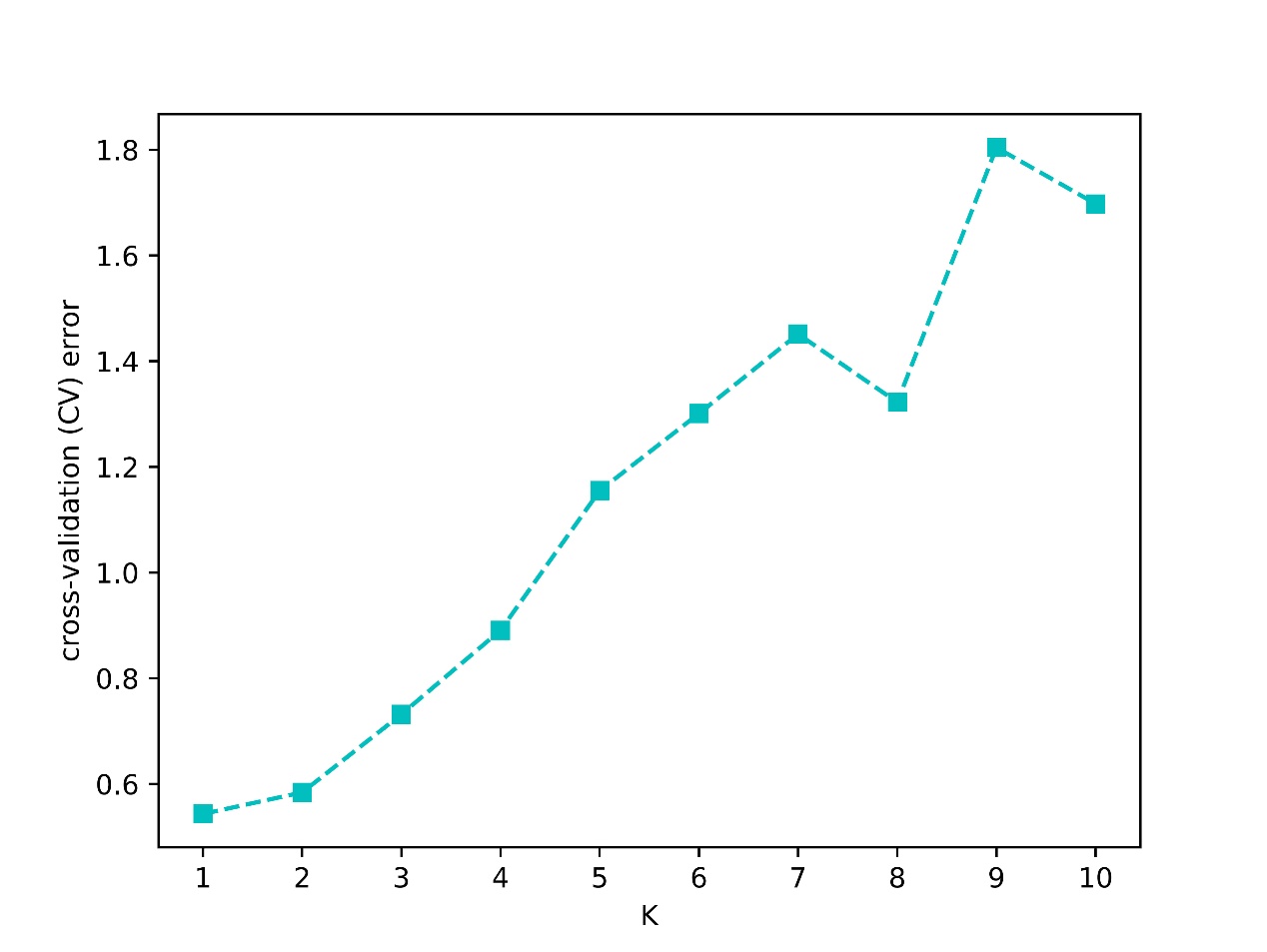
 **Figure S11** Cross validation error (CV) based on Admixture output.


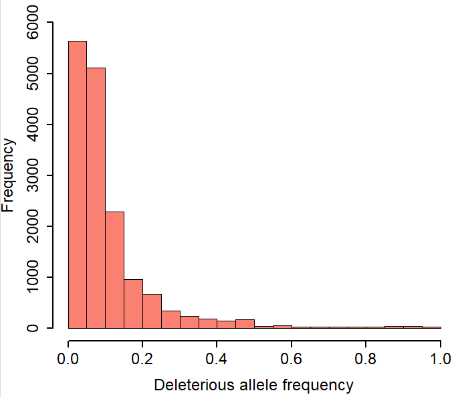


**Figure S12** Deleterious allele frequency distribution of homozygous deleterious SNPs. The density on the left of y axis is the number of alleles in a given allele frequency.
