# Supplementary material for: The chromosome-scale genome of Magnolia sinica (Magnoliaceae) provides insights into the conservation of plant species with extremely small populations (PSESP)
Source: Gigascience. 2024 Jan 11;13:giad110. doi: 10.1093/gigascience/giad110 (PMC10999834; doi:10.1093/gigascience/giad110)
Supplement: giad110_Response_to_Reviewer_Comments_Original_Submission [file giad110_response_to_reviewer_comments_original_submission.pdf]

Author's Response To Reviewer Comments

Comments to the editor and reviewers
Dear the Editor of GigaScience,
Thank you very much for editing this manuscript entitled “The chromosome-scale genome of Magnolia sinica (Magnoliaceae) provides insights into the conservation of plant species with extremely small populations (PSESP)” and making suggestions. We are also very grateful for the efforts of the two reviewers. We have revised the manuscript carefully according to their comments and have made responses listed below.
We have accepted most of the comments from the two reviewers, made revisions to the errors that occurred, added some relevant analyses, and have responded to and explained a small portion of the questions. 1) We have added discussions of the coexistence of high genetic diversity and low genetic differentiation to the manuscript in the DISCUSSION part. 2) We have added relevant supplementary figures with bootstrap values in the phylogenetic tree (Figure S5). 3) We have added parameters and we have added KAT analysis. 4) We have released all the data produced to date (https://www.ncbi.nlm.nih.gov/bioproject/PRJNA774088). 5) We have explained why the whole genome sequencing and transcriptome sequencing (RNA-seq) analyses did not use material from the same individual, and also explained why only 21 individuals were re sequenced. Please review the specific revisions and responses.
We resubmit the revised manuscript and we hope this version is now suitable for the publication in GigaScience. If you have any further questions or requirements, please do not hesitate to contact the corresponding author (MYP).
Yours sincerely,
Yongpeng Ma (corresponding authors on behalf of all authors).
26th JULY 2023

Reviewer #1: In this paper, authors reported the first genome of a critically endangered species Magnolia sinica. This large tree is widely known as "giant pandas in plants" due to its extremely rare individuals in wild, thus is under the first-class state protection in China. Here, authors obtained a high-quality chromosome-level genome assembly via combining Illumina, PacBio and Hi-C sequencing data.
Authors mainly focus on the population resequencing, showing a high genetic diversity of M. sinica population but a low genetic differentiation among subpopulations. Authors provide some explanations for each result. I wonder if author can discuss the potential connections between these two observed phenomenons. In addition, authors detected many deleterious mutations which were mostly related to lipids. Authors didn't mention this result in the DISCUSSION part. Are these deleterious mutations related to lipids results of or reasons for the endangered status of this species? Authors may provide further discussions or even conclusive evidences to clearly elucidate point of view this issue.
Response: Thank you for your suggestion. We now added discussions of coexistence of high genetic diversity and low genetic differentiation to the manuscript in the DISCUSSION part as below:
“M. sinica has a pollinator-dependent outcrossing mating system, which may contribute to its high genetic diversity; while high gene flow among populations may maintain links between populations of this species, and may contribute to its low genetic differentiation. The recent reduction in population size due to anthropogenic activities has led to isolation of the populations, leading to the high genetic diversity and low genetic differentiation now observed in the fragmented populations of this endangered tree species. Similar patterns have been reported in Michelia coriacea, another species in the Magnoliaceae [131].”
Regarding the deleterious mutations related to lipids, we could not conclude whether they were the results of or the reasons for the endangered status of Magnolia sinica, and we have therefore deleted the parts of the GO and KEGG anotations and enrichment analysis regarding deleterious mutations from the manuscript.

Reference
Zhao X, Ma Y, Sun W, et al. (2012) High genetic diversity and low differentiation of Michelia coriacea (Magnoliaceae), a critically endangered endemic in southeast Yunnan, China. International Journal of Molecular Sciences, 13(4): 4396–4411.
Minor concerns:

1. Introduction part: authors should point out what's the major limitations of the current protection of Huagaimu. And how a reference genome helps to overcome such limitations.
Response: Thank you. We have added the first part in the manuscript. And, the second part was included in last paragraph of the introduction as below.
“Although a great deal of protection and research action has been carried out, the lack of natural regeneration and genetic rescue still limits the protection of M. sinica. Therefore, the formulation of genetic rescue strategies for M. sinica will benefit greatly from the exploration of harmful cumulative mutations, population historical dynamics and effective population size from the whole genome level.
Here, we report a high-quality chromosome-scale genome sequence of Magnolia sinica, and compare it with other relevant published genomic data. By exploring the evolution of the genome, as well as the genetic characteristics, demographic history and genetic load of M. sinica, we have identified genomic factors that may contribute to the threats to this species, and, on the basis of this, we propose further strategies for the conservation of M. sinica.”
2. Magnolia sinica was first occurred in Line 79 in the main text and it should be written as M. sinica afterwards.
Response: Thank you. We have checked and revised this.
3. Line 206: "integrated annotated protein" should be "integrated annotated proteins".
Response: Thank you. We have revised this.
4. Line 222-224: References were needed here.
Response: Thank you. We have added relevant references.
5. Line 253: "θW" should be "θw".
Response: Thank you. We have revised this.
6. Fig. 2c, there shouldn't be a "_" within species name. And, bootstrap values should be indicated in the phylogenetic tree. In addition, Fig. 2 contained different results with no obvious connections. I do recommend to layout the content of this figure, focusing on one particular theme.
Response: Thank you. We now deleted the "_" within species name. We have added a relevant supplementary figure with the bootstrap values in the phylogenetic tree, please check (Figure S5). Because of the large number of figures in the manuscript, we have tried to save space and have given the figures (genomic character and genome evolution), where related figures are merged into one plate and explanations are provided separately.
7. No title was found in Fig. 3. Authors should give a strong title that reflects the major finding of this figure.
Response: Thank you. We have added a title (Distribution map, population structure, demographic history and Venn diagram of Magnolia sinica) for this Figure 3.

Reviewer #2: This manuscript described the assembly and analyses of the chromosome-scale genome assembly for Magnolia sinica, an endangered Magnoliaceae species. Despite the authors provided a useful piece of work, it can still be greatly improved. In particular, it needs a thorough proofing to clarify many points in the Material & Methods section, as well as in results.

However, a major interrogation is the rational of resequencing only 21 M. sinica and 22 other Magnolia, while there is only 52 remaining M. sinica in the wild. I think it would have shown a much complete picture to generate data for all (known) individuals in the species.
Response: Thank you for your questions. In 2019, we only re-sequenced the materials that we had collected (21 samples). These materials included samples from all populations and covered the full range of the Magnolia sinica distribution, representing >40% of all M. sinica individuals. Because the collection of these materials took a lot of money and time, considering the cost of re-collection and the expensive re-sequencing costs at the time, we were unable to collect material from more individuals. Furthermore, based on the preliminary analysis of our sequencing data, we found that there were no significant differences (such as genetic diversity or genetic structure) compared to previous population studies based on SSR (Chen 2017, in Chinese). Therefore, we only sequenced 21 individuals of M. sinica from that time. The phylogenetic position of M. sinica has always been controversial, so we chose to sequence 22 samples from other eight Magnolia species. We have provided the relevant chloroplast tree (attached figure 1 chloroplast tree) and SNPs tree (attached figure 2 SNP_tree) as attachments at the bottom of this file.

I noticed several mistakes in the description of used data and methods. For example:
(1) line 21 the authors mentioned using Pacbio data for genome assembly, but from the Material &

Methods, they used only ONT data to generate long reads for assembly
Response: We have revised this mistake.
(2) they mentioned a QiaGen kit that seems to not exist in Material & Methods
line 149 they mentioned using Pilon to modifiy - correct? - Illumina reads; should be the opposite
Response: The reagent kit with product number 13323, Qiagen, is available. Genomic DNA kit (cat. no. 13323. Qiagen, Hilden, Germany). Please check: https://www.qiagen.com/us/products/discovery-and-translational-research/dna-rna-purification/dna-purification/genomic-dna/blood-and-cell-culture-dna-kits. We have corrected the description of correcting with Illumina reads.
(3) Parameters used for pipelines are missing in several part of the manuscript
Also, the usually used metrics and quality assessment methods were not used here; I would appreciate to get a Merqury / KAT/ GenomeScope analysis in addition to the BUSCO and LAI.
Response: We have added parameters and a KAT analysis.

Also, I don't really understand why the authors performed RNAseq for annotation from a different individual, instead of using the same individual as for the genome assembly.
Response: Thank you. We understand your concern regarding this issue, unfortunately we faced some challenges during this project. In 2019, when we started sequencing, leaf samples were initially sent to a company in dry ice for genome sequencing. Later in 2020, when we collected multiple tissues for RNA-seq, it became very difficult to send samples rapidly in dry ice because of special policies (special periods of COVID-19). Therefore, for simplicity, we decided to directly send a living seedling (including leaf, stem, root tissues, but excluding other tissues such as flowers) and fresh fruits at room temperature (without dry ice) for RNA-seq. Therefore, the RNAseq and genome assembly analyses were conducted using different individuals. However, because we used the PacBio platform to sequence the full-length cDNA, the variations between individuals should have very limited negative effects on gene annotation. In fact, 99.5% PacBio CCS reads were mapped to the genome.
The ancestral sequence reconstruction part appeared quite weak with the method used, not taking into account the emergence of potentially large Structural Variations (SVs) across the chromosomes during their evolutions. I would suggest, if the authors want to keep this part to use a more robust approach (e.g. based on Salse, 2021 approach)
Response: Thank you for your suggestion. We agree that the emergence of SV may influence the reconstruction of ancestral state. However, SV is difficult to detect from our short reseqencing reads. Here we used an empirical Bayesian method based on posterior probability of the sites to reconstruct ancestral sequence. This method can produce accurate reconstruction of the ancestral sequence (Hanson-Smith et al. 2010) and has been previously used to reconstruct the ancestral state in other works (Cristofari et al., 2016; Salojärvi et al., 2017; Ma et al., 2021; Fukushima et al., 2023). We apologize for not being able to find the article by “Salse, 2021”. After explaining our method above, if it is necessary to use Salse's approach, could you please provide us more information about it and give us another chance to revise it?
References
Cristofari R, Bertorelle G, Ancel A, et al. Full circumpolar migration ensures evolutionary unity in the Emperor penguin. Nat Commun. 2016;7:11842. doi: org/10.1038/ncomms11842.
Fukushima K, Pollock DD. Detecting macroevolutionary genotype–phenotype associations using error-corrected rates of protein convergence. Nat Ecol Evol. 2023;7: 155–170. doi: org/10.1038/s41559-022-01932-7.
Hanson-Smith V, Kolaczkowski B, Thornton JW. Robustness of Ancestral Sequence Reconstruction to Phylogenetic Uncertainty. Mol Biol Evol. 2010;27 (9):1988–1999. Doi: org/10.1093/molbev/msq081.
Ma H, Liu YB, Liu DT, et al. Chromosome-level genome assembly and population genetic analysis of a critically endangered rhododendron provide insights into its conservation. Plant J. 2021;107(5):1533–45. doi: 10.1111/tpj.15399.
Salojärvi J, Smolander OP, Nieminen K. et al. Genome sequencing and population genomic analyses provide insights into the adaptive landscape of silver birch. Nat Genet. 2017;49:904–912. doi: org/10.1038/ng.3862.
The data accessibility is also questionable, as the authors mentioned the BioProject PRJNA774088, that is already cited by a published paper, but not accessible
Response: We apologize that the data were not released earlier. The data have now been completely released (https://www.ncbi.nlm.nih.gov/bioproject/PRJNA774088). A copy of the data can be found in China National Center for Bioinformation (https://ngdc.cncb.ac.cn/bioproject/browse/PRJCA015437).

Specific comments:
- Line 21 : Only ONT data were combined with short reads to assemble the genome ;
Response: Sorry, we have revised this mistake.
- Line 59 : please add the date when the database have been accessed ;
Response: Thank you. We have corrected this and added the access dates.
- Line 93-97 : this seems more adequate for a Data Notes than for a research article ;
Response: Thank you, this is indeed only a partial summary. Here, we not only reported the high-quality chromosome-scale genome sequence of Magnolia sinica and re-sequenced 21 samples of the same species and 22 samples from other species, but also investigated genome evolution, genome-wide diversity, and population structure of this species, inferred its demographic history, and estimated its genetic load and inbreeding level. We further discussed the possible reason for its high genetic diversity but low genetic differentiation, the climatic, tectonic and anthropogenic explanation of its demographic history, the likely genetic basis of the extremely small populations, and provided conservation measures based on our findings. We think it is worthy of a research article.
- Line 107 : dry ice temperature is -78.5°C
Response: We have revised this mistake.
- Line 118 : this kit does not exist (the reference number is for an other kit)
Response: We have revised this. The Genomic DNA kit (cat. no. 13323. Qiagen, Hilden, Germany) is available, and this kit can also extract genomic DNA from diverse materials. The kit was also used to extract plant DNA after treatment of CTAB.
- Line 121 : more details are needed for the library construction method. What was the DNA input ? any modification from the ONT protocol ? barcoded library or not ?
Response: The DNA input was total genomic DNA. The ONT protocol was not modified, and the library was not barcoded.
- Line 124 : please choose the machine the library was run on (or precise which library was run on which machine) ; how many flowcells ?
Response: PromethION was used yielding 7 flowcells. This has been added to the manuscript.
- Line 126 : what fragment size for the Illumina library
Response: We have added insertion size of 300–500 bp.
- Line 130 : what was considered as "high molecular weight DNA" ?
Response: This refers to longer and more complete DNA with high "molecular weight".
- Line 147: please precise what assembly strategies did you used (= assemblers ?)
Response: Thank you, we have added a descriptions of the assembly method.
- Line 148 : this reference is for the Celera assembler only, did you use it ?
Response: No. We have revised the text.
- Line 149 : short reads were used to correct long reads, not the opposite ;
Response: Thank you, this has been revised.
- Line 151 : how they were polished ?
Response: The method has been added.
- Line 151 : please described the parameters used in GetOrganelles to assemble both the mitochondrial genome and plastome
Response: The parameters have been added.
- Line 159 : "scaffolded" instead of "scattered" ?
Response: This has been revised as "un-anchored" meaning contigs that were not anchored onto chromosomes.
- Line 161 : what parameters for LR_Gapcloser and NextPolish ?
Response: The parameters have been added.
- Line 163 : Redundant (typo)
Response: It has been revised.
- Line 165 : what is the NT library ?
Response: The NT library is NT database from NCBI for BLAST (https://ftp.ncbi.nlm.nih.gov/blast/db/). We have revised this in the text for clarification.
- Line 167 : how low was a coverage considered ?
Response: We have revised this in the text.
- Line 172-183 : see above for addition of QC pipelines results
Response: We have added KAT analysis.
- Line 189 : how these two libraries were combined ?

Response: We concatenated the two libraries (fasta files) directly using the Linux command `cat`.
- Line 194 : Considering Magnoliaceae position in angiosperms, I think it could be useful to add at least one monocots in the annotation process (e.g. the wheat or maize, or rice genome)
Response: Thank you for your suggestion. We tested this by adding the wheat genome, and found only 551 new genes (1.3% more than before) predicted by the MAKER2 pipeline. We also tested it with the Aristolochia fimbriata (Piperales) genome as evidence, and 1419 genes (3.3% more) were newly identified. It appears that more protein evidences would certainly produce more genes, but considering the improvements (1.3-3.3% more genes) are quite limited and would not significantly affect our downstream conclusions regarding comparative and conservation genomics, we chose to not include the update in the revision.
- Line 201 : Augustus is usually used as an ab initio annotator ; please specify more in details how you used it the integrate previous annotations
Response: Yes, Augustus is an ab initio annotator, but it supports biological evidence (hint file from transcript and protein alignments) as input for better prediction. This step is integrated in the MAKER2 pipeline. We have revised the text for a clearer description.
- Line 217, 220, 222 : why there is a discrepancy between the single-copy gene numbers ?
Response: We used different cutoffs to allow for missing data. For the ASTRAL method, more genes are better with high ILS (incomplete lineage sorting) level, and missing data are more tolerated (References below), so we used more genes with higher missing rate (30%). For the IQTREE method, missing data are moderately tolerated, so we used the dataset with moderate missing rate (12.5%; the dataset was generated in OrthoFinder2 to infer a species tree in its pipeline). MCMCtree uses only non-missing data by default, so we just included 1:1 orthologous single-copy genes (with none missing). Different dataset may provide cross-validations to reduce sampling bias. We have added detailed descriptions.
References:
Molloy E K, Warnow T. To Include or Not to Include: The Impact of Gene Filtering on Species Tree Estimation Methods [J]. Syst. Biol., 2017, 67 (2): 285–303 [http://doi.org/10.1093/sysbio/syx077]
Shekhar S, Roch S, Mirarab S. Species Tree Estimation Using ASTRAL: How Many Genes Are Enough? [J]. IEEE/ACM Transactions on Computational Biology and Bioinformatics, 2018, 15 (5): 1738–1747 [http://doi.org/10.1109/TCBB.2017.2757930]

- Line 235 : Why not using the 52 M. sinica individuals (see above) ?
Response: Thank you for your questions. In 2019, we only re-sequenced the materials that we had collected (21 samples). These materials included samples from all populations, and covered the full range of the Magnolia sinica distribution, representing >40% of all M. sinica individuals. Because the collection of these materials took a lot of money and time, considering the cost of re-collection and the expensive re-sequencing costs at the time, we were unable to collect material from more individuals. Furthermore, based on the preliminary analysis of our sequencing data, we found that there were no significant differences (such as genetic diversity or genetic structure) compared to previous population studies based on SSR (Chen 2017, in Chinese). Therefore, we only sequenced 21 individuals of M. sinica from that time.
- Line 241 : sequences with quality score <20 should not be found in the clean reads (from line 238)
Response: After filtering with fastp, the proportion of sequences with a quality score <20 decreases, however, there are still some bases with a quality score <20. Fastp trims reads using a sliding window, but did not trim all bases with a quality score <20. Thus, we excluded the potentially retained bases with quality score <20 in downstream analysis (ANGSD and freebayes).
- Line 242 : considering a sequencing depth ranging from 8.8X to 12.6X for M. sinica (max 14.3X for other Magnolia), it seems unrealistic to remove sites with a mapping depth <100X
Response: The depth of sites refers to the sum of all samples, but not average depth across samples. The distribution of the depth of sites is as follows. The peak value is at 331x, so empirically the upper limit is set to 600x, about twice that of the peak, and the lower limit is about 1/3 of the peak. We have revised the text to make this clear.

- Line 243 : please specify how these sites were retained
Response: We have described this in more detail in the paper.
- Line 248 : why the authors did not use the widely used 10% missing data threshold?
Response: Thank you for your question. We wanted to balance the threshold and the number of SNPs. Considering that there are many species, a stricter threshold would lead to fewer SNPs, which may be not have been sufficient for downstream analyses. In fact, the threshold of 20% or higher has also been used

in previous studies (References below).
References:
Liu S, Zhang L, Sang Y et. al. Demographic History and Natural Selection Shape Patterns of Deleterious Mutation Load and Barriers to Introgression across Populus Genome [J]. Mol. Biol. Evol., 2022, 39 (2) [http://doi.org/10.1093/molbev/msac008]
Dai F, Zhuo X, Luo G et. al. Genomic Resequencing Unravels the Genetic Basis of Domestication, Expansion, and Trait Improvement in Morus Atropurpurea [J]. Adv. Sci., 2023 [http://doi.org/10.1002/advs.202300039]
Wang P, Zhou G, Jian J et. al. Whole-genome assembly and resequencing reveal genomic imprint and key genes of rapid domestication in narrow-leafed lupin [J]. Plant J., 2021, 105 (5): 1192–1210 [http://doi.org/10.1111/tpj.15100]
Ma Z, Zhang Y, Wu L et. al. High-quality genome assembly and resequencing of modern cotton cultivars provide resources for crop improvement [J]. Nat. Genet., 2021 [http://doi.org/10.1038/s41588-021-00910-2]
- Line 249 : due to both the relatively low number of indiviuals and the large part of the sampling made of other Magnolia species, such a classic MAF value would results in removing SNPs present in 1 or 2 samples, making them potentially diagnostic of a given species
Response: We did not aim to make diagnostic of a given species, so the species-specific SNPs were not necessary for our analyses. In the phylogenetic tree based on the filtered SNPs (attached figure 2 SNP_tree), each species has formed a separate monophyletic clade, suggesting that our filtering with the classic MAF value did not obscure the relationships among these species.
- Line 250 and following : Please described more in details, but concisely, how these different datasets are made, and how they are each useful (at least more useful than only one or two datasets)
Response: We apologized for the imprecise and incorrect descriptions. We have revised this and have also added an additional schematic diagram to the supplementary figures to illustrate it.
- Line 309 : please add the parameters used
Response: Thank you, we have added these.
- Line 319 : did the authors considered flow cytometry to get a (more) accurate estimate of the genome size ? Considering the patrimonial value of the species, it could be valuable.
Response: Thank you. At that time, the Genome size of Magnolia sinica was estimated by k-mer analysis of the Illumina sequencing data. This method is widely used and is sufficiently accurate, so we felt that we did not need to use an experimental method based on Flow Cytometry.
- Line 327 : Did the authors compared the LAI value obtained here with other Magnolia genome assemblies ?
Response: Thank you. We could not compare the relevant LAI values of several Magnolia species because of the other three genomic articles did not calculate this value.
- Line 335-336 : Please add values for gene annotations from transcriptomic, ab initio and similarity approaches separately, then indicate how many were supported, filtered and so on, with the final value.
Response: The MAKER annotation pipeline used in the study does not generate individual gene annotations; instead, it only produced intermediate alignments of evidence. Here we compared these intermediate alignments to the final gene set. Please refer to the attached table for details.

- Line 343 : what is "certain other databases of M. sinica" ?
Response: Thank you, we have revised this and added the annotated percentages from several different databases, and these can be found in Supplementary Table 19.
"certain other databases, including Pfam (25,850, 59.46%), Coils (2,533, 5.83%), CDD (28,110, 64.70%), SMART (8,247, 18.97%) and others were annotated with IntrerProScan. (Table S19)".
- Line 343 : InterProScan (typo)
Response: It has been revised.
- Line 344 : 90 % BUSCO value seems very low for a modern assembly. What could explain such a low value ?
Response: Thank you. This was because previously we used an old version of BUSCO (v2). In the revision, we have used the last version BUSCO5 and the value improved significantly (97.9%). We have revised this text.
- Line 357-361 : How is it different from (or similar with) the other studies ?
Response: We have discussed the relationship between our research results and those from other studies in the discussion section.

- Line 381 : what could explain the very low mapping rate (~90%) of M. sinica against itself (same species) ?
Response: They are the same species according to the SNP tree and the chloroplast tree, so the low mapping rate of this individuals could be attributed to sequencing artifacts.
- Line 391 : the end of the sentence does not make sense.
Response: Thank you, we have deleted this.
- Line 440- 445 : Are these values significant ?
Response: Yes, these terms were significant, and we revised the expressions.
- Line 447-448 : There is also M. obovata / M. hypoleuca
Response: Thank you, we have added these.
- Line 631 : Is this script available ?
Response: Thank you, it is available, we still have this script. If you would like it, you are welcome to apply to write to the provided communication email and you will receive it soon.
- Table 1. contigs (typo)
Response: Thank you, we have revised this.

attached figure 1 chloroplast_tree attached figure 2 SNP_tree

Reference

Cristofari R, Bertorelle G, Ancel A, et al. Full circumpolar migration ensures evolutionary unity in the Emperor penguin. Nat Commun. 2016;7:11842. doi: org/10.1038/ncomms11842.
Dai F, Zhuo X, Luo G et. al. Genomic Resequencing Unravels the Genetic Basis of Domestication, Expansion, and Trait Improvement in Morus atropurpurea [J]. Adv. Sci., 2023 [http://doi.org/10.1002/advs.202300039]
Fukushima K, Pollock DD. Detecting macroevolutionary genotype–phenotype associations using error-corrected rates of protein convergence [J]. Nat Ecol Evol. 2023;7: 155–170. doi: org/10.1038/s41559-022-01932-7.
Hanson-Smith V, Kolaczkowski B, Thornton JW. Robustness of Ancestral Sequence Reconstruction to Phylogenetic Uncertainty [J]. Mol Biol Evol. 2010;27 (9):1988–1999. Doi: org/10.1093/molbev/msq081.
Liu S, Zhang L, Sang Y et. al. Demographic History and Natural Selection Shape Patterns of Deleterious Mutation Load and Barriers to Introgression across Populus Genome [J]. Mol. Biol. Evol., 2022, 39 (2). [http://doi.org/10.1093/molbev/msac008]
Ma H, Liu YB, Liu DT, et al. Chromosome-level genome assembly and population genetic analysis of a critically endangered rhododendron provide insights into its conservation [J]. Plant J. 2021;107(5):1533–45. doi: 10.1111/tpj.15399.
Ma Z, Zhang Y, Wu L et. al. High-quality genome assembly and resequencing of modern cotton cultivars provide resources for crop improvement [J]. Nat. Genet., 2021 [http://doi.org/10.1038/s41588-021-00910-2]
Molloy E K, Warnow T. To Include or Not to Include: The Impact of Gene Filtering on Species Tree Estimation Methods [J]. Syst. Biol., 2017, 67 (2): 285–303 [http://doi.org/10.1093/sysbio/syx077]
Salojärvi J, Smolander OP, Nieminen K. et al. Genome sequencing and population genomic analyses provide insights into the adaptive landscape of silver birch [J]. Nat Genet. 2017;49:904–912. doi: org/10.1038/ng.3862.
Shekhar S, Roch S, Mirarab S. Species Tree Estimation Using ASTRAL: How Many Genes Are Enough? [J]. IEEE/ACM Transactions on Computational Biology and Bioinformatics, 2018, 15 (5): 1738–1747 [http://doi.org/10.1109/TCBB.2017.2757930]
Wang P, Zhou G, Jian J et. al. Whole-genome assembly and resequencing reveal genomic imprint and key genes of rapid domestication in narrow-leafed lupin [J]. Plant J., 2021, 105 (5): 1192–1210 [http://doi.org/10.1111/tpj.15100]
Zhao XF, Ma YP, Sun WB, et al. High genetic diversity and low differentiation of Michelia coriacea (Magnoliaceae), a critically endangered endemic in southeast Yunnan, China [J]. Int J Mol Sci. 2012;13(4):4396–411. doi:https://doi.org/10.3390/ijms13044396.
