# Supplementary material for: The chromosome-scale genome of Magnolia sinica (Magnoliaceae) provides insights into the conservation of plant species with extremely small populations (PSESP)
Source: Gigascience. 2024 Jan 11;13:giad110. doi: 10.1093/gigascience/giad110 (PMC10999834; doi:10.1093/gigascience/giad110)
Supplement: giad110_Reviewer_2_Report_Original_Submission — Damien Hinsinger -- 5/3/2023 Reviewed [file giad110_reviewer_2_report_original_submission.pdf]

Reviewer Report

Title: The chromosome-scale genome of Magnolia sinica (Magnoliaceae) provides insights into the conservation of plant species with extremely small populations (PSESP)

Version: Original Submission **Date:** 5/3/2023

Reviewer name: Damien Hinsinger

Reviewer Comments to Author:

This manuscript described the assembly and analyses of the chromosome-scale genome assembly for Magnolia sinica, an endangered Magnoliaceae species.
Despite the authors provided a useful piece of work, it can still be greatly improved. In particular, it needs a thorough proofing to clarify many points in the Material & Methods section, as well as in results.
However, a major interrogation is the rational of resequencing only 21 M. sinica and 22 other Magnolia, while there is only 52 remaining M. sinica in the wild. I think it would have shown a much complete picture to generate data for all (known) individuals in the species.
I noticed several mistakes in the description of used data and methods. For example :
- line 21 the authors mentioned using Pacbio data for genome assembly, but from the Material & Methods, they used only ONT data to generate long reads for assembly
- they mentioned a QiaGen kit that seems to not exist in Material & Methods
- line 149 they mentioned using Pilon to modifiy - correct ? - Illumina reads ; should be the opposite
- Parameters used for pipelines are missing in several part of the manuscript
Also, the usually used metrics and quality assessment methods were not used here ; I would appreciate to get a Merqury / KAT/ GenomeScope analysis in addition to the BUSCO and LAI.
Also, I don't really understand why the authors performed RNAseq for annotation from a different individual, instead of using the same individual as for the genome assembly.
The ancestral sequence reconstruction part appeared quite weak with the method used, not taking into account the emergence of potentially large Structural Variations (SVs) across the chromosomes during their evolutions. I would suggest, if the authors want to keep this part to use a more robust approach (e.g. based on Salse, 2021 approach)
The data accessibility is also questionable, as the authors mentioned the BioProject PRJNA774088, that is already cited by a published paper, but not accessible
Specific comments :
- Line 21 : Only ONT data were combined with short reads to assemble the genome ;
- Line 59 : please add the date when the database have been accessed ;
- Line 93-97 : this seems more adequate for a Data Notes than for a research article ;
- Line 107 : dry ice temperature is -78.5Â°C
- Line 118 : this kit does not exist (the reference number is for an other kit)
- Line 121 : more details are needed for the library construction method. What was the DNA input ? any modification from the ONT protocol ? barcoded library or not ?

- Line 124 : please choose the machine the library was run on (or precise which library was run on which machine) ; how many flowcells ?
- Line 126 : what fragment size for the Illumina library
- Line 130 : what was considered as "high molecular weight DNA" ?
- Line 147: please precise what assembly strategies did you used (= assemblers ?)
- Line 148 : this reference is for the Celera assembler only, did you use it ?
- Line 149 : short reads were used to correct long reads, not the opposite ;
- Line 151 : how they were polished ?
- Line 151 : please described the parameters used in GetOrganelles to assemble both the mitochondrial genome and plastome
- Line 159 : "scaffolded" instead of "scattered" ?
- Line 161 : what parameters for LR_Gapcloser and NextPolish ?
- Line 163 : Redundant (typo)
- Line 165 : what is the NT library ?
- Line 167 : how low was a coverage considered ?
- Line 172-183 : see above for addition of QC pipelines results
- Line 189 : how these two libraries were combined ?
- Line 194 : Considering Magnoliaceae position in angiosperms, I think it could be useful to add at least one monocots in the annotation process (e.g. the wheat or maize, or rice genome)
- Line 201 : Augustus is usually used as an ab initio annotator ; please specify more in details how you used it the integrate previous annotations
- Line 217, 220, 222 : why there is a discrepancy between the single-copy gene numbers ?
- Line 235 : Why not using the 52 M. sinica individuals (see above) ?
- Line 241 : sequences with quality score <20 should not be found in the clean reads (from line 238)
- Line 242 : considering a sequencing depth ranging from 8.8X to 12.6X for M. sinica (max 14.3X for other Magnolia), it seems unrealistic to remove sites with a mapping depth <100X
- Line 243 : please specify how these sites were retained
- Line 248 : why the authors did not use the widely used 10% missing data threshold ?
- Line 249 : due to both the relatively low number of indiviuals and the large part of the sampling made of other Magnolia species, such a classic MAF value would results in removing SNPs present in 1 or 2 samples, making them potentially diagnostic of a given species
- Line 250 and following : Please described more in details, but concisely, how these different datasets are made, and how they are each useful (at least more useful than only one or two datasets)
- Line 309 : please add the parameters used
- Line 319 : did the authors considered flow cytometry to get a (more) accurate estimate of the genome size ? Considering the patrimonial value of the species, it could be valuable
- Line 327 : Did the authors compared the LAI value obtained here with other Magnolia genome assemblies ?
- Line 335-336 : Please add values for gene annotations from transcriptomic, ab initio and similarity approaches separately, then indicate how many were supported, filtered and so on, with the final value.
- Line 343 : what is "certain other databases of M. sinica" ?
- Line 343 : InterProScan (typo)

- Line 344 : 90 % BUSCO value seems very low for a modern assembly. What could explain such a low value ?
- Line 357-361 : How is it different from (or similar with) the other studies ?
- Line 381 : what could explain the very low mapping rate (~90%) of M. sinica against itself (same species) ?
- Line 391 : the end of the sentence does not make sense.
- Line 440- 445 : Are these values significant ?
- Line 447-448 : There is also M. obovata / M. hypoleuca
- Line 631 : Is this script available ?
- Table 1. contigs (typo)

Methods

Are the methods appropriate to the aims of the study, are they well described, and are necessary controls included? Choose an item.

Conclusions

Are the conclusions adequately supported by the data shown? Choose an item.

Reporting Standards

Does the manuscript adhere to the journal's guidelines on minimum standards of reporting? Choose an item.

Choose an item.

Statistics

Are you able to assess all statistics in the manuscript, including the appropriateness of statistical tests used? Choose an item.

Quality of Written English

Please indicate the quality of language in the manuscript: Choose an item.

Declaration of Competing Interests

Please complete a declaration of competing interests, considering the following questions:

- Have you in the past five years received reimbursements, fees, funding, or salary from an organisation that may in any way gain or lose financially from the publication of this manuscript, either now or in the future?
- Do you hold any stocks or shares in an organisation that may in any way gain or lose financially from the publication of this manuscript, either now or in the future?

- Do you hold or are you currently applying for any patents relating to the content of the manuscript?
- Have you received reimbursements, fees, funding, or salary from an organization that holds or has applied for patents relating to the content of the manuscript?
- Do you have any other financial competing interests?
- Do you have any non-financial competing interests in relation to this paper?

If you can answer no to all of the above, write 'I declare that I have no competing interests' below. If your reply is yes to any, please give details below.

I declare that I have no competing interests

I agree to the open peer review policy of the journal. I understand that my name will be included on my report to the authors and, if the manuscript is accepted for publication, my named report including any attachments I upload will be posted on the website along with the authors' responses. I agree for my report to be made available under an Open Access Creative Commons CC-BY license (http://creativecommons.org/licenses/by/4.0/). I understand that any comments which I do not wish to be included in my named report can be included as confidential comments to the editors, which will not be published.

Choose an item.

To further support our reviewers, we have joined with Publons, where you can gain additional credit to further highlight your hard work (see: https://publons.com/journal/530/gigascience). On publication of this paper, your review will be automatically added to Publons, you can then choose whether or not to claim your Publons credit. I understand this statement.

Yes Choose an item.
